# Supplementary material for: Damage evolution and constitutive model of the rock masses with non-penetrating cracks under repeated impact loading
Source: PLoS One. 2023 Sep 15;18(9):e0289022. doi: 10.1371/journal.pone.0289022 (PMC10503746; doi:10.1371/journal.pone.0289022)
Supplement: S1 Dataset — (DOCX) [file pone.0289022.s001.docx]

**Fig. 4 Waveforms of the cyclic-impact SHPB test of granite samples**

1 1.69874E-7 1.33133E-6 1.78815E-7 4.76209E-7 -9.46582E-6 -1.01783E-5 1.183E-9 -4.576E-9

2 -1.32505E-6 2.21218E-6 -1.39479E-6 1.21673E-6 -8.25717E-6 -9.16747E-6 -1.85638E-6 -1.39446E-6

3 -3.17173E-6 2.00891E-6 -3.33866E-6 2.42008E-6 -7.11563E-6 -8.58982E-6 -1.33628E-6 -1.85771E-6

4 -2.46823E-6 1.67013E-6 -2.59814E-6 1.86469E-6 -6.30989E-6 -8.01223E-6 -1.48467E-6 -1.32826E-6

5 -2.55616E-6 1.0603E-6 -2.6907E-6 1.95725E-6 -6.64563E-6 -8.22887E-6 -2.00485E-6 -9.31145E-7

6 -1.85267E-6 -2.27093E-7 -1.95018E-6 1.95725E-6 -7.11563E-6 -8.95089E-6 -1.41056E-6 -1.52681E-6

7 -2.29236E-6 1.33133E-6 -2.41301E-6 1.95725E-6 -7.38421E-6 -1.09725E-5 -1.0391E-6 -1.1297E-6

8 -8.85369E-7 1.46685E-6 -9.31967E-7 2.14238E-6 -8.52575E-6 -1.09002E-5 -1.0391E-6 -1.39446E-6

9 -1.23712E-6 9.92549E-7 -1.30223E-6 2.79034E-6 -9.26439E-6 -1.24887E-5 -1.11337E-6 -1.85771E-6

10 -2.73205E-6 1.33133E-6 -2.87584E-6 1.95725E-6 -9.60011E-6 -1.29941E-5 -2.00485E-6 -1.92392E-6

11 -2.55616E-6 2.27994E-6 -2.6907E-6 2.79034E-6 -9.8687E-6 -1.38605E-5 -1.41056E-6 -2.05627E-6

12 -1.41299E-6 4.39372E-8 -1.48736E-6 2.42008E-6 -9.06291E-6 -1.38605E-5 -1.78253E-6 -2.38717E-6

13 -1.85267E-6 1.80564E-6 -1.95018E-6 2.32751E-6 -9.46582E-6 -1.42938E-5 -1.78253E-6 -1.85771E-6

14 -1.23712E-6 1.80564E-6 -1.30223E-6 2.04982E-6 -9.06291E-6 -1.34273E-5 -2.59973E-6 -1.99013E-6

15 -2.11649E-6 1.67013E-6 -2.22788E-6 1.33827E-8 -8.72717E-6 -1.30663E-5 -1.70783E-6 -1.72537E-6

16 -1.32505E-6 2.95752E-6 -1.39479E-6 1.3093E-6 -9.19723E-6 -1.27053E-5 -2.07955E-6 -1.65916E-6

17 -2.02854E-6 2.95752E-6 -2.13531E-6 4.76209E-7 -8.72717E-6 -1.21999E-5 -1.26201E-6 -2.58573E-6

18 1.13718E-6 2.41545E-6 1.19703E-6 1.05948E-7 -8.72717E-6 -1.21277E-5 -9.64821E-7 -2.51959E-6

19 -4.45684E-7 1.33133E-6 -4.69141E-7 -3.56878E-7 -8.45859E-6 -1.18389E-5 -1.47368E-7 -2.05627E-6

20 -1.32505E-6 1.94115E-6 -1.39479E-6 1.49443E-6 -8.79433E-6 -1.16945E-5 -3.70279E-7 -2.85049E-6

21 -1.85267E-6 2.00891E-6 -1.95018E-6 -3.56878E-7 -8.92859E-6 -1.14057E-5 5.21365E-7 -2.65194E-6

22 -1.23712E-6 1.33133E-6 -1.30223E-6 -7.91825E-8 -9.19723E-6 -1.16223E-5 7.5543E-8 -2.51959E-6

23 -2.38029E-6 2.75425E-6 -2.50557E-6 1.05948E-7 -8.86149E-6 -1.14057E-5 -7.30925E-8 -2.18862E-6

24 -3.17173E-6 2.3477E-6 -3.33866E-6 -2.64313E-7 -8.92859E-6 -1.15501E-5 2.24179E-7 -2.38717E-6

25 -1.14918E-6 2.68649E-6 -1.20966E-6 1.21673E-6 -1.06073E-5 -1.08281E-5 1.49819E-7 -1.72537E-6

26 -1.06125E-6 2.21218E-6 -1.1171E-6 7.53904E-7 -1.10102E-5 -1.01783E-5 4.4709E-7 -1.92392E-6

27 -1.23712E-6 3.02528E-6 -1.30223E-6 -5.4201E-7 -1.13459E-5 -1.00339E-5 -2.96004E-7 -1.26205E-6

28 -1.41299E-6 3.22855E-6 -1.48736E-6 -1.71748E-7 -1.14131E-5 -1.06115E-5 -7.4191E-7 -4.01687E-7

29 -1.58887E-6 2.88976E-6 -1.67249E-6 8.46469E-7 -1.10102E-5 -1.05393E-5 -1.47368E-7 -7.32589E-7

30 -1.76474E-6 7.4295E-6 -1.85762E-6 1.49443E-6 -1.05402E-5 -1.16223E-5 -2.96004E-7 -4.67896E-7

31 -1.14918E-6 5.26128E-6 -1.20966E-6 2.51264E-6 -1.0473E-5 -1.20556E-5 1.183E-9 -5.34034E-7

32 -3.3476E-6 6.00662E-6 -3.52379E-6 2.42008E-6 -1.04059E-5 -1.31385E-5 6.70001E-7 -1.4606E-6

33 -1.85267E-6 5.19353E-6 -1.95018E-6 3.99368E-6 -1.03387E-5 -1.32829E-5 5.21365E-7 -1.32826E-6

34 -2.29236E-6 4.5837E-6 -2.41301E-6 5.0119E-6 -9.19723E-6 -1.21277E-5 -2.96004E-7 -1.79158E-6

35 -1.94061E-6 3.29631E-6 -2.04275E-6 4.45651E-6 -7.98853E-6 -1.18389E-5 -7.30925E-8 -2.38717E-6

36 -2.02854E-6 2.3477E-6 -2.13531E-6 4.17882E-6 -7.65285E-6 -1.21999E-5 2.24179E-7 -1.99013E-6

37 -1.6768E-6 3.22855E-6 -1.76505E-6 1.3093E-6 -6.91422E-6 -1.16223E-5 -7.4191E-7 -1.99013E-6

38 -4.13904E-6 2.68649E-6 -4.35688E-6 8.46469E-7 -7.45137E-6 -1.16223E-5 -6.6755E-7 -2.45338E-6

39 -2.38029E-6 1.8734E-6 -2.50557E-6 8.46469E-7 -8.86149E-6 -1.12613E-5 -5.18999E-7 -2.58573E-6

40 -2.11649E-6 2.21218E-6 -2.22788E-6 -3.56878E-7 -1.0003E-5 -1.06837E-5 1.183E-9 -2.18862E-6

41 -1.41299E-6 2.07667E-6 -1.48736E-6 -2.30075E-6 -1.10773E-5 -1.05393E-5 1.49819E-7 -2.38717E-6

42 -1.32505E-6 3.36406E-6 -1.39479E-6 -2.02305E-6 -1.16816E-5 -1.11891E-5 3.7273E-7 -2.38717E-6

43 -1.76474E-6 3.6351E-6 -1.85762E-6 -8.19704E-7 -1.13459E-5 -1.09002E-5 6.70001E-7 -2.85049E-6

44 -1.32505E-6 4.1094E-6 -1.39479E-6 -1.28253E-6 -1.07416E-5 -1.09725E-5 9.67187E-7 -3.2476E-6

45 -1.81874E-7 4.85473E-6 -1.91446E-7 -2.57844E-6 -1.06073E-5 -1.11891E-5 5.95641E-7 -2.65194E-6

46 -9.39363E-8 4.92249E-6 -9.88803E-8 1.40186E-6 -1.07416E-5 -1.14778E-5 -2.96004E-7 -3.31381E-6

47 2.80798E-6 2.82201E-6 2.95577E-6 1.33827E-8 -1.04059E-5 -1.22721E-5 -5.18999E-7 -3.31381E-6

48 5.21622E-7 2.68649E-6 5.49076E-7 1.21673E-6 -1.07416E-5 -1.27053E-5 -1.11337E-6 -3.44616E-6

49 -7.97432E-7 1.73788E-6 -8.39402E-7 2.32751E-6 -1.00701E-5 -1.33551E-5 -1.11337E-6 -2.58573E-6

50 -2.02854E-6 2.47211E-7 -2.13531E-6 2.32751E-6 -9.33153E-6 -1.34273E-5 -1.33628E-6 -2.71814E-6

51 -1.6768E-6 2.4721E-7 -1.76505E-6 2.32751E-6 -8.92859E-6 -1.33551E-5 -2.74819E-6 -1.79158E-6

52 -1.14918E-6 1.53461E-6 -1.20966E-6 2.8829E-6 -8.12291E-6 -1.32829E-5 -3.19376E-6 -1.99013E-6

53 -1.14918E-6 9.24784E-7 -1.20966E-6 1.77212E-6 -8.25717E-6 -1.4041E-5 -3.3053E-6 -1.75847E-6

54 -2.73205E-6 1.73788E-6 -2.87584E-6 2.42008E-6 -8.19001E-6 -1.34273E-5 -4.01172E-6 -1.72537E-6

55 -1.76474E-6 1.33133E-6 -1.85762E-6 3.25316E-6 -9.8687E-6 -1.27775E-5 -3.93711E-6 -6.00243E-7

56 -1.85267E-6 1.67013E-6 -1.95018E-6 9.39033E-7 -1.06073E-5 -1.20556E-5 -4.23413E-6 2.60189E-7

57 -2.11649E-6 1.26358E-6 -2.22788E-6 6.61339E-7 -1.17488E-5 -1.15501E-5 -4.16027E-6 1.18676E-6

58 -1.58887E-6 3.43182E-6 -1.67249E-6 1.58699E-6 -1.18159E-5 -1.08281E-5 -3.64009E-6 6.573E-7

59 -9.39363E-8 3.77061E-6 -9.88803E-8 1.67956E-6 -1.13459E-5 -1.10447E-5 -2.67358E-6 -1.36923E-7

60 -2.11649E-6 2.68649E-6 -2.22788E-6 1.49443E-6 -1.14131E-5 -1.03949E-5 -2.37656E-6 6.1633E-8

61 -7.09494E-7 3.09304E-6 -7.46836E-7 2.42008E-6 -1.16145E-5 -9.88949E-6 -1.78253E-6 -1.19591E-6

62 -2.81998E-6 2.48321E-6 -2.9684E-6 1.95725E-6 -1.14131E-5 -9.88949E-6 -1.78253E-6 -1.92392E-6

63 4.47878E-6 2.61873E-6 4.71451E-6 2.79034E-6 -1.13459E-5 -1.03949E-5 -1.0391E-6 -2.58573E-6

64 4.83053E-6 2.61873E-6 5.08477E-6 1.77212E-6 -1.08087E-5 -1.10447E-5 -7.4191E-7 -3.04905E-6

65 1.40099E-6 2.48321E-6 1.47473E-6 2.04982E-6 -1.02044E-5 -1.06837E-5 -6.6755E-7 -3.5785E-6

66 -1.41299E-6 2.88976E-6 -1.48736E-6 2.14238E-6 -9.60011E-6 -9.74506E-6 -5.9319E-7 -3.77706E-6

67 -2.02854E-6 2.61873E-6 -2.13531E-6 2.42008E-6 -9.33153E-6 -1.00339E-5 -7.4191E-7 -4.10768E-6

68 -1.85267E-6 2.07667E-6 -1.95018E-6 2.60521E-6 -9.60011E-6 -1.01783E-5 -1.0391E-6 -3.64471E-6

69 -1.23712E-6 2.82201E-6 -1.30223E-6 2.14238E-6 -9.19723E-6 -9.02304E-6 -1.55936E-6 -2.71814E-6

70 -1.14918E-6 2.14442E-6 -1.20966E-6 6.61339E-7 -9.13001E-6 -8.87868E-6 -1.0391E-6 -2.58573E-6

71 -1.50092E-6 3.29631E-6 -1.57992E-6 2.14238E-6 -8.99575E-6 -9.60063E-6 -1.18765E-6 -2.18862E-6

72 -9.73304E-7 4.5837E-6 -1.02453E-6 1.86469E-6 -9.66726E-6 -1.04671E-5 -2.1534E-6 -1.92392E-6

73 -1.32505E-6 5.26128E-6 -1.39479E-6 2.60521E-6 -9.26439E-6 -1.08281E-5 -1.63322E-6 -1.19591E-6

74 -1.06125E-6 6.95522E-6 -1.1171E-6 3.06803E-6 -8.79433E-6 -1.15501E-5 -2.07955E-6 -8.64936E-7

75 8.73369E-7 7.02299E-6 9.19336E-7 2.23495E-6 -9.39867E-6 -1.13335E-5 -1.70783E-6 -1.65916E-6

76 2.01655E-6 5.26128E-6 2.12268E-6 1.95725E-6 -8.25717E-6 -1.12613E-5 -1.26201E-6 -1.52681E-6

77 6.09559E-7 4.5837E-6 6.41641E-7 1.58699E-6 -7.85427E-6 -1.16945E-5 -1.70783E-6 -1.32826E-6

78 -9.39363E-8 2.07667E-6 -9.88803E-8 1.86469E-6 -8.12291E-6 -1.15501E-5 -2.67358E-6 -1.79158E-6

79 -2.11649E-6 7.89273E-7 -2.22788E-6 1.86469E-6 -9.33153E-6 -1.18389E-5 -2.52503E-6 -1.32826E-6

80 -1.58887E-6 1.19582E-6 -1.67249E-6 1.95725E-6 -1.0003E-5 -1.23443E-5 -2.74819E-6 -7.32589E-7

81 -2.29236E-6 -1.59336E-7 -2.41301E-6 1.40186E-6 -1.00701E-5 -1.23443E-5 -2.45118E-6 -1.32826E-6

82 -9.73304E-7 -2.66637E-6 -1.02453E-6 2.14238E-6 -9.33153E-6 -1.29219E-5 -2.22801E-6 -1.26205E-6

83 -1.6768E-6 -2.59861E-6 -1.76505E-6 2.51264E-6 -9.46582E-6 -1.32829E-5 -1.33628E-6 -1.32826E-6

84 -1.32505E-6 -2.9374E-6 -1.39479E-6 1.95725E-6 -9.53287E-6 -1.26331E-5 -9.64821E-7 -1.4606E-6

85 -1.94061E-6 -2.39534E-6 -2.04275E-6 3.80855E-6 -8.92859E-6 -1.32829E-5 -2.96004E-7 -2.12248E-6

86 -2.73205E-6 -2.38199E-8 -2.87584E-6 3.53086E-6 -8.86149E-6 -1.37883E-5 3.7273E-7 -1.4606E-6

87 -1.76474E-6 1.33133E-6 -1.85762E-6 3.34573E-6 -8.45859E-6 -1.41493E-5 6.70001E-7 -1.79158E-6

88 -3.52347E-6 1.80564E-6 -3.70892E-6 2.79034E-6 -8.39143E-6 -1.43659E-5 3.7273E-7 -1.72537E-6

89 -3.61142E-6 1.60236E-6 -3.80149E-6 2.51264E-6 -8.99575E-6 -1.40049E-5 -7.30925E-8 -1.85771E-6

90 -2.46823E-6 7.89273E-7 -2.59814E-6 2.42008E-6 -9.46582E-6 -1.30663E-5 -1.47368E-7 -2.98284E-6

91 -2.20442E-6 2.88976E-6 -2.32044E-6 2.04982E-6 -1.04059E-5 -1.19111E-5 -1.0391E-6 -3.37995E-6

92 -1.6768E-6 3.02528E-6 -1.76505E-6 2.04982E-6 -1.0473E-5 -1.14778E-5 -1.63322E-6 -2.98284E-6

93 -1.41299E-6 4.31268E-6 -1.48736E-6 1.0316E-6 -9.93584E-6 -1.09725E-5 -1.48467E-6 -3.5785E-6

94 -2.38029E-6 3.09304E-6 -2.50557E-6 1.77212E-6 -8.72717E-6 -1.19111E-5 -2.07955E-6 -3.71085E-6

95 -2.11649E-6 4.1094E-6 -2.22788E-6 1.40186E-6 -9.06291E-6 -1.19833E-5 -2.00485E-6 -2.51959E-6

96 -1.50092E-6 3.43182E-6 -1.57992E-6 1.58699E-6 -8.25717E-6 -1.15501E-5 -1.48467E-6 -2.18862E-6

97 -1.32505E-6 3.36406E-6 -1.39479E-6 1.67956E-6 -7.78711E-6 -1.20556E-5 -7.4191E-7 -1.1297E-6

98 -1.14918E-6 2.95752E-6 -1.20966E-6 1.95725E-6 -8.99575E-6 -1.31385E-5 -4.44639E-7 -7.32589E-7

99 -1.14918E-6 2.07667E-6 -1.20966E-6 1.0316E-6 -8.86149E-6 -1.26331E-5 -5.18999E-7 -8.64936E-7

100 -5.33621E-7 3.77061E-6 -5.61706E-7 8.46469E-7 -9.7344E-6 -1.24165E-5 -1.0391E-6 -4.576E-9

101 -1.32505E-6 1.3991E-6 -1.39479E-6 2.42008E-6 -1.08087E-5 -1.26331E-5 -1.93099E-6 -7.0785E-8

102 -3.25967E-6 2.75425E-6 -3.43123E-6 2.23495E-6 -1.00701E-5 -1.26331E-5 -2.74819E-6 -9.31145E-7

103 6.09559E-7 2.95752E-6 6.41641E-7 2.79034E-6 -9.60011E-6 -1.30663E-5 -2.00485E-6 -5.34034E-7

104 -5.33621E-7 2.55097E-6 -5.61706E-7 3.43829E-6 -9.7344E-6 -1.21999E-5 -2.22801E-6 -2.69341E-7

105 -1.50092E-6 1.46685E-6 -1.57992E-6 2.51264E-6 -8.86149E-6 -1.24165E-5 -1.11337E-6 -6.6638E-7

106 -1.50092E-6 1.26358E-6 -1.57992E-6 2.23495E-6 -9.19723E-6 -1.27053E-5 -1.11337E-6 -2.03132E-7

107 -1.41299E-6 1.12806E-6 -1.48736E-6 1.95725E-6 -8.59285E-6 -1.24887E-5 -1.48467E-6 -1.79158E-6

108 -1.50092E-6 2.07667E-6 -1.57992E-6 2.32751E-6 -8.39143E-6 -1.24887E-5 -1.41056E-6 -1.99013E-6

109 -1.32505E-6 9.92549E-7 -1.39479E-6 2.60521E-6 -7.98853E-6 -1.29219E-5 -2.30271E-6 -2.25482E-6

110 -1.85267E-6 2.75425E-6 -1.95018E-6 2.14238E-6 -7.45137E-6 -1.20556E-5 -2.74819E-6 -3.04905E-6

111 -1.41299E-6 1.46685E-6 -1.48736E-6 2.60521E-6 -7.45137E-6 -1.27053E-5 -2.45118E-6 -2.78428E-6

112 -1.58887E-6 1.53461E-6 -1.67249E-6 1.77212E-6 -7.92143E-6 -1.26331E-5 -1.93099E-6 -2.78428E-6

113 -1.50092E-6 1.80564E-6 -1.57992E-6 3.43829E-6 -8.05569E-6 -1.13335E-5 -1.78253E-6 -3.5785E-6

114 -1.06125E-6 9.92549E-7 -1.1171E-6 4.76209E-7 -8.99575E-6 -1.18389E-5 -1.11337E-6 -3.77706E-6

115 -1.23712E-6 3.43182E-6 -1.30223E-6 3.83643E-7 -9.7344E-6 -1.10447E-5 -1.55936E-6 -3.44616E-6

116 -1.06125E-6 2.95752E-6 -1.1171E-6 2.04982E-6 -1.0473E-5 -1.07559E-5 -9.64821E-7 -2.85049E-6

117 -5.33621E-7 3.16079E-6 -5.61706E-7 2.32751E-6 -1.0943E-5 -1.09725E-5 -7.4191E-7 -1.72537E-6

118 -9.73304E-7 2.95752E-6 -1.02453E-6 1.95725E-6 -1.17488E-5 -1.20556E-5 -1.18765E-6 -1.32826E-6

119 -2.73205E-6 2.48321E-6 -2.87584E-6 1.95725E-6 -1.12788E-5 -1.16223E-5 -1.18765E-6 -1.79158E-6

120 -2.11649E-6 1.80564E-6 -2.22788E-6 2.32751E-6 -1.10102E-5 -1.14778E-5 -1.41056E-6 -1.32826E-6

121 -1.76474E-6 2.41545E-6 -1.85762E-6 2.69777E-6 -1.04059E-5 -1.13335E-5 -1.63322E-6 -8.64936E-7

122 -1.85267E-6 1.94115E-6 -1.95018E-6 3.06803E-6 -9.80155E-6 -1.09002E-5 -2.1534E-6 -9.31145E-7

123 -6.21557E-7 5.85999E-7 -6.54271E-7 2.42008E-6 -9.06291E-6 -1.11169E-5 -1.93099E-6 -6.6638E-7

124 -1.41299E-6 1.19582E-6 -1.48736E-6 1.95725E-6 -8.99575E-6 -1.09002E-5 -2.07955E-6 -6.00243E-7

125 -7.97432E-7 -1.59336E-7 -8.39402E-7 2.60521E-6 -8.45859E-6 -1.09002E-5 -2.52503E-6 -7.0785E-8

126 -1.58887E-6 1.19582E-6 -1.67249E-6 2.23495E-6 -6.98137E-6 -1.19111E-5 -1.85638E-6 1.27771E-7

127 -1.14918E-6 1.73788E-6 -1.20966E-6 1.86469E-6 -6.37706E-6 -1.19833E-5 -2.45118E-6 4.58744E-7

128 -1.06125E-6 5.85999E-7 -1.1171E-6 2.32751E-6 -6.24274E-6 -1.19111E-5 -2.52503E-6 3.26326E-7

129 -1.85267E-6 2.88976E-6 -1.95018E-6 1.95725E-6 -6.51131E-6 -1.24887E-5 -1.11337E-6 4.58744E-7

130 8.73369E-7 3.22855E-6 9.19336E-7 8.46469E-7 -7.72001E-6 -1.25609E-5 -1.41056E-6 7.23437E-7

131 -2.6981E-7 2.68649E-6 -2.84011E-7 1.33827E-8 -8.25717E-6 -1.22721E-5 -1.33628E-6 6.573E-7

132 7.85432E-7 1.8734E-6 8.26771E-7 -5.42008E-7 -8.66007E-6 -1.16945E-5 -1.93099E-6 5.91091E-7

133 -9.39363E-8 2.48321E-6 -9.88803E-8 1.3093E-6 -8.99575E-6 -1.12613E-5 -1.33628E-6 -2.69341E-7

134 -1.41299E-6 2.14442E-6 -1.48736E-6 2.91078E-7 -8.72717E-6 -1.16223E-5 -1.33628E-6 -2.05627E-6

135 -2.38029E-6 3.16079E-6 -2.50557E-6 3.83643E-7 -8.25717E-6 -1.19833E-5 -1.33628E-6 -2.25482E-6

136 -1.6768E-6 3.6351E-6 -1.76505E-6 2.91078E-7 -8.86149E-6 -1.19833E-5 -1.33628E-6 -3.31381E-6

137 -4.22697E-6 4.78698E-6 -4.44944E-6 1.05948E-7 -9.46582E-6 -1.20556E-5 -8.90461E-7 -4.63749E-6

138 -4.57872E-6 6.14215E-6 -4.8197E-6 -5.42008E-7 -1.03387E-5 -1.24887E-5 -1.11337E-6 -3.9754E-6

139 -5.89777E-6 4.85473E-6 -6.20818E-6 -1.00483E-6 -1.14131E-5 -1.21277E-5 -2.07955E-6 -4.70399E-6

140 -5.98571E-6 3.16079E-6 -6.30075E-6 -2.64313E-7 -1.12117E-5 -1.21277E-5 -1.48467E-6 -4.30645E-6

141 -5.37015E-6 3.56733E-6 -5.65279E-6 -6.34575E-7 -1.12788E-5 -1.20556E-5 -1.26201E-6 -3.5785E-6

142 -4.93046E-6 3.36406E-6 -5.18996E-6 -1.74536E-6 -1.10773E-5 -1.11169E-5 -1.70783E-6 -2.51959E-6

143 -6.24952E-6 3.43182E-6 -6.57844E-6 -1.65279E-6 -1.03387E-5 -1.09002E-5 -1.55936E-6 -1.85771E-6

144 -3.25967E-6 4.78698E-6 -3.43123E-6 -1.18996E-6 -1.08087E-5 -1.10447E-5 -1.48467E-6 -8.64936E-7

145 -6.07364E-6 3.02528E-6 -6.39331E-6 -2.76357E-6 -1.08759E-5 -1.06837E-5 -1.78253E-6 -9.31145E-7

146 -6.77714E-6 2.27994E-6 -7.13383E-6 -4.15205E-6 -1.06073E-5 -1.01061E-5 -1.55936E-6 -1.4606E-6

147 -5.80984E-6 9.92549E-7 -6.11562E-6 -4.24461E-6 -1.13459E-5 -9.60063E-6 -9.64821E-7 -1.4606E-6

148 -5.89777E-6 1.80564E-6 -6.20818E-6 -5.26283E-6 -1.08087E-5 -9.67285E-6 -1.18765E-6 -2.45338E-6

149 -5.54602E-6 1.26358E-6 -5.83792E-6 -6.83644E-6 -9.7344E-6 -9.96171E-6 -4.44639E-7 -2.65194E-6

150 -6.68921E-6 1.73788E-6 -7.04127E-6 -8.78031E-6 -9.13001E-6 -1.04671E-5 -4.44639E-7 -2.65194E-6

151 -6.86508E-6 3.70285E-6 -7.2264E-6 -7.4844E-6 -9.26439E-6 -1.11891E-5 -6.6755E-7 -2.65194E-6

152 -6.77714E-6 2.07667E-6 -7.13383E-6 -1.02614E-5 -8.79433E-6 -1.12613E-5 3.7273E-7 -2.38717E-6

153 -7.3927E-6 2.00891E-6 -7.78179E-6 -1.22978E-5 -8.99575E-6 -1.27053E-5 2.98454E-7 -1.59302E-6

154 -7.74445E-6 3.16079E-6 -8.15205E-6 -1.23903E-5 -8.32433E-6 -1.27053E-5 8.18552E-7 -9.97354E-7

155 -1.01187E-5 2.3477E-6 -1.06513E-5 -1.2668E-5 -8.45859E-6 -1.26692E-5 9.67187E-7 -1.26205E-6

156 -1.1262E-5 2.21218E-6 -1.18547E-5 -1.36863E-5 -8.59285E-6 -1.29219E-5 -7.4191E-7 -1.19591E-6

157 -1.28448E-5 7.89273E-7 -1.35208E-5 -1.55376E-5 -8.25717E-6 -1.26331E-5 -9.64821E-7 -2.51959E-6

158 -1.61864E-5 1.26358E-6 -1.70383E-5 -1.95179E-5 -7.45137E-6 -1.29219E-5 -1.26201E-6 -3.2476E-6

159 -1.68899E-5 -1.78552E-6 -1.77788E-5 -2.22948E-5 -7.45137E-6 -1.24165E-5 -1.48467E-6 -2.9167E-6

160 -1.57467E-5 -2.66637E-6 -1.65755E-5 -2.51643E-5 -7.72001E-6 -1.23443E-5 -1.55936E-6 -3.84327E-6

161 -1.88245E-5 -4.56359E-6 -1.98153E-5 -2.77562E-5 -6.51131E-6 -1.28497E-5 -1.33628E-6 -4.0419E-6

162 -1.99677E-5 -7.1384E-6 -2.10186E-5 -2.68305E-5 -6.98137E-6 -1.31385E-5 -1.55936E-6 -3.37995E-6

163 -2.27817E-5 -1.03907E-5 -2.39807E-5 -3.02554E-5 -7.58563E-6 -1.28497E-5 -1.78253E-6 -3.71085E-6

164 -2.70906E-5 -1.18814E-5 -2.85164E-5 -3.56242E-5 -7.92143E-6 -1.27775E-5 -2.07955E-6 -3.90941E-6

165 -2.82337E-5 -1.17459E-5 -2.97197E-5 -3.98822E-5 -9.06291E-6 -1.21277E-5 -2.59973E-6 -3.2476E-6

166 -2.99046E-5 -1.43207E-5 -3.14785E-5 -4.19186E-5 -9.39867E-6 -1.11891E-5 -1.33628E-6 -3.04905E-6

167 -3.36859E-5 -1.80474E-5 -3.54588E-5 -4.54361E-5 -9.33153E-6 -1.11891E-5 -2.07955E-6 -2.9167E-6

168 -3.64119E-5 -2.08254E-5 -3.83283E-5 -5.07123E-5 -9.80155E-6 -1.08281E-5 -2.22801E-6 -1.99013E-6

169 -4.06328E-5 -2.46198E-5 -4.27714E-5 -5.56183E-5 -8.86149E-6 -1.06115E-5 -1.55936E-6 -2.12248E-6

170 -4.51176E-5 -2.50942E-5 -4.74922E-5 -5.90432E-5 -8.92859E-6 -9.96171E-6 -1.78253E-6 -1.72537E-6

171 -4.73161E-5 -2.83466E-5 -4.98064E-5 -6.49674E-5 -9.26439E-6 -1.05393E-5 -1.78253E-6 -9.31145E-7

172 -5.35596E-5 -3.2412E-5 -5.63785E-5 -7.05213E-5 -9.33153E-6 -1.02504E-5 -1.48467E-6 -1.52681E-6

173 -6.05945E-5 -3.74261E-5 -6.37837E-5 -7.49644E-5 -8.86149E-6 -1.06115E-5 -1.33628E-6 -1.06349E-6

174 -6.61345E-5 -4.33887E-5 -6.96153E-5 -8.11663E-5 -8.72717E-6 -1.09725E-5 -1.0391E-6 -7.32589E-7

175 -7.36971E-5 -4.71154E-5 -7.75759E-5 -8.59797E-5 -8.25717E-6 -1.14057E-5 2.24179E-7 -1.06349E-6

176 -8.11718E-5 -5.40267E-5 -8.5444E-5 -8.88492E-5 -8.12291E-6 -1.15501E-5 -2.96004E-7 -1.19591E-6

177 -8.88222E-5 -5.84988E-5 -9.34971E-5 -9.72726E-5 -9.06291E-6 -1.14057E-5 -1.11337E-6 -1.19591E-6

178 -9.81435E-5 -6.52743E-5 -1.03309E-4 -1.01531E-4 -9.33153E-6 -1.09002E-5 -9.64821E-7 -1.72537E-6

179 -1.05003E-4 -7.32024E-5 -1.10529E-4 -1.07547E-4 -1.00701E-5 -1.16945E-5 -2.07955E-6 -1.79158E-6

180 -1.13972E-4 -7.80129E-5 -1.19971E-4 -1.1486E-4 -1.11445E-5 -1.18389E-5 -2.59973E-6 -2.58573E-6

181 -1.22766E-4 -8.76344E-5 -1.29227E-4 -1.20692E-4 -1.12117E-5 -1.15501E-5 -1.85638E-6 -2.65194E-6

182 -1.31296E-4 -9.62397E-5 -1.38206E-4 -1.30133E-4 -1.0943E-5 -1.26331E-5 -2.52503E-6 -2.65194E-6

183 -1.41057E-4 -1.041E-4 -1.48481E-4 -1.37724E-4 -9.13001E-6 -1.21999E-5 -2.59973E-6 -3.2476E-6

184 -1.52841E-4 -1.1406E-4 -1.60885E-4 -1.4624E-4 -7.45137E-6 -1.23443E-5 -2.52503E-6 -3.84327E-6

185 -1.62954E-4 -1.23139E-4 -1.7153E-4 -1.5781E-4 -6.37706E-6 -1.29941E-5 -2.22801E-6 -3.9754E-6

186 -1.72538E-4 -1.34184E-4 -1.81619E-4 -1.6503E-4 -6.24274E-6 -1.16945E-5 -1.78253E-6 -3.37995E-6

187 -1.82915E-4 -1.45025E-4 -1.92542E-4 -1.77527E-4 -5.10125E-6 -1.13335E-5 -1.18765E-6 -3.31381E-6

188 -1.88718E-4 -1.58848E-4 -1.98651E-4 -1.88634E-4 -6.17557E-6 -1.17667E-5 -1.41056E-6 -3.44616E-6

189 -1.98655E-4 -1.72196E-4 -2.09111E-4 -2.00483E-4 -6.37706E-6 -1.10447E-5 -9.64821E-7 -3.64471E-6

190 -2.09208E-4 -1.85409E-4 -2.20219E-4 -2.12979E-4 -6.57848E-6 -1.21999E-5 -8.16101E-7 -3.64471E-6

191 -2.20992E-4 -1.99774E-4 -2.32623E-4 -2.24827E-4 -7.65285E-6 -1.19833E-5 -6.6755E-7 -3.37995E-6

192 -2.31456E-4 -2.13528E-4 -2.43638E-4 -2.39082E-4 -8.32433E-6 -1.22721E-5 1.183E-9 -2.58573E-6

193 -2.44734E-4 -2.26538E-4 -2.57615E-4 -2.53245E-4 -7.78711E-6 -1.20556E-5 3.7273E-7 -2.38717E-6

194 -2.55287E-4 -2.39818E-4 -2.68723E-4 -2.69259E-4 -8.05569E-6 -1.19833E-5 8.18552E-7 -1.32826E-6

195 -2.66367E-4 -2.5337E-4 -2.80386E-4 -2.84625E-4 -7.78711E-6 -1.19833E-5 1.41301E-6 -1.4606E-6

196 -2.77886E-4 -2.66921E-4 -2.92512E-4 -2.99805E-4 -7.04854E-6 -1.19111E-5 1.56173E-6 -1.59302E-6

197 -2.87648E-4 -2.79118E-4 -3.02787E-4 -3.13783E-4 -7.18279E-6 -1.20556E-5 1.41301E-6 -1.65916E-6

198 -2.98024E-4 -2.92873E-4 -3.1371E-4 -3.29148E-4 -8.25717E-6 -1.09725E-5 1.1901E-6 -2.05627E-6

199 -3.09368E-4 -3.07847E-4 -3.25651E-4 -3.43866E-4 -7.45137E-6 -1.16945E-5 9.67187E-7 -2.12248E-6

200 -3.17019E-4 -3.22686E-4 -3.33704E-4 -3.59139E-4 -7.24995E-6 -1.09002E-5 -1.47368E-7 -1.79158E-6

201 -3.33099E-4 -3.47029E-4 -3.50631E-4 -3.80517E-4 -6.47395E-6 -1.069E-5 -1.05219E-6 -1.88117E-6

202 -3.46302E-4 -3.72488E-4 -3.64528E-4 -4.00331E-4 -6.91422E-6 -1.03227E-5 -2.37656E-6 -1.52681E-6

203 -3.58524E-4 -3.90512E-4 -3.77394E-4 -4.15049E-4 -8.05569E-6 -9.67285E-6 -2.1534E-6 -1.52681E-6

204 -3.70836E-4 -4.07383E-4 -3.90354E-4 -4.28841E-4 -8.72717E-6 -9.88949E-6 -2.97136E-6 -1.32826E-6

205 -3.81652E-4 -4.2439E-4 -4.01739E-4 -4.42911E-4 -9.7344E-6 -9.96171E-6 -3.19376E-6 -1.99013E-6

206 -3.94754E-4 -4.40652E-4 -4.15531E-4 -4.56703E-4 -9.60011E-6 -9.81721E-6 -2.82205E-6 -1.4606E-6

207 -4.0434E-4 -4.57795E-4 -4.25621E-4 -4.69385E-4 -8.79433E-6 -1.03227E-5 -3.04521E-6 -8.64936E-7

208 -4.16827E-4 -4.7033E-4 -4.38765E-4 -4.83177E-4 -9.80155E-6 -9.88949E-6 -2.67358E-6 -1.52681E-6

209 -4.25533E-4 -4.86388E-4 -4.47929E-4 -4.93637E-4 -9.26439E-6 -1.01061E-5 -1.85638E-6 -1.79158E-6

210 -4.38108E-4 -5.0265E-4 -4.61166E-4 -5.04004E-4 -9.13001E-6 -1.02504E-5 -1.41056E-6 -1.1297E-6

211 -4.49803E-4 -5.19589E-4 -4.73477E-4 -5.165E-4 -9.80155E-6 -1.11891E-5 -1.41056E-6 -1.1297E-6

212 -4.62994E-4 -5.35107E-4 -4.87362E-4 -5.28163E-4 -1.01372E-5 -1.16945E-5 -6.6755E-7 -1.65916E-6

213 -4.74953E-4 -5.50149E-4 -4.99951E-4 -5.37698E-4 -1.05402E-5 -1.20556E-5 -1.41056E-6 -1.99013E-6

214 -4.87176E-4 -5.62277E-4 -5.12817E-4 -5.48806E-4 -1.04059E-5 -1.24165E-5 -1.26201E-6 -2.58573E-6

215 -4.96586E-4 -5.73254E-4 -5.22722E-4 -5.60469E-4 -9.19723E-6 -1.19833E-5 -1.18765E-6 -3.2476E-6

216 -5.07226E-4 -5.83621E-4 -5.33922E-4 -5.71854E-4 -8.86149E-6 -1.15501E-5 -7.4191E-7 -2.71814E-6

217 -5.20505E-4 -5.94259E-4 -5.479E-4 -5.80926E-4 -8.05569E-6 -1.02504E-5 -2.96004E-7 -2.9167E-6

218 -5.33168E-4 -6.03541E-4 -5.61229E-4 -5.92681E-4 -7.18279E-6 -8.87868E-6 -7.30925E-8 -3.44616E-6

219 -5.45654E-4 -6.161E-4 -5.74373E-4 -6.04345E-4 -7.31711E-6 -7.94001E-6 -1.0391E-6 -2.51959E-6

220 -5.56207E-4 -6.283E-4 -5.85481E-4 -6.15638E-4 -7.92143E-6 -7.57907E-6 -1.33628E-6 -3.18139E-6

221 -5.6676E-4 -6.344E-4 -5.96589E-4 -6.26005E-4 -7.45137E-6 -9.67285E-6 -8.16101E-7 -2.58573E-6

222 -5.78631E-4 -6.466E-4 -6.09085E-4 -6.36835E-4 -8.05569E-6 -1.24165E-5 -1.0391E-6 -2.25482E-6

223 -5.89535E-4 -6.588E-4 -6.20563E-4 -6.46925E-4 -8.39143E-6 -1.38605E-5 -1.11337E-6 -2.05627E-6

224 -5.97274E-4 -6.71E-4 -6.28709E-4 -6.56922E-4 -7.85427E-6 -1.52323E-5 -1.55936E-6 -2.12248E-6

225 -6.0765E-4 -6.832E-4 -6.39632E-4 -6.66548E-4 -8.12291E-6 -1.60987E-5 -2.59973E-6 -1.65916E-6

226 -6.17235E-4 -6.954E-4 -6.49721E-4 -6.7923E-4 -7.85427E-6 -1.64597E-5 -2.52503E-6 -1.4606E-6

227 -6.26556E-4 -7.076E-4 -6.59533E-4 -6.91356E-4 -7.18279E-6 -1.81203E-5 -2.30271E-6 -1.59302E-6

228 -6.37373E-4 -7.198E-4 -6.70919E-4 -7.05241E-4 -7.92143E-6 -1.6171E-5 -2.00485E-6 -6.00243E-7

229 -6.44935E-4 -7.32E-4 -6.78879E-4 -7.1533E-4 -8.05569E-6 -1.49436E-5 -1.85638E-6 -1.19591E-6

230 -6.56279E-4 -7.442E-4 -6.9082E-4 -7.26993E-4 -8.19001E-6 -1.37883E-5 -1.26201E-6 -7.98798E-7

231 -6.65249E-4 -7.625E-4 -7.00262E-4 -7.36805E-4 -9.7344E-6 -1.21277E-5 -1.48467E-6 -9.97354E-7

232 -6.76856E-4 -7.686E-4 -7.1248E-4 -7.41526E-4 -9.46582E-6 -1.11891E-5 -9.64821E-7 -5.34034E-7

233 -6.84331E-4 -7.747E-4 -7.20348E-4 -7.50875E-4 -9.39867E-6 -9.96171E-6 -9.64821E-7 -7.98798E-7

234 -6.91629E-4 -7.808E-4 -7.28031E-4 -7.54485E-4 -9.80155E-6 -9.81721E-6 -1.18765E-6 -8.64936E-7

235 -6.99895E-4 -7.93E-4 -7.36732E-4 -7.6152E-4 -1.02044E-5 -9.45627E-6 -1.63322E-6 -1.39446E-6

236 -7.07107E-4 -7.991E-4 -7.44323E-4 -7.66519E-4 -1.0943E-5 -9.60063E-6 -1.48467E-6 -1.32826E-6

237 -7.14406E-4 -7.991E-4 -7.52006E-4 -7.68555E-4 -1.22188E-5 -1.11891E-5 -8.90461E-7 -1.26205E-6

238 -7.20737E-4 -8.052E-4 -7.58671E-4 -7.71332E-4 -1.19502E-5 -1.20556E-5 -7.30925E-8 -1.52681E-6

239 -7.28388E-4 -8.052E-4 -7.66724E-4 -7.75035E-4 -1.12117E-5 -1.24165E-5 -3.70279E-7 -5.34034E-7

240 -7.33048E-4 -8.052E-4 -7.7163E-4 -7.77349E-4 -1.08759E-5 -1.29941E-5 -3.70279E-7 -9.31145E-7

241 -7.40523E-4 -8.052E-4 -7.79498E-4 -7.80589E-4 -1.02716E-5 -1.28497E-5 8.18552E-7 -1.39446E-6

242 -7.46238E-4 -7.991E-4 -7.85514E-4 -7.82347E-4 -8.99575E-6 -1.30663E-5 1.41301E-6 -9.31145E-7

243 -7.48173E-4 -7.991E-4 -7.87551E-4 -7.8318E-4 -9.66726E-6 -1.23443E-5 1.26446E-6 -1.32826E-6

244 -7.50812E-4 -7.991E-4 -7.90328E-4 -7.82347E-4 -9.39867E-6 -1.23443E-5 1.48737E-6 -1.26205E-6

245 -7.54417E-4 -7.991E-4 -7.94123E-4 -7.78645E-4 -8.39143E-6 -1.19111E-5 1.93319E-6 -8.64936E-7

246 -7.56527E-4 -7.991E-4 -7.96344E-4 -7.77256E-4 -8.19001E-6 -1.27053E-5 1.11582E-6 -1.19591E-6

247 -7.61188E-4 -7.93E-4 -8.0125E-4 -7.73831E-4 -7.78711E-6 -1.31385E-5 6.70001E-7 -9.97354E-7

248 -7.60924E-4 -7.93E-4 -8.00973E-4 -7.68463E-4 -7.58563E-6 -1.42938E-5 5.21365E-7 -5.34034E-7

249 -7.62419E-4 -7.808E-4 -8.02546E-4 -7.67444E-4 -7.92143E-6 -1.42215E-5 -2.96004E-7 -7.98798E-7

250 -7.61276E-4 -7.808E-4 -8.01343E-4 -7.63001E-4 -8.12291E-6 -1.48714E-5 1.183E-9 -3.35478E-7

251 -7.61012E-4 -7.808E-4 -8.01065E-4 -7.58188E-4 -8.25717E-6 -1.43659E-5 1.183E-9 -9.31145E-7

252 -7.60133E-4 -7.808E-4 -8.0014E-4 -7.53282E-4 -8.66007E-6 -1.40049E-5 -2.21728E-7 -1.32826E-6

253 -7.57318E-4 -7.747E-4 -7.97177E-4 -7.52079E-4 -8.99575E-6 -1.36439E-5 -2.21728E-7 -1.85771E-6

254 -7.54329E-4 -7.747E-4 -7.9403E-4 -7.47821E-4 -9.26439E-6 -1.33551E-5 -8.16101E-7 -1.79158E-6

255 -7.5002E-4 -7.747E-4 -7.89495E-4 -7.38934E-4 -9.80155E-6 -1.27053E-5 -8.90461E-7 -1.52681E-6

256 -7.47206E-4 -7.686E-4 -7.86533E-4 -7.34676E-4 -9.60011E-6 -1.28497E-5 -4.44639E-7 -1.4606E-6

257 -7.43073E-4 -7.686E-4 -7.82182E-4 -7.2542E-4 -1.0003E-5 -1.38244E-5 3.8363E-8 -1.06349E-6

258 -7.39116E-4 -7.686E-4 -7.78017E-4 -7.20606E-4 -9.80155E-6 -1.34995E-5 -7.30925E-8 -1.65916E-6

259 -7.34983E-4 -7.625E-4 -7.73666E-4 -7.11998E-4 -1.06073E-5 -1.40049E-5 9.67187E-7 -1.79158E-6

260 -7.33487E-4 -7.625E-4 -7.72092E-4 -7.07277E-4 -1.06073E-5 -1.41493E-5 -2.21728E-7 -1.92392E-6

261 -7.28035E-4 -7.625E-4 -7.66353E-4 -7.02279E-4 -1.06744E-5 -1.37883E-5 -1.11337E-6 -1.99013E-6

262 -7.22936E-4 -7.625E-4 -7.60985E-4 -6.97558E-4 -1.06073E-5 -1.41493E-5 -2.30271E-6 -1.85771E-6

263 -7.20298E-4 -7.564E-4 -7.58208E-4 -6.94318E-4 -1.07416E-5 -1.40049E-5 -3.19376E-6 -2.18862E-6

264 -7.15373E-4 -7.564E-4 -7.53024E-4 -6.92374E-4 -1.11445E-5 -1.28497E-5 -4.01172E-6 -2.18862E-6

265 -7.12559E-4 -7.564E-4 -7.50062E-4 -6.87746E-4 -1.04059E-5 -1.22721E-5 -3.49154E-6 -2.05627E-6

266 -7.07547E-4 -7.442E-4 -7.44786E-4 -6.80433E-4 -1.05402E-5 -1.12613E-5 -4.08558E-6 -2.18862E-6

267 -7.02446E-4 -7.381E-4 -7.39417E-4 -6.77101E-4 -8.12291E-6 -1.04671E-5 -3.04521E-6 -2.12248E-6

268 -6.97521E-4 -7.381E-4 -7.34233E-4 -6.70621E-4 -6.57848E-6 -1.01783E-5 -2.37656E-6 -1.39446E-6

269 -6.91014E-4 -7.32E-4 -7.27383E-4 -6.64049E-4 -6.04131E-6 -1.09725E-5 -1.63322E-6 -1.1297E-6

270 -6.89607E-4 -7.259E-4 -7.25902E-4 -6.58125E-4 -5.57129E-6 -1.09002E-5 -6.6755E-7 -1.4606E-6

271 -6.82396E-4 -7.198E-4 -7.18312E-4 -6.50072E-4 -5.10125E-6 -1.16945E-5 -5.9319E-7 -1.26205E-6

272 -6.73515E-4 -7.198E-4 -7.08963E-4 -6.42944E-4 -6.17557E-6 -1.18389E-5 -8.16101E-7 -1.65916E-6

273 -6.69734E-4 -7.137E-4 -7.04983E-4 -6.34984E-4 -6.64563E-6 -1.17667E-5 -6.6755E-7 -2.18862E-6

274 -6.63051E-4 -7.076E-4 -6.97948E-4 -6.28134E-4 -7.31711E-6 -1.24887E-5 -5.18999E-7 -2.12248E-6

275 -6.58829E-4 -7.015E-4 -6.93504E-4 -6.20358E-4 -9.13001E-6 -1.28497E-5 -5.9319E-7 -2.58573E-6

276 -6.51355E-4 -6.954E-4 -6.85637E-4 -6.14712E-4 -1.06744E-5 -1.21999E-5 -2.96004E-7 -2.98284E-6

277 -6.47309E-4 -6.893E-4 -6.81378E-4 -6.05455E-4 -1.14131E-5 -1.32829E-5 -7.4191E-7 -3.2476E-6

278 -6.41506E-4 -6.832E-4 -6.75269E-4 -6.00179E-4 -1.22188E-5 -1.26331E-5 -5.18999E-7 -3.5785E-6

279 -6.35614E-4 -6.771E-4 -6.69067E-4 -5.91848E-4 -1.26217E-5 -1.24165E-5 7.5543E-8 -3.2476E-6

280 -6.31217E-4 -6.71E-4 -6.64439E-4 -5.84721E-4 -1.15473E-5 -1.29219E-5 1.11582E-6 -2.45338E-6

281 -6.23391E-4 -6.649E-4 -6.56201E-4 -5.76945E-4 -1.20845E-5 -1.30663E-5 1.49819E-7 -2.18862E-6

282 -6.20225E-4 -6.588E-4 -6.52868E-4 -5.72317E-4 -1.17488E-5 -1.26331E-5 8.92912E-7 -1.59302E-6

283 -6.10992E-4 -6.466E-4 -6.43149E-4 -5.66023E-4 -1.11445E-5 -1.28497E-5 -7.30925E-8 -1.59302E-6

284 -6.051E-4 -6.466E-4 -6.36947E-4 -5.61394E-4 -1.12788E-5 -1.29941E-5 -7.30925E-8 -1.52681E-6

285 -5.96922E-4 -6.344E-4 -6.28339E-4 -5.57044E-4 -1.05402E-5 -1.33551E-5 1.49819E-7 -1.52681E-6

286 -5.88216E-4 -6.283E-4 -6.19175E-4 -5.50101E-4 -1.08087E-5 -1.36439E-5 -5.18999E-7 -8.64936E-7

287 -5.81709E-4 -6.161E-4 -6.12325E-4 -5.4464E-4 -1.04059E-5 -1.33551E-5 -1.26201E-6 -1.52681E-6

288 -5.76257E-4 -6.1E-4 -6.06586E-4 -5.37513E-4 -1.01372E-5 -1.30663E-5 -1.70783E-6 -2.45338E-6

289 -5.72299E-4 -6.01238E-4 -6.0242E-4 -5.31403E-4 -9.8687E-6 -1.29941E-5 -2.07955E-6 -2.05627E-6

290 -5.66495E-4 -5.92226E-4 -5.96311E-4 -5.22147E-4 -1.05402E-5 -1.26331E-5 -9.64821E-7 -2.12248E-6

291 -5.59725E-4 -5.83621E-4 -5.89184E-4 -5.14094E-4 -9.8687E-6 -1.26331E-5 2.98454E-7 -2.58573E-6

292 -5.52602E-4 -5.7427E-4 -5.81686E-4 -5.04652E-4 -1.02716E-5 -1.25609E-5 2.98454E-7 -1.92392E-6

293 -5.48117E-4 -5.64243E-4 -5.76965E-4 -4.96969E-4 -1.02716E-5 -1.28497E-5 8.92912E-7 -3.11526E-6

294 -5.42313E-4 -5.53333E-4 -5.70856E-4 -4.86324E-4 -9.7344E-6 -1.27775E-5 1.63592E-6 -2.71814E-6

295 -5.34135E-4 -5.42086E-4 -5.62247E-4 -4.75957E-4 -9.26439E-6 -1.25609E-5 -7.30925E-8 -2.25482E-6

296 -5.23495E-4 -5.30363E-4 -5.51047E-4 -4.68089E-4 -8.72717E-6 -1.19111E-5 -1.18765E-6 -2.18862E-6

297 -5.17603E-4 -5.20132E-4 -5.44845E-4 -4.57444E-4 -8.39143E-6 -1.18389E-5 -1.55936E-6 -2.65194E-6

298 -5.06874E-4 -5.08139E-4 -5.33552E-4 -4.49298E-4 -8.39143E-6 -1.10447E-5 -1.63322E-6 -2.65194E-6

299 -5.02125E-4 -4.98788E-4 -5.28553E-4 -4.40041E-4 -8.59285E-6 -1.02504E-5 1.183E-9 -3.11526E-6

300 -4.97026E-4 -4.87405E-4 -5.23185E-4 -4.29304E-4 -8.99575E-6 -9.81721E-6 5.95641E-7 -3.44616E-6

301 -4.84802E-4 -4.74531E-4 -5.10318E-4 -4.21714E-4 -9.7344E-6 -1.02504E-5 1.1901E-6 -3.11526E-6

302 -4.80054E-4 -4.63351E-4 -5.0532E-4 -4.11994E-4 -1.02044E-5 -1.09002E-5 6.70001E-7 -2.9167E-6

303 -4.71436E-4 -4.51629E-4 -4.96248E-4 -4.02275E-4 -1.03387E-5 -1.21999E-5 2.24179E-7 -3.04905E-6

304 -4.63081E-4 -4.39906E-4 -4.87454E-4 -3.94222E-4 -1.06744E-5 -1.23443E-5 -6.6755E-7 -2.85049E-6

305 -4.53321E-4 -4.27236E-4 -4.7718E-4 -3.83392E-4 -1.08759E-5 -1.21999E-5 -1.85638E-6 -2.98284E-6

306 -4.4312E-4 -4.16057E-4 -4.66442E-4 -3.74783E-4 -1.08759E-5 -1.27775E-5 -2.74819E-6 -1.92392E-6

307 -4.3292E-4 -4.02301E-4 -4.55705E-4 -3.65249E-4 -1.16145E-5 -1.30663E-5 -3.49154E-6 -1.79158E-6

308 -4.24038E-4 -3.90309E-4 -4.46356E-4 -3.54604E-4 -1.12117E-5 -1.35717E-5 -3.49154E-6 -1.59302E-6

309 -4.13486E-4 -3.77367E-4 -4.35248E-4 -3.44607E-4 -1.06073E-5 -1.37883E-5 -3.26838E-6 -1.19591E-6

310 -4.04428E-4 -3.64357E-4 -4.25714E-4 -3.34425E-4 -1.05402E-5 -1.42938E-5 -2.74819E-6 -6.00243E-7

311 -3.94667E-4 -3.50873E-4 -4.15439E-4 -3.23502E-4 -9.93584E-6 -1.32107E-5 -2.52503E-6 -4.67896E-7

312 -3.82883E-4 -3.36644E-4 -4.03035E-4 -3.12579E-4 -9.8687E-6 -1.32829E-5 -2.22801E-6 1.9398E-7

313 -3.71364E-4 -3.22483E-4 -3.90909E-4 -3.02397E-4 -9.8687E-6 -1.27775E-5 -1.93099E-6 5.24882E-7

314 -3.63098E-4 -3.10151E-4 -3.82208E-4 -2.9277E-4 -9.66726E-6 -1.15501E-5 -1.18765E-6 4.58744E-7

315 -3.52281E-4 -2.97074E-4 -3.70822E-4 -2.81385E-4 -8.72717E-6 -1.09725E-5 -6.6755E-7 1.45145E-6

316 -3.44279E-4 -2.86164E-4 -3.62399E-4 -2.68055E-4 -9.93584E-6 -1.06115E-5 -9.64821E-7 2.44423E-6

317 -3.383E-4 -2.72884E-4 -3.56105E-4 -2.58521E-4 -9.19723E-6 -1.14057E-5 -1.33628E-6 2.84134E-6

318 -3.28714E-4 -2.61094E-4 -3.46015E-4 -2.47876E-4 -8.79433E-6 -1.24165E-5 -1.18765E-6 3.3708E-6

319 -3.23789E-4 -2.50118E-4 -3.40831E-4 -2.37416E-4 -8.32433E-6 -1.29219E-5 -1.33628E-6 2.77513E-6

320 -3.13589E-4 -2.39683E-4 -3.30094E-4 -2.27697E-4 -8.52575E-6 -1.31385E-5 -5.18999E-7 1.91477E-6

321 -3.07962E-4 -2.28435E-4 -3.2417E-4 -2.19459E-4 -8.86149E-6 -1.34995E-5 -1.0391E-6 1.58387E-6

322 -2.99695E-4 -2.17255E-4 -3.15468E-4 -2.08629E-4 -9.66726E-6 -1.21999E-5 -1.0391E-6 3.26326E-7

323 -2.88879E-4 -2.07294E-4 -3.04083E-4 -2.01316E-4 -9.19723E-6 -1.18389E-5 -5.9319E-7 -7.32589E-7

324 -2.84218E-4 -1.97267E-4 -2.99177E-4 -1.91782E-4 -9.26439E-6 -1.18389E-5 -6.6755E-7 -1.72537E-6

325 -2.73841E-4 -1.88119E-4 -2.88254E-4 -1.80211E-4 -9.53287E-6 -1.07559E-5 -8.90461E-7 -2.51959E-6

326 -2.65311E-4 -1.79107E-4 -2.79275E-4 -1.70399E-4 -9.60011E-6 -1.11169E-5 -1.41056E-6 -2.65194E-6

327 -2.58892E-4 -1.70366E-4 -2.72518E-4 -1.61143E-4 -9.39867E-6 -1.03949E-5 -1.70783E-6 -3.2476E-6

328 -2.53528E-4 -1.60067E-4 -2.66872E-4 -1.52997E-4 -9.8687E-6 -1.06837E-5 -1.26201E-6 -3.9754E-6

329 -2.43767E-4 -1.52208E-4 -2.56597E-4 -1.45684E-4 -1.08087E-5 -1.12613E-5 -1.26201E-6 -4.43872E-6

330 -2.40162E-4 -1.42993E-4 -2.52802E-4 -1.37261E-4 -1.08759E-5 -1.12613E-5 -1.11337E-6 -3.9754E-6

331 -2.32423E-4 -1.34116E-4 -2.44656E-4 -1.2856E-4 -1.02716E-5 -1.21999E-5 -1.18765E-6 -3.77706E-6

332 -2.26531E-4 -1.27273E-4 -2.38454E-4 -1.17359E-4 -9.33153E-6 -1.19111E-5 -6.6755E-7 -3.2476E-6

333 -2.19497E-4 -1.19006E-4 -2.31049E-4 -1.07918E-4 -9.46582E-6 -1.19833E-5 -6.6755E-7 -3.18139E-6

334 -2.12022E-4 -1.10198E-4 -2.23181E-4 -1.00883E-4 -9.80155E-6 -1.09002E-5 -7.30925E-8 -3.2476E-6

335 -2.03228E-4 -1.05387E-4 -2.13924E-4 -9.15336E-5 -9.13001E-6 -1.14057E-5 -2.96004E-7 -2.9167E-6

336 -1.95577E-4 -9.85431E-5 -2.05871E-4 -8.51466E-5 -8.59285E-6 -1.19111E-5 -7.4191E-7 -3.18139E-6

337 -1.85729E-4 -9.22424E-5 -1.95504E-4 -7.68157E-5 -7.92143E-6 -1.11891E-5 -5.9319E-7 -3.77706E-6

338 -1.80101E-4 -8.71599E-5 -1.8958E-4 -7.19098E-5 -6.57848E-6 -1.14778E-5 -8.90461E-7 -4.30645E-6

339 -1.71131E-4 -8.03846E-5 -1.80138E-4 -6.68187E-5 -5.97416E-6 -1.13335E-5 -1.85638E-6 -4.30645E-6

340 -1.64712E-4 -7.65898E-5 -1.73381E-4 -6.33012E-5 -5.70558E-6 -1.14057E-5 -9.64821E-7 -4.30645E-6

341 -1.57149E-4 -6.85268E-5 -1.6542E-4 -5.86729E-5 -6.84705E-6 -1.11891E-5 -1.33628E-6 -3.84327E-6

342 -1.49411E-4 -6.58166E-5 -1.57275E-4 -5.71919E-5 -7.65285E-6 -1.07559E-5 -1.0391E-6 -3.64471E-6

343 -1.39386E-4 -6.20224E-5 -1.46722E-4 -5.74696E-5 -8.05569E-6 -1.11891E-5 -5.9319E-7 -3.44616E-6

344 -1.30768E-4 -5.72791E-5 -1.37651E-4 -5.68216E-5 -7.98853E-6 -1.19111E-5 -3.70279E-7 -2.78428E-6

345 -1.25492E-4 -5.423E-5 -1.32097E-4 -5.39521E-5 -8.19001E-6 -1.25609E-5 -7.4191E-7 -2.18862E-6

346 -1.16962E-4 -5.11808E-5 -1.23118E-4 -5.28413E-5 -8.12291E-6 -1.24887E-5 -4.44639E-7 -1.52681E-6

347 -1.11334E-4 -5.06388E-5 -1.17194E-4 -5.45075E-5 -7.51853E-6 -1.26331E-5 -1.0391E-6 -1.1297E-6

348 -1.03771E-4 -5.10453E-5 -1.09233E-4 -5.1638E-5 -8.39143E-6 -1.24165E-5 -2.07955E-6 -2.05627E-6

349 -9.83193E-5 -5.13164E-5 -1.03494E-4 -5.02495E-5 -9.06291E-6 -1.16223E-5 -1.33628E-6 -2.98284E-6

350 -9.18121E-5 -5.11808E-5 -9.66443E-5 -4.74725E-5 -8.99575E-6 -1.16945E-5 -1.41056E-6 -3.31381E-6

351 -8.45133E-5 -5.03678E-5 -8.89614E-5 -4.71949E-5 -9.53287E-6 -1.13335E-5 -1.78253E-6 -2.85049E-6

352 -8.30184E-5 -4.87416E-5 -8.73878E-5 -4.33997E-5 -9.39867E-6 -1.16223E-5 -1.33628E-6 -2.9167E-6

353 -7.65111E-5 -4.63701E-5 -8.0538E-5 -4.30294E-5 -9.39867E-6 -1.24165E-5 -1.78253E-6 -2.9167E-6

354 -7.19384E-5 -4.48794E-5 -7.57246E-5 -4.22889E-5 -9.39867E-6 -1.19111E-5 -1.85638E-6 -3.31381E-6

355 -6.82451E-5 -4.1356E-5 -7.18369E-5 -4.21963E-5 -9.66726E-6 -1.21277E-5 -1.11337E-6 -3.77706E-6

356 -6.32327E-5 -3.83747E-5 -6.65607E-5 -3.79383E-5 -1.00701E-5 -1.18389E-5 -1.11337E-6 -3.64471E-6

357 -6.05945E-5 -4.18981E-5 -6.37837E-5 -3.23844E-5 -1.0473E-5 -1.06837E-5 -9.64821E-7 -3.71085E-6

358 -5.36475E-5 -3.95943E-5 -5.64711E-5 -2.68305E-5 -1.04059E-5 -1.03227E-5 -6.6755E-7 -3.64471E-6

359 -5.39114E-5 -3.59354E-5 -5.67488E-5 -2.73859E-5 -9.7344E-6 -1.09725E-5 -1.00192E-6 -3.77706E-6

360 -5.17129E-5 -3.23442E-5 -5.44346E-5 -2.09989E-5 -9.93584E-6 -1.09002E-5 -1.33628E-6 -3.5785E-6

361 -4.72281E-5 -2.55685E-5 -4.97138E-5 -1.46119E-5 -9.46582E-6 -1.02504E-5 -1.48467E-6 -2.98284E-6

362 -4.40624E-5 -2.10965E-5 -4.63815E-5 -1.20201E-5 -1.0003E-5 -9.60063E-6 -2.22801E-6 -1.99013E-6

363 -4.29192E-5 -1.51338E-5 -4.51781E-5 -1.17424E-5 -1.0003E-5 -9.88949E-6 -1.78253E-6 -9.97354E-7

364 -4.18639E-5 -1.04585E-5 -4.40673E-5 -9.79852E-6 -9.26439E-6 -9.52848E-6 -1.48467E-6 -5.34034E-7

365 -3.82586E-5 -5.03789E-6 -4.02722E-5 -6.55874E-6 -8.99575E-6 -1.03227E-5 -1.55936E-6 6.1633E-8

366 -3.37737E-5 -2.73413E-6 -3.55513E-5 -3.41153E-6 -7.85427E-6 -9.81721E-6 -1.0391E-6 6.1633E-8

367 -3.08719E-5 3.14968E-7 -3.24967E-5 -3.04127E-6 -7.58563E-6 -9.88949E-6 -8.90461E-7 -6.6638E-7

368 -2.96408E-5 3.43182E-6 -3.12008E-5 1.33827E-8 -8.19001E-6 -1.09002E-5 -8.90461E-7 -1.1297E-6

369 -2.69147E-5 6.81968E-6 -2.83313E-5 5.68774E-7 -8.86149E-6 -1.10447E-5 4.4709E-7 -1.52681E-6

370 -2.2342E-5 9.52997E-6 -2.35179E-5 1.86469E-6 -8.99575E-6 -1.19111E-5 8.18552E-7 -1.32826E-6

371 -1.82969E-5 1.00721E-5 -1.92599E-5 2.14238E-6 -9.66726E-6 -1.24165E-5 6.70001E-7 -1.59302E-6

372 -1.47794E-5 1.02076E-5 -1.55573E-5 3.25316E-6 -9.26439E-6 -1.28497E-5 7.44276E-7 -2.25482E-6

373 -1.3988E-5 8.71696E-6 -1.47242E-5 -7.91825E-8 -8.99575E-6 -1.32829E-5 1.11582E-6 -2.05627E-6

374 -1.37242E-5 9.39449E-6 -1.44465E-5 -1.46766E-6 -8.32433E-6 -1.28497E-5 5.95641E-7 -1.1297E-6

375 -1.1262E-5 9.59774E-6 -1.18547E-5 -3.13383E-6 -8.25717E-6 -1.23443E-5 -3.70279E-7 -1.79158E-6

376 -1.02946E-5 9.73328E-6 -1.08364E-5 -5.81822E-6 -8.19001E-6 -1.22721E-5 -1.11337E-6 -7.32589E-7

377 -6.60126E-6 1.0682E-5 -6.9487E-6 -6.74387E-6 -9.19723E-6 -1.11891E-5 -1.55936E-6 -1.19591E-6

378 -5.54602E-6 1.10884E-5 -5.83792E-6 -8.31748E-6 -9.33153E-6 -1.06837E-5 -2.07955E-6 -1.52681E-6

379 -5.37015E-6 1.25791E-5 -5.65279E-6 -9.058E-6 -1.00701E-5 -1.09002E-5 -1.48467E-6 -1.1297E-6

380 -4.40284E-6 1.55604E-5 -4.63457E-6 -8.96544E-6 -9.46582E-6 -1.11169E-5 -2.1534E-6 -1.59302E-6

381 -3.17173E-6 1.64413E-5 -3.33866E-6 -7.57696E-6 -8.66007E-6 -1.09725E-5 -2.45118E-6 -1.85771E-6

382 -1.32505E-6 1.88128E-5 -1.39479E-6 -7.39183E-6 -8.25717E-6 -1.20556E-5 -2.82205E-6 -1.52681E-6

383 -1.32505E-6 1.98292E-5 -1.39479E-6 -5.81822E-6 -8.72717E-6 -1.14778E-5 -2.97136E-6 -1.59302E-6

384 -1.94061E-6 2.25395E-5 -2.04275E-6 -4.61488E-6 -9.7344E-6 -1.15501E-5 -3.19376E-6 -1.99013E-6

385 4.33685E-7 2.44367E-5 4.5651E-7 -1.74536E-6 -9.60011E-6 -1.19833E-5 -2.52503E-6 -2.18862E-6

386 4.39084E-6 2.69438E-5 4.62194E-6 -8.19704E-7 -1.08759E-5 -1.16223E-5 -2.1534E-6 -2.25482E-6

387 -1.81873E-7 2.89765E-5 -1.91445E-7 1.49443E-6 -1.01372E-5 -1.16223E-5 -1.41056E-6 -2.71814E-6

388 -1.41299E-6 2.97895E-5 -1.48736E-6 1.49443E-6 -1.01372E-5 -1.21277E-5 -1.0391E-6 -2.51959E-6

389 -1.32505E-6 3.1619E-5 -1.39479E-6 2.42008E-6 -1.04059E-5 -1.20556E-5 -1.48467E-6 -2.71814E-6

390 -9.73304E-7 3.3584E-5 -1.02453E-6 2.32751E-6 -9.80155E-6 -1.14057E-5 -8.90461E-7 -2.51959E-6

391 -2.02854E-6 3.41939E-5 -2.13531E-6 2.14238E-6 -9.26439E-6 -1.15501E-5 -1.0391E-6 -2.58573E-6

392 -3.17173E-6 3.50746E-5 -3.33866E-6 2.97547E-6 -8.66007E-6 -1.16945E-5 -1.48467E-6 -2.85049E-6

393 -4.75459E-6 3.50069E-5 -5.00483E-6 3.34573E-6 -9.19723E-6 -1.21999E-5 -1.33628E-6 -2.32103E-6

394 -6.60126E-6 3.63621E-5 -6.9487E-6 2.79034E-6 -1.0003E-5 -1.30663E-5 -1.63322E-6 -2.05627E-6

395 -5.0184E-6 3.68364E-5 -5.28253E-6 4.82677E-6 -1.10102E-5 -1.29219E-5 -1.70783E-6 -2.05627E-6

396 -3.78729E-6 3.76494E-5 -3.98662E-6 1.08435E-5 -1.12788E-5 -1.34995E-5 -8.16101E-7 -1.65916E-6

397 -4.84253E-6 3.69719E-5 -5.0974E-6 1.02881E-5 -1.17488E-5 -1.37883E-5 -1.55936E-6 -6.6638E-7

398 -4.49078E-6 3.78528E-5 -4.72714E-6 1.24171E-5 -1.16145E-5 -1.30663E-5 -1.55936E-6 -4.67896E-7

399 -4.75459E-6 3.78528E-5 -5.00483E-6 1.4361E-5 -1.05402E-5 -1.32107E-5 -1.48467E-6 -1.1297E-6

400 -7.56857E-6 3.8056E-5 -7.96692E-6 1.649E-5 -1.02716E-5 -1.28497E-5 -2.30271E-6 -1.1297E-6

401 -7.74445E-6 3.79205E-5 -8.15205E-6 1.73231E-5 -1.06073E-5 -1.20556E-5 -2.59973E-6 -1.39446E-6

402 -8.27207E-6 3.70396E-5 -8.70744E-6 1.649E-5 -1.11445E-5 -1.16223E-5 -2.52503E-6 -1.52681E-6

403 -1.04705E-5 3.81238E-5 -1.10216E-5 1.61197E-5 -1.12117E-5 -1.16223E-5 -2.74819E-6 -1.06349E-6

404 -9.41525E-6 3.82593E-5 -9.91079E-6 1.649E-5 -1.12117E-5 -1.11891E-5 -1.93099E-6 -1.32826E-6

405 -1.09102E-5 3.59555E-5 -1.14844E-5 1.69528E-5 -1.0943E-5 -1.08281E-5 -1.63322E-6 -9.97354E-7

406 -1.16137E-5 3.72429E-5 -1.22249E-5 1.74156E-5 -1.18159E-5 -1.06837E-5 -1.48467E-6 -9.97354E-7

407 -1.09102E-5 3.5549E-5 -1.14844E-5 1.76008E-5 -1.05402E-5 -9.02304E-6 -9.64821E-7 -1.79158E-6

408 -1.09982E-5 3.39905E-5 -1.1577E-5 1.65825E-5 -9.80155E-6 -8.73431E-6 -2.96004E-7 -1.32826E-6

409 -1.13498E-5 3.37195E-5 -1.19472E-5 1.75082E-5 -8.25717E-6 -8.66203E-6 -4.44639E-7 -6.6638E-7

410 -1.07343E-5 3.24321E-5 -1.12993E-5 1.83413E-5 -7.38421E-6 -7.72343E-6 -8.16101E-7 -1.52681E-6

411 -8.88763E-6 3.43971E-5 -9.3554E-6 1.90818E-5 -7.45137E-6 -7.65122E-6 -1.63322E-6 -1.59302E-6

412 -6.68921E-6 3.29064E-5 -7.04127E-6 1.84339E-5 -6.98137E-6 -6.06282E-6 -1.78253E-6 -1.72537E-6

413 -5.0184E-6 3.36517E-5 -5.28253E-6 1.76008E-5 -7.85427E-6 -5.05201E-6 -1.41056E-6 -1.99013E-6

414 -4.84253E-6 3.09414E-5 -5.0974E-6 2.04703E-5 -8.19001E-6 -4.97981E-6 -8.90461E-7 -1.32826E-6

415 -4.75459E-6 3.12125E-5 -5.00483E-6 2.14885E-5 -9.33153E-6 -5.84622E-6 -6.6755E-7 -1.79158E-6

416 -3.96316E-6 3.00606E-5 -4.17175E-6 2.13034E-5 -8.86149E-6 -5.70183E-6 -6.6755E-7 -2.45338E-6

417 -3.0838E-6 2.95186E-5 -3.2461E-6 2.33398E-5 -7.98853E-6 -8.37324E-6 2.98454E-7 -2.05627E-6

418 -4.13904E-6 2.9112E-5 -4.35688E-6 2.34324E-5 -8.66007E-6 -1.09002E-5 7.5543E-8 -2.9167E-6

419 -3.52347E-6 2.87732E-5 -3.70892E-6 2.47283E-5 -8.32433E-6 -1.29941E-5 8.92912E-7 -2.71814E-6

420 -3.87522E-6 2.73503E-5 -4.07918E-6 2.42655E-5 -9.19723E-6 -1.39328E-5 1.33873E-6 -2.32103E-6

421 -2.64411E-6 2.70792E-5 -2.78327E-6 2.41729E-5 -1.05402E-5 -1.47269E-5 3.7273E-7 -2.05627E-6

422 -1.50092E-6 2.55886E-5 -1.57992E-6 2.34324E-5 -1.0473E-5 -1.50879E-5 1.04155E-6 -2.78428E-6

423 -3.57747E-7 2.56564E-5 -3.76576E-7 2.52837E-5 -1.11445E-5 -1.52323E-5 5.21365E-7 -2.51959E-6

424 5.21622E-7 2.39624E-5 5.49076E-7 2.55614E-5 -1.07416E-5 -1.49436E-5 6.70001E-7 -2.18862E-6

425 4.65466E-6 2.14553E-5 4.89964E-6 2.93565E-5 -1.00701E-5 -1.42215E-5 3.7273E-7 -1.85771E-6

426 7.82038E-6 2.36914E-5 8.23198E-6 3.05599E-5 -9.39867E-6 -1.39328E-5 -3.70279E-7 -1.52681E-6

427 1.22172E-5 2.37591E-5 1.28602E-5 3.09301E-5 -9.33153E-6 -1.39328E-5 -1.47368E-7 -1.65916E-6

428 1.29208E-5 2.07778E-5 1.36008E-5 3.25038E-5 -8.25717E-6 -1.34273E-5 -5.9319E-7 -1.65916E-6

429 1.28328E-5 1.97614E-5 1.35082E-5 3.29666E-5 -8.25717E-6 -1.34995E-5 -1.26201E-6 -1.72537E-6

430 1.30966E-5 1.89483E-5 1.37859E-5 3.34294E-5 -8.12291E-6 -1.32107E-5 -1.85638E-6 -2.45338E-6

431 1.22172E-5 1.81352E-5 1.28602E-5 3.48179E-5 -7.98853E-6 -1.24165E-5 -2.07955E-6 -2.65194E-6

432 1.22172E-5 1.78642E-5 1.28602E-5 3.47253E-5 -7.51853E-6 -1.25609E-5 -2.97136E-6 -2.45338E-6

433 1.20413E-5 1.70511E-5 1.26751E-5 3.78725E-5 -7.51853E-6 -1.16223E-5 -2.52503E-6 -1.92392E-6

434 1.18655E-5 1.63058E-5 1.249E-5 3.87982E-5 -7.45137E-6 -1.12613E-5 -2.59973E-6 -1.92392E-6

435 1.04585E-5 1.65091E-5 1.10089E-5 4.14826E-5 -8.25717E-6 -1.11891E-5 -2.82205E-6 -1.4606E-6

436 1.5295E-5 1.61025E-5 1.61E-5 4.42595E-5 -9.06291E-6 -1.02504E-5 -2.1534E-6 -1.19591E-6

437 1.69659E-5 1.61025E-5 1.78588E-5 4.5E-5 -9.13001E-6 -1.06115E-5 -1.78253E-6 -1.19591E-6

438 1.74055E-5 1.55604E-5 1.83216E-5 4.25934E-5 -9.80155E-6 -1.07559E-5 -6.6755E-7 -7.98798E-7

439 2.0835E-5 1.36632E-5 2.19316E-5 4.46298E-5 -1.01372E-5 -1.03949E-5 1.183E-9 -1.26205E-6

440 2.30335E-5 1.49507E-5 2.42458E-5 3.91684E-5 -9.80155E-6 -1.14778E-5 1.33873E-6 -9.31145E-7

441 2.6463E-5 1.26468E-5 2.78558E-5 3.75023E-5 -1.00701E-5 -1.12613E-5 8.18552E-7 -4.01687E-7

442 2.90132E-5 1.08174E-5 3.05402E-5 3.64841E-5 -9.60011E-6 -1.14057E-5 1.33873E-6 -1.32826E-6

443 3.14754E-5 7.5651E-6 3.3132E-5 3.72246E-5 -9.13001E-6 -1.16945E-5 5.95641E-7 -2.18862E-6

444 3.36738E-5 4.1094E-6 3.54461E-5 3.86131E-5 -8.59285E-6 -1.21999E-5 3.7273E-7 -1.52681E-6

445 3.4905E-5 -1.59336E-7 3.67421E-5 3.94461E-5 -8.92859E-6 -1.20556E-5 1.183E-9 -2.12248E-6

446 3.54325E-5 -1.78552E-6 3.72974E-5 4.15751E-5 -8.59285E-6 -1.21277E-5 -8.90461E-7 -1.99013E-6

447 3.77189E-5 -4.83462E-6 3.97041E-5 4.40744E-5 -9.8687E-6 -1.15501E-5 -9.64821E-7 -2.05627E-6

448 3.70154E-5 -5.44443E-6 3.89636E-5 4.06495E-5 -1.05402E-5 -1.23443E-5 -9.64821E-7 -2.45338E-6

449 3.56085E-5 -6.5963E-6 3.74826E-5 4.10197E-5 -1.01372E-5 -1.32829E-5 -9.64821E-7 -2.25482E-6

450 3.78069E-5 -7.95147E-6 3.97967E-5 4.04644E-5 -9.93584E-6 -1.39328E-5 -1.11337E-6 -2.71814E-6

451 1.72685E-5 -1.00519E-5 3.98893E-5 3.81502E-5 -9.60011E-6 -1.39328E-5 -2.96004E-7 -2.71814E-6

452 1.72685E-5 -1.16104E-5 4.72945E-5 3.5651E-5 -9.60011E-6 -1.38605E-5 -2.96004E-7 -2.12248E-6

453 1.45417E-5 -1.37787E-5 4.36844E-5 3.49104E-5 -9.53287E-6 -1.36439E-5 2.98454E-7 -2.45338E-6

454 1.13812E-5 -1.36431E-5 4.20183E-5 3.27815E-5 -9.7344E-6 -1.27775E-5 1.78464E-6 -2.18862E-6

455 9.33601E-6 -1.3101E-5 4.27588E-5 3.29666E-5 -1.00701E-5 -1.25609E-5 2.89911E-6 -1.92392E-6

456 7.9107E-6 -1.7302E-5 4.29439E-5 3.31517E-5 -9.7344E-6 -1.15501E-5 2.00755E-6 -2.18862E-6

457 7.66278E-6 -1.75054E-5 4.27588E-5 3.29666E-5 -8.59285E-6 -1.16945E-5 1.26446E-6 -2.58573E-6

458 7.35293E-6 -1.87927E-5 4.56283E-5 3.31517E-5 -8.72717E-6 -1.20556E-5 1.93319E-6 -2.45338E-6

459 7.53883E-6 -2.11643E-5 4.2296E-5 3.59287E-5 -9.13001E-6 -1.14778E-5 1.41301E-6 -1.92392E-6

460 6.73322E-6 -2.14352E-5 4.36844E-5 3.49105E-5 -8.59285E-6 -1.16223E-5 1.11582E-6 -1.99013E-6

461 3.85148E-6 -2.21129E-5 4.30365E-5 3.33368E-5 -8.99575E-6 -1.09002E-5 -3.70279E-7 -1.36136E-6

462 2.705E-6 -2.29259E-5 4.23885E-5 3.29666E-5 -9.66726E-6 -1.06115E-5 -2.21728E-7 -8.64936E-7

463 1.21766E-6 -2.3468E-5 4.07223E-5 3.11153E-5 -9.06291E-6 -1.16223E-5 7.5543E-8 -4.67896E-7

464 1.03175E-6 -2.46198E-5 4.00744E-5 3.20409E-5 -8.86149E-6 -1.19111E-5 -1.0391E-6 8.55784E-7

465 -1.63307E-6 -2.44844E-5 3.9519E-5 3.31517E-5 -7.85427E-6 -1.16223E-5 -1.33628E-6 1.05434E-6

466 -5.79538E-7 -2.62461E-5 4.08149E-5 3.53733E-5 -6.37706E-6 -1.09002E-5 -1.78253E-6 1.91477E-6

467 -1.94293E-6 -2.78045E-5 3.94264E-5 3.79651E-5 -6.51131E-6 -1.06837E-5 -1.85638E-6 2.04712E-6

468 -1.50912E-6 -2.95662E-5 3.92413E-5 4.24082E-5 -7.45137E-6 -9.60063E-6 -2.22801E-6 1.65001E-6

469 -1.01334E-6 -2.83466E-5 3.96116E-5 4.63885E-5 -8.72717E-6 -1.01783E-5 -2.1534E-6 2.60189E-7

470 -1.94293E-6 -3.03115E-5 3.92413E-5 4.72216E-5 -1.05402E-5 -1.05393E-5 -1.63322E-6 -2.03132E-7

471 -5.79538E-7 -3.00405E-5 3.99818E-5 4.70365E-5 -1.16816E-5 -1.08281E-5 -2.22801E-6 -1.19591E-6

472 1.15569E-6 -2.94984E-5 3.85008E-5 4.64811E-5 -1.16816E-5 -1.14057E-5 -1.93099E-6 -1.99013E-6

473 2.82894E-6 -2.81433E-5 3.83157E-5 4.36116E-5 -1.15473E-5 -1.21999E-5 -2.22801E-6 -2.45338E-6

474 3.75853E-6 -2.8211E-5 3.62792E-5 4.30562E-5 -1.16145E-5 -1.33551E-5 -2.1534E-6 -2.9167E-6

475 3.26275E-6 -2.794E-5 3.35948E-5 4.08346E-5 -1.10773E-5 -1.36439E-5 -1.78253E-6 -3.2476E-6

476 4.37826E-6 -2.50942E-5 3.1003E-5 3.9909E-5 -1.16145E-5 -1.35717E-5 -2.00485E-6 -2.9167E-6

477 5.43178E-6 -2.43488E-5 3.28543E-5 4.15751E-5 -1.14802E-5 -1.36439E-5 -1.55936E-6 -3.37995E-6

478 6.85718E-6 -2.50264E-5 3.48907E-5 4.10198E-5 -1.05402E-5 -1.39328E-5 -1.0391E-6 -3.77706E-6

479 7.72473E-6 -2.33325E-5 3.62792E-5 4.25934E-5 -1.02044E-5 -1.39328E-5 -1.85638E-6 -2.85049E-6

480 7.04308E-6 -2.41455E-5 3.84082E-5 4.36116E-5 -9.93584E-6 -1.23443E-5 -1.18765E-6 -3.71085E-6

481 7.29092E-6 -2.19096E-5 4.09075E-5 4.26859E-5 -9.46582E-6 -1.18389E-5 -1.41056E-6 -2.65194E-6

482 1.01416E-5 -2.25194E-5 4.3129E-5 4.75919E-5 -9.93584E-6 -1.06837E-5 -1.26201E-6 -2.12248E-6

483 1.1567E-5 -1.73698E-5 4.26662E-5 4.6296E-5 -1.06073E-5 -1.04671E-5 -3.70279E-7 -1.39446E-6

484 1.21868E-5 -2.09609E-5 4.4425E-5 4.70365E-5 -1.05402E-5 -1.10447E-5 -1.47368E-7 -6.6638E-7

485 1.54094E-5 -1.70988E-5 4.55357E-5 5.00911E-5 -1.08759E-5 -1.11891E-5 -4.44639E-7 -5.34034E-7

486 1.67727E-5 -1.91993E-5 4.69242E-5 5.42566E-5 -1.16816E-5 -1.16223E-5 -8.16101E-7 -9.97354E-7

487 1.72065E-5 -1.93347E-5 4.57209E-5 5.65707E-5 -1.11445E-5 -1.23443E-5 -5.18999E-7 -6.00243E-7

488 1.67727E-5 -1.79796E-5 4.3129E-5 6.12915E-5 -1.08087E-5 -1.23443E-5 -1.33628E-6 -1.06349E-6

489 1.89418E-5 -1.71665E-5 4.40547E-5 6.21246E-5 -1.03387E-5 -1.24887E-5 -9.64821E-7 -1.32826E-6

490 1.97474E-5 -1.44562E-5 4.72019E-5 6.36982E-5 -9.53287E-6 -1.21999E-5 -5.18999E-7 -1.26205E-6

491 1.91897E-5 -1.23557E-5 5.00714E-5 6.49941E-5 -9.13001E-6 -1.19111E-5 -3.70279E-7 -2.38717E-6

492 2.14826E-5 -9.78092E-6 5.00714E-5 6.4809E-5 -8.92859E-6 -1.21277E-5 7.44276E-7 -2.58573E-6

493 2.40235E-5 -7.34166E-6 5.32186E-5 6.52718E-5 -8.72717E-6 -1.24887E-5 -7.30925E-8 -2.65194E-6

494 2.55108E-5 -6.39304E-6 5.51625E-5 6.61975E-5 -9.19723E-6 -1.27053E-5 -3.70279E-7 -3.18139E-6

495 2.72461E-5 -2.73413E-6 5.74766E-5 6.52718E-5 -9.7344E-6 -1.18389E-5 -7.4191E-7 -3.51237E-6

496 2.86095E-5 -1.78552E-6 5.78469E-5 6.49941E-5 -9.8687E-6 -1.25609E-5 -2.00485E-6 -3.5785E-6

497 3.00348E-5 -6.33638E-7 6.0809E-5 6.77711E-5 -1.02716E-5 -1.09725E-5 -2.30271E-6 -3.90941E-6

498 3.29475E-5 1.94115E-6 6.45116E-5 6.86042E-5 -9.8687E-6 -1.10447E-5 -2.00485E-6 -3.37995E-6

499 3.49306E-5 3.6351E-6 6.69183E-5 6.76785E-5 -1.03387E-5 -1.07559E-5 -1.93099E-6 -3.18139E-6

500 3.52405E-5 7.29401E-6 7.10837E-5 7.1196E-5 -1.11445E-5 -1.18389E-5 -2.74819E-6 -2.78428E-6

501 3.65419E-5 8.37811E-6 7.22871E-5 7.50837E-5 -1.06073E-5 -1.24165E-5 -2.82205E-6 -2.51959E-6

502 3.72856E-5 1.10207E-5 7.28425E-5 7.50837E-5 -1.06073E-5 -1.24887E-5 -2.07955E-6 -1.85771E-6

503 3.74715E-5 1.44763E-5 7.24722E-5 7.63796E-5 -1.0003E-5 -1.21999E-5 -1.11337E-6 -1.59302E-6

504 3.70997E-5 1.75932E-5 7.19168E-5 7.65648E-5 -9.53287E-6 -1.27053E-5 -3.70279E-7 -2.05627E-6

505 3.70997E-5 2.03713E-5 7.30276E-5 8.01748E-5 -8.59285E-6 -1.28497E-5 -7.30925E-8 -1.79158E-6

506 3.80912E-5 2.34203E-5 7.47863E-5 8.11004E-5 -8.05569E-6 -1.19833E-5 1.183E-9 -2.25482E-6

507 3.98884E-5 2.63339E-5 7.55268E-5 8.21187E-5 -7.24995E-6 -1.26331E-5 -5.18999E-7 -1.72537E-6

508 4.29251E-5 2.95863E-5 7.53417E-5 8.35071E-5 -8.39143E-6 -1.32107E-5 -1.0391E-6 -9.31145E-7

509 4.72012E-5 3.00606E-5 7.69153E-5 8.85057E-5 -7.85427E-6 -1.21999E-5 -1.33628E-6 -4.67896E-7

510 4.70772E-5 3.19578E-5 7.84889E-5 9.08198E-5 -7.92143E-6 -1.27775E-5 -1.55936E-6 -1.36923E-7

511 5.11674E-5 3.66331E-5 8.06179E-5 9.06347E-5 -8.19001E-6 -1.27053E-5 -1.63322E-6 3.92535E-7

512 5.37702E-5 3.79882E-5 8.50611E-5 9.07272E-5 -8.45859E-6 -1.20556E-5 -9.64821E-7 1.27771E-7

513 5.67449E-5 4.09018E-5 8.70975E-5 9.10975E-5 -7.58563E-6 -1.27775E-5 -2.00485E-6 -7.32589E-7

514 5.69308E-5 4.28668E-5 9.01521E-5 9.10975E-5 -7.85427E-6 -1.32829E-5 -2.30271E-6 -9.31145E-7

515 5.92858E-5 4.37477E-5 9.21886E-5 9.34116E-5 -9.66726E-6 -1.31385E-5 -1.93099E-6 -1.85771E-6

516 6.23224E-5 4.67968E-5 9.26514E-5 9.50778E-5 -9.60011E-6 -1.35717E-5 -2.1534E-6 -1.79158E-6

517 6.60407E-5 4.8965E-5 9.37622E-5 9.65588E-5 -9.66726E-6 -1.27053E-5 -2.89674E-6 -2.51959E-6

518 6.68466E-5 5.1472E-5 9.46878E-5 9.65588E-5 -1.05402E-5 -1.26331E-5 -2.45118E-6 -3.71085E-6

519 6.67842E-5 5.26239E-5 9.47804E-5 9.8873E-5 -9.66726E-6 -1.29219E-5 -2.89674E-6 -4.0419E-6

520 6.65983E-5 5.5402E-5 9.59837E-5 1.00261E-4 -1.02716E-5 -1.20556E-5 -2.37656E-6 -4.30645E-6

521 6.7156E-5 5.90609E-5 9.62614E-5 1.02205E-4 -1.05402E-5 -1.23443E-5 -1.48467E-6 -3.90941E-6

522 6.74037E-5 6.18388E-5 9.69094E-5 1.03409E-4 -9.53287E-6 -1.10447E-5 -1.26201E-6 -4.0419E-6

523 6.80862E-5 6.25165E-5 9.79276E-5 1.03871E-4 -9.19723E-6 -1.01783E-5 -1.63322E-6 -4.571E-6

524 6.90774E-5 6.80046E-5 9.9964E-5 1.02946E-4 -8.32433E-6 -1.01061E-5 -1.70783E-6 -3.71085E-6

525 7.05029E-5 7.09863E-5 1.00705E-4 1.02113E-4 -8.32433E-6 -1.00339E-5 -1.85638E-6 -3.44616E-6

526 7.05029E-5 7.51874E-5 1.01815E-4 1.01095E-4 -8.66007E-6 -1.06115E-5 -2.45118E-6 -2.98284E-6

527 7.10606E-5 7.69485E-5 9.9964E-5 9.8225E-5 -7.58563E-6 -1.05393E-5 -2.22801E-6 -2.9167E-6

528 7.23002E-5 8.12856E-5 1.06166E-4 9.81324E-5 -8.79433E-6 -1.11169E-5 -2.1534E-6 -2.65194E-6

529 7.27961E-5 8.38604E-5 1.07462E-4 9.79473E-5 -9.33153E-6 -1.01783E-5 -1.63322E-6 -2.38717E-6

530 7.46544E-5 8.43343E-5 1.07647E-4 9.92432E-5 -8.66007E-6 -1.09002E-5 -5.9319E-7 -2.25482E-6

531 7.52745E-5 8.61637E-5 1.07277E-4 1.00909E-4 -7.78711E-6 -1.14778E-5 -5.9319E-7 -1.79158E-6

532 7.61423E-5 8.7316E-5 1.07277E-4 1.03224E-4 -7.45137E-6 -1.09002E-5 -3.70279E-7 -2.71814E-6

533 7.68235E-5 8.81962E-5 1.09591E-4 1.04057E-4 -5.97416E-6 -1.11169E-5 2.98454E-7 -2.85049E-6

534 7.65758E-5 9.15171E-5 1.0885E-4 1.01835E-4 -5.57129E-6 -1.17667E-5 -1.0391E-6 -1.85771E-6

535 7.63282E-5 9.28719E-5 1.09961E-4 1.02298E-4 -5.36984E-6 -1.19833E-5 -1.33628E-6 -1.99013E-6

536 7.68859E-5 9.4769E-5 1.13386E-4 1.01557E-4 -5.50413E-6 -1.16223E-5 -1.48467E-6 -1.99013E-6

537 7.68859E-5 9.51759E-5 1.13386E-4 9.8873E-5 -6.37706E-6 -1.23443E-5 -1.93099E-6 -1.39446E-6

538 7.60799E-5 9.52436E-5 1.14682E-4 1.02113E-4 -6.91422E-6 -1.19111E-5 -2.59973E-6 -4.01687E-7

539 7.70101E-5 9.8903E-5 1.13293E-4 1.00817E-4 -7.04854E-6 -1.19111E-5 -1.63322E-6 -4.67896E-7

540 7.65141E-5 1.01477E-4 1.09406E-4 1.04334E-4 -7.85427E-6 -1.30663E-5 -1.93099E-6 5.24882E-7

541 7.75053E-5 1.02087E-4 1.0774E-4 1.05815E-4 -9.13001E-6 -1.23443E-5 -2.37656E-6 1.12055E-6

542 7.91167E-5 1.0412E-4 1.07277E-4 1.04797E-4 -8.32433E-6 -1.30663E-5 -2.89674E-6 1.05434E-6

543 8.06046E-5 1.05474E-4 1.07092E-4 1.0563E-4 -9.93584E-6 -1.28497E-5 -2.82205E-6 1.9398E-7

544 8.26488E-5 1.05339E-4 1.03204E-4 1.05723E-4 -1.04059E-5 -1.21999E-5 -2.1534E-6 -9.31145E-7

545 8.20911E-5 1.05543E-4 1.02E-4 1.04057E-4 -1.03387E-5 -1.20556E-5 -2.00485E-6 -1.19591E-6

546 8.24635E-5 1.06288E-4 9.95938E-5 1.02946E-4 -9.53287E-6 -1.20556E-5 -2.00485E-6 -9.31145E-7

547 8.14723E-5 1.0744E-4 9.5706E-5 1.00447E-4 -1.02716E-5 -1.13335E-5 -2.22801E-6 -1.4606E-6

548 8.0728E-5 1.07101E-4 9.37622E-5 9.85953E-5 -1.05402E-5 -1.16223E-5 -2.07955E-6 -2.12248E-6

549 8.14099E-5 1.0893E-4 9.10778E-5 9.79473E-5 -9.26439E-6 -1.19111E-5 -2.45118E-6 -1.79158E-6

550 8.04187E-5 1.09269E-4 9.1448E-5 9.71142E-5 -9.06291E-6 -1.13335E-5 -1.70783E-6 -1.79158E-6

551 7.97368E-5 1.10896E-4 8.98744E-5 9.74845E-5 -8.19001E-6 -1.14057E-5 -2.1534E-6 -9.97354E-7

552 8.02945E-5 1.13064E-4 9.28365E-5 9.85953E-5 -7.92143E-6 -1.04671E-5 -1.33628E-6 -1.32826E-6

553 7.78778E-5 1.13944E-4 9.26514E-5 9.80399E-5 -8.05569E-6 -9.81721E-6 2.24179E-7 -1.1297E-6

554 7.67E-5 1.153E-4 9.33919E-5 9.86878E-5 -8.45859E-6 -1.03949E-5 6.70001E-7 -1.19591E-6

555 7.67624E-5 1.16791E-4 9.58912E-5 9.81324E-5 -9.39867E-6 -1.02504E-5 2.98454E-7 -1.4606E-6

556 7.47168E-5 1.17265E-4 9.72797E-5 9.89655E-5 -9.80155E-6 -1.01061E-5 3.7273E-7 -1.72537E-6

557 7.23625E-5 1.16994E-4 9.77425E-5 9.85953E-5 -1.00701E-5 -1.07559E-5 2.24179E-7 -3.35478E-7

558 6.89533E-5 1.18755E-4 9.47804E-5 9.8225E-5 -9.46582E-6 -1.17667E-5 1.49819E-7 -1.1297E-6

559 6.69084E-5 1.21127E-4 9.18183E-5 9.79473E-5 -9.39867E-6 -1.24165E-5 2.98454E-7 -1.19591E-6

560 6.59789E-5 1.2194E-4 8.83008E-5 9.78547E-5 -9.60011E-6 -1.34995E-5 -1.47368E-7 -1.1297E-6

561 6.27563E-5 1.24312E-4 8.79306E-5 9.78547E-5 -9.8687E-6 -1.36439E-5 -1.11337E-6 -1.32826E-6

562 6.12069E-5 1.24244E-4 8.61718E-5 9.72068E-5 -1.03387E-5 -1.36439E-5 -4.44639E-7 -1.65916E-6

563 6.07111E-5 1.24718E-4 8.4228E-5 9.68365E-5 -9.39867E-6 -1.37883E-5 -1.33628E-6 -1.85771E-6

564 5.83562E-5 1.2133E-4 8.36263E-5 9.78085E-5 -8.8951E-6 -1.49436E-5 -1.11337E-6 -2.71814E-6

565 5.77364E-5 1.19772E-4 8.34874E-5 9.93358E-5 -9.66726E-6 -1.55934E-5 -7.30925E-8 -3.18139E-6

566 5.73026E-5 1.19298E-4 8.29321E-5 9.98912E-5 -1.02044E-5 -1.71096E-5 1.183E-9 -2.78428E-6

567 5.69308E-5 1.18214E-4 8.18213E-5 1.02668E-4 -1.0943E-5 -1.73984E-5 3.7273E-7 -3.64471E-6

568 5.74885E-5 1.21669E-4 8.19138E-5 1.03131E-4 -1.10773E-5 -1.78316E-5 1.04155E-6 -3.64471E-6

569 5.50097E-5 1.20585E-4 8.2099E-5 1.00261E-4 -1.19502E-5 -1.95644E-5 -7.30925E-8 -3.31381E-6

570 5.46998E-5 1.19365E-4 8.1451E-5 9.97986E-5 -1.22188E-5 -1.97809E-5 -8.16101E-7 -3.77706E-6

571 5.39562E-5 1.19908E-4 8.43205E-5 9.9706E-5 -1.14802E-5 -2.05752E-5 -1.55936E-6 -3.71085E-6

572 5.33984E-5 1.21533E-4 8.51536E-5 9.95209E-5 -1.25545E-5 -2.1225E-5 -2.22801E-6 -3.9754E-6

573 5.16632E-5 1.20043E-4 8.59867E-5 9.98912E-5 -1.31588E-5 -2.05752E-5 -2.89674E-6 -4.17417E-6

574 5.08576E-5 1.19908E-4 8.71901E-5 9.93358E-5 -1.3226E-5 -2.13694E-5 -2.97136E-6 -3.9754E-6

575 4.80687E-5 1.20517E-4 8.76529E-5 9.98912E-5 -1.34274E-5 -2.18748E-5 -4.45729E-6 -3.44616E-6

576 4.67674E-5 1.19908E-4 8.64495E-5 9.95209E-5 -1.43675E-5 -2.32467E-5 -3.78856E-6 -4.30645E-6

577 4.72631E-5 1.19162E-4 8.74677E-5 1.00354E-4 -1.30917E-5 -2.44741E-5 -3.41693E-6 -4.0419E-6

578 4.65815E-5 1.17332E-4 9.2096E-5 1.01372E-4 -1.26888E-5 -2.57736E-5 -3.64009E-6 -4.0419E-6

579 4.7511E-5 1.1652E-4 9.21886E-5 1.03131E-4 -1.26888E-5 -2.78675E-5 -4.08558E-6 -5.29958E-6

580 4.7573E-5 1.14623E-4 9.4873E-5 1.03594E-4 -1.26217E-5 -3.01779E-5 -5.12594E-6 -5.82868E-6

581 4.73871E-5 1.13606E-4 9.66317E-5 1.05538E-4 -1.39646E-5 -3.21274E-5 -5.86937E-6 -6.42428E-6

582 4.70152E-5 1.12522E-4 9.82979E-5 1.05723E-4 -1.54419E-5 -3.51597E-5 -6.09161E-6 -7.48319E-6

583 4.84406E-5 1.11166E-4 9.96863E-5 1.07111E-4 -1.68519E-5 -3.86976E-5 -6.16597E-6 -8.40983E-6

584 4.968E-5 1.08253E-4 9.74648E-5 1.09333E-4 -1.88664E-5 -4.22354E-5 -6.83521E-6 -9.40297E-6

585 5.00519E-5 1.0744E-4 1.00982E-4 1.13221E-4 -2.08136E-5 -4.52678E-5 -6.68649E-6 -1.07929E-5

586 4.968E-5 1.06356E-4 9.96863E-5 1.15905E-4 -2.21566E-5 -4.89501E-5 -7.8754E-6 -1.22487E-5

587 5.15393E-5 1.06017E-4 1.01723E-4 1.19793E-4 -2.41038E-5 -5.26324E-5 -8.46944E-6 -1.35721E-5

588 5.00519E-5 1.07033E-4 1.02278E-4 1.20441E-4 -2.65211E-5 -5.62424E-5 -8.98996E-6 -1.46975E-5

589 5.14773E-5 1.07914E-4 1.03204E-4 1.20163E-4 -2.9677E-5 -6.0791E-5 -1.09216E-5 -1.57565E-5

590 5.25308E-5 1.06695E-4 1.0487E-4 1.20903E-4 -3.20272E-5 -6.52678E-5 -1.21105E-5 -1.72122E-5

591 5.38322E-5 1.06288E-4 1.07277E-4 1.21089E-4 -3.5653E-5 -7.06102E-5 -1.37456E-5 -1.80066E-5

592 5.69928E-5 1.05814E-4 1.09221E-4 1.20626E-4 -3.90776E-5 -7.70361E-5 -1.47115E-5 -1.87344E-5

593 5.68689E-5 1.05678E-4 1.12923E-4 1.21551E-4 -4.37778E-5 -8.23069E-5 -1.74611E-5 -2.07865E-5

594 5.80463E-5 1.06017E-4 1.14774E-4 1.24606E-4 -4.89481E-5 -8.80107E-5 -1.87235E-5 -2.25733E-5

595 6.02774E-5 1.07237E-4 1.15237E-4 1.29697E-4 -5.35812E-5 -9.51587E-5 -2.17706E-5 -2.42278E-5

596 6.00915E-5 1.05001E-4 1.20143E-4 1.32196E-4 -5.91544E-5 -1.01873E-4 -2.44459E-5 -2.72065E-5

597 6.12689E-5 1.08185E-4 1.21347E-4 1.33585E-4 -6.58694E-5 -1.09527E-4 -2.62296E-5 -2.98534E-5

598 6.18886E-5 1.07237E-4 1.30973E-4 1.35251E-4 -7.22481E-5 -1.17324E-4 -2.96477E-5 -3.22365E-5

599 6.26943E-5 1.07711E-4 1.294E-4 1.35806E-4 -8.02389E-5 -1.24761E-4 -3.31392E-5 -3.58766E-5

600 6.49253E-5 1.07644E-4 1.30325E-4 1.38954E-4 -9.03111E-5 -1.3292E-4 -3.65572E-5 -3.99134E-5

601 6.7156E-5 1.10015E-4 1.31806E-4 1.40342E-4 -9.96439E-5 -1.41873E-4 -4.16103E-5 -4.29579E-5

602 6.70326E-5 1.12657E-4 1.33935E-4 1.43027E-4 -1.09716E-4 -1.50609E-4 -4.63668E-5 -4.77892E-5

603 6.89533E-5 1.14013E-4 1.37175E-4 1.45711E-4 -1.19519E-4 -1.59995E-4 -5.18644E-5 -5.13635E-5

604 7.13083E-5 1.15774E-4 1.41618E-4 1.48673E-4 -1.30867E-4 -1.69814E-4 -5.78098E-5 -5.52681E-5

605 7.39115E-5 1.18078E-4 1.4458E-4 1.49691E-4 -1.43625E-4 -1.79706E-4 -6.41997E-5 -5.9769E-5

606 7.97985E-5 1.20179E-4 1.51708E-4 1.51357E-4 -1.55712E-4 -1.90969E-4 -7.0293E-5 -6.44015E-5

607 8.41367E-5 1.24922E-4 1.54115E-4 1.52838E-4 -1.68134E-4 -2.02016E-4 -7.77231E-5 -7.00934E-5

608 8.68634E-5 1.27022E-4 1.59391E-4 1.57837E-4 -1.8257E-4 -2.12557E-4 -8.51552E-5 -7.61163E-5

609 9.10156E-5 1.28987E-4 1.6152E-4 1.59596E-4 -1.9647E-4 -2.22737E-4 -9.25106E-5 -8.22049E-5

610 9.60973E-5 1.33933E-4 1.63927E-4 1.63113E-4 -2.10638E-4 -2.33712E-4 -1.00684E-4 -8.82941E-5

611 9.98777E-5 1.35627E-4 1.69758E-4 1.64965E-4 -2.26081E-4 -2.44182E-4 -1.08486E-4 -9.55084E-5

612 1.05455E-4 1.37592E-4 1.72998E-4 1.6589E-4 -2.40183E-4 -2.54722E-4 -1.16809E-4 -1.01597E-4

613 1.08492E-4 1.38405E-4 1.78089E-4 1.68389E-4 -2.55492E-4 -2.64975E-4 -1.26246E-4 -1.08679E-4

614 1.14317E-4 1.40573E-4 1.81607E-4 1.72555E-4 -2.70801E-4 -2.7566E-4 -1.35237E-4 -1.16819E-4

615 1.16176E-4 1.43216E-4 1.88086E-4 1.75702E-4 -2.85371E-4 -2.87574E-4 -1.43411E-4 -1.23571E-4

616 1.19151E-4 1.45587E-4 1.90863E-4 1.78201E-4 -3.00077E-4 -2.99559E-4 -1.52773E-4 -1.31182E-4

617 1.22498E-4 1.49992E-4 1.92992E-4 1.7959E-4 -3.15185E-4 -3.10389E-4 -1.61914E-4 -1.38528E-4

618 1.24418E-4 1.52702E-4 1.99379E-4 1.832E-4 -3.30158E-4 -3.21003E-4 -1.71573E-4 -1.46734E-4

619 1.26526E-4 1.57377E-4 2.04933E-4 1.87365E-4 -3.43923E-4 -3.31761E-4 -1.81159E-4 -1.5428E-4

620 1.26464E-4 1.60968E-4 2.08265E-4 1.93289E-4 -3.57152E-4 -3.41797E-4 -1.91116E-4 -1.62354E-4

621 1.28075E-4 1.62256E-4 2.13634E-4 1.94308E-4 -3.69306E-4 -3.51543E-4 -2.01221E-4 -1.70098E-4

622 1.31297E-4 1.67812E-4 2.18725E-4 2.0125E-4 -3.79914E-4 -3.59197E-4 -2.10807E-4 -1.77908E-4

623 1.33715E-4 1.69235E-4 2.22058E-4 2.0449E-4 -3.8945E-4 -3.65984E-4 -2.21508E-4 -1.85386E-4

624 1.36442E-4 1.71538E-4 2.28722E-4 2.09951E-4 -3.98179E-4 -3.72988E-4 -2.30944E-4 -1.93064E-4

625 1.39602E-4 1.75875E-4 2.35665E-4 2.15227E-4 -4.059E-4 -3.78042E-4 -2.40752E-4 -2.01006E-4

626 1.44188E-4 1.82245E-4 2.37886E-4 2.21429E-4 -4.11877E-4 -3.82157E-4 -2.49075E-4 -2.08815E-4

627 1.48898E-4 1.87394E-4 2.49457E-4 2.25965E-4 -4.16443E-4 -3.85984E-4 -2.5762E-4 -2.15765E-4

628 1.55653E-4 1.93221E-4 2.54733E-4 2.34018E-4 -4.20136E-4 -3.88294E-4 -2.66016E-4 -2.22979E-4

629 1.63337E-4 2.01216E-4 2.60194E-4 2.40498E-4 -4.21747E-4 -3.90822E-4 -2.73224E-4 -2.30458E-4

630 1.70588E-4 2.08466E-4 2.64267E-4 2.48181E-4 -4.22419E-4 -3.94864E-4 -2.80209E-4 -2.36944E-4

631 1.77157E-4 2.17004E-4 2.71765E-4 2.5392E-4 -4.22486E-4 -3.97825E-4 -2.8712E-4 -2.43562E-4

632 1.84842E-4 2.23983E-4 2.78893E-4 2.57067E-4 -4.2215E-4 -4.01651E-4 -2.92618E-4 -2.4899E-4

633 1.94448E-4 2.34079E-4 2.81299E-4 2.6549E-4 -4.2121E-4 -4.0555E-4 -2.98117E-4 -2.5435E-4

634 2.0362E-4 2.42346E-4 2.87686E-4 2.69841E-4 -4.22486E-4 -4.08077E-4 -3.02204E-4 -2.58916E-4

635 2.14217E-4 2.49663E-4 2.9287E-4 2.78172E-4 -4.23024E-4 -4.11037E-4 -3.06513E-4 -2.63351E-4

636 2.25248E-4 2.58065E-4 2.95925E-4 2.84836E-4 -4.22821E-4 -4.12842E-4 -3.11492E-4 -2.66793E-4

637 2.36651E-4 2.66602E-4 3.00183E-4 2.90668E-4 -4.25239E-4 -4.14359E-4 -3.15728E-4 -2.71624E-4

638 2.48673E-4 2.73243E-4 3.04718E-4 2.98351E-4 -4.27454E-4 -4.16886E-4 -3.19889E-4 -2.75397E-4

639 2.59518E-4 2.82187E-4 3.08791E-4 3.01868E-4 -4.29804E-4 -4.20423E-4 -3.24051E-4 -2.795E-4

640 2.70239E-4 2.9147E-4 3.09532E-4 3.11125E-4 -4.34505E-4 -4.24178E-4 -3.27618E-4 -2.8314E-4

641 2.81829E-4 2.98449E-4 3.17585E-4 3.15846E-4 -4.38332E-4 -4.29016E-4 -3.30515E-4 -2.8645E-4

642 2.94099E-4 3.06919E-4 3.23139E-4 3.23529E-4 -4.41891E-4 -4.34719E-4 -3.34156E-4 -2.89229E-4

643 3.04758E-4 3.13153E-4 3.24249E-4 3.29545E-4 -4.46726E-4 -4.41001E-4 -3.37574E-4 -2.92406E-4

644 3.16285E-4 3.21757E-4 3.25823E-4 3.37413E-4 -4.51761E-4 -4.47498E-4 -3.41513E-4 -2.95649E-4

645 3.26635E-4 3.29143E-4 3.28137E-4 3.43245E-4 -4.57133E-4 -4.53852E-4 -3.45376E-4 -2.98098E-4

646 3.37852E-4 3.36867E-4 3.32951E-4 3.4991E-4 -4.63176E-4 -4.59845E-4 -3.48275E-4 -3.00414E-4

647 3.47209E-4 3.4405E-4 3.35172E-4 3.56482E-4 -4.68279E-4 -4.6555E-4 -3.51767E-4 -3.02599E-4

648 3.57931E-4 3.51571E-4 3.38504E-4 3.57407E-4 -4.73585E-4 -4.7183E-4 -3.56226E-4 -3.04319E-4

649 3.66545E-4 3.56585E-4 3.41559E-4 3.62499E-4 -4.78688E-4 -4.7674E-4 -3.60313E-4 -3.07033E-4

650 3.73671E-4 3.63632E-4 3.47113E-4 3.66942E-4 -4.83656E-4 -4.81433E-4 -3.64622E-4 -3.09746E-4

651 3.80674E-4 3.67833E-4 3.52945E-4 3.70274E-4 -4.87283E-4 -4.86848E-4 -3.67446E-4 -3.12658E-4

652 3.865E-4 3.73524E-4 3.54703E-4 3.73421E-4 -4.91445E-4 -4.90603E-4 -3.70715E-4 -3.15702E-4

653 3.92821E-4 3.81249E-4 3.60905E-4 3.76383E-4 -4.96145E-4 -4.95223E-4 -3.7391E-4 -3.18549E-4

654 3.99019E-4 3.8423E-4 3.67663E-4 3.80179E-4 -5.0051E-4 -4.99411E-4 -3.77551E-4 -3.21328E-4

655 4.0472E-4 3.87754E-4 3.69606E-4 3.84529E-4 -5.04875E-4 -5.01794E-4 -3.81341E-4 -3.24903E-4

656 4.12405E-4 3.9087E-4 3.76086E-4 3.8638E-4 -5.0877E-4 -5.05476E-4 -3.84536E-4 -3.2788E-4

657 4.17425E-4 3.9602E-4 3.79881E-4 3.88602E-4 -5.12664E-4 -5.07642E-4 -3.87137E-4 -3.31057E-4

658 4.23808E-4 3.99272E-4 3.83121E-4 3.89528E-4 -5.15618E-4 -5.09664E-4 -3.88178E-4 -3.33771E-4

659 4.27588E-4 4.04083E-4 3.83399E-4 3.91101E-4 -5.18506E-4 -5.10747E-4 -3.90778E-4 -3.35757E-4

660 4.33289E-4 4.08691E-4 3.88767E-4 3.94341E-4 -5.20251E-4 -5.10747E-4 -3.9271E-4 -3.38139E-4

661 4.34777E-4 4.14179E-4 3.91822E-4 3.98506E-4 -5.2193E-4 -5.10097E-4 -3.94047E-4 -3.40654E-4

662 4.39301E-4 4.19329E-4 3.94692E-4 4.01561E-4 -5.23139E-4 -5.10025E-4 -3.95756E-4 -3.42176E-4

663 4.43515E-4 4.21971E-4 3.95987E-4 4.02116E-4 -5.24011E-4 -5.08436E-4 -3.97688E-4 -3.44956E-4

664 4.43763E-4 4.25765E-4 3.98209E-4 4.02857E-4 -5.24145E-4 -5.07642E-4 -3.991E-4 -3.4714E-4

665 4.4841E-4 4.29763E-4 3.98487E-4 4.0767E-4 -5.24549E-4 -5.07281E-4 -3.99992E-4 -3.48927E-4

666 4.48845E-4 4.3244E-4 3.9895E-4 4.10818E-4 -5.25624E-4 -5.06775E-4 -4.01515E-4 -3.49821E-4

667 4.51632E-4 4.3742E-4 3.98764E-4 4.11373E-4 -5.27302E-4 -5.05693E-4 -4.00883E-4 -3.50714E-4

668 4.53864E-4 4.41893E-4 4.01819E-4 4.14057E-4 -5.27973E-4 -5.05693E-4 -4.02072E-4 -3.50979E-4

669 4.53802E-4 4.41824E-4 4.04226E-4 4.14798E-4 -5.29383E-4 -5.04176E-4 -4.01552E-4 -3.51972E-4

670 4.55166E-4 4.41824E-4 4.08021E-4 4.1489E-4 -5.31398E-4 -5.02949E-4 -4.01627E-4 -3.52104E-4

671 4.56095E-4 4.40605E-4 4.07651E-4 4.16279E-4 -5.32204E-4 -5.01938E-4 -4.02592E-4 -3.52766E-4

672 4.56776E-4 4.36065E-4 4.10243E-4 4.16742E-4 -5.34083E-4 -5.01361E-4 -4.04228E-4 -3.52832E-4

673 4.56839E-4 4.30847E-4 4.10428E-4 4.15446E-4 -5.35628E-4 -5.00422E-4 -4.05267E-4 -3.53163E-4

674 4.57582E-4 4.26036E-4 4.08947E-4 4.13039E-4 -5.36769E-4 -4.98978E-4 -4.0616E-4 -3.527E-4

675 4.60309E-4 4.20751E-4 4.06725E-4 4.12299E-4 -5.38113E-4 -4.98183E-4 -4.06902E-4 -3.52502E-4

676 4.61982E-4 4.1235E-4 4.09409E-4 4.09985E-4 -5.39321E-4 -4.95802E-4 -4.06754E-4 -3.52832E-4

677 4.61549E-4 4.051E-4 4.05892E-4 4.06097E-4 -5.38649E-4 -4.93996E-4 -4.07868E-4 -3.53163E-4

678 4.58574E-4 3.96359E-4 4.05985E-4 4.03783E-4 -5.39388E-4 -4.91685E-4 -4.07794E-4 -3.53163E-4

679 4.54112E-4 3.87483E-4 4.0654E-4 4.01839E-4 -5.39523E-4 -4.89014E-4 -4.07943E-4 -3.54024E-4

680 4.51509E-4 3.78064E-4 4.04874E-4 4.01006E-4 -5.38113E-4 -4.86054E-4 -4.08389E-4 -3.53693E-4

681 4.44816E-4 3.7156E-4 4.03393E-4 3.97025E-4 -5.37843E-4 -4.81433E-4 -4.07347E-4 -3.54222E-4

682 4.37193E-4 3.64242E-4 3.9932E-4 3.92397E-4 -5.3489E-4 -4.77101E-4 -4.07719E-4 -3.54288E-4

683 4.30562E-4 3.55027E-4 3.98487E-4 3.88509E-4 -5.29987E-4 -4.72192E-4 -4.07719E-4 -3.5356E-4

684 4.22134E-4 3.45337E-4 3.95525E-4 3.8601E-4 -5.23206E-4 -4.67498E-4 -4.07497E-4 -3.53825E-4

685 4.12281E-4 3.36596E-4 3.92748E-4 3.78975E-4 -5.15014E-4 -4.62084E-4 -4.06828E-4 -3.53361E-4

686 4.02985E-4 3.28669E-4 3.86453E-4 3.73606E-4 -5.05748E-4 -4.57751E-4 -4.06011E-4 -3.51773E-4

687 3.92945E-4 3.21419E-4 3.83861E-4 3.687E-4 -4.96145E-4 -4.51831E-4 -4.04524E-4 -3.51112E-4

688 3.82968E-4 3.13897E-4 3.81177E-4 3.59999E-4 -4.86744E-4 -4.47498E-4 -4.03558E-4 -3.49324E-4

689 3.74477E-4 3.06038E-4 3.76179E-4 3.55649E-4 -4.76539E-4 -4.43095E-4 -4.03558E-4 -3.48398E-4

690 3.64437E-4 2.98788E-4 3.7118E-4 3.48521E-4 -4.67608E-4 -4.39557E-4 -4.02741E-4 -3.47273E-4

691 3.56195E-4 2.91131E-4 3.672E-4 3.42504E-4 -4.59215E-4 -4.36164E-4 -4.03856E-4 -3.4436E-4

692 3.47767E-4 2.84355E-4 3.63682E-4 3.35655E-4 -4.51895E-4 -4.31614E-4 -4.04896E-4 -3.41647E-4

693 3.38471E-4 2.77173E-4 3.56555E-4 3.27416E-4 -4.4498E-4 -4.26488E-4 -4.03707E-4 -3.40059E-4

694 3.29052E-4 2.69787E-4 3.53593E-4 3.19548E-4 -4.38601E-4 -4.21507E-4 -4.01701E-4 -3.36749E-4

695 3.20809E-4 2.62063E-4 3.45169E-4 3.12791E-4 -4.32759E-4 -4.1537E-4 -3.97688E-4 -3.34896E-4

696 3.12753E-4 2.55219E-4 3.38782E-4 3.02887E-4 -4.25977E-4 -4.08727E-4 -3.9271E-4 -3.32513E-4

697 3.04821E-4 2.46411E-4 3.36838E-4 2.98073E-4 -4.198E-4 -4.02951E-4 -3.86988E-4 -3.3066E-4

698 2.95463E-4 2.38348E-4 3.28322E-4 2.90946E-4 -4.13354E-4 -3.96236E-4 -3.81267E-4 -3.2894E-4

699 2.8722E-4 2.31233E-4 3.21565E-4 2.83911E-4 -4.06707E-4 -3.89161E-4 -3.74355E-4 -3.28873E-4

700 2.78668E-4 2.23441E-4 3.15178E-4 2.79282E-4 -4.012E-4 -3.83096E-4 -3.67446E-4 -3.29006E-4

701 2.70239E-4 2.16462E-4 3.06755E-4 2.70859E-4 -3.95695E-4 -3.75731E-4 -3.6165E-4 -3.28278E-4

702 2.62927E-4 2.07585E-4 2.95369E-4 2.64102E-4 -3.90591E-4 -3.68005E-4 -3.54888E-4 -3.26557E-4

703 2.55118E-4 2.00878E-4 2.91389E-4 2.54568E-4 -3.85957E-4 -3.61724E-4 -3.48571E-4 -3.22917E-4

704 2.46009E-4 1.9234E-4 2.81577E-4 2.49662E-4 -3.8213E-4 -3.54721E-4 -3.42553E-4 -3.19012E-4

705 2.37518E-4 1.8326E-4 2.77134E-4 2.39665E-4 -3.77497E-4 -3.48078E-4 -3.35717E-4 -3.1385E-4

706 2.29462E-4 1.73978E-4 2.70932E-4 2.34481E-4 -3.73133E-4 -3.41725E-4 -3.29772E-4 -3.07959E-4

707 2.20228E-4 1.66118E-4 2.65656E-4 2.2652E-4 -3.69373E-4 -3.33566E-4 -3.24051E-4 -3.02069E-4

708 2.1149E-4 1.57242E-4 2.61953E-4 2.1856E-4 -3.64605E-4 -3.2714E-4 -3.18551E-4 -2.96906E-4

709 2.02689E-4 1.49179E-4 2.53159E-4 2.14302E-4 -3.58294E-4 -3.20497E-4 -3.13276E-4 -2.91082E-4

710 1.93642E-4 1.422E-4 2.50012E-4 2.06156E-4 -3.5225E-4 -3.13638E-4 -3.08446E-4 -2.85457E-4

711 1.8509E-4 1.32307E-4 2.47606E-4 1.98566E-4 -3.45602E-4 -3.07356E-4 -3.0265E-4 -2.79367E-4

712 1.76786E-4 1.24244E-4 2.38442E-4 1.88661E-4 -3.39156E-4 -3.00931E-4 -2.98712E-4 -2.73411E-4

713 1.67675E-4 1.1469E-4 2.34276E-4 1.7922E-4 -3.33113E-4 -2.93927E-4 -2.92841E-4 -2.68117E-4

714 1.59123E-4 1.03171E-4 2.27889E-4 1.67464E-4 -3.26533E-4 -2.87068E-4 -2.87863E-4 -2.62359E-4

715 1.4958E-4 9.3075E-5 2.18818E-4 1.5867E-4 -3.19752E-4 -2.80643E-4 -2.83033E-4 -2.57064E-4

716 1.40098E-4 8.35212E-5 2.09284E-4 1.51543E-4 -3.12767E-4 -2.74217E-4 -2.77832E-4 -2.51835E-4

717 1.30988E-4 7.40351E-5 2.02897E-4 1.43304E-4 -3.04308E-4 -2.68368E-4 -2.73001E-4 -2.46938E-4

718 1.22559E-4 6.59721E-5 1.92344E-4 1.35436E-4 -2.95645E-4 -2.62592E-4 -2.6847E-4 -2.40981E-4

719 1.13511E-4 5.82478E-5 1.84013E-4 1.28124E-4 -2.88662E-4 -2.57322E-4 -2.63565E-4 -2.36414E-4

720 1.0341E-4 5.03879E-5 1.79015E-4 1.21922E-4 -2.80202E-4 -2.51329E-4 -2.57992E-4 -2.30723E-4

721 9.45484E-5 4.29346E-5 1.68092E-4 1.13313E-4 -2.71606E-4 -2.45264E-4 -2.53756E-4 -2.2523E-4

722 8.63057E-5 3.70396E-5 1.60131E-4 1.07296E-4 -2.63482E-4 -2.38477E-4 -2.47811E-4 -2.20133E-4

723 7.76301E-5 3.01284E-5 1.51801E-4 9.89655E-5 -2.55693E-4 -2.3234E-4 -2.42833E-4 -2.15367E-4

724 6.93251E-5 2.34881E-5 1.45691E-4 9.1838E-5 -2.4777E-4 -2.26348E-4 -2.37335E-4 -2.10999E-4

725 6.18886E-5 1.66446E-5 1.36712E-4 8.67469E-5 -2.40585E-4 -2.21005E-4 -2.3139E-4 -2.07624E-4

726 5.48857E-5 9.73328E-6 1.2977E-4 7.84161E-5 -2.34005E-4 -2.16457E-4 -2.26039E-4 -2.03653E-4

727 4.7635E-5 -2.38201E-8 1.29215E-4 7.50837E-5 -2.28096E-4 -2.10464E-4 -2.21061E-4 -2.00476E-4

728 3.88349E-5 -7.00286E-6 1.25605E-4 6.4161E-5 -2.20911E-4 -2.06203E-4 -2.14894E-4 -1.97035E-4

729 2.94151E-5 -1.47272E-5 1.17089E-4 5.81443E-5 -2.1413E-4 -2.02016E-4 -2.09916E-4 -1.93196E-4

730 2.2846E-5 -2.31292E-5 1.07647E-4 4.83324E-5 -2.06408E-4 -1.97756E-4 -2.04639E-4 -1.89688E-4

731 1.45417E-5 -3.05148E-5 9.7835E-5 3.97238E-5 -1.98954E-4 -1.93207E-4 -1.9929E-4 -1.86115E-4

732 6.17545E-6 -3.97976E-5 8.73752E-5 3.18558E-5 -1.91837E-4 -1.89525E-4 -1.93939E-4 -1.81746E-4

733 -2.74857E-6 -4.69121E-5 7.71004E-5 2.27844E-5 -1.86129E-4 -1.86854E-4 -1.88068E-4 -1.7804E-4

734 -1.06191E-5 -5.57884E-5 6.90473E-5 1.70454E-5 -1.79683E-4 -1.82738E-4 -1.81903E-4 -1.73672E-4

735 -1.91713E-5 -6.50034E-5 5.97908E-5 9.36246E-6 -1.7411E-4 -1.79417E-4 -1.76106E-4 -1.69304E-4

736 -2.90248E-5 -7.26602E-5 5.1645E-5 2.69777E-6 -1.69208E-4 -1.75807E-4 -1.70978E-4 -1.65134E-4

737 -3.68334E-5 -8.18077E-5 4.25737E-5 -5.0777E-6 -1.63903E-4 -1.72197E-4 -1.65406E-4 -1.60832E-4

738 -4.36504E-5 -8.79059E-5 3.48908E-5 -1.37788E-5 -1.58532E-4 -1.68948E-4 -1.60502E-4 -1.56199E-4

739 -5.30701E-5 -9.6782E-5 2.72078E-5 -2.34982E-5 -1.53764E-4 -1.65554E-4 -1.56192E-4 -1.51897E-4

740 -6.25519E-5 -1.06064E-4 2.02655E-5 -2.84041E-5 -1.48795E-4 -1.61872E-4 -1.51139E-4 -1.4839E-4

741 -7.15384E-5 -1.13112E-4 1.48041E-5 -3.71052E-5 -1.43625E-4 -1.59128E-4 -1.47126E-4 -1.43293E-4

742 -8.02757E-5 -1.21919E-4 8.87994E-6 -4.33071E-5 -1.41074E-4 -1.54725E-4 -1.43337E-4 -1.39454E-4

743 -8.9076E-5 -1.30728E-4 5.49076E-7 -4.96941E-5 -1.37783E-4 -1.52125E-4 -1.39695E-4 -1.35219E-4

744 -9.81864E-5 -1.36827E-4 -6.11562E-6 -5.60811E-5 -1.33419E-4 -1.48442E-4 -1.36871E-4 -1.30917E-4

745 -1.0711E-4 -1.43941E-4 -1.60201E-5 -5.91358E-5 -1.31403E-4 -1.45699E-4 -1.33751E-4 -1.27012E-4

746 -1.13742E-4 -1.50378E-4 -1.76863E-5 -6.63558E-5 -1.29323E-4 -1.42306E-4 -1.30779E-4 -1.23173E-4

747 -1.21488E-4 -1.55257E-4 -2.56469E-5 -7.23726E-5 -1.27845E-4 -1.39346E-4 -1.28995E-4 -1.20261E-4

748 -1.28862E-4 -1.62642E-4 -3.06454E-5 -7.38536E-5 -1.27778E-4 -1.3588E-4 -1.25949E-4 -1.17482E-4

749 -1.3506E-4 -1.67724E-4 -3.67547E-5 -7.90373E-5 -1.28114E-4 -1.32126E-4 -1.23794E-4 -1.14966E-4

750 -1.41505E-4 -1.72873E-4 -4.55484E-5 -8.59797E-5 -1.29323E-4 -1.29382E-4 -1.21044E-4 -1.12518E-4

751 -1.48012E-4 -1.77888E-4 -5.20279E-5 -9.03302E-5 -1.31136E-4 -1.25411E-4 -1.18815E-4 -1.104E-4

752 -1.53713E-4 -1.80666E-4 -5.99885E-5 -9.68098E-5 -1.32545E-4 -1.22162E-4 -1.15546E-4 -1.07554E-4

753 -1.58981E-4 -1.8385E-4 -6.35986E-5 -1.00698E-4 -1.33553E-4 -1.18479E-4 -1.13391E-4 -1.05238E-4

754 -1.62018E-4 -1.88526E-4 -6.89674E-5 -1.01531E-4 -1.34426E-4 -1.15159E-4 -1.10047E-4 -1.03782E-4

755 -1.68648E-4 -1.92049E-4 -7.03558E-5 -1.06251E-4 -1.34695E-4 -1.1227E-4 -1.07743E-4 -1.01067E-4

756 -1.71499E-4 -1.91642E-4 -7.60949E-5 -1.1199E-4 -1.33553E-4 -1.0931E-4 -1.05515E-4 -9.86187E-5

757 -1.77635E-4 -1.94624E-4 -7.64651E-5 -1.16341E-4 -1.3315E-4 -1.07361E-4 -1.03656E-4 -9.74272E-5

758 -1.81229E-4 -1.9869E-4 -8.20191E-5 -1.19488E-4 -1.32345E-4 -1.05556E-4 -1.02245E-4 -9.72296E-5

759 -1.85877E-4 -2.03297E-4 -8.60919E-5 -1.2569E-4 -1.32143E-4 -1.031E-4 -1.0113E-4 -9.6501E-5

760 -1.89223E-4 -2.05194E-4 -8.79432E-5 -1.31151E-4 -1.31069E-4 -1.00862E-4 -9.94955E-5 -9.53758E-5

761 -1.91082E-4 -2.06684E-4 -9.04425E-5 -1.32077E-4 -1.29994E-4 -9.94188E-5 -9.77119E-5 -9.37872E-5

762 -1.90649E-4 -2.0682E-4 -9.1183E-5 -1.34484E-4 -1.28585E-4 -9.8191E-5 -9.67467E-5 -9.19341E-5

763 -1.91827E-4 -2.0682E-4 -9.28492E-5 -1.39205E-4 -1.27778E-4 -9.56638E-5 -9.54824E-5 -9.03461E-5

764 -1.935E-4 -2.05601E-4 -9.50707E-5 -1.42074E-4 -1.26301E-4 -9.56638E-5 -9.40713E-5 -8.75004E-5

765 -1.94181E-4 -2.05261E-4 -9.77551E-5 -1.41982E-4 -1.25294E-4 -9.43644E-5 -9.36254E-5 -8.58449E-5

766 -1.94925E-4 -2.02348E-4 -9.9051E-5 -1.43925E-4 -1.23883E-4 -9.36423E-5 -9.13959E-5 -8.4125E-5

767 -1.93686E-4 -2.01332E-4 -1.02106E-4 -1.45036E-4 -1.22541E-4 -9.32815E-5 -9.0727E-5 -8.26027E-5

768 -1.92137E-4 -2.00586E-4 -1.0155E-4 -1.46517E-4 -1.21064E-4 -9.24151E-5 -8.92411E-5 -8.20729E-5

769 -1.91454E-4 -1.98215E-4 -1.03772E-4 -1.45314E-4 -1.17606E-4 -9.10793E-5 -8.77552E-5 -8.00872E-5

770 -1.90835E-4 -1.97741E-4 -1.02939E-4 -1.44481E-4 -1.14752E-4 -9.0105E-5 -8.67893E-5 -7.81014E-5

771 -1.89595E-4 -1.97877E-4 -1.05531E-4 -1.44296E-4 -1.1079E-4 -8.82999E-5 -8.53028E-5 -7.66461E-5

772 -1.84328E-4 -1.94895E-4 -1.06919E-4 -1.46054E-4 -1.0703E-4 -8.72164E-5 -8.37428E-5 -7.49912E-5

773 -1.81043E-4 -1.91507E-4 -1.07474E-4 -1.45221E-4 -1.02196E-4 -8.64227E-5 -8.28503E-5 -7.3866E-5

774 -1.77758E-4 -1.88593E-4 -1.10714E-4 -1.45129E-4 -9.74947E-5 -8.48335E-5 -8.16615E-5 -7.3138E-5

775 -1.72243E-4 -1.87171E-4 -1.12843E-4 -1.43092E-4 -9.1586E-5 -8.33183E-5 -7.96556E-5 -7.35352E-5

776 -1.7088E-4 -1.85409E-4 -1.1488E-4 -1.40038E-4 -8.49383E-5 -8.11519E-5 -7.80208E-5 -7.30717E-5

777 -1.66665E-4 -1.80937E-4 -1.17379E-4 -1.3615E-4 -7.83584E-5 -7.92019E-5 -7.64608E-5 -7.29391E-5

778 -1.62141E-4 -1.77414E-4 -1.19878E-4 -1.3254E-4 -7.23823E-5 -7.60975E-5 -7.49743E-5 -7.28072E-5

779 -1.59787E-4 -1.75245E-4 -1.20156E-4 -1.31244E-4 -6.60036E-5 -7.33538E-5 -7.28195E-5 -7.20129E-5

780 -1.57741E-4 -1.70773E-4 -1.19971E-4 -1.29948E-4 -6.04302E-5 -6.9888E-5 -7.111E-5 -7.20792E-5

781 -1.51978E-4 -1.66573E-4 -1.19045E-4 -1.23931E-4 -5.45213E-5 -6.7145E-5 -6.98471E-5 -7.10203E-5

782 -1.51297E-4 -1.63658E-4 -1.21822E-4 -1.2134E-4 -4.8881E-5 -6.39679E-5 -6.89553E-5 -6.91002E-5

783 -1.46958E-4 -1.60271E-4 -1.26543E-4 -1.16804E-4 -4.37107E-5 -5.97802E-5 -6.75435E-5 -6.83729E-5

784 -1.42186E-4 -1.57493E-4 -1.24877E-4 -1.16156E-4 -3.90776E-5 -5.65312E-5 -6.53887E-5 -6.6718E-5

785 -1.41195E-4 -1.54105E-4 -1.28302E-4 -1.16248E-4 -3.59888E-5 -5.59536E-5 -6.43484E-5 -6.55272E-5

786 -1.38158E-4 -1.53223E-4 -1.28024E-4 -1.1162E-4 -3.53845E-5 -5.37154E-5 -6.18219E-5 -6.32103E-5

787 -1.34874E-4 -1.50716E-4 -1.30246E-4 -1.09584E-4 -3.36387E-5 -5.24879E-5 -5.95928E-5 -6.20191E-5

788 -1.31403E-4 -1.4611E-4 -1.30893E-4 -1.05326E-4 -3.17585E-5 -5.27768E-5 -5.81064E-5 -5.9769E-5

789 -1.31589E-4 -1.36041E-4 -1.27098E-4 -1.01993E-4 -3.07513E-5 -5.30656E-5 -5.49115E-5 -5.79157E-5

790 -1.29792E-4 -1.35041E-4 -1.25895E-4 -1.0116E-4 -2.99455E-5 -5.27045E-5 -5.33508E-5 -5.54668E-5

791 -1.26445E-4 -1.30041E-4 -1.2608E-4 -9.90313E-5 -2.94084E-5 -5.25602E-5 -5.2014E-5 -5.34813E-5

792 -1.24028E-4 -1.29041E-4 -1.21637E-4 -9.90313E-5 -3.12213E-5 -4.93111E-5 -4.98584E-5 -5.1628E-5

793 -1.21611E-4 -1.28041E-4 -1.21082E-4 -9.90313E-5 -3.22286E-5 -4.61343E-5 -4.96361E-5 -4.95767E-5

794 -1.21053E-4 -1.27041E-4 -1.20248E-4 -9.90313E-5 -3.53845E-5 -4.43293E-5 -4.99327E-5 -4.79879E-5

795 -1.20125E-4 -1.26041E-4 -1.20063E-4 -9.90313E-5 -3.87418E-5 -4.21632E-5 -4.90413E-5 -4.61347E-5

796 -1.16406E-4 -1.25041E-4 -1.20156E-4 -9.90313E-5 -4.25019E-5 -4.1008E-5 -4.88925E-5 -4.44144E-5

797 -1.12687E-4 -1.24041E-4 -1.14695E-4 -9.90313E-5 -4.72694E-5 -3.97807E-5 -4.88925E-5 -4.34877E-5

798 -1.11324E-4 -1.22304E-4 -1.13861E-4 -9.90313E-5 -5.29769E-5 -3.86253E-5 -4.71088E-5 -4.42156E-5

799 -1.11138E-4 -1.23041E-4 -1.09881E-4 -9.90313E-5 -5.67371E-5 -3.94918E-5 -4.61429E-5 -4.32225E-5

800 -1.11138E-4 -1.23041E-4 -1.06364E-4 -9.90313E-5 -6.07662E-5 -4.12246E-5 -4.52514E-5 -4.29579E-5

801 -1.09775E-4 -1.22041E-4 -1.05716E-4 -9.90313E-5 -6.35191E-5 -4.29574E-5 -4.436E-5 -4.27591E-5

802 -1.07482E-4 -1.21041E-4 -1.05345E-4 -9.90313E-5 -6.57345E-5 -4.5701E-5 -4.41369E-5 -4.26269E-5

803 -1.06243E-4 -1.20041E-4 -1.03494E-4 -1.06243E-4 -6.87565E-5 -4.72173E-5 -4.3989E-5 -4.223E-5

804 -1.05189E-4 -1.19041E-4 -1.01273E-4 -1.05189E-4 -7.1241E-5 -4.90223E-5 -4.38394E-5 -4.30902E-5

805 -1.03206E-4 -1.18041E-4 -9.71997E-5 -1.03206E-4 -7.34571E-5 -5.11161E-5 -4.45087E-5 -4.35535E-5

806 -1.03454E-4 -1.17041E-4 -9.49782E-5 -1.03454E-4 -7.51357E-5 -5.19103E-5 -4.47309E-5 -4.42156E-5

807 -1.02152E-4 -1.16041E-4 -9.34046E-5 -1.02152E-4 -7.66126E-5 -5.19826E-5 -4.47309E-5 -4.5341E-5

808 -1.01533E-4 -1.15041E-4 -9.01648E-5 -1.01533E-4 -7.62765E-5 -5.37154E-5 -4.59198E-5 -4.62669E-5

809 -1.01161E-4 -1.14041E-4 -9.14607E-5 -1.01161E-4 -7.621E-5 -5.36432E-5 -4.5995E-5 -4.81202E-5

810 -9.97354E-5 -1.13041E-4 -8.85912E-5 -9.97354E-5 -7.5068E-5 -5.34266E-5 -4.65147E-5 -4.78557E-5

811 -9.91776E-5 -1.12041E-4 -8.46109E-5 -9.91776E-5 -7.3121E-5 -5.37876E-5 -4.7184E-5 -4.87823E-5

812 -9.62033E-5 -1.11041E-4 -7.86867E-5 -9.62033E-5 -7.14422E-5 -5.34266E-5 -4.68865E-5 -4.97747E-5

813 -9.37241E-5 -1.04041E-4 -7.81313E-5 -9.37241E-5 -6.87565E-5 -5.40764E-5 -4.7778E-5 -4.95102E-5

814 -9.1741E-5 -1.01041E-4 -7.51692E-5 -9.1741E-5 -6.51977E-5 -5.47984E-5 -4.82977E-5 -4.91791E-5

815 -9.11833E-5 -9.93934E-5 -7.58172E-5 -9.11833E-5 -6.10346E-5 -5.47984E-5 -4.84464E-5 -4.99077E-5

816 -8.77123E-5 -9.83934E-5 -7.34105E-5 -8.77123E-5 -5.73414E-5 -5.45817E-5 -4.80754E-5 -4.92456E-5

817 -8.44279E-5 -9.73934E-5 -7.11889E-5 -8.44279E-5 -5.27083E-5 -5.30656E-5 -4.87438E-5 -4.85178E-5

818 -8.1887E-5 -9.63934E-5 -6.82268E-5 -8.1887E-5 -4.76723E-5 -5.16216E-5 -4.82977E-5 -4.84513E-5

819 -8.21971E-5 -9.53934E-5 -6.65607E-5 -8.21971E-5 -4.30391E-5 -4.95277E-5 -4.80754E-5 -4.87823E-5

820 -7.80449E-5 -9.44934E-5 -6.44317E-5 -7.80449E-5 -3.78017E-5 -4.77227E-5 -4.82977E-5 -4.75911E-5

821 -7.56281E-5 -9.33934E-5 -6.19324E-5 -7.56281E-5 -3.28328E-5 -4.53401E-5 -4.78524E-5 -4.72601E-5

822 -7.23437E-5 -9.33934E-5 -6.05439E-5 -7.23437E-5 -2.79983E-5 -4.2813E-5 -4.75549E-5 -4.6598E-5

823 -7.18478E-5 -9.33934E-5 -6.12845E-5 -7.18478E-5 -2.4171E-5 -3.9564E-5 -4.65147E-5 -4.65322E-5

824 -7.06082E-5 -8.79059E-5 -6.11919E-5 -7.06082E-5 -2.10151E-5 -3.6676E-5 -4.52514E-5 -4.62012E-5

825 -6.88727E-5 -8.07237E-5 -5.82298E-5 -6.88727E-5 -1.81277E-5 -3.41489E-5 -4.3989E-5 -4.52745E-5

826 -6.63943E-5 -7.75383E-5 -5.66562E-5 -6.63943E-5 -1.60462E-5 -3.31382E-5 -4.28001E-5 -4.48112E-5

827 -6.52164E-5 -7.15762E-5 -5.46198E-5 -6.52164E-5 -1.60462E-5 -3.27049E-5 -4.1165E-5 -4.42156E-5

828 -6.34195E-5 -6.6562E-5 -5.32313E-5 -6.34195E-5 -1.47704E-5 -3.25606E-5 -4.04214E-5 -4.28921E-5

829 -6.19322E-5 -6.4258E-5 -5.24908E-5 -6.19322E-5 -1.22188E-5 -3.28493E-5 -3.96787E-5 -4.1899E-5

830 -6.2366E-5 -6.07347E-5 -5.0732E-5 -6.2366E-5 -1.14802E-5 -3.28493E-5 -3.85641E-5 -4.13699E-5

831 -6.16843E-5 -5.98539E-5 -4.97138E-5 -6.16843E-5 -1.00701E-5 -3.27772E-5 -3.84898E-5 -4.0509E-5

832 -6.09406E-5 -5.82954E-5 -5.01766E-5 -6.09406E-5 -1.08087E-5 -3.16219E-5 -3.67812E-5 -3.95166E-5

833 -6.05068E-5 -5.6737E-5 -4.9251E-5 -6.05068E-5 -1.13459E-5 -2.98169E-5 -3.49974E-5 -3.78621E-5

834 -5.87716E-5 -5.65338E-5 -4.84179E-5 -5.72845E-5 -1.14131E-5 -2.89505E-5 -3.4105E-5 -3.61411E-5

835 -5.68504E-5 -5.68725E-5 -4.66592E-5 -5.72845E-5 -1.20174E-5 -2.78675E-5 -3.40315E-5 -3.50822E-5

836 -5.58589E-5 -5.78889E-5 -4.72145E-5 -5.72845E-5 -1.26888E-5 -2.66401E-5 -3.30657E-5 -3.39568E-5

837 -5.45575E-5 -5.16552E-5 -4.62889E-5 -5.72845E-5 -1.28231E-5 -2.54849E-5 -3.24708E-5 -3.23688E-5

838 -5.36279E-5 -5.15874E-5 -4.61038E-5 -5.72845E-5 -1.34946E-5 -2.47628E-5 -3.26939E-5 -3.17067E-5

839 -5.36279E-5 -4.92159E-5 -4.57335E-5 -5.72845E-5 -1.55762E-5 -2.25246E-5 -3.22477E-5 -3.09788E-5

840 -5.28842E-5 -4.7251E-5 -4.10127E-5 -5.72845E-5 -1.6382E-5 -2.11528E-5 -3.1505E-5 -3.0449E-5

841 -5.44335E-5 -4.58958E-5 -4.18458E-5 -5.72845E-5 -1.78591E-5 -1.94922E-5 -3.1505E-5 -3.07143E-5

842 -5.31321E-5 -4.65055E-5 -4.49004E-5 -5.72845E-5 -1.96721E-5 -1.76872E-5 -3.0687E-5 -3.09788E-5

843 -5.16448E-5 -4.37953E-5 -4.60112E-5 -5.72845E-5 -2.09479E-5 -1.63153E-5 -3.01673E-5 -3.10446E-5

844 -5.28842E-5 -4.27789E-5 -4.60112E-5 -5.72845E-5 -2.32981E-5 -1.45825E-5 -3.05383E-5 -3.11776E-5

845 -5.20166E-5 -4.21013E-5 -4.60112E-5 -5.72845E-5 -2.49766E-5 -1.31385E-5 -2.98699E-5 -3.14421E-5

846 -5.22025E-5 -3.95943E-5 -4.7122E-5 -5.72845E-5 -2.58496E-5 -1.32829E-5 -3.0093E-5 -3.09788E-5

847 -5.21405E-5 -3.69518E-5 -4.85105E-5 -5.72845E-5 -2.73268E-5 -1.23443E-5 -2.94246E-5 -3.217E-5

848 -5.23884E-5 -3.50545E-5 -4.83253E-5 -5.70068E-5 -2.76626E-5 -1.16223E-5 -2.89784E-5 -3.22365E-5

849 -5.20786E-5 -3.51223E-5 -4.87882E-5 -5.8025E-5 -2.75954E-5 -1.10447E-5 -2.94246E-5 -3.13099E-5

850 -5.18927E-5 -3.54611E-5 -4.84179E-5 -4.90462E-5 -2.6924E-5 -9.23962E-6 -2.80126E-5 -3.09788E-5

851 -5.09011E-5 -3.28185E-5 -4.67517E-5 -5.01569E-5 -2.63197E-5 -8.80646E-6 -2.74921E-5 -2.94566E-5

852 -4.86701E-5 -2.87531E-5 -4.55484E-5 -4.89536E-5 -2.52453E-5 -7.50685E-6 -2.74186E-5 -2.75375E-5

853 -4.69968E-5 -2.90919E-5 -4.49004E-5 -4.96941E-5 -2.53124E-5 -5.34082E-6 -2.74186E-5 -2.68754E-5

854 -4.47658E-5 -2.99727E-5 -4.16606E-5 -4.51584E-5 -2.45067E-5 -4.691E-6 -2.69724E-5 -2.60153E-5

855 -4.31545E-5 -2.96339E-5 -4.02722E-5 -4.76577E-5 -2.26265E-5 -3.10259E-6 -2.63784E-5 -2.4162E-5

856 -4.17292E-5 -2.94984E-5 -4.55484E-5 -5.06198E-5 -2.19551E-5 -1.65858E-6 -2.49655E-5 -2.40297E-5

857 -4.23489E-5 -2.9363E-5 -4.80476E-5 -5.41372E-5 -2.04107E-5 -1.29757E-6 -2.40005E-5 -2.2441E-5

858 -3.88164E-5 -2.86853E-5 -4.88807E-5 -5.67291E-5 -1.84635E-5 -1.65858E-6 -2.31826E-5 -2.13156E-5

859 -3.70193E-5 -2.84143E-5 -4.98989E-5 -5.66365E-5 -1.6382E-5 -5.75562E-7 -2.16219E-5 -2.07865E-5

860 -3.49742E-5 -2.78722E-5 -4.94361E-5 -5.74696E-5 -1.37632E-5 -9.36572E-7 -2.02107E-5 -1.9463E-5

861 -3.3177E-5 -2.78045E-5 -5.0732E-5 -5.66365E-5 -1.22188E-5 -4.3116E-7 -1.93184E-5 -1.80066E-5

862 -3.28052E-5 -2.794E-5 -5.00841E-5 -5.63588E-5 -1.12788E-5 -7.01558E-8 -1.82782E-5 -1.70799E-5

863 -3.23714E-5 -2.65171E-5 -4.93435E-5 -5.76547E-5 -8.19001E-6 2.90848E-7 -1.68662E-5 -1.66831E-5

864 -3.23094E-5 -2.5975E-5 -5.37867E-5 -5.90432E-5 -5.97416E-6 5.07451E-7 -1.53807E-5 -1.4631E-5

865 -3.37347E-5 -2.69914E-5 -5.21205E-5 -6.14499E-5 -4.36263E-6 1.59047E-6 -1.34482E-5 -1.49621E-5

866 -3.36108E-5 -2.48909E-5 -5.53603E-5 -6.27458E-5 -1.20674E-6 2.16808E-6 -1.25567E-5 -1.35721E-5

867 -3.28052E-5 -2.65848E-5 -5.43421E-5 -6.38566E-5 2.70489E-7 2.16808E-6 -1.16652E-5 -1.27778E-5

868 -3.33009E-5 -2.63138E-5 -5.54528E-5 -6.50599E-5 2.2849E-6 4.1175E-6 -1.06985E-5 -1.21164E-5

869 -3.46644E-5 -2.71269E-5 -5.18428E-5 -6.51525E-5 4.09786E-6 4.6951E-6 -1.04763E-5 -1.11898E-5

870 -3.44165E-5 -2.66526E-5 -5.67488E-5 -6.51525E-5 5.84367E-6 4.7673E-6 -1.0625E-5 -1.1322E-5

871 -3.42306E-5 -2.67882E-5 -5.61934E-5 -6.55228E-5 6.75015E-6 5.45321E-6 -9.88143E-6 -1.11898E-5

872 -3.21235E-5 -2.68559E-5 -5.89703E-5 -6.47822E-5 7.45524E-6 5.92251E-6 -9.95579E-6 -1.03954E-5

873 -3.27432E-5 -2.51619E-5 -6.06365E-5 -6.59856E-5 7.1866E-6 6.42792E-6 -1.01045E-5 -1.05277E-5

874 -3.20615E-5 -2.51619E-5 -5.91555E-5 -6.54302E-5 7.25376E-6 6.42792E-6 -9.2874E-6 -1.06599E-5

875 -3.17517E-5 -2.40778E-5 -5.86926E-5 -6.39491E-5 8.79813E-6 6.93336E-6 -8.32072E-6 -9.46875E-6

876 -3.3425E-5 -2.46198E-5 -5.80447E-5 -6.41343E-5 1.00068E-5 6.78893E-6 -7.94892E-6 -8.40983E-6

877 -3.03883E-5 -2.50264E-5 -5.99885E-5 -6.3764E-5 1.00068E-5 8.01639E-6 -7.20701E-6 -7.48319E-6

878 -3.11319E-5 -2.42133E-5 -6.09142E-5 -6.33938E-5 1.12154E-5 8.88271E-6 -6.31469E-6 -6.42428E-6

879 -3.23851E-5 -2.25595E-5 -6.05714E-5 -6.28795E-5 1.05291E-5 8.64741E-6 -5.50323E-6 -5.98598E-6

**Fig. 5 Stress equilibrium curves of granite samples**

1 -2.10186E-5 8.74677E-6 -1.30917E-5 -1.56348E-5

2 -2.39807E-5 1.01306E-5 -1.26888E-5 -1.76871E-5

3 -2.85164E-5 1.10626E-5 -1.26888E-5 -2.20164E-5

4 -2.97197E-5 1.23335E-5 -1.26217E-5 -2.21414E-5

5 -3.14785E-5 1.35284E-5 -1.39646E-5 -2.29866E-5

6 -3.54588E-5 1.47447E-5 -1.54419E-5 -2.63875E-5

7 -3.83283E-5 1.59498E-5 -1.68519E-5 -2.8511E-5

8 -4.27714E-5 1.6569E-5 -1.88664E-5 -3.30458E-5

9 -4.74922E-5 1.81768E-5 -2.08136E-5 -3.69142E-5

10 -4.98064E-5 1.89404E-5 -2.21566E-5 -3.8835E-5

11 -5.63785E-5 2.03446E-5 -2.41038E-5 -4.50545E-5

12 -6.37837E-5 2.14784E-5 -2.65211E-5 -5.25107E-5

13 -6.96153E-5 2.27049E-5 -2.9677E-5 -5.80489E-5

14 -7.75759E-5 2.41201E-5 -3.20272E-5 -6.58679E-5

15 -8.5444E-5 2.57465E-5 -3.5653E-5 -7.33686E-5

16 -9.34971E-5 2.73053E-5 -3.90776E-5 -8.11514E-5

17 -1.03309E-4 2.936E-5 -4.37778E-5 -9.04785E-5

18 -1.10529E-4 3.0989E-5 -4.89481E-5 -9.72247E-5

19 -1.19971E-4 3.22664E-5 -5.35812E-5 -1.069E-4

20 -1.29227E-4 3.48415E-5 -5.91544E-5 -1.15062E-4

21 -1.38206E-4 3.64041E-5 -6.58694E-5 -1.23915E-4

22 -1.48481E-4 4.06016E-5 -7.22481E-5 -1.31636E-4

23 -1.60885E-4 4.1408E-5 -8.02389E-5 -1.45219E-4

24 -1.7153E-4 4.30073E-5 -9.03111E-5 -1.55968E-4

25 -1.81619E-4 4.4814E-5 -9.96439E-5 -1.65864E-4

26 -1.92542E-4 4.68773E-5 -1.09716E-4 -1.76471E-4

27 -1.98651E-4 4.9383E-5 -1.19519E-4 -1.81052E-4

28 -2.09111E-4 5.23987E-5 -1.30867E-4 -1.9017E-4

29 -2.20219E-4 5.49404E-5 -1.43625E-4 -2.00514E-4

30 -2.32623E-4 5.91661E-5 -1.55712E-4 -2.10677E-4

31 -2.43638E-4 6.1646E-5 -1.68134E-4 -2.20974E-4

32 -2.57615E-4 6.53503E-5 -1.8257E-4 -2.33483E-4

33 -2.68723E-4 6.78384E-5 -1.9647E-4 -2.4388E-4

34 -2.80386E-4 7.04886E-5 -2.10638E-4 -2.54759E-4

35 -2.92512E-4 7.46935E-5 -2.26081E-4 -2.6462E-4

36 -3.02787E-4 7.78491E-5 -2.40183E-4 -2.73384E-4

37 -3.1371E-4 8.19209E-5 -2.55492E-4 -2.81983E-4

38 -3.25651E-4 8.53553E-5 -2.70801E-4 -2.924E-4

39 -3.33704E-4 9.02813E-5 -2.85371E-4 -2.96815E-4

40 -3.50631E-4 9.35229E-5 -3.00077E-4 -3.13209E-4

41 -3.64528E-4 9.6496E-5 -3.15185E-4 -3.26356E-4

42 -3.77394E-4 1.01683E-4 -3.30158E-4 -3.36094E-4

43 -3.90354E-4 1.06565E-4 -3.43923E-4 -3.46245E-4

44 -4.01739E-4 1.1038E-4 -3.57152E-4 -3.55637E-4

45 -4.15531E-4 1.15362E-4 -3.69306E-4 -3.66654E-4

46 -4.25621E-4 1.20299E-4 -3.79914E-4 -3.73422E-4

47 -4.38765E-4 1.24352E-4 -3.8945E-4 -3.84615E-4

48 -4.47929E-4 1.30372E-4 -3.98179E-4 -3.89226E-4

49 -4.61166E-4 1.36686E-4 -4.059E-4 -3.98267E-4

50 -4.73477E-4 1.40353E-4 -4.11877E-4 -4.08881E-4

51 -4.87362E-4 1.49674E-4 -4.16443E-4 -4.15666E-4

52 -4.99951E-4 1.55387E-4 -4.20136E-4 -4.24556E-4

53 -5.12817E-4 1.6132E-4 -4.21747E-4 -4.33547E-4

54 -5.22722E-4 1.66488E-4 -4.22419E-4 -4.39869E-4

55 -5.33922E-4 1.7393E-4 -4.22486E-4 -4.4542E-4

56 -5.479E-4 1.8128E-4 -4.2215E-4 -4.54284E-4

57 -5.61229E-4 1.85657E-4 -4.2121E-4 -4.65368E-4

58 -5.74373E-4 1.9275E-4 -4.22486E-4 -4.73523E-4

59 -5.85481E-4 1.99152E-4 -4.23024E-4 -4.80006E-4

60 -5.96589E-4 2.04188E-4 -4.22821E-4 -4.87855E-4

61 -6.09085E-4 2.10128E-4 -4.25239E-4 -4.96411E-4

62 -6.20563E-4 2.1635E-4 -4.27454E-4 -5.03503E-4

63 -6.28709E-4 2.2233E-4 -4.29804E-4 -5.06973E-4

64 -6.39632E-4 2.25958E-4 -4.34505E-4 -5.16015E-4

65 -6.49721E-4 2.35013E-4 -4.38332E-4 -5.18663E-4

66 -6.59533E-4 2.42354E-4 -4.41891E-4 -5.22704E-4

67 -6.70919E-4 2.46429E-4 -4.46726E-4 -5.31837E-4

68 -6.78879E-4 2.50884E-4 -4.51761E-4 -5.36616E-4

69 -6.9082E-4 2.55947E-4 -4.57133E-4 -5.45404E-4

70 -7.00262E-4 2.63031E-4 -4.63176E-4 -5.49273E-4

71 -7.1248E-4 2.68138E-4 -4.68279E-4 -5.58339E-4

72 -7.20348E-4 2.74188E-4 -4.73585E-4 -5.61415E-4

73 -7.28031E-4 2.80078E-4 -4.78688E-4 -5.64438E-4

74 -7.36732E-4 2.88104E-4 -4.83656E-4 -5.66505E-4

75 -7.44323E-4 2.96474E-4 -4.87283E-4 -5.66941E-4

76 -7.52006E-4 3.01498E-4 -4.91445E-4 -5.70829E-4

77 -7.58671E-4 3.10378E-4 -4.96145E-4 -5.6968E-4

78 -7.66724E-4 3.19867E-4 -5.0051E-4 -5.69533E-4

79 -7.7163E-4 3.25253E-4 -5.04875E-4 -5.69838E-4

80 -7.79498E-4 3.34717E-4 -5.0877E-4 -5.69501E-4

81 -7.85514E-4 3.41893E-4 -5.12664E-4 -5.69303E-4

82 -7.87551E-4 3.4864E-4 -5.15618E-4 -5.64919E-4

83 -7.90328E-4 3.52727E-4 -5.18506E-4 -5.64053E-4

84 -7.94123E-4 3.61553E-4 -5.20251E-4 -5.59629E-4

85 -7.96344E-4 3.68313E-4 -5.2193E-4 -5.55446E-4

86 -8.0125E-4 3.74957E-4 -5.23139E-4 -5.54493E-4

87 -8.00973E-4 3.80148E-4 -5.24011E-4 -5.48981E-4

88 -8.02546E-4 3.86263E-4 -5.24145E-4 -5.44691E-4

89 -8.01343E-4 3.90517E-4 -5.24549E-4 -5.39041E-4

90 -8.01065E-4 3.9496E-4 -5.25624E-4 -5.34275E-4

91 -8.0014E-4 3.98764E-4 -5.27302E-4 -5.29398E-4

92 -7.97177E-4 4.01819E-4 -5.27973E-4 -5.22906E-4

93 -7.9403E-4 4.04226E-4 -5.29383E-4 -5.16849E-4

94 -7.89495E-4 4.08021E-4 -5.31398E-4 -5.07793E-4

95 -7.86533E-4 4.07651E-4 -5.32204E-4 -5.04727E-4

96 -7.82182E-4 4.10243E-4 -5.34083E-4 -4.97088E-4

97 -7.78017E-4 4.10428E-4 -5.35628E-4 -4.92072E-4

98 -7.73666E-4 4.08947E-4 -5.36769E-4 -4.88506E-4

99 -7.72092E-4 4.06725E-4 -5.38113E-4 -4.88902E-4

100 -7.66353E-4 4.09409E-4 -5.39321E-4 -4.7956E-4

101 -7.60985E-4 4.05892E-4 -5.38649E-4 -4.76851E-4

102 -7.58208E-4 4.05985E-4 -5.39388E-4 -4.73536E-4

103 -7.53024E-4 4.0654E-4 -5.39523E-4 -4.66968E-4

104 -7.50062E-4 4.04874E-4 -5.38113E-4 -4.65198E-4

105 -7.44786E-4 4.03393E-4 -5.37843E-4 -4.60559E-4

106 -7.39417E-4 3.9932E-4 -5.3489E-4 -4.58404E-4

107 -7.34233E-4 3.98487E-4 -5.29987E-4 -4.53223E-4

108 -7.27383E-4 3.95525E-4 -5.23206E-4 -4.48239E-4

109 -7.25902E-4 3.92748E-4 -5.15014E-4 -4.49298E-4

110 -7.18312E-4 3.86453E-4 -5.05748E-4 -4.46789E-4

111 -7.08963E-4 3.83861E-4 -4.96145E-4 -4.38536E-4

112 -7.04983E-4 3.81177E-4 -4.86744E-4 -4.36603E-4

113 -6.97948E-4 3.76179E-4 -4.76539E-4 -4.33441E-4

114 -6.93504E-4 3.7118E-4 -4.67608E-4 -4.33285E-4

115 -6.85637E-4 3.672E-4 -4.59215E-4 -4.28139E-4

116 -6.81378E-4 3.63682E-4 -4.51895E-4 -4.26716E-4

117 -6.75269E-4 3.56555E-4 -4.4498E-4 -4.26757E-4

118 -6.69067E-4 3.53593E-4 -4.38601E-4 -4.22525E-4

119 -6.64439E-4 3.45169E-4 -4.32759E-4 -4.2558E-4

120 -6.56201E-4 3.38782E-4 -4.25977E-4 -4.22411E-4

121 -6.52868E-4 3.36838E-4 -4.198E-4 -4.20489E-4

122 -6.43149E-4 3.28322E-4 -4.13354E-4 -4.17731E-4

123 -6.36947E-4 3.21565E-4 -4.06707E-4 -4.17294E-4

124 -6.28339E-4 3.15178E-4 -4.012E-4 -4.13695E-4

125 -6.19175E-4 3.06755E-4 -3.95695E-4 -4.11488E-4

126 -6.12325E-4 2.95369E-4 -3.90591E-4 -4.14928E-4

127 -6.06586E-4 2.91389E-4 -3.85957E-4 -4.12251E-4

128 -6.0242E-4 2.81577E-4 -3.8213E-4 -4.1723E-4

129 -5.96311E-4 2.77134E-4 -3.77497E-4 -4.14587E-4

130 -5.89184E-4 2.70932E-4 -3.73133E-4 -4.12521E-4

131 -5.81686E-4 2.65656E-4 -3.69373E-4 -4.091E-4

132 -5.76965E-4 2.61953E-4 -3.64605E-4 -4.07326E-4

133 -5.70856E-4 2.53159E-4 -3.58294E-4 -4.09034E-4

134 -5.62247E-4 2.50012E-4 -3.5225E-4 -4.02195E-4

135 -5.51047E-4 2.47606E-4 -3.45602E-4 -3.91609E-4

136 -5.44845E-4 2.38442E-4 -3.39156E-4 -3.93578E-4

137 -5.33552E-4 2.34276E-4 -3.33113E-4 -3.84644E-4

138 -5.28553E-4 2.27889E-4 -3.26533E-4 -3.85232E-4

139 -5.23185E-4 2.18818E-4 -3.19752E-4 -3.88077E-4

140 -5.10318E-4 2.09284E-4 -3.12767E-4 -3.82685E-4

141 -5.0532E-4 2.02897E-4 -3.04308E-4 -3.83274E-4

142 -4.96248E-4 1.92344E-4 -2.95645E-4 -3.83304E-4

143 -4.87454E-4 1.84013E-4 -2.88662E-4 -3.81434E-4

144 -4.7718E-4 1.79015E-4 -2.80202E-4 -3.74514E-4

145 -4.66442E-4 1.68092E-4 -2.71606E-4 -3.72981E-4

146 -4.55705E-4 1.60131E-4 -2.63482E-4 -3.68487E-4

147 -4.46356E-4 1.51801E-4 -2.55693E-4 -3.65972E-4

148 -4.35248E-4 1.45691E-4 -2.4777E-4 -3.59197E-4

149 -4.25714E-4 1.36712E-4 -2.40585E-4 -3.57116E-4

150 -4.15439E-4 1.2977E-4 -2.34005E-4 -3.52139E-4

151 -4.03035E-4 1.29215E-4 -2.28096E-4 -3.38306E-4

152 -3.90909E-4 1.25605E-4 -2.20911E-4 -3.27849E-4

153 -3.82208E-4 1.17089E-4 -2.1413E-4 -3.26272E-4

154 -3.70822E-4 1.07647E-4 -2.06408E-4 -3.22507E-4

155 -3.62399E-4 9.7835E-5 -1.98954E-4 -3.22548E-4

156 -3.56105E-4 8.73752E-5 -1.91837E-4 -3.25707E-4

157 -3.46015E-4 7.71004E-5 -1.86129E-4 -3.24277E-4

158 -3.40831E-4 6.90473E-5 -1.79683E-4 -3.26317E-4

159 -3.30094E-4 5.97908E-5 -1.7411E-4 -3.23118E-4

160 -3.2417E-4 5.1645E-5 -1.69208E-4 -3.24392E-4

161 -3.15468E-4 4.25737E-5 -1.63903E-4 -3.23369E-4

162 -3.04083E-4 3.48908E-5 -1.58532E-4 -3.17845E-4

163 -2.99177E-4 2.72078E-5 -1.53764E-4 -3.19838E-4

164 -2.88254E-4 2.02655E-5 -1.48795E-4 -3.14109E-4

165 -2.79275E-4 1.48041E-5 -1.43625E-4 -3.09155E-4

166 -2.72518E-4 8.87994E-6 -1.41074E-4 -3.07241E-4

167 -2.66872E-4 5.49076E-7 -1.37783E-4 -3.09022E-4

168 -2.56597E-4 -6.11562E-6 -1.33419E-4 -3.03768E-4

169 -2.52802E-4 -1.60201E-5 -1.31403E-4 -3.0927E-4

170 -2.44656E-4 -1.76863E-5 -1.29323E-4 -3.01487E-4

171 -2.38454E-4 -2.56469E-5 -1.27845E-4 -3.02254E-4

172 -2.31049E-4 -3.06454E-5 -1.27778E-4 -2.98662E-4

173 -2.23181E-4 -3.67547E-5 -1.28114E-4 -2.95645E-4

174 -2.13924E-4 -4.55484E-5 -1.29323E-4 -2.937E-4

175 -2.05871E-4 -5.20279E-5 -1.31136E-4 -2.90838E-4

176 -1.95504E-4 -5.99885E-5 -1.32545E-4 -2.86773E-4

177 -1.8958E-4 -6.35986E-5 -1.33553E-4 -2.83511E-4

178 -1.80138E-4 -6.89674E-5 -1.34426E-4 -2.77927E-4

179 -1.73381E-4 -7.03558E-5 -1.34695E-4 -2.71478E-4

180 -1.6542E-4 -7.60949E-5 -1.33553E-4 -2.67982E-4

181 -1.57275E-4 -7.64651E-5 -1.3315E-4 -2.58904E-4

182 -1.46722E-4 -8.20191E-5 -1.32345E-4 -2.52217E-4

183 -1.37651E-4 -8.60919E-5 -1.32143E-4 -2.45767E-4

184 -1.32097E-4 -8.79432E-5 -1.31069E-4 -2.41176E-4

185 -1.23118E-4 -9.04425E-5 -1.29994E-4 -2.33259E-4

186 -1.17194E-4 -9.1183E-5 -1.28585E-4 -2.27128E-4

187 -1.09233E-4 -9.28492E-5 -1.27778E-4 -2.19559E-4

188 -1.03494E-4 -9.50707E-5 -1.26301E-4 -2.15124E-4

189 -9.66443E-5 -9.77551E-5 -1.25294E-4 -2.09862E-4

190 -8.89614E-5 -9.9051E-5 -1.23883E-4 -2.02246E-4

191 -8.73878E-5 -1.02106E-4 -1.22541E-4 -2.03476E-4

192 -8.0538E-5 -1.0155E-4 -1.21064E-4 -1.94974E-4

193 -7.57246E-5 -1.03772E-4 -1.17606E-4 -1.91613E-4

194 -7.18369E-5 -1.02939E-4 -1.14752E-4 -1.8627E-4

195 -6.65607E-5 -1.05531E-4 -1.1079E-4 -1.82741E-4

196 -6.37837E-5 -1.06919E-4 -1.0703E-4 -1.80908E-4

197 -5.64711E-5 -1.07474E-4 -1.02196E-4 -1.7298E-4

198 -5.67488E-5 -1.10714E-4 -9.74947E-5 -1.76543E-4

199 -5.44346E-5 -1.12843E-4 -9.1586E-5 -1.75987E-4

200 -4.97138E-5 -1.1488E-4 -8.49383E-5 -1.72548E-4

201 -4.63815E-5 -1.17379E-4 -7.83584E-5 -1.71182E-4

202 -4.51781E-5 -1.19878E-4 -7.23823E-5 -1.72285E-4

203 -4.40673E-5 -1.20156E-4 -6.60036E-5 -1.71274E-4

204 -4.02722E-5 -1.19971E-4 -6.04302E-5 -1.66687E-4

205 -3.55513E-5 -1.19045E-4 -5.45213E-5 -1.60285E-4

206 -3.24967E-5 -1.21822E-4 -4.8881E-5 -1.59518E-4

207 -3.12008E-5 -1.26543E-4 -4.37107E-5 -1.62736E-4

208 -2.83313E-5 -1.24877E-4 -3.90776E-5 -1.57741E-4

**Fig. 6 Stress-strain curves of rock samples with different cracks’ inclination angles**

**(a) *β*=0°**

0 0 0 0 0 0 0 0 0 0

6.77753E-8 0.37507 -7.01919E-8 -0.03795 -1.32913E-9 0.15171 -3.93366E-8 0.05119 1.49647E-7 0.30505

1.95878E-7 1.22849 1.94979E-8 0.24666 1.18457E-7 0.66382 7.58751E-8 0.40972 4.32573E-7 1.00898

3.73137E-7 1.70684 3.05465E-7 0.42691 3.73231E-7 0.96018 3.73283E-7 0.6242 8.1604E-7 1.31405

6.20405E-7 1.67423 9.00797E-7 0.88228 8.36661E-7 1.15043 9.55602E-7 0.91472 1.26263E-6 1.3845

9.74922E-7 2.4135 1.7353E-6 0.33205 1.49062E-6 1.2355 1.77426E-6 0.70539 1.70806E-6 1.50182

1.41286E-6 2.84836 2.601E-6 1.29972 2.20762E-6 1.86663 2.64474E-6 1.42485 2.12894E-6 1.65424

1.87909E-6 3.41369 3.5161E-6 2.24841 2.96735E-6 2.54795 3.5659E-6 2.15836 2.58839E-6 1.65439

2.42725E-6 4.09859 4.57288E-6 3.10224 3.85007E-6 3.24037 4.63262E-6 2.85417 3.11333E-6 2.11182

3.1348E-6 4.14208 5.70895E-6 4.13631 4.86406E-6 3.72528 5.81516E-6 3.53771 3.69554E-6 2.75718

3.97044E-6 5.06616 6.89571E-6 5.08501 5.97638E-6 4.56802 7.07965E-6 4.34387 4.35258E-6 3.25001

4.84184E-6 5.81631 8.18776E-6 5.8914 7.16628E-6 5.26847 8.44472E-6 5.02195 5.03535E-6 3.41416

5.75495E-6 6.30553 9.56431E-6 7.16265 8.42559E-6 6.06068 9.89445E-6 5.9505 5.73566E-6 3.57839

6.76487E-6 7.33833 1.09577E-5 8.69005 9.74741E-6 7.21277 1.13878E-5 7.15626 6.46518E-6 3.82482

7.94163E-6 8.31678 1.23746E-5 9.9613 1.11739E-5 8.22514 1.29517E-5 8.18389 7.24965E-6 3.91872

9.36864E-6 8.36026 1.37784E-5 12.0674 1.27309E-5 9.19245 1.45801E-5 9.56693 8.15216E-6 4.79872

1.09625E-5 8.66467 1.51953E-5 13.73711 1.43868E-5 10.08081 1.62701E-5 10.71806 9.11784E-6 5.54957

1.2595E-5 9.96925 1.67811E-5 16.10885 1.61569E-5 11.73515 1.81159E-5 12.52979 1.00754E-5 6.3943

1.42276E-5 10.59981 1.85333E-5 18.04419 1.80185E-5 12.8898 2.01035E-5 13.92029 1.11066E-5 7.2742

1.59093E-5 12.03486 2.04051E-5 20.26414 1.99729E-5 14.53455 2.22079E-5 15.65941 1.2216E-5 8.14247

1.7603E-5 13.1329 2.23847E-5 22.46511 2.19932E-5 16.0191 2.44079E-5 17.3179 1.34553E-5 8.90514

1.93115E-5 13.88303 2.44736E-5 24.90326 2.40818E-5 17.45383 2.67055E-5 19.06069 1.49143E-5 8.97555

2.11407E-5 15.32896 2.66898E-5 27.79678 2.63068E-5 19.40658 2.91481E-5 21.24152 1.6481E-5 10.75879

2.31263E-5 16.32915 2.9092E-5 30.40569 2.87201E-5 21.03068 3.17966E-5 23.14637 1.81703E-5 11.83825

2.52698E-5 17.29672 3.17567E-5 33.00511 3.13646E-5 22.63583 3.47167E-5 25.03842 2.00666E-5 13.25782

2.74431E-5 18.63393 3.47502E-5 35.69941 3.42063E-5 24.45 3.79261E-5 27.06724 2.21815E-5 14.73614

2.96789E-5 19.99287 3.80116E-5 37.92884 3.72298E-5 26.06477 4.13828E-5 28.79713 2.4494E-5 15.82745

3.19877E-5 21.35183 4.14783E-5 40.86031 4.04063E-5 27.99546 4.50365E-5 30.98509 2.69994E-5 17.46976

3.42981E-5 23.00431 4.50983E-5 43.96254 4.3668E-5 30.13509 4.88215E-5 33.34393 2.97598E-5 19.52317

3.67261E-5 24.31977 4.8877E-5 46.02122 4.70817E-5 31.65345 5.27773E-5 34.9536 3.28486E-5 20.63761

3.93388E-5 25.64611 5.28741E-5 49.12345 5.07171E-5 33.64631 5.69752E-5 37.24639 3.6218E-5 22.11584

4.21242E-5 27.38557 5.71194E-5 51.29596 5.4584E-5 35.40669 6.14369E-5 39.01619 3.98375E-5 23.89935

4.50438E-5 29.19025 6.16207E-5 53.97128 5.86655E-5 37.42269 6.61574E-5 41.12729 4.3788E-5 26.05821

4.80929E-5 30.66879 6.63782E-5 56.67507 6.29591E-5 39.30474 7.11355E-5 43.19092 4.80729E-5 28.08778

5.13089E-5 32.61481 7.14034E-5 59.10373 6.74918E-5 41.27334 7.63923E-5 45.16968 5.26172E-5 30.7043

5.46873E-5 34.06073 7.67094E-5 61.36163 7.22682E-5 42.94005 8.19377E-5 46.93576 5.74422E-5 32.38209

5.81654E-5 36.03936 8.23989E-5 63.41081 7.73104E-5 44.75258 8.78401E-5 48.67353 6.25828E-5 35.42078

6.18089E-5 38.3985 8.84367E-5 65.56435 8.26351E-5 46.78329 9.40895E-5 50.55643 6.80799E-5 37.99026

6.56445E-5 40.11621 9.49281E-5 67.88865 8.83149E-5 48.60219 1.00784E-4 52.42088 7.39278E-5 39.82053

6.95919E-5 41.88829 1.01953E-4 68.66658 9.43497E-5 49.74969 1.07966E-4 53.28732 8.0096E-5 41.95587

7.36718E-5 44.08435 1.09459E-4 70.41218 1.00722E-4 51.52344 1.156E-4 54.87103 8.66456E-5 43.95038

7.79409E-5 46.20431 1.17473E-4 72.46856 1.07478E-4 53.40279 1.23723E-4 56.64211 9.35256E-5 45.40513

8.24081E-5 48.53084 1.25664E-4 73.91286 1.1444E-4 55.09967 1.32057E-4 58.05564 1.00723E-4 47.55227

8.70526E-5 50.97695 1.33872E-4 74.03619 1.21509E-4 56.25591 1.40459E-4 58.63145 1.08278E-4 49.2184

9.18803E-5 52.97732 1.4234E-4 74.42515 1.28821E-4 57.33111 1.49139E-4 59.29032 1.16185E-4 50.83757

9.69225E-5 55.29298 1.51266E-4 75.94306 1.36504E-4 59.05621 1.58273E-4 60.74968 1.24451E-4 52.3745

1.02335E-4 57.21724 1.60771E-4 77.812 1.44708E-4 60.76317 1.68014E-4 62.35882 1.33026E-4 53.67679

1.08179E-4 60.14556 1.70919E-4 80.00348 1.53504E-4 63.06706 1.78433E-4 64.38175 1.41939E-4 55.67115

1.14313E-4 63.10966 1.81696E-4 81.8155 1.62805E-4 65.21632 1.89476E-4 66.16432 1.5123E-4 56.98536

1.20675E-4 65.32746 1.93101E-4 83.49468 1.72577E-4 66.96997 2.01123E-4 67.70909 1.6102E-4 58.61615

1.27269E-4 66.94733 2.0507E-4 85.67668 1.82786E-4 68.68081 2.13321E-4 69.46087 1.71434E-4 60.42356

1.3409E-4 68.89335 2.17481E-4 87.34638 1.93364E-4 70.30788 2.25965E-4 70.94442 1.82359E-4 62.66395

1.41211E-4 70.90459 2.30355E-4 89.90786 2.04361E-4 72.36561 2.39094E-4 73.02306 1.93688E-4 63.66128

1.48664E-4 72.72015 2.43738E-4 92.14679 2.15821E-4 74.19012 2.52758E-4 74.85161 2.05394E-4 65.32728

1.56377E-4 74.57919 2.57487E-4 93.79751 2.27625E-4 75.76952 2.66812E-4 76.30516 2.17494E-4 66.41846

1.64306E-4 76.40563 2.7149E-4 96.61514 2.39688E-4 77.85934 2.81148E-4 78.51352 2.30016E-4 67.84978

1.72476E-4 77.78632 2.85639E-4 99.28097 2.51963E-4 79.68028 2.95681E-4 80.53255 2.4302E-4 69.18741

1.80922E-4 79.73234 2.99871E-4 101.18785 2.64436E-4 81.41408 3.10369E-4 82.17087 2.56502E-4 70.32542

1.89612E-4 81.45005 3.14229E-4 103.72087 2.77113E-4 83.32691 3.25238E-4 84.1715 2.70446E-4 71.85059

1.98514E-4 82.90684 3.28684E-4 105.97876 2.89959E-4 84.99852 3.40254E-4 85.93978 2.84824E-4 73.01203

2.07624E-4 84.86372 3.43274E-4 108.02794 3.02994E-4 86.80125 3.55447E-4 87.67314 2.99575E-4 74.70159

2.16892E-4 86.62493 3.57954E-4 109.63125 3.16165E-4 88.31528 3.70766E-4 89.07594 3.14694E-4 76.33238

2.26248E-4 88.14695 3.7265E-4 111.31044 3.29394E-4 89.75583 3.86124E-4 90.47982 3.30181E-4 77.92812

2.35732E-4 89.83205 3.87411E-4 112.56272 3.42729E-4 91.07764 4.01577E-4 91.63816 3.45992E-4 79.54713

2.4536E-4 91.79982 4.02273E-4 114.0996 3.56198E-4 92.65473 4.17159E-4 93.03945 3.62151E-4 81.37738

2.55084E-4 92.97394 4.17118E-4 115.37086 3.69711E-4 93.75516 4.32756E-4 94.10671 3.78722E-4 82.43334

2.64963E-4 94.90909 4.31915E-4 116.39544 3.83283E-4 95.08704 4.48359E-4 95.16712 3.95654E-4 83.72398

2.75144E-4 96.19194 4.46778E-4 118.16001 3.97057E-4 96.45837 4.64109E-4 96.57828 4.12945E-4 84.92077

2.8555E-4 97.09428 4.61597E-4 118.63436 4.10931E-4 97.07788 4.7989E-4 97.07051 4.30613E-4 86.00002

2.96074E-4 99.24686 4.76243E-4 120.08586 4.24774E-4 98.69972 4.9556E-4 98.45351 4.48562E-4 86.93887

3.06723E-4 100.6058 4.90733E-4 121.61327 4.38601E-4 99.99858 5.11134E-4 99.72533 4.66746E-4 88.89802

3.17432E-4 102.4866 5.05027E-4 122.97938 4.52352E-4 101.45969 5.26559E-4 100.99758 4.85164E-4 89.66082

3.28227E-4 104.56307 5.19107E-4 124.1368 4.66034E-4 102.91494 5.41827E-4 102.17328 5.03877E-4 90.97474

3.39108E-4 106.40037 5.32985E-4 125.61675 4.79651E-4 104.4077 5.5695E-4 103.51101 5.22845E-4 91.4675

3.49936E-4 108.60732 5.4663E-4 127.31493 4.93111E-4 106.165 5.71858E-4 105.06597 5.42034E-4 92.61731

3.60739E-4 110.59682 5.5996E-4 128.48183 5.06384E-4 107.58539 5.86489E-4 106.23025 5.61466E-4 93.70848

3.71467E-4 112.60806 5.72855E-4 129.78154 5.19377E-4 109.07531 6.00728E-4 107.48559 5.81111E-4 94.4125

3.81973E-4 114.82587 5.85292E-4 130.92946 5.31996E-4 110.58989 6.14508E-4 108.68372 6.00987E-4 95.5738

3.92297E-4 117.31546 5.97239E-4 132.39046 5.44245E-4 112.36767 6.27816E-4 110.14115 6.21143E-4 95.56216

4.02534E-4 119.06579 6.08713E-4 134.14555 5.56186E-4 113.9451 6.40694E-4 111.64079 6.41494E-4 96.10193

4.12706E-4 121.15316 6.19771E-4 135.35037 5.67862E-4 115.42659 6.53198E-4 112.84963 6.62005E-4 96.4188

4.22811E-4 122.44686 6.30538E-4 137.29522 5.79342E-4 116.88393 6.65434E-4 114.38062 6.82758E-4 96.06686

4.3283E-4 124.42549 6.41111E-4 138.57594 5.90668E-4 118.35064 6.77478E-4 115.61697 7.03692E-4 96.87622

4.42849E-4 125.70834 6.51425E-4 140.37848 6.01851E-4 119.73907 6.89302E-4 117.05289 7.24758E-4 97.13417

4.52912E-4 126.55632 6.61356E-4 142.26638 6.12847E-4 120.97022 7.00812E-4 118.45647 7.45966E-4 97.66229

4.62907E-4 127.8881 6.71001E-4 143.51865 6.23649E-4 122.13304 7.12058E-4 119.54326 7.67278E-4 98.7651

4.72858E-4 128.93721 6.80526E-4 145.24528 6.34361E-4 123.38213 7.23188E-4 120.88233 7.88604E-4 99.27571

4.82772E-4 129.5895 6.89873E-4 147.1237 6.44955E-4 124.52094 7.34155E-4 122.24009 8.08E-4 99.83286

4.92617E-4 130.37224 6.99136E-4 148.74596 6.55464E-4 125.60319 7.4503E-4 123.45712 8.32E-4 100.65414

5.0239E-4 131.86167 7.08362E-4 149.97928 6.65914E-4 126.82843 7.55852E-4 124.56346 8.56E-4 101.26432

5.12126E-4 133.63375 7.17566E-4 151.70589 6.76331E-4 128.40284 7.66643E-4 126.04893 8.72E-4 101.60449

5.21875E-4 135.46018 7.2674E-4 152.60715 6.86738E-4 129.63029 7.77413E-4 127.00686 8.96E-4 102.44919

5.31685E-4 137.51491 7.35906E-4 153.56534 6.97175E-4 130.98612 7.88195E-4 128.04816 9.12E-4 102.8599

5.41512E-4 139.42831 7.45109E-4 154.51403 7.07642E-4 132.27406 7.99013E-4 129.05464 9.36E-4 103.77518

5.51403E-4 141.43954 7.54312E-4 155.46273 7.18143E-4 133.60602 8.0985E-4 130.08093 9.52E-4 103.66956

5.61383E-4 143.20075 7.63603E-4 156.33554 7.28742E-4 134.79133 8.2079E-4 131.00709 9.76E-4 103.99807

5.71337E-4 145.41857 7.72949E-4 156.8668 7.39357E-4 136.02842 8.31768E-4 131.80284 9.92E-4 104.59632

5.81261E-4 147.1254 7.82208E-4 157.22731 7.49908E-4 136.95872 8.42664E-4 132.38371 0.00102 104.37328

5.91164E-4 148.88661 7.9138E-4 157.61627 7.60399E-4 137.92629 8.53479E-4 132.99415 0.00103 105.01882

6.01008E-4 150.64781 8.00457E-4 157.69216 7.70806E-4 138.75299 8.64195E-4 133.40031 0.00106 105.26514

6.10707E-4 151.59362 8.09436E-4 157.07551 7.81079E-4 138.90111 8.74783E-4 133.18948 0.00107 104.9132

6.20308E-4 153.82232 8.18319E-4 157.45499 7.91245E-4 140.07479 8.8526E-4 133.8884 0.00109 105.37062

6.29817E-4 155.10516 8.26994E-4 157.17038 8.01246E-4 140.52399 8.95532E-4 133.96247 0.0011 104.92484

6.39157E-4 156.23036 8.35476E-4 157.13243 8.11048E-4 141.01326 9.05588E-4 134.16556 0.00113 105.26514

6.48391E-4 157.61649 8.43686E-4 156.38296 8.20642E-4 141.29975 9.15381E-4 133.95722 0.00114 105.49952

6.57549E-4 159.17115 8.51552E-4 156.09835 8.30006E-4 141.87127 9.24857E-4 134.08633 0.00116 105.73434

6.66563E-4 159.73647 8.59066E-4 155.7758 8.39096E-4 141.98053 9.33989E-4 133.99034 0.00118 105.75777

6.75445E-4 161.13891 8.66214E-4 155.09274 8.47912E-4 142.30425 9.4277E-4 133.82864 0.00119 106.33259

6.84221E-4 161.30197 8.73091E-4 154.49506 8.56522E-4 142.10867 9.51287E-4 133.47167 0.00121 106.03957

6.92788E-4 162.04125 8.79612E-4 153.65073 8.6482E-4 142.06139 9.59438E-4 133.07045 0.00122 106.55577

7.01173E-4 162.22605 8.85828E-4 153.4515 8.72851E-4 142.05489 9.67273E-4 132.97788 0.00124 106.39137

7.09388E-4 162.84575 8.9177E-4 152.12332 8.80637E-4 141.73608 9.74824E-4 132.23673 0.00126 106.00438

7.17257E-4 163.11754 8.97288E-4 151.58256 8.88E-4 141.61504 9.81908E-4 131.93892 0.00127 105.78147

7.24791E-4 163.25887 9.02407E-4 151.1936 9.76319E-4 141.50361 0.00113 131.71374 0.00129 104.831

7.32068E-4 163.6285 9.072E-4 150.58644 9.83561E-4 141.39672 0.00113 131.39242 0.0013 104.5729

7.39021E-4 163.44368 9.11677E-4 150.26389 9.90419E-4 141.16841 0.00114 131.14453 0.00131 103.97435

7.45735E-4 163.42193 9.15863E-4 149.78953 9.96959E-4 140.94515 0.00115 130.83061 0.00133 103.54065

7.52219E-4 163.15015 9.19779E-4 149.35314 1E-3 140.62648 0.00115 130.49083 0.00134 103.45816

7.58451E-4 163.40019 9.23456E-4 148.7934 0.00101 140.48711 0.00116 130.17623 0.00135 103.51679

7.64457E-4 163.09579 9.26904E-4 147.89213 0.00101 139.94456 0.00117 129.52652 0.00137 103.68105

7.70202E-4 162.37826 9.30087E-4 147.20908 0.00102 139.3143 0.00117 128.93552 0.00138 103.51694

7.75715E-4 162.30217 9.32937E-4 146.38372 0.00103 138.90865 0.00117 128.38157 0.00139 103.81025

7.8104E-4 161.57376 9.35467E-4 145.805 0.00103 138.32044 0.00118 127.85645 0.0014 103.73983

7.86214E-4 160.75838 9.3774E-4 144.91324 0.00103 137.55223 0.00118 127.10947 0.00142 103.38803

7.91208E-4 160.63881 9.39707E-4 143.5566 0.00104 136.88794 0.00119 126.20004 0.00142 103.45831

7.96052E-4 159.58426 9.41383E-4 142.59842 0.00104 135.98221 0.00119 125.36128 0.00143 103.18828

8.00749E-4 158.58407 9.42707E-4 141.22281 0.00105 134.91309 0.00119 124.26116 0.00145 103.15322

8.05284E-4 157.89916 9.43669E-4 139.56259 0.00105 133.85779 0.0012 123.03917 0.00146 102.78949

8.09744E-4 156.6924 9.44303E-4 138.34826 0.00105 132.7683 0.0012 122.00245 0.00146 102.66044

8.14003E-4 155.90966 9.44626E-4 136.90624 0.00106 131.76716 0.0012 120.90303 0.00148 102.33193

8.17935E-4 154.96382 9.44702E-4 136.03345 0.00106 130.94877 0.0012 120.142 0.00149 101.61627

8.21613E-4 154.22456 9.44479E-4 134.37322 0.00106 129.86901 0.0012 118.909 0.0015 101.34652

8.25099E-4 152.95258 9.43913E-4 133.17788 0.00106 128.7587 0.0012 117.87146 0.0015 100.68936

8.28365E-4 151.80019 9.43024E-4 131.57457 0.00106 127.51865 0.0012 116.59195 0.00151 100.44302

8.31374E-4 150.94133 9.41765E-4 130.51204 0.00106 126.65402 0.0012 115.72472 0.00152 99.87976

8.34118E-4 149.89766 9.40191E-4 128.83284 0.00106 125.42873 0.0012 114.41771 0.00153 98.85907

8.3657E-4 148.85399 9.38402E-4 126.88801 0.00106 124.0839 0.0012 112.93736 0.00154 98.22538

8.38791E-4 147.98426 9.36333E-4 125.69267 0.00107 123.15461 0.0012 111.98128 0.00154 98.0612

8.40709E-4 147.23412 9.33971E-4 124.55423 0.00106 122.30476 0.0012 111.08654 0.00155 97.32204

8.42311E-4 146.12521 9.31316E-4 123.25451 0.00106 121.22087 0.0012 110.01393 0.00156 97.00529

8.43629E-4 145.35333 9.28294E-4 122.24889 0.00106 120.421 0.00119 109.20146 0.00157 96.5126

8.44605E-4 144.08135 9.24847E-4 121.59429 0.00106 119.55405 0.00119 108.51675 0.00158 96.40692

8.45272E-4 143.30947 9.2102E-4 119.67793 0.00106 118.34433 0.00119 107.11002 0.00158 95.7382

8.45644E-4 142.9181 9.16962E-4 118.7482 0.00106 117.74983 0.00118 106.42412 0.00158 95.44487

8.45765E-4 141.59175 9.12684E-4 117.53388 0.00106 116.60653 0.00118 105.36319 0.00159 95.15168

8.45647E-4 140.68941 9.0813E-4 115.98751 0.00105 115.50461 0.00118 104.17145 0.00159 94.44758

8.45288E-4 139.96101 9.03292E-4 114.27987 0.00105 114.4084 0.00117 102.90972 0.0016 94.09569

8.44731E-4 138.94996 8.98143E-4 112.66708 0.00105 113.22766 0.00117 101.65263 0.0016 93.4152

8.43998E-4 138.21068 8.92659E-4 110.77917 0.00104 112.04543 0.00116 100.27108 0.00161 92.55871

8.43052E-4 137.64535 8.86771E-4 109.02409 0.00104 111.00124 0.00115 99.0114 0.00161 91.43245

8.41925E-4 137.19963 8.80417E-4 107.10773 0.00103 109.93831 0.00115 97.67072 0.00162 90.54067

8.4068E-4 136.03636 8.7363E-4 105.19136 0.00103 108.55247 0.00114 96.18472 0.00162 89.1445

8.39266E-4 135.55801 8.66522E-4 103.64499 0.00102 107.64135 0.00113 95.07885 0.00162 88.10027

8.37647E-4 134.63392 8.59066E-4 100.8653 0.00102 105.97465 0.00113 93.07799 0.00162 86.83297

8.35907E-4 133.77506 8.51159E-4 99.1956 0.00101 104.8368 0.00112 91.81458 0.00162 85.76536

8.3403E-4 132.66616 8.42806E-4 97.34565 0.00101 103.50532 0.00111 90.38293 0.00162 84.47489

8.31995E-4 131.64422 8.33981E-4 95.60004 9.99586E-4 102.25992 0.0011 89.03698 0.00162 83.325

8.29794E-4 131.10066 8.24718E-4 93.40856 9.92707E-4 101.02915 0.00109 87.49697 0.00162 81.97583

8.27425E-4 129.75256 8.15057E-4 92.00449 9.85489E-4 99.79067 0.00108 86.30782 0.00162 80.36855

8.24956E-4 129.27423 8.05029E-4 90.72375 9.77991E-4 98.99909 0.00107 85.37527 0.00162 79.12484

8.22365E-4 128.36101 7.94674E-4 89.24379 9.70223E-4 97.92216 0.00106 84.22467 0.00162 77.85759

8.19644E-4 127.176 7.83989E-4 87.96305 9.6218E-4 96.81257 0.00105 83.14903 0.00162 76.15642

8.16767E-4 126.18669 7.73039E-4 86.65384 9.53884E-4 95.77824 0.00104 82.09444 0.00162 74.98322

8.13843E-4 124.71902 7.618E-4 85.40157 9.45386E-4 94.55427 0.00102 80.98013 0.00162 73.72799

8.1087E-4 123.27311 7.50275E-4 83.86468 9.36687E-4 93.212 0.00101 79.68451 0.00162 72.95341

8.07705E-4 121.49016 7.38534E-4 82.26139 9.27743E-4 91.6882 9.99766E-4 78.27731 0.00162 71.53386

8.04316E-4 120.39214 7.26643E-4 80.8763 9.18575E-4 90.5708 9.87131E-4 77.15119 0.00161 71.07615

8.00643E-4 119.38107 7.1469E-4 79.64299 9.092E-4 89.56083 9.74334E-4 76.14172 0.00161 69.59795

7.96877E-4 117.33722 7.02489E-4 78.50455 8.9962E-4 88.1288 9.61265E-4 74.985 0.0016 68.45993

7.9304E-4 116.01088 6.89932E-4 76.96767 8.89783E-4 86.84035 9.47829E-4 73.71361 0.0016 67.50943

7.88991E-4 114.48886 6.76916E-4 75.76282 8.79544E-4 85.61325 9.33876E-4 72.61923 0.00159 65.70271

7.84837E-4 112.70591 6.6337E-4 72.9452 8.68924E-4 83.543 9.19377E-4 70.41968 0.00159 64.7054

7.80558E-4 111.32521 6.49552E-4 70.75372 8.58066E-4 81.93552 9.04571E-4 68.71015 0.00158 64.0248

7.76141E-4 109.46618 6.35713E-4 69.62476 8.47112E-4 80.59092 8.89695E-4 67.59706 0.00158 62.87512

7.71555E-4 107.82456 6.21908E-4 68.96068 8.36078E-4 79.55336 8.74791E-4 66.83132 0.00158 62.39419

7.66778E-4 106.48735 6.08112E-4 68.70452 8.24934E-4 78.83635 8.59828E-4 66.39339 0.00157 61.64329

7.6179E-4 104.78051 5.94289E-4 67.93608 8.13647E-4 77.72247 8.44762E-4 65.54634 0.00156 60.69279

7.56581E-4 103.80206 5.80407E-4 67.24353 8.02193E-4 76.97052 8.2956E-4 64.89633 0.00155 60.04762

7.51213E-4 101.92128 5.66503E-4 66.74073 7.9063E-4 75.89791 8.1428E-4 64.18738 0.00154 59.2849

7.45712E-4 100.35577 5.52574E-4 65.80152 7.78972E-4 74.77078 7.98927E-4 63.25753 0.00154 58.55754

7.40105E-4 98.90985 5.38741E-4 65.45525 7.67308E-4 73.9643 7.83629E-4 62.73879 0.00154 58.36984

7.34348E-4 97.60525 5.24956E-4 63.55311 7.55582E-4 72.52127 7.68323E-4 61.23346 0.00153 57.37247

7.28351E-4 96.48548 5.11108E-4 62.78467 7.43675E-4 71.67156 7.5287E-4 60.5053 0.00152 56.89153

7.22184E-4 94.96345 4.97106E-4 62.31981 7.31574E-4 70.77747 7.37208E-4 59.89378 0.00151 56.35181

7.15846E-4 94.1807 4.82873E-4 60.87779 7.19231E-4 69.77632 7.21263E-4 58.79435 0.0015 55.89432

7.09329E-4 92.60432 4.68542E-4 59.45475 7.06723E-4 68.42658 0.0015 55.53042

7.0265E-4 91.83243 4.54218E-4 58.21196 6.94121E-4 67.51998 0.0015 54.22818

6.95829E-4 90.31041 6.81433E-4 66.4167 0.00149 53.55939

6.88885E-4 89.38632 6.6868E-4 65.27937 0.00148 52.83202

6.81801E-4 88.53834 6.5583E-4 64.44098 0.00147 52.31574

6.74527E-4 87.23374 6.42818E-4 63.15803 0.00147 50.96657

6.67067E-4 86.39663 6.29644E-4 62.10255 0.00146 50.0749

6.59496E-4 85.30947 6.16318E-4 60.96442 0.00146 49.20674

6.51775E-4 84.25493 6.02819E-4 59.87299 0.00145 48.29161

6.43931E-4 83.02644 0.00145 46.6137

6.35943E-4 81.41743 -- -- 0.00144 45.17058

6.27827E-4 80.31941 -- -- 0.00143 44.48415

6.19543E-4 78.38969 -- -- 0.00142 43.34035

6.11028E-4 77.30797 -- -- 0.00142 41.82683

6.02289E-4 75.67723 -- -- 0.00142 40.11378

5.93305E-4 74.2748 -- -- 0.00141 39.2221

5.84124E-4 72.8941 -- -- -- -- -- -- 0.00141 38.22479

5.7479E-4 70.9807 -- -- -- -- -- -- 0.0014 37.11023

5.65272E-4 69.19775 -- -- -- -- -- -- 0.00139 36.28905

5.55551E-4 67.39307 -- -- -- -- -- -- 0.00139 36.03097

5.45598E-4 66.06673 -- -- -- -- -- -- 0.00138 34.62303

5.35404E-4 64.91434 -- -- -- -- -- -- 0.00138 34.1772

5.24994E-4 63.18576 -- -- -- -- -- -- 0.00138 33.75485

5.14321E-4 61.43543 -- -- -- -- -- -- 0.00137 33.43791

-- -- -- -- -- -- 0.00136 32.53455

**(b) *β*=30°**

0 0 0 0 0 0 0 0

7.24055E-8 0.24615 5.75146E-8 0.18044 3.44424E-8 -0.20119 1.38187E-7 0.16124

2.04979E-7 0.23769 1.44608E-7 0.49966 8.57313E-8 -0.19306 3.96093E-7 0.23721

3.91134E-7 0.31406 2.82638E-7 0.48579 1.93239E-7 0.40142 7.46545E-7 -0.13616

6.70579E-7 0.19523 4.86398E-7 1.15201 4.00725E-7 0.32552 1.1766E-6 -0.31352

1.07576E-6 0.25463 7.19738E-7 1.81825 7.5814E-7 0.29832 1.70193E-6 -0.34076

1.56236E-6 0.16977 9.39928E-7 2.09597 1.23521E-6 0.71783 2.3396E-6 -0.48802

2.13432E-6 0.49222 1.23078E-6 1.90144 1.85007E-6 0.74667 3.01164E-6 -0.48157

2.80791E-6 0.65353 1.63994E-6 2.41499 2.64387E-6 1.03513 3.75701E-6 -0.17737

3.59139E-6 0.89103 2.12795E-6 3.06731 3.62181E-6 1.26272 4.60378E-6 0.35867

4.48438E-6 0.86559 2.72605E-6 3.52542 4.7651E-6 1.52404 5.59228E-6 0.86138

5.48584E-6 1.10333 3.36196E-6 4.38608 6.05054E-6 1.87521 6.74483E-6 1.07888

6.59678E-6 1.36636 3.97815E-6 4.78859 7.48805E-6 2.73426 8.05906E-6 1.30425

7.79808E-6 1.87547 4.59105E-6 4.94124 9.09673E-6 3.13371 9.55522E-6 1.47014

9.09568E-6 2.36771 5.32387E-6 5.8434 1.08931E-5 3.74482 1.12047E-5 1.24298

1.05233E-5 2.57991 6.29991E-6 6.20429 1.28887E-5 4.35959 1.29101E-5 1.98755

1.20759E-5 2.80055 7.40742E-6 6.39864 1.50559E-5 5.49899 1.46343E-5 2.25449

1.37467E-5 3.06365 8.62504E-6 6.84275 1.74453E-5 6.16054 1.64148E-5 2.63538

1.55535E-5 3.6153 1.01006E-5 7.57856 2.015E-5 6.86813 1.82542E-5 3.31223

1.73651E-5 4.26877 1.17535E-5 8.21688 2.3107E-5 7.97431 2.019E-5 3.96542

1.91472E-5 5.88966 1.35544E-5 9.1886 2.63101E-5 9.06663 2.21464E-5 4.87769

2.10439E-5 7.4936 1.56379E-5 10.16007 2.98373E-5 10.12794 2.40802E-5 5.7018

2.308E-5 8.63932 1.79202E-5 11.07625 3.36509E-5 11.13469 2.60485E-5 6.18122

2.5269E-5 9.3351 2.05066E-5 12.00623 3.77347E-5 12.62303 2.80736E-5 6.82296

2.7678E-5 10.345 2.33837E-5 12.56134 4.21427E-5 13.98035 3.01974E-5 8.25226

3.03384E-5 10.8033 2.65418E-5 13.29706 4.68688E-5 15.15502 3.24704E-5 9.62491

3.32664E-5 11.59253 3.0063E-5 14.57402 5.19231E-5 16.60769 3.49057E-5 10.17422

3.64891E-5 12.23747 3.39358E-5 15.11534 5.73472E-5 18.00297 3.75541E-5 10.75651

4.00258E-5 12.79759 3.82426E-5 16.35062 6.31597E-5 19.42991 4.04957E-5 11.76518

4.38779E-5 13.51911 4.28975E-5 17.33624 6.93438E-5 20.82191 4.37361E-5 12.78814

4.80574E-5 14.57975 4.79354E-5 18.40492 7.59191E-5 22.44863 4.71544E-5 14.1214

5.25721E-5 15.52174 5.34102E-5 19.82074 8.28719E-5 24.00644 5.08402E-5 15.71695

5.73952E-5 16.33655 5.92418E-5 20.90338 9.01642E-5 25.47045 5.49149E-5 16.39558

6.25072E-5 17.76226 6.54709E-5 22.22188 9.78087E-5 27.18934 5.93232E-5 17.84638

6.79084E-5 19.15403 7.20943E-5 23.6515 1.05758E-4 28.68973 6.40419E-5 19.05179

7.3593E-5 20.69002 7.90743E-5 25.46978 1.13999E-4 30.54682 6.90117E-5 20.73192

7.95612E-5 22.65039 8.64704E-5 26.85786 1.22557E-4 32.39659 7.41969E-5 22.3862

8.57924E-5 24.29693 9.42336E-5 28.84274 1.31442E-4 34.20707 7.9578E-5 24.11823

9.22774E-5 26.47807 1.0229E-4 30.38347 1.40658E-4 35.6983 8.51947E-5 25.57554

9.90268E-5 28.54854 1.10688E-4 32.81249 1.50187E-4 37.56993 9.10292E-5 26.91952

1.0604E-4 30.82287 1.19502E-4 34.90833 1.60069E-4 39.14476 9.70892E-5 28.5337

1.1339E-4 32.94459 1.28783E-4 38.16043 1.70349E-4 40.85816 1.03337E-4 29.8602

1.21138E-4 35.59241 1.38463E-4 41.23769 1.81062E-4 43.06021 1.09835E-4 31.47651

1.29208E-4 38.01085 1.4841E-4 43.00034 1.92247E-4 44.66506 1.16714E-4 32.76185

1.37567E-4 40.03075 1.58593E-4 45.0269 2.03903E-4 46.82881 1.23857E-4 34.28784

1.46211E-4 41.76187 1.68934E-4 47.23386 2.15996E-4 48.65919 1.31235E-4 35.58246

1.55073E-4 44.10423 1.79405E-4 49.19084 2.28448E-4 50.17514 1.389E-4 36.69535

1.64166E-4 46.5229 1.90039E-4 51.48117 2.4125E-4 52.17565 1.46814E-4 38.40986

1.73481E-4 48.83113 2.00846E-4 53.09132 2.54401E-4 54.09893 1.54919E-4 39.51774

1.82952E-4 51.58949 2.11922E-4 54.96494 2.67914E-4 56.2222 1.63268E-4 41.10114

1.92573E-4 54.69526 2.23274E-4 57.00545 2.8179E-4 58.17688 1.71814E-4 41.99868

2.0233E-4 57.43662 2.34828E-4 58.92075 2.95972E-4 60.2387 1.80512E-4 43.06466

2.12222E-4 60.43228 2.46624E-4 60.55864 3.1047E-4 62.2944 1.89374E-4 44.2876

2.22306E-4 63.18192 2.58606E-4 62.40467 3.2532E-4 64.1887 1.98301E-4 45.23068

2.32588E-4 65.77031 2.70765E-4 63.9316 3.40538E-4 66.18697 2.0738E-4 47.22094

2.43044E-4 68.14646 2.83069E-4 65.70805 3.56124E-4 68.14977 2.16731E-4 48.80875

2.53707E-4 70.55663 2.95487E-4 66.54087 3.72034E-4 70.04233 2.26332E-4 50.62934

2.64577E-4 72.44059 3.08044E-4 68.37309 3.88216E-4 71.77003 2.36125E-4 52.07801

2.75615E-4 74.46889 3.207E-4 69.59473 4.04686E-4 73.79572 2.46098E-4 53.74309

2.86853E-4 76.68391 3.33546E-4 70.62173 4.2144E-4 75.57273 2.562E-4 55.18129

2.98303E-4 78.65269 3.46498E-4 72.03755 4.3847E-4 76.94384 2.66502E-4 56.50101

3.0997E-4 80.33301 3.59534E-4 72.55113 4.55755E-4 78.97691 2.77051E-4 57.96323

3.21863E-4 82.41233 3.72733E-4 73.73093 4.73273E-4 80.66626 2.8781E-4 59.49183

3.33959E-4 84.34708 3.85955E-4 75.54906 4.91006E-4 82.48103 2.98781E-4 60.85736

3.46217E-4 86.10382 3.9922E-4 76.45146 5.08893E-4 84.4133 3.09944E-4 62.16478

3.58602E-4 88.04305 4.12518E-4 78.04752 5.26862E-4 86.24917 3.21288E-4 63.97824

3.71102E-4 90.10111 4.25807E-4 79.17184 5.44897E-4 87.8799 3.3273E-4 65.16494

3.8371E-4 92.01908 4.39191E-4 80.89295 5.62873E-4 89.5725 3.44284E-4 66.72828

3.964E-4 93.87741 4.52618E-4 82.82235 5.80762E-4 91.00775 3.55912E-4 67.83223

4.09151E-4 95.85491 4.66066E-4 84.1548 5.98654E-4 92.80692 3.67594E-4 69.20776

4.21926E-4 97.85758 4.79543E-4 86.29217 6.16549E-4 94.50302 3.79396E-4 70.42641

4.34669E-4 100.48011 4.92987E-4 87.87474 6.34419E-4 96.2496 3.91319E-4 70.84203

4.47368E-4 102.83076 5.06414E-4 89.23493 6.52283E-4 97.76755 4.03385E-4 71.53217

4.60493E-4 105.06268 5.19864E-4 90.76186 6.71187E-4 99.3704 4.15612E-4 72.31682

4.73136E-4 107.69361 5.33171E-4 92.63562 6.89211E-4 100.95757 4.28282E-4 73.10121

4.85651E-4 110.03585 5.46223E-4 93.92639 7.07176E-4 102.39369 4.40744E-4 73.83714

4.98034E-4 112.64965 5.59089E-4 95.9666 7.25092E-4 104.22799 4.53373E-4 73.978

5.10281E-4 115.21243 5.71789E-4 98.15975 7.4296E-4 105.88449 4.66049E-4 74.91816

5.20359E-4 117.6312 5.84302E-4 99.92255 7.56573E-4 107.53474 4.78747E-4 75.99706

5.32278E-4 120.33831 5.96603E-4 101.31048 7.74298E-4 109.38542 4.89976E-4 76.48862

5.44032E-4 122.63827 6.08601E-4 102.50423 7.91921E-4 110.96619 5.02703E-4 77.59584

5.55595E-4 124.9549 6.20245E-4 103.94779 8.09432E-4 112.76796 5.15459E-4 78.08922

5.66986E-4 127.18694 6.3161E-4 105.36361 8.26819E-4 113.77173 5.28146E-4 78.96672

5.78218E-4 129.19822 6.42721E-4 106.34908 8.4407E-4 115.37413 5.40698E-4 79.62853

5.89272E-4 131.17561 6.53508E-4 107.04304 8.61201E-4 116.60574 5.53184E-4 80.2151

5.98162E-4 132.58427 6.63954E-4 108.94455 8.74008E-4 117.69781 5.65606E-4 81.25276

6.08911E-4 134.70576 6.74083E-4 110.76298 8.90839E-4 119.06275 5.76481E-4 81.48744

6.19509E-4 136.11477 6.8395E-4 111.74845 9.07521E-4 119.99593 5.88801E-4 81.46738

6.27949E-4 137.26877 6.93639E-4 112.81714 9.19885E-4 121.04815 6.01068E-4 82.1657

6.38227E-4 138.33819 7.03221E-4 115.16984 9.36332E-4 122.06957 6.17546E-4 82.94222

6.48362E-4 139.62814 7.12651E-4 117.38384 9.52697E-4 122.97456 6.37641E-4 83.77343

6.5638E-4 140.63792 7.22048E-4 119.11875 9.64798E-4 124.34773 6.58112E-4 83.75555

6.64299E-4 142.0552 7.3146E-4 120.32629 9.76803E-4 125.53832 6.78838E-4 83.94726

6.74165E-4 143.24344 7.40755E-4 122.54719 9.92925E-4 126.51139 6.99761E-4 84.80214

6.81967E-4 144.67762 7.49983E-4 123.92117 0.00101 127.85233 7.2101E-4 85.6603

6.91689E-4 145.76393 7.59199E-4 125.55892 0.00102 129.02162 7.42552E-4 86.44253

6.99346E-4 146.72292 7.68446E-4 127.07206 0.00103 130.11711 7.64395E-4 87.32724

7.0696E-4 148.22489 7.77657E-4 128.09921 0.00104 131.05622 7.86474E-4 88.14669

7.14495E-4 149.22214 7.86709E-4 128.55715 0.00105 132.12689 8.08657E-4 89.48354

7.21914E-4 150.20228 7.95641E-4 129.23717 0.00107 133.00038 8.3083E-4 90.61542

7.31219E-4 151.13575 8.08E-4 130.26432 0.00108 134.00946 8.53119E-4 91.50584

7.38431E-4 152.28136 8.16E-4 131.44427 0.0011 134.60566 8.75466E-4 92.65813

7.45524E-4 153.29999 8.24E-4 132.37411 0.00111 135.24051 8.97737E-4 93.53098

7.52455E-4 154.41158 8.32E-4 133.69276 0.00112 135.83359 9.20079E-4 94.80305

7.59274E-4 155.31116 8.4E-4 134.40067 0.00113 136.23284 9.42401E-4 95.68408

7.63975E-4 156.15132 8.48E-4 135.17801 0.00114 136.61134 9.64462E-4 97.02954

7.70547E-4 156.88978 8.56E-4 135.42797 0.00115 136.62759 9.8627E-4 97.5541

7.76977E-4 157.36485 8.64E-4 136.27444 0.00116 136.88777 0.00101 98.40228

7.83243E-4 158.0778 8.72E-4 136.62142 0.00117 136.93708 0.00103 99.01993

7.87382E-4 158.53586 8.8E-4 137.57915 0.00118 136.65302 0.00105 100.10064

7.93373E-4 158.65468 8.88E-4 138.95343 0.00119 136.67676 0.00107 100.93262

7.99194E-4 159.01116 8.96E-4 139.6336 0.0012 136.21318 0.00109 101.69155

8.02874E-4 158.57838 9.04E-4 140.09154 0.0012 136.49164 0.00111 102.60482

8.08407E-4 158.56133 9.12E-4 141.27135 0.00121 136.13793 0.00113 103.53472

8.11781E-4 158.2815 9.2E-4 141.78477 0.00121 136.00955 0.00115 103.807

8.14982E-4 158.17113 9.28E-4 141.57679 0.00123 135.81021 0.00118 104.65837

8.20009E-4 157.49221 9.36E-4 142.32624 0.00124 135.50857 0.00119 105.45162

8.22879E-4 157.35646 9.44E-4 143.43661 0.00124 135.26755 0.00121 105.81458

8.2558E-4 156.67755 9.52E-4 143.93624 0.00125 135.29224 0.00123 106.39367

8.30102E-4 155.93918 9.6E-4 143.88072 0.00125 134.81023 0.00125 106.99345

8.32484E-4 155.48943 9.68E-4 144.76907 0.00126 134.71781 0.00127 108.15401

8.34721E-4 154.42006 9.76E-4 145.21325 0.00126 134.18698 0.00129 108.89071

8.36772E-4 153.95333 9.84E-4 145.33822 0.00127 134.2714 0.00131 108.98178

8.38627E-4 153.23198 9.92E-4 146.08774 0.00127 134.00886 0.00133 109.73059

8.40306E-4 152.78231 1E-3 146.82329 0.00127 133.84319 0.00134 110.16273

8.41812E-4 152.27305 0.00101 146.54579 0.00128 133.51847 0.00136 110.38126

8.43142E-4 151.75529 0.00101 146.25429 0.00128 133.20641 0.00138 110.91553

8.44321E-4 151.38191 0.00102 146.15702 0.00128 132.68193 0.0014 110.67909

8.45348E-4 150.66896 0.00102 146.79547 0.00128 131.93595 0.00141 110.88076

8.44232E-4 150.13434 0.00103 146.46248 0.00128 131.47709 0.00143 111.38353

8.44985E-4 149.5404 0.00103 146.51793 0.00128 130.69948 0.00145 111.20037

8.45579E-4 148.60684 0.00104 146.62895 0.00129 129.91137 0.00146 111.20002

8.44039E-4 148.40308 0.00105 146.6012 0.00128 129.35379 0.00148 111.66119

8.44369E-4 147.63093 0.00105 146.85107 0.00129 128.25183 0.00149 111.27353

8.4454E-4 146.95204 0.00106 146.89267 0.00129 127.69321 0.00151 111.51108

8.42553E-4 146.10333 0.00106 146.35137 0.00129 126.91051 0.00152 111.73753

8.40406E-4 145.32252 0.00106 145.83778 0.00129 126.11501 0.00154 111.2305

8.40118E-4 144.31271 0.00106 146.00446 0.00129 125.16216 0.00155 111.28887

8.37663E-4 143.34528 0.00106 145.50467 0.00129 124.25467 0.00157 111.32003

8.35038E-4 142.45423 0.00107 145.08836 0.00129 123.63441 0.00158 110.88977

8.34282E-4 141.27447 0.00107 144.60243 0.00129 122.27583 0.00159 111.06569

8.31392E-4 139.94221 0.00108 144.21388 0.00129 121.25487 0.0016 110.98293

8.28358E-4 138.65217 0.00108 143.58939 0.00128 120.15753 0.00162 110.05161

8.25205E-4 137.33674 0.00108 143.506 0.00128 118.90246 0.00163 109.75206

8.21951E-4 135.61413 0.00109 143.50596 0.00128 117.55215 0.00164 110.04269

8.18567E-4 133.94223 0.00109 142.49277 0.00128 116.12599 0.00165 109.36154

8.15003E-4 132.60982 0.00109 141.6322 0.00128 114.80525 0.00166 108.72766

8.11306E-4 131.07376 0.00109 141.06302 0.00128 113.46387 0.00167 108.40951

8.07511E-4 129.51223 0.0011 141.03533 0.00127 112.00098 0.00169 107.489

8.03608E-4 128.31572 0.0011 140.23026 0.00127 110.66928 0.0017 107.37719

7.97607E-4 127.35671 0.0011 139.34202 0.00126 109.41141 0.00171 107.18089

7.93525E-4 126.59285 0.0011 138.92554 0.00125 107.79388 0.00172 106.38502

7.89333E-4 125.19264 0.0011 138.81449 0.00125 106.19802 0.00173 105.74043

7.83003E-4 124.08092 0.0011 138.37044 0.00124 104.79278 0.00173 105.66305

7.78546E-4 123.15586 0.0011 137.50987 0.00124 103.37963 0.00174 104.90807

7.71956E-4 121.61986 0.0011 136.84356 0.00123 101.76012 0.00175 104.03632

7.67231E-4 120.55914 0.0011 136.59365 0.00123 100.41823 0.00176 104.19933

7.60387E-4 118.98067 0.0011 135.40011 0.00121 99.1094 0.00177 103.52393

7.55413E-4 117.47846 0.0011 133.91484 0.00121 97.41918 0.00177 102.46042

7.48313E-4 116.48536 0.0011 133.55392 0.0012 96.17594 0.00178 102.37878

7.41087E-4 115.0597 0.0011 132.85992 0.0012 94.56305 0.00178 101.98822

7.33751E-4 113.41339 0.0011 131.68016 0.00119 93.01536 0.00179 101.58833

7.28317E-4 111.74157 0.0011 130.56986 0.00118 91.6133 0.00179 100.95373

7.20772E-4 109.91702 0.0011 129.79239 0.00117 90.17749 0.0018 100.08699

7.13118E-4 108.10098 0.0011 128.02971 0.00116 88.93755 0.0018 100.17843

7.05343E-4 106.21668 0.0011 127.34955 0.00115 87.6237 0.0018 99.7559

6.97454E-4 104.1122 0.0011 126.44746 0.00114 86.03993 0.00182 99.16975

6.91461E-4 102.36395 0.0011 125.07331 0.00114 84.91268 0.00182 98.58637

6.83366E-4 100.42496 0.0011 124.18487 0.00113 83.36013 0.00182 97.65762

6.75186E-4 98.3923 0.0011 123.71303 0.00112 82.24522 0.00182 97.1872

6.66908E-4 96.56763 0.0011 122.92882 0.00111 80.93353 0.00182 96.96537

6.58552E-4 94.67518 0.0011 121.92246 0.00111 79.38944 0.00182 96.96576

6.50143E-4 92.81661 0.0011 120.93694 0.0011 77.92257 0.00182 95.82345

6.4166E-4 91.22972 0.0011 120.53456 0.00109 76.78941 0.00184 95.63639

6.33099E-4 89.50687 0.0011 119.90988 0.00108 75.70552 0.00184 95.1323

6.2449E-4 87.96238 0.0011 119.53496 0.00106 74.72113 0.00184 94.85319

6.15826E-4 86.48558 0.0011 118.82725 0.00105 73.85209 0.00184 94.20107

6.07068E-4 84.81387 0.0011 118.86885 0.00104 72.69024 0.00184 93.78894

6.00229E-4 83.25249 0.0011 117.98059 0.00104 71.5571 0.00184 93.07846

5.91344E-4 82.08976 0.0011 117.56415 0.00103 70.47613 0.00184 92.79254

5.82422E-4 80.70649 0.0011 117.7445 0.00102 69.96935 0.00184 92.51914

5.73455E-4 79.44194 0.0011 117.31432 0.00101 68.92671 0.00182 91.66961

5.64436E-4 78.44905 0.0011 117.3005 9.95979E-4 67.93139 0.00182 90.47893

5.55377E-4 77.27804 0.00109 117.05059 9.85283E-4 67.00235 0.00182 90.25678

5.46295E-4 76.21712 0.00109 116.56476 9.74522E-4 65.87933 0.00182 89.66662

5.3719E-4 74.78284 0.00109 116.10672 9.63703E-4 65.12472 0.00182 89.09513

5.28073E-4 73.78983 0.00109 115.57942 9.52851E-4 64.34562 0.0018 88.19577

5.20952E-4 72.68674 0.00109 115.07961 9.48896E-4 63.11073 0.0018 87.65287

5.11836E-4 71.27808 0.00109 114.34393 9.37981E-4 62.25716 0.0018 87.19746

5.02705E-4 70.59064 0.00109 114.21912 9.27613E-4 61.25505 0.00179 86.5095

4.93554E-4 69.75037 0.00108 113.48341 9.16884E-4 60.45437 0.00178 85.93089

4.84401E-4 69.02053 0.00108 112.1093 9.06086E-4 59.6028 0.00178 84.64842

4.75222E-4 68.42652 0.00108 110.84625 8.95227E-4 58.64548 0.00177 83.90209

4.68013E-4 67.38258 0.00108 109.84687 8.88528E-4 57.69348 0.00176 83.38831

4.58774E-4 66.78853 0.00108 109.31941 8.77569E-4 56.68735 0.00175 82.43443

4.49475E-4 65.78729 0.00108 107.70916 8.66557E-4 55.46082 0.00174 81.61929

4.40133E-4 64.86227 0.00108 105.69666 8.55519E-4 54.41209 0.00173 80.45518

4.30723E-4 64.21727 0.00107 104.58626 8.44464E-4 53.04505 0.00172 79.71488

4.23257E-4 63.10546 0.00107 103.39257 8.37589E-4 52.21079 0.0017 78.4407

4.13765E-4 62.39258 0.00107 102.32384 8.26521E-4 51.35792 0.00169 77.78964

4.04216E-4 61.43787 0.00107 100.0752 8.15438E-4 50.31766 0.00168 76.7653

3.94605E-4 60.27518 0.00107 98.88146 8.04338E-4 49.32695 0.00166 75.28546

3.84953E-4 59.0575 0.00106 97.13275 7.93238E-4 48.58647 0.00165 74.89382

3.77248E-4 57.89478 0.00106 95.38389 7.86323E-4 47.43033 0.00163 73.90383

3.67483E-4 56.24848 0.00106 92.74659 7.7518E-4 46.56016 0.00162 73.1409

3.57699E-4 54.967 0.00106 90.91436 7.63992E-4 45.64398 0.0016 72.47803

3.47907E-4 53.60065 0.00106 89.22098 7.52768E-4 44.80604 0.00159 71.2523

3.40126E-4 52.10701 0.00105 87.19472 7.45701E-4 43.50348 0.00158 70.05772

3.30381E-4 50.56253 0.00105 84.7378 7.34396E-4 42.97109 0.00157 68.99599

3.20661E-4 48.98397 0.00104 83.00259 7.23062E-4 42.61586 0.00156 68.21669

3.10981E-4 48.02507 0.00104 80.79579 7.11701E-4 41.77566 0.00155 67.34242

3.01353E-4 46.87083 0.00103 79.04693 7.00294E-4 41.13732 0.00154 67.00596

2.93777E-4 45.27539 0.00103 77.13148 6.93023E-4 40.38394 0.00153 65.89197

2.84255E-4 44.06186 0.00102 75.52147 6.81499E-4 39.46968 0.00152 64.99083

2.74772E-4 42.79737 0.00102 73.6615 6.69911E-4 39.01117 0.00151 64.1305

2.65321E-4 41.57527 0.00102 71.95433 6.58255E-4 38.14938 0.0015 63.54038

2.57908E-4 40.92189 0.00101 70.34418 6.5074E-4 37.54299 0.00149 62.29129

2.48544E-4 39.60643 1E-3 68.97004 6.38991E-4 36.96553 0.00148 61.642

2.3921E-4 38.68139 9.92E-4 67.84572 6.27205E-4 36.32919 0.00147 60.35466

-- -- 9.92E-4 66.92968 6.15407E-4 35.59139 0.00146 59.64838

-- -- 9.84E-4 65.72199 6.03584E-4 34.91155 0.00145 58.29214

-- -- 9.76E-4 65.22236 5.91733E-4 34.36708 0.00144 57.34303

-- -- 9.68E-4 63.25143 5.84081E-4 33.77335 0.00143 56.00755

-- -- 9.6E-4 62.50183 5.72243E-4 33.21689 0.00142 54.74243

-- -- 9.52E-4 61.43315 5.60414E-4 0.00141 53.30379

**Fig. 7 Relationship between the dynamic deformation modulus and repeated impact number**

1 65 1 55 1 51 1 57 1 78

3 56 3 43 2 47 3 50 3 71

5 50 5 41 4 36 5 40 5 58

7 52 6 37 5 32 7 29 7 53

8 49 -- 9 48

**Fig. 9 Variation curves of the macroscopic damage variables of the cracked rock masses with cracks’ inclination angles**

0 0.28 0 0.35 0 0.42

30 0.35 30 0.5 30 0.6

45 0.37 45 0.52 45 0.63

60 0.32 60 0.47 60 0.56

90 0.26 90 0.31 90 0.36

**Fig. 12 Comparison between experimental results and theoretical calculation results**

7.24055E-8 0.24615 5.75146E-8 0.18044 3.44424E-8 -0.20119 1.38187E-7 0.16124 8.5E-5 1.29992 1.1E-4 1.22385 1.25E-4 1.2055 1.81E-4 1.39663

2.04979E-7 0.23769 1.44608E-7 0.49966 8.57313E-8 -0.19306 3.96093E-7 0.23721 1.7E-4 2.59727 2.2E-4 2.44639 2.5E-4 2.41067 3.62E-4 2.79281

3.91134E-7 0.31406 2.82638E-7 0.48579 1.93239E-7 0.40142 7.46545E-7 -0.13616 2.55E-4 3.89165 3.3E-4 3.66721 3.75E-4 3.61531 5.43E-4 4.18847

6.70579E-7 0.19523 4.86398E-7 1.15201 4.00725E-7 0.32552 1.1766E-6 -0.31352 3.4E-4 5.18281 4.4E-4 4.88603 5E-4 4.81921 7.24E-4 5.58354

1.07576E-6 0.25463 7.19738E-7 1.81825 7.5814E-7 0.29832 1.70193E-6 -0.34076 4.25E-4 6.47056 5.5E-4 6.10262 6.25E-4 6.02219 9.05E-4 6.97792

1.56236E-6 0.16977 9.39928E-7 2.09597 1.23521E-6 0.71783 2.3396E-6 -0.48802 5.1E-4 7.75477 6.6E-4 7.31678 7.5E-4 7.22403 0.00109 8.37148

2.13432E-6 0.49222 1.23078E-6 1.90144 1.85007E-6 0.74667 3.01164E-6 -0.48157 5.95E-4 9.03531 7.7E-4 8.52834 8.75E-4 8.42454 0.00127 9.76409

2.80791E-6 0.65353 1.63994E-6 2.41499 2.64387E-6 1.03513 3.75701E-6 -0.17737 6.8E-4 10.31207 8.8E-4 9.73715 1E-3 9.62353 0.00145 11.15557

3.59139E-6 0.89103 2.12795E-6 3.06731 3.62181E-6 1.26272 4.60378E-6 0.35867 7.65E-4 11.58495 9.9E-4 10.94306 0.00112 10.82079 0.00163 12.54575

4.48438E-6 0.86559 2.72605E-6 3.52542 4.7651E-6 1.52404 5.59228E-6 0.86138 8.5E-4 12.85388 0.0011 12.14595 0.00125 12.01614 0.00181 13.93443

5.48584E-6 1.10333 3.36196E-6 4.38608 6.05054E-6 1.87521 6.74483E-6 1.07888 9.35E-4 14.11878 0.00121 13.34569 0.00137 13.20936 0.00199 15.32139

6.59678E-6 1.36636 3.97815E-6 4.78859 7.48805E-6 2.73426 8.05906E-6 1.30425 0.00102 15.37958 0.00132 14.54218 0.0015 14.40026 0.00217 16.70639

7.79808E-6 1.87547 4.59105E-6 4.94124 9.09673E-6 3.13371 9.55522E-6 1.47014 0.00111 16.63622 0.00143 15.7353 0.00162 15.58866 0.00235 18.08918

9.09568E-6 2.36771 5.32387E-6 5.8434 1.08931E-5 3.74482 1.12047E-5 1.24298 0.00119 17.88864 0.00154 16.92496 0.00175 16.77435 0.00253 19.4695

1.05233E-5 2.57991 6.29991E-6 6.20429 1.28887E-5 4.35959 1.29101E-5 1.98755 0.00128 19.13679 0.00165 18.11107 0.00187 17.95714 0.00272 20.84705

1.20759E-5 2.80055 7.40742E-6 6.39864 1.50559E-5 5.49899 1.46343E-5 2.25449 0.00136 20.38062 0.00176 19.29353 0.002 19.13684 0.0029 22.22154

1.37467E-5 3.06365 8.62504E-6 6.84275 1.74453E-5 6.16054 1.64148E-5 2.63538 0.00145 21.62009 0.00187 20.47226 0.00213 20.31325 0.00308 23.59265

1.55535E-5 3.6153 1.01006E-5 7.57856 2.015E-5 6.86813 1.82542E-5 3.31223 0.00153 22.85514 0.00198 21.64719 0.00225 21.48619 0.00326 24.96005

1.73651E-5 4.26877 1.17535E-5 8.21688 2.3107E-5 7.97431 2.019E-5 3.96542 0.00161 24.08575 0.00209 22.81823 0.00237 22.65547 0.00344 26.32338

1.91472E-5 5.88966 1.35544E-5 9.1886 2.63101E-5 9.06663 2.21464E-5 4.87769 0.0017 25.31187 0.0022 23.98531 0.0025 23.82089 0.00362 27.68229

2.10439E-5 7.4936 1.56379E-5 10.16007 2.98373E-5 10.12794 2.40802E-5 5.7018 0.00178 26.53348 0.00231 25.14836 0.00263 24.98227 0.0038 29.0364

2.308E-5 8.63932 1.79202E-5 11.07625 3.36509E-5 11.13469 2.60485E-5 6.18122 0.00187 27.75053 0.00242 26.30732 0.00275 26.13942 0.00398 30.38532

2.5269E-5 9.3351 2.05066E-5 12.00623 3.77347E-5 12.62303 2.80736E-5 6.82296 0.00196 28.96301 0.00253 27.46213 0.00288 27.29217 0.00416 31.72865

2.7678E-5 10.345 2.33837E-5 12.56134 4.21427E-5 13.98035 3.01974E-5 8.25226 0.00204 30.17088 0.00264 28.61271 0.003 28.44033 0.00434 33.06597

3.03384E-5 10.8033 2.65418E-5 13.29706 4.68688E-5 15.15502 3.24704E-5 9.62491 0.00213 31.37412 0.00275 29.75903 0.00313 29.58371 0.00453 34.39685

3.32664E-5 11.59253 3.0063E-5 14.57402 5.19231E-5 16.60769 3.49057E-5 10.17422 0.00221 32.57269 0.00286 30.90101 0.00325 30.72215 0.00471 35.72085

3.64891E-5 12.23747 3.39358E-5 15.11534 5.73472E-5 18.00297 3.75541E-5 10.75651 0.0023 33.76659 0.00297 32.03861 0.00338 31.85547 0.00489 37.03752

4.00258E-5 12.79759 3.82426E-5 16.35062 6.31597E-5 19.42991 4.04957E-5 11.76518 0.00238 34.95579 0.00308 33.17178 0.0035 32.98348 0.00507 38.3464

4.38779E-5 13.51911 4.28975E-5 17.33624 6.93438E-5 20.82191 4.37361E-5 12.78814 0.00247 36.14026 0.00319 34.30047 0.00363 34.10603 0.00525 39.647

4.80574E-5 14.57975 4.79354E-5 18.40492 7.59191E-5 22.44863 4.71544E-5 14.1214 0.00255 37.32 0.0033 35.42463 0.00375 35.22295 0.00543 40.93885

5.25721E-5 15.52174 5.34102E-5 19.82074 8.28719E-5 24.00644 5.08402E-5 15.71695 0.00264 38.49498 0.00341 36.54423 0.00388 36.33406 0.00561 42.22144

5.73952E-5 16.33655 5.92418E-5 20.90338 9.01642E-5 25.47045 5.49149E-5 16.39558 0.00272 39.66519 0.00352 37.65921 0.004 37.4392 0.00579 43.49429

6.25072E-5 17.76226 6.54709E-5 22.22188 9.78087E-5 27.18934 5.93232E-5 17.84638 0.00281 40.83062 0.00363 38.76954 0.00413 38.53823 0.00597 44.75688

6.79084E-5 19.15403 7.20943E-5 23.6515 1.05758E-4 28.68973 6.40419E-5 19.05179 0.00289 41.99125 0.00374 39.87519 0.00425 39.63097 0.00615 46.00869

7.3593E-5 20.69002 7.90743E-5 25.46978 1.13999E-4 30.54682 6.90117E-5 20.73192 0.00298 43.14707 0.00385 40.97611 0.00438 40.71728 0.00634 47.24919

7.95612E-5 22.65039 8.64704E-5 26.85786 1.22557E-4 32.39659 7.41969E-5 22.3862 0.00306 44.29806 0.00396 42.07228 0.0045 41.79701 0.00652 48.47787

8.57924E-5 24.29693 9.42336E-5 28.84274 1.31442E-4 34.20707 7.9578E-5 24.11823 0.00314 45.44423 0.00407 43.16366 0.00462 42.87001 0.0067 49.69418

9.22774E-5 26.47807 1.0229E-4 30.38347 1.40658E-4 35.6983 8.51947E-5 25.57554 0.00323 46.58556 0.00418 44.25023 0.00475 43.93615 0.00688 50.89759

9.90268E-5 28.54854 1.10688E-4 32.81249 1.50187E-4 37.56993 9.10292E-5 26.91952 0.00331 47.72205 0.00429 45.33195 0.00488 44.99528 0.00706 52.08756

1.0604E-4 30.82287 1.19502E-4 34.90833 1.60069E-4 39.14476 9.70892E-5 28.5337 0.0034 48.85368 0.0044 46.4088 0.005 46.04727 0.00724 53.26355

1.1339E-4 32.94459 1.28783E-4 38.16043 1.70349E-4 40.85816 1.03337E-4 29.8602 0.00348 49.98045 0.00451 47.48075 0.00513 47.092 0.00742 54.42502

1.21138E-4 35.59241 1.38463E-4 41.23769 1.81062E-4 43.06021 1.09835E-4 31.47651 0.00357 51.10236 0.00462 48.54779 0.00525 48.12934 0.0076 55.57143

1.29208E-4 38.01085 1.4841E-4 43.00034 1.92247E-4 44.66506 1.16714E-4 32.76185 0.00365 52.2194 0.00473 49.60988 0.00537 49.15917 0.00778 56.70225

1.37567E-4 40.03075 1.58593E-4 45.0269 2.03903E-4 46.82881 1.23857E-4 34.28784 0.00374 53.33157 0.00484 50.66702 0.0055 50.18138 0.00796 57.81695

1.46211E-4 41.76187 1.68934E-4 47.23386 2.15996E-4 48.65919 1.31235E-4 35.58246 0.00382 54.43887 0.00495 51.71918 0.00562 51.19587 0.00814 58.915

1.55073E-4 44.10423 1.79405E-4 49.19084 2.28448E-4 50.17514 1.389E-4 36.69535 0.00391 55.5413 0.00506 52.76635 0.00575 52.20253 0.00833 59.99589

1.64166E-4 46.5229 1.90039E-4 51.48117 2.4125E-4 52.17565 1.46814E-4 38.40986 0.004 56.63885 0.00517 53.80852 0.00588 53.20126 0.00851 61.05911

1.73481E-4 48.83113 2.00846E-4 53.09132 2.54401E-4 54.09893 1.54919E-4 39.51774 0.00408 57.73153 0.00528 54.84566 0.006 54.19198 0.00869 62.10416

1.82952E-4 51.58949 2.11922E-4 54.96494 2.67914E-4 56.2222 1.63268E-4 41.10114 0.00417 58.81933 0.00539 55.87778 0.00613 55.1746 0.00887 63.13056

1.92573E-4 54.69526 2.23274E-4 57.00545 2.8179E-4 58.17688 1.71814E-4 41.99868 0.00425 59.90226 0.0055 56.90485 0.00625 56.14904 0.00905 64.13785

2.0233E-4 57.43662 2.34828E-4 58.92075 2.95972E-4 60.2387 1.80512E-4 43.06466 0.00434 60.98032 0.00561 57.92687 0.00637 57.11523 0.00923 65.12557

2.12222E-4 60.43228 2.46624E-4 60.55864 3.1047E-4 62.2944 1.89374E-4 44.2876 0.00442 62.05352 0.00572 58.94384 0.0065 58.07311 0.00941 66.09331

2.22306E-4 63.18192 2.58606E-4 62.40467 3.2532E-4 64.1887 1.98301E-4 45.23068 0.00451 63.12185 0.00583 59.95575 0.00662 59.02263 0.00959 67.04064

2.32588E-4 65.77031 2.70765E-4 63.9316 3.40538E-4 66.18697 2.0738E-4 47.22094 0.00459 64.18532 0.00594 60.96259 0.00675 59.96372 0.00977 67.9672

2.43044E-4 68.14646 2.83069E-4 65.70805 3.56124E-4 68.14977 2.16731E-4 48.80875 0.00468 65.24394 0.00605 61.96437 0.00688 60.89635 0.00996 68.87262

2.53707E-4 70.55663 2.95487E-4 66.54087 3.72034E-4 70.04233 2.26332E-4 50.62934 0.00476 66.29771 0.00616 62.96107 0.007 61.82049 0.01014 69.75658

2.64577E-4 72.44059 3.08044E-4 68.37309 3.88216E-4 71.77003 2.36125E-4 52.07801 0.00485 67.34663 0.00627 63.95271 0.00713 62.7361 0.01032 70.61879

2.75615E-4 74.46889 3.207E-4 69.59473 4.04686E-4 73.79572 2.46098E-4 53.74309 0.00493 68.39072 0.00638 64.93927 0.00725 63.64317 0.0105 71.45898

2.86853E-4 76.68391 3.33546E-4 70.62173 4.2144E-4 75.57273 2.562E-4 55.18129 0.00501 69.42998 0.00649 65.92078 0.00737 64.54167 0.01068 72.27695

2.98303E-4 78.65269 3.46498E-4 72.03755 4.3847E-4 76.94384 2.66502E-4 56.50101 0.0051 70.46442 0.0066 66.89722 0.0075 65.43162 0.01086 73.0725

3.0997E-4 80.33301 3.59534E-4 72.55113 4.55755E-4 78.97691 2.77051E-4 57.96323 0.00518 71.49405 0.00671 67.8686 0.00762 66.31301 0.01104 73.84549

3.21863E-4 82.41233 3.72733E-4 73.73093 4.73273E-4 80.66626 2.8781E-4 59.49183 0.00527 72.51887 0.00682 68.83494 0.00775 67.18586 0.01122 74.59584

3.33959E-4 84.34708 3.85955E-4 75.54906 4.91006E-4 82.48103 2.98781E-4 60.85736 0.00535 73.53889 0.00693 69.79624 0.00787 68.05018 0.0114 75.32348

3.46217E-4 86.10382 3.9922E-4 76.45146 5.08893E-4 84.4133 3.09944E-4 62.16478 0.00544 74.55413 0.00704 70.7525 0.008 68.90601 0.01158 76.02843

3.58602E-4 88.04305 4.12518E-4 78.04752 5.26862E-4 86.24917 3.21288E-4 63.97824 0.00552 75.5646 0.00715 71.70374 0.00812 69.75337 0.01176 76.71074

3.71102E-4 90.10111 4.25807E-4 79.17184 5.44897E-4 87.8799 3.3273E-4 65.16494 0.00561 76.5703 0.00726 72.64997 0.00825 70.59233 0.01195 77.3705

3.8371E-4 92.01908 4.39191E-4 80.89295 5.62873E-4 89.5725 3.44284E-4 66.72828 0.00569 77.57124 0.00737 73.5912 0.00837 71.42292 0.01213 78.00788

3.964E-4 93.87741 4.52618E-4 82.82235 5.80762E-4 91.00775 3.55912E-4 67.83223 0.00578 78.56744 0.00748 74.52745 0.0085 72.24522 0.01231 78.6231

4.09151E-4 95.85491 4.66066E-4 84.1548 5.98654E-4 92.80692 3.67594E-4 69.20776 0.00586 79.5589 0.00759 75.45873 0.00862 73.05929 0.01249 79.21642

4.21926E-4 97.85758 4.79543E-4 86.29217 6.16549E-4 94.50302 3.79396E-4 70.42641 0.00595 80.54565 0.0077 76.38505 0.00875 73.86521 0.01267 79.78819

4.34669E-4 100.48011 4.92987E-4 87.87474 6.34419E-4 96.2496 3.91319E-4 70.84203 0.00603 81.52769 0.00781 77.30643 0.00887 74.66307 0.01285 80.33878

4.47368E-4 102.83076 5.06414E-4 89.23493 6.52283E-4 97.76755 4.03385E-4 71.53217 0.00612 82.50503 0.00792 78.22289 0.009 75.45296 0.01303 80.86866

4.60493E-4 105.06268 5.19864E-4 90.76186 6.71187E-4 99.3704 4.15612E-4 72.31682 0.0062 83.47769 0.00803 79.13445 0.00912 76.23499 0.01321 81.37832

4.73136E-4 107.69361 5.33171E-4 92.63562 6.89211E-4 100.95757 4.28282E-4 73.10121 0.00629 84.44568 0.00814 80.04112 0.00925 77.00927 0.01339 81.86835

4.85651E-4 110.03585 5.46223E-4 93.92639 7.07176E-4 102.39369 4.40744E-4 73.83714 0.00637 85.40902 0.00825 80.94293 0.00937 77.77592 0.01358 82.33937

4.98034E-4 112.64965 5.59089E-4 95.9666 7.25092E-4 104.22799 4.53373E-4 73.978 0.00646 86.36771 0.00836 81.8399 0.0095 78.53505 0.01376 82.79205

5.10281E-4 115.21243 5.71789E-4 98.15975 7.4296E-4 105.88449 4.66049E-4 74.91816 0.00654 87.32177 0.00847 82.73204 0.00962 79.28681 0.01394 83.22715

5.20359E-4 117.6312 5.84302E-4 99.92255 7.56573E-4 107.53474 4.78747E-4 75.99706 0.00663 88.27122 0.00858 83.61939 0.00975 80.03134 0.01412 83.64545

5.32278E-4 120.33831 5.96603E-4 101.31048 7.74298E-4 109.38542 4.89976E-4 76.48862 0.00671 89.21607 0.00869 84.50196 0.00987 80.76878 0.0143 84.04779

5.44032E-4 122.63827 6.08601E-4 102.50423 7.91921E-4 110.96619 5.02703E-4 77.59584 0.0068 90.15633 0.0088 85.37979 0.01 81.49929 0.01448 84.43506

5.55595E-4 124.9549 6.20245E-4 103.94779 8.09432E-4 112.76796 5.15459E-4 78.08922 0.00688 91.09201 0.00891 86.25288 0.01013 82.22303 0.01466 84.8082

5.66986E-4 127.18694 6.3161E-4 105.36361 8.26819E-4 113.77173 5.28146E-4 78.96672 0.00697 92.02314 0.00902 87.12128 0.01025 82.94015 0.01484 85.16818

5.78218E-4 129.19822 6.42721E-4 106.34908 8.4407E-4 115.37413 5.40698E-4 79.62853 0.00705 92.94973 0.00913 87.98499 0.01038 83.65085 0.01502 85.516

5.89272E-4 131.17561 6.53508E-4 107.04304 8.61201E-4 116.60574 5.53184E-4 80.2151 0.00714 93.87179 0.00924 88.84406 0.0105 84.35528 0.0152 85.85271

5.98162E-4 132.58427 6.63954E-4 108.94455 8.74008E-4 117.69781 5.65606E-4 81.25276 0.00722 94.78933 0.00935 89.69851 0.01063 85.05364 0.01538 86.17936

6.08911E-4 134.70576 6.74083E-4 110.76298 8.90839E-4 119.06275 5.76481E-4 81.48744 0.00731 95.70237 0.00946 90.54836 0.01075 85.74612 0.01557 86.49704

6.19509E-4 136.11477 6.8395E-4 111.74845 9.07521E-4 119.99593 5.88801E-4 81.46738 0.00739 96.61093 0.00957 91.39364 0.01087 86.43289 0.01575 86.80685

6.27949E-4 137.26877 6.93639E-4 112.81714 9.19885E-4 121.04815 6.01068E-4 82.1657 0.00748 97.51502 0.00968 92.23438 0.011 87.11416 0.01593 87.1099

6.38227E-4 138.33819 7.03221E-4 115.16984 9.36332E-4 122.06957 6.17546E-4 82.94222 0.00756 98.41465 0.00979 93.07061 0.01112 87.79012 0.01611 87.40729

6.48362E-4 139.62814 7.12651E-4 117.38384 9.52697E-4 122.97456 6.37641E-4 83.77343 0.00765 99.30984 0.0099 93.90235 0.01125 88.46098 0.01629 87.70013

6.5638E-4 140.63792 7.22048E-4 119.11875 9.64798E-4 124.34773 6.58112E-4 83.75555 0.00773 100.20061 0.01001 94.72964 0.01138 89.12693 0.01647 87.98951

6.64299E-4 142.0552 7.3146E-4 120.32629 9.76803E-4 125.53832 6.78838E-4 83.94726 0.00782 101.08696 0.01012 95.5525 0.0115 89.78819 0.01665 88.27652

6.74165E-4 143.24344 7.40755E-4 122.54719 9.92925E-4 126.51139 6.99761E-4 84.80214 0.0079 101.96891 0.01023 96.37096 0.01163 90.44496 0.01683 88.56222

6.81967E-4 144.67762 7.49983E-4 123.92117 0.00101 127.85233 7.2101E-4 85.6603 0.00799 102.84647 0.01034 97.18505 0.01175 91.09745 0.01701 88.84762

6.91689E-4 145.76393 7.59199E-4 125.55892 0.00102 129.02162 7.42552E-4 86.44253 0.00807 103.71966 0.01045 97.9948 0.01188 91.74586 0.01719 89.13374

6.99346E-4 146.72292 7.68446E-4 127.07206 0.00103 130.11711 7.64395E-4 87.32724 0.00816 104.58849 0.01056 98.80024 0.012 92.3904 0.01738 89.42153

7.0696E-4 148.22489 7.77657E-4 128.09921 0.00104 131.05622 7.86474E-4 88.14669 0.00824 105.45298 0.01067 99.60139 0.01213 93.03129 0.01756 89.7119

7.14495E-4 149.22214 7.86709E-4 128.55715 0.00105 132.12689 8.08657E-4 89.48354 0.00833 106.31313 0.01078 100.39829 0.01225 93.66873 0.01774 90.00572

7.21914E-4 150.20228 7.95641E-4 129.23717 0.00107 133.00038 8.3083E-4 90.61542 0.00841 107.16896 0.01089 101.19096 0.01238 94.30293 0.01792 90.30381

7.31219E-4 151.13575 8.08E-4 130.26432 0.00108 134.00946 8.53119E-4 91.50584 0.0085 108.02048 0.011 101.97943 0.0125 94.93409 0.0181 90.60693

7.38431E-4 152.28136 8.16E-4 131.44427 0.0011 134.60566 8.75466E-4 92.65813 0.00859 108.8677 0.01111 102.76373 0.01263 95.56241 0.01828 90.91576

7.45524E-4 153.29999 8.24E-4 132.37411 0.00111 135.24051 8.97737E-4 93.53098 0.00867 109.71062 0.01122 103.54387 0.01275 96.18809 0.01846 91.23095

7.52455E-4 154.41158 8.32E-4 133.69276 0.00112 135.83359 9.20079E-4 94.80305 0.00876 110.54927 0.01133 104.3199 0.01287 96.81133 0.01864 91.55306

7.59274E-4 155.31116 8.4E-4 134.40067 0.00113 136.23284 9.42401E-4 95.68408 0.00884 111.38365 0.01144 105.09183 0.013 97.43232 0.01882 91.88261

7.63975E-4 156.15132 8.48E-4 135.17801 0.00114 136.61134 9.64462E-4 97.02954 0.00893 112.21377 0.01155 105.85969 0.01313 98.05125 0.01901 92.22002

7.70547E-4 156.88978 8.56E-4 135.42797 0.00115 136.62759 9.8627E-4 97.5541 0.00901 113.03963 0.01166 106.62351 0.01325 98.6683 0.01919 92.56567

7.76977E-4 157.36485 8.64E-4 136.27444 0.00116 136.88777 0.00101 98.40228 0.0091 113.86124 0.01177 107.3833 0.01338 99.28366 0.01937 92.91986

7.83243E-4 158.0778 8.72E-4 136.62142 0.00117 136.93708 0.00103 99.01993 0.00918 114.67862 0.01188 108.13908 0.0135 99.89749 0.01955 93.28281

7.87382E-4 158.53586 8.8E-4 137.57915 0.00118 136.65302 0.00105 100.10064 0.00927 115.49176 0.01199 108.89089 0.01363 100.50997 0.01973 93.6547

7.93373E-4 158.65468 8.88E-4 138.95343 0.00119 136.67676 0.00107 100.93262 0.00935 116.30067 0.0121 109.63873 0.01375 101.12125 0.01991 94.03562

7.99194E-4 159.01116 8.96E-4 139.6336 0.0012 136.21318 0.00109 101.69155 0.00944 117.10535 0.01221 110.38264 0.01388 101.73149 0.02009 94.42561

8.02874E-4 158.57838 9.04E-4 140.09154 0.0012 136.49164 0.00111 102.60482 0.00952 117.9058 0.01232 111.12261 0.014 102.34085 0.02027 94.82463

8.08407E-4 158.56133 9.12E-4 141.27135 0.00121 136.13793 0.00113 103.53472 0.00961 118.70203 0.01243 111.85868 0.01413 102.94946 0.02045 95.2326

8.11781E-4 158.2815 9.2E-4 141.78477 0.00121 136.00955 0.00115 103.807 0.00969 119.49403 0.01254 112.59085 0.01425 103.55746 0.02063 95.64939

8.14982E-4 158.17113 9.28E-4 141.57679 0.00123 135.81021 0.00118 104.65837 0.00978 120.28181 0.01265 113.31913 0.01438 104.16497 0.02082 96.07477

8.20009E-4 157.49221 9.36E-4 142.32624 0.00124 135.50857 0.00119 105.45162 0.00986 121.06536 0.01276 114.04354 0.0145 104.77211 0.021 96.50851

8.22879E-4 157.35646 9.44E-4 143.43661 0.00124 135.26755 0.00121 105.81458 0.00995 121.84468 0.01287 114.76409 0.01463 105.379 0.02118 96.95031

8.2558E-4 156.67755 9.52E-4 143.93624 0.00125 135.29224 0.00123 106.39367 0.01003 122.61975 0.01298 115.48078 0.01475 105.98573 0.02136 97.39982

8.30102E-4 155.93918 9.6E-4 143.88072 0.00125 134.81023 0.00125 106.99345 0.01012 123.39058 0.01309 116.19361 0.01487 106.59241 0.02154 97.85666

8.32484E-4 155.48943 9.68E-4 144.76907 0.00126 134.71781 0.00127 108.15401 0.0102 124.15714 0.0132 116.9026 0.015 107.1991 0.02172 98.3204

8.34721E-4 154.42006 9.76E-4 145.21325 0.00126 134.18698 0.00129 108.89071 0.01029 124.91944 0.01331 117.60773 0.01513 107.80589 0.0219 98.79058

8.36772E-4 153.95333 9.84E-4 145.33822 0.00127 134.2714 0.00131 108.98178 0.01037 125.67746 0.01342 118.309 0.01525 108.41284 0.02208 99.26671

8.38627E-4 153.23198 9.92E-4 146.08774 0.00127 134.00886 0.00133 109.73059 0.01046 126.43118 0.01353 119.00641 0.01538 109.02002 0.02226 99.74828

8.40306E-4 152.78231 1E-3 146.82329 0.00127 133.84319 0.00134 110.16273 0.01054 127.18058 0.01364 119.69995 0.0155 109.62745 0.02244 100.23474

8.41812E-4 152.27305 0.00101 146.54579 0.00128 133.51847 0.00136 110.38126 0.01063 127.92564 0.01375 120.38961 0.01563 110.23519 0.02262 100.72551

8.43142E-4 151.75529 0.00101 146.25429 0.00128 133.20641 0.00138 110.91553 0.01071 128.66634 0.01386 121.07537 0.01575 110.84325 0.02281 101.22003

8.44321E-4 151.38191 0.00102 146.15702 0.00128 132.68193 0.0014 110.67909 0.0108 129.40265 0.01397 121.75721 0.01588 111.45166 0.02299 101.71767

8.45348E-4 150.66896 0.00102 146.79547 0.00128 131.93595 0.00141 110.88076 0.01088 130.13455 0.01408 122.4351 0.016 112.06042 0.02317 102.21782

8.44232E-4 150.13434 0.00103 146.46248 0.00128 131.47709 0.00143 111.38353 0.01097 130.86199 0.01419 123.10903 0.01613 112.66951 0.02335 102.71985

8.44985E-4 149.5404 0.00103 146.51793 0.00128 130.69948 0.00145 111.20037 0.01105 131.58496 0.0143 123.77895 0.01625 113.27893 0.02353 103.22312

8.45579E-4 148.60684 0.00104 146.62895 0.00129 129.91137 0.00146 111.20002 0.01114 132.30339 0.01441 124.44483 0.01638 113.88865 0.02371 103.72698

8.44039E-4 148.40308 0.00105 146.6012 0.00128 129.35379 0.00148 111.66119 0.01122 133.01726 0.01452 125.10663 0.0165 114.49863 0.02389 104.23075

8.44369E-4 147.63093 0.00105 146.85107 0.00129 128.25183 0.00149 111.27353 0.01131 133.72652 0.01463 125.7643 0.01663 115.10882 0.02407 104.73378

8.4454E-4 146.95204 0.00106 146.89267 0.00129 127.69321 0.00151 111.51108 0.01139 134.4311 0.01474 126.41779 0.01675 115.71916 0.02425 105.2354

8.42553E-4 146.10333 0.00106 146.35137 0.00129 126.91051 0.00152 111.73753 0.01148 135.13097 0.01485 127.06704 0.01688 116.32957 0.02443 105.73492

8.40406E-4 145.32252 0.00106 145.83778 0.00129 126.11501 0.00154 111.2305 0.01156 135.82605 0.01496 127.71199 0.017 116.93997 0.02462 106.23166

8.40118E-4 144.31271 0.00106 146.00446 0.00129 125.16216 0.00155 111.28887 0.01165 136.51627 0.01507 128.35255 0.01713 117.55026 0.0248 106.72494

8.37663E-4 143.34528 0.00106 145.50467 0.00129 124.25467 0.00157 111.32003 0.01173 137.20157 0.01518 128.98866 0.01725 118.16033 0.02498 107.21406

8.35038E-4 142.45423 0.00107 145.08836 0.00129 123.63441 0.00158 110.88977 0.01182 137.88187 0.01529 129.62022 0.01738 118.77006 0.02516 107.69832

8.34282E-4 141.27447 0.00107 144.60243 0.00129 122.27583 0.00159 111.06569 0.0119 138.55707 0.0154 130.24714 0.0175 119.37932 0.02534 108.17702

8.31392E-4 139.94221 0.00108 144.21388 0.00129 121.25487 0.0016 110.98293 0.01199 139.22709 0.01551 130.86931 0.01762 119.98794 0.02552 108.64945

8.28358E-4 138.65217 0.00108 143.58939 0.00128 120.15753 0.00162 110.05161 0.01207 139.89183 0.01562 131.48661 0.01775 120.59577 0.0257 109.11489

8.25205E-4 137.33674 0.00108 143.506 0.00128 118.90246 0.00163 109.75206 0.01215 140.55117 0.01573 132.09893 0.01787 121.20263 0.02588 109.57261

8.21951E-4 135.61413 0.00109 143.50596 0.00128 117.55215 0.00164 110.04269 0.01224 141.205 0.01584 132.70612 0.018 121.80832 0.02606 110.02187

8.18567E-4 133.94223 0.00109 142.49277 0.00128 116.12599 0.00165 109.36154 0.01232 141.85319 0.01595 133.30804 0.01812 122.41264 0.02625 110.46193

8.15003E-4 132.60982 0.00109 141.6322 0.00128 114.80525 0.00166 108.72766 0.01241 142.49561 0.01606 133.90452 0.01825 123.01535 0.02643 110.89201

8.11306E-4 131.07376 0.00109 141.06302 0.00128 113.46387 0.00167 108.40951 0.01249 143.13209 0.01617 134.4954 0.01837 123.61621 0.02661 111.31133

8.07511E-4 129.51223 0.0011 141.03533 0.00127 112.00098 0.00169 107.489 0.01258 143.76249 0.01628 135.08046 0.0185 124.21496 0.02679 111.7191

8.03608E-4 128.31572 0.0011 140.23026 0.00127 110.66928 0.0017 107.37719 0.01266 144.38663 0.01639 135.65952 0.01862 124.81132 0.02697 112.11448

7.97607E-4 127.35671 0.0011 139.34202 0.00126 109.41141 0.00171 107.18089 0.01275 145.00432 0.0165 136.23234 0.01875 125.40498 0.02715 112.49662

7.93525E-4 126.59285 0.0011 138.92554 0.00125 107.79388 0.00172 106.38502 0.01283 145.61535 0.01661 136.79868 0.01887 125.99562 0.02733 112.86464

7.89333E-4 125.19264 0.0011 138.81449 0.00125 106.19802 0.00173 105.74043 0.01292 146.2195 0.01672 137.35828 0.019 126.58289 0.02751 113.21764

7.83003E-4 124.08092 0.0011 138.37044 0.00124 104.79278 0.00173 105.66305 0.013 146.81654 0.01683 137.91084 0.01913 127.16643 0.02769 113.55466

7.78546E-4 123.15586 0.0011 137.50987 0.00124 103.37963 0.00174 104.90807 0.01309 147.40619 0.01694 138.45606 0.01925 127.74582 0.02787 113.87471

7.71956E-4 121.61986 0.0011 136.84356 0.00123 101.76012 0.00175 104.03632 0.01317 147.98819 0.01705 138.9936 0.01938 128.32064 0.02806 114.17675

7.67231E-4 120.55914 0.0011 136.59365 0.00123 100.41823 0.00176 104.19933 0.01326 148.56223 0.01716 139.52308 0.0195 128.89043 0.02824 114.45969

7.60387E-4 118.98067 0.0011 135.40011 0.00121 99.1094 0.00177 103.52393 0.01334 149.12797 0.01727 140.0441 0.01963 129.45469 0.02842 114.7224

7.55413E-4 117.47846 0.0011 133.91484 0.00121 97.41918 0.00177 102.46042 0.01343 149.68504 0.01738 140.55624 0.01975 130.01291 0.0286 114.96365

7.48313E-4 116.48536 0.0011 133.55392 0.0012 96.17594 0.00178 102.37878 0.01351 150.23307 0.01749 141.05901 0.01988 130.56449 0.02878 115.18217

7.41087E-4 115.0597 0.0011 132.85992 0.0012 94.56305 0.00178 101.98822 0.0136 150.77162 0.0176 141.5519 0.02 131.10884 0.02896 115.3766

7.33751E-4 113.41339 0.0011 131.68016 0.00119 93.01536 0.00179 101.58833 0.01368 151.30022 0.01771 142.03434 0.02013 131.64529 0.02914 115.54549

7.28317E-4 111.74157 0.0011 130.56986 0.00118 91.6133 0.00179 100.95373 0.01377 151.81837 0.01782 142.50573 0.02025 132.17312 0.02932 115.68731

7.20772E-4 109.91702 0.0011 129.79239 0.00117 90.17749 0.0018 100.08699 0.01385 152.3255 0.01793 142.96537 0.02038 132.69155 0.0295 115.80041

7.13118E-4 108.10098 0.0011 128.02971 0.00116 88.93755 0.0018 100.17843 0.01394 152.82101 0.01804 143.41254 0.0205 133.19976 0.02968 115.88302

7.05343E-4 0.0011 127.34955 0.00115 87.6237 0.0018 99.7559 0.01402 153.30422 0.01815 143.84644 0.02063 133.69682 0.02986 115.93326

6.97454E-4 0.0011 126.44746 0.00114 86.03993 0.00182 99.16975 0.01411 153.77441 0.01826 144.26616 0.02075 134.18175 0.03005 115.94909

6.91461E-4 0.0011 125.07331 0.00114 84.91268 0.00182 98.58637 0.01419 154.23076 0.01837 144.67074 0.02088 134.65346 0.03023 115.92832

6.83366E-4 0.0011 124.18487 0.00113 83.36013 0.00182 97.65762 0.01428 154.67238 0.01848 145.05911 0.021 135.11077 0.03041 115.86857

6.75186E-4 0.0011 123.71303 0.00112 82.24522 0.00182 97.1872 0.01436 155.09829 0.01859 145.43007 0.02113 135.55239 0.03059 115.7673

6.66908E-4 0.0011 122.92882 0.00111 80.93353 0.00182 96.96537 0.01445 155.5074 0.0187 145.78232 0.02125 135.97691 0.03077 115.62171

6.58552E-4 0.0011 121.92246 0.00111 0.00182 96.96576 0.01453 155.8985 0.01881 146.1144 0.02138 136.38274 0.03095 115.4288

6.50143E-4 0.0011 120.93694 0.0011 0.00182 95.82345 0.01462 156.27025 0.01892 146.4247 0.0215 136.76818 0.03113 115.18527

6.4166E-4 0.0011 120.53456 0.00109 0.00184 95.63639 0.0147 156.62114 0.01903 146.71143 0.02162 137.1313 0.03131 114.88755

6.33099E-4 0.0011 119.90988 0.00108 0.00184 95.1323 0.01479 156.94951 0.01914 146.97258 0.02175 137.46999 0.03149 114.53171

6.2449E-4 0.0011 119.53496 0.00106 0.00184 94.85319 0.01487 157.25347 0.01925 147.2059 0.02187 137.78188 0.03168 114.11348

6.15826E-4 0.0011 118.82725 0.00105 0.00184 94.20107 0.01496 157.53092 0.01936 147.40888 0.022 138.06432 0.03186 113.62813

6.07068E-4 0.0011 118.86885 0.00104 0.00184 93.78894 0.01504 157.77949 0.01947 147.57868 0.02212 138.31436 0.03204 113.0705

6.00229E-4 0.0011 117.98059 0.00104 0.00184 93.07846 0.01513 157.99646 0.01958 147.71209 0.02225 138.52864 0.03222 112.43489

5.91344E-4 0.0011 117.56415 0.00103 0.00184 92.79254 0.01521 158.17879 0.01969 147.80547 0.02237 138.7034 0.0324 111.715

5.82422E-4 0.0011 117.7445 0.00102 0.00184 92.51914 0.0153 158.32299 0.0198 147.85469 0.0225 138.83435 0.03258 110.90386

5.73455E-4 0.0011 117.31432 0.00101 0.00182 91.66961 0.01538 158.42505 0.01991 147.85501 0.02262 138.91661 0.03276 109.99374

5.64436E-4 0.0011 117.3005 9.95979E-4 0.00182 90.47893 0.01547 158.48037 0.02002 147.801 0.02275 138.94457 0.03294 108.97606

5.55377E-4 0.00109 117.05059 9.85283E-4 0.00182 90.25678 0.01555 158.48364 0.02013 147.68639 0.02288 138.91179 0.03312 107.84126

5.46295E-4 0.00109 116.56476 9.74522E-4 0.00182 89.66662 0.01564 158.42867 0.02024 147.5039 0.023 138.81082 0.0333 106.57865

5.3719E-4 0.00109 116.10672 9.63703E-4 0.00182 89.09513 0.01572 158.30824 0.02035 147.24507 0.02313 138.63298 0.03349 105.17629

5.28073E-4 0.00109 115.57942 9.52851E-4 0.0018 88.19577 0.01581 158.11382 0.02046 146.89994 0.02325 138.36808 0.03367 103.6208

5.20952E-4 0.00109 115.07961 9.48896E-4 0.0018 87.65287 0.01589 157.83536 0.02057 146.45677 0.02338 138.00415 0.03385 101.89715

5.11836E-4 0.00109 114.34393 9.37981E-4 0.0018 87.19746 0.01598 157.46084 0.02068 145.90163 0.0235 137.52695 0.03403 99.98845

5.02705E-4 0.00109 114.21912 9.27613E-4 0.00179 86.5095 0.01606 156.97581 0.02079 145.21778 0.02363 136.91945 0.03421 97.87568

4.93554E-4 0.00108 113.48341 9.16884E-4 0.00178 85.93089 0.01615 156.36275 0.0209 144.38504 0.02375 136.1611 0.03439 95.53742

4.84401E-4 0.00108 112.1093 9.06086E-4 0.00178 84.64842 0.01623 155.6002 0.02101 143.37881 0.02388 135.22687 0.03457 92.94956

4.75222E-4 0.00108 110.84625 8.95227E-4 0.00177 83.90209 0.01632 154.66162 0.02112 142.16881 0.024 134.086 0.03475 90.08498

4.68013E-4 0.00108 109.84687 8.88528E-4 0.00176 83.38831 0.0164 153.51376 0.02123 140.71736 0.02413 132.70021 0.03493 86.91334

4.58774E-4 0.00108 109.31941 8.77569E-4 0.00175 82.43443 0.01649 152.11445 0.02134 138.97699 0.02425 131.02135 0.03511 83.40082

4.49475E-4 0.00108 107.70916 8.66557E-4 0.00174 81.61929 0.01657 150.40946 0.02145 136.8871 0.02438 128.98798 0.0353 79.51022

4.40133E-4 0.00108 105.69666 8.55519E-4 0.00173 80.45518 0.01666 148.3278 0.02156 134.36921 0.0245 126.52059 0.03548 75.20129

4.30723E-4 0.00107 104.58626 8.44464E-4 0.00172 79.71488 0.01674 145.77495 0.02167 131.3199 0.02463 123.51453 0.03566 70.43186

4.23257E-4 0.00107 103.39257 8.37589E-4 0.0017 78.4407 0.01683 142.62232 0.02178 127.60046 0.02475 119.82955 0.03584 65.16002

4.13765E-4 0.00107 102.32384 8.26521E-4 0.00169 77.78964 0.01691 138.69086 0.02189 123.02102 0.02488 115.27393 0.03602 59.34841

4.04216E-4 0.00107 100.0752 8.15438E-4 0.00168 76.7653

3.94605E-4 0.00107 98.88146 8.04338E-4 0.00166 75.28546

3.84953E-4 0.00106 7.93238E-4 0.00165 74.89382

3.77248E-4 0.00106 7.86323E-4 0.00163 73.90383

3.67483E-4 0.00106 7.7518E-4 0.00162 73.1409

3.57699E-4 0.00106 7.63992E-4 0.0016 72.47803

0.00106 7.52768E-4 0.00159 71.2523

0.00105 7.45701E-4 0.00158 70.05772

0.00105 7.34396E-4 0.00157 68.99599

0.00104 7.23062E-4 0.00156 68.21669

0.00104 7.11701E-4 0.00155 67.34242

0.00103 7.00294E-4 0.00154 67.00596

0.00103 6.93023E-4 0.00153 65.89197

0.00102 6.81499E-4 0.00152 64.99083

0.00102 6.69911E-4 0.00151 64.1305

0.00102 6.58255E-4 0.0015 63.54038

0.00101 6.5074E-4 0.00149 62.29129

1E-3 6.38991E-4 0.00148 61.642

9.92E-4 6.27205E-4 0.00147 60.35466

9.92E-4 6.15407E-4 0.00146 59.64838

9.84E-4 6.03584E-4 0.00145 58.29214

9.76E-4 5.91733E-4 0.00144 57.34303

9.68E-4 5.84081E-4 0.00143 56.00755

9.6E-4 5.72243E-4 0.00142 54.74243

9.52E-4 5.60414E-4 0.00141 53.30379

**Fig. 13 Damage variable and strain relationship curves**

1.81E-4 6.33936E-6 1.81E-4 0.35002

3.62E-4 3.58603E-5 3.62E-4 0.35004

5.43E-4 9.88163E-5 5.43E-4 0.35009

7.24E-4 2.0284E-4 7.24E-4 0.35015

9.05E-4 3.54319E-4 9.05E-4 0.35024

0.00109 5.58861E-4 0.00109 0.35035

0.00127 8.21511E-4 0.00127 0.35048

0.00145 0.00115 0.00145 0.35065

0.00163 0.00154 0.00163 0.35085

0.00181 0.002 0.00181 0.35107

0.00199 0.00254 0.00199 0.35134

0.00217 0.00316 0.00217 0.35163

0.00235 0.00386 0.00235 0.35196

0.00253 0.00464 0.00253 0.35233

0.00272 0.00551 0.00272 0.35274

0.0029 0.00647 0.0029 0.35319

0.00308 0.00753 0.00308 0.35368

0.00326 0.00868 0.00326 0.35421

0.00344 0.00993 0.00344 0.35478

0.00362 0.01128 0.00362 0.3554

0.0038 0.01273 0.0038 0.35607

0.00398 0.01429 0.00398 0.35678

0.00416 0.01595 0.00416 0.35754

0.00434 0.01773 0.00434 0.35834

0.00453 0.01962 0.00453 0.3592

0.00471 0.02161 0.00471 0.36011

0.00489 0.02373 0.00489 0.36107

0.00507 0.02596 0.00507 0.36208

0.00525 0.0283 0.00525 0.36314

0.00543 0.03077 0.00543 0.36426

0.00561 0.03335 0.00561 0.36543

0.00579 0.03606 0.00579 0.36665

0.00597 0.03888 0.00597 0.36794

0.00615 0.04183 0.00615 0.36927

0.00634 0.0449 0.00634 0.37067

0.00652 0.0481 0.00652 0.37212

0.0067 0.05142 0.0067 0.37364

0.00688 0.05487 0.00688 0.37521

0.00706 0.05844 0.00706 0.37684

0.00724 0.06214 0.00724 0.37853

0.00742 0.06596 0.00742 0.38028

0.0076 0.06991 0.0076 0.38209

0.00778 0.07398 0.00778 0.38396

0.00796 0.07818 0.00796 0.3859

0.00814 0.08251 0.00814 0.3879

0.00833 0.08696 0.00833 0.38996

0.00851 0.09154 0.00851 0.39208

0.00869 0.09624 0.00869 0.39427

0.00887 0.10107 0.00887 0.39652

0.00905 0.10601 0.00905 0.39883

0.00923 0.11108 0.00923 0.40121

0.00941 0.11628 0.00941 0.40365

0.00959 0.12159 0.00959 0.40616

0.00977 0.12702 0.00977 0.40874

0.00996 0.13257 0.00996 0.41137

0.01014 0.13823 0.01014 0.41408

0.01032 0.14401 0.01032 0.41684

0.0105 0.14991 0.0105 0.41968

0.01068 0.15592 0.01068 0.42257

0.01086 0.16203 0.01086 0.42554

0.01104 0.16826 0.01104 0.42857

0.01122 0.17459 0.01122 0.43166

0.0114 0.18103 0.0114 0.43482

0.01158 0.18757 0.01158 0.43804

0.01176 0.19422 0.01176 0.44133

0.01195 0.20096 0.01195 0.44468

0.01213 0.20779 0.01213 0.44809

0.01231 0.21473 0.01231 0.45157

0.01249 0.22175 0.01249 0.45512

0.01267 0.22886 0.01267 0.45872

0.01285 0.23607 0.01285 0.46239

0.01303 0.24335 0.01303 0.46612

0.01321 0.25072 0.01321 0.46991

0.01339 0.25816 0.01339 0.47376

0.01358 0.26568 0.01358 0.47767

0.01376 0.27328 0.01376 0.48164

0.01394 0.28095 0.01394 0.48567

0.01412 0.28868 0.01412 0.48976

0.0143 0.29648 0.0143 0.49391

0.01448 0.30434 0.01448 0.49811

0.01466 0.31225 0.01466 0.50237

0.01484 0.32023 0.01484 0.50669

0.01502 0.32825 0.01502 0.51106

0.0152 0.33633 0.0152 0.51548

0.01538 0.34445 0.01538 0.51995

0.01557 0.35261 0.01557 0.52448

0.01575 0.36081 0.01575 0.52905

0.01593 0.36905 0.01593 0.53367

0.01611 0.37732 0.01611 0.53834

0.01629 0.38562 0.01629 0.54306

0.01647 0.39394 0.01647 0.54782

0.01665 0.40229 0.01665 0.55263

0.01683 0.41066 0.01683 0.55748

0.01701 0.41905 0.01701 0.56237

0.01719 0.42744 0.01719 0.56729

0.01738 0.43585 0.01738 0.57226

0.01756 0.44426 0.01756 0.57726

0.01774 0.45268 0.01774 0.5823

0.01792 0.46109 0.01792 0.58737

0.0181 0.4695 0.0181 0.59247

0.01828 0.4779 0.01828 0.5976

0.01846 0.4863 0.01846 0.60276

0.01864 0.49468 0.01864 0.60795

0.01882 0.50304 0.01882 0.61316

0.01901 0.51138 0.01901 0.61839

0.01919 0.5197 0.01919 0.62365

0.01937 0.528 0.01937 0.62892

0.01955 0.53626 0.01955 0.63421

0.01973 0.54449 0.01973 0.63952

0.01991 0.55269 0.01991 0.64483

0.02009 0.56085 0.02009 0.65016

0.02027 0.56897 0.02027 0.6555

0.02045 0.57704 0.02045 0.66085

0.02063 0.58507 0.02063 0.6662

0.02082 0.59305 0.02082 0.67155

0.021 0.60098 0.021 0.6769

0.02118 0.60885 0.02118 0.68225

0.02136 0.61667 0.02136 0.6876

0.02154 0.62442 0.02154 0.69294

0.02172 0.63212 0.02172 0.69828

0.0219 0.63975 0.0219 0.70361

0.02208 0.64732 0.02208 0.70892

0.02226 0.65482 0.02226 0.71422

0.02244 0.66224 0.02244 0.7195

0.02262 0.6696 0.02262 0.72477

0.02281 0.67688 0.02281 0.73001

0.02299 0.68408 0.02299 0.73523

0.02317 0.69121 0.02317 0.74043

0.02335 0.69826 0.02335 0.7456

0.02353 0.70522 0.02353 0.75074

0.02371 0.71211 0.02371 0.75586

0.02389 0.7189 0.02389 0.76094

0.02407 0.72562 0.02407 0.76598

0.02425 0.73224 0.02425 0.77099

0.02443 0.73878 0.02443 0.77596

0.02462 0.74523 0.02462 0.7809

0.0248 0.75159 0.0248 0.78579

0.02498 0.75786 0.02498 0.79063

0.02516 0.76403 0.02516 0.79544

0.02534 0.77011 0.02534 0.80019

0.02552 0.7761 0.02552 0.8049

0.0257 0.782 0.0257 0.80956

0.02588 0.78779 0.02588 0.81416

0.02606 0.7935 0.02606 0.81871

0.02625 0.7991 0.02625 0.82321

0.02643 0.80462 0.02643 0.82766

0.02661 0.81003 0.02661 0.83205

0.02679 0.81535 0.02679 0.83638

0.02697 0.82057 0.02697 0.84065

0.02715 0.82569 0.02715 0.84486

0.02733 0.83072 0.02733 0.84901

0.02751 0.83565 0.02751 0.8531

0.02769 0.84048 0.02769 0.85712

0.02787 0.84522 0.02787 0.86109

0.02806 0.84986 0.02806 0.86499

0.02824 0.8544 0.02824 0.86882

0.02842 0.85885 0.02842 0.87259

0.0286 0.8632 0.0286 0.87629

0.02878 0.86746 0.02878 0.87993

0.02896 0.87163 0.02896 0.88349

0.02914 0.8757 0.02914 0.887

0.02932 0.87967 0.02932 0.89043

0.0295 0.88356 0.0295 0.8938

0.02968 0.88736 0.02968 0.8971

0.02986 0.89106 0.02986 0.90033

0.03005 0.89467 0.03005 0.90349

0.03023 0.8982 0.03023 0.90659

0.03041 0.90164 0.03041 0.90961

0.03059 0.90499 0.03059 0.91257

0.03077 0.90825 0.03077 0.91547

0.03095 0.91144 0.03095 0.91829

0.03113 0.91453 0.03113 0.92105

0.03131 0.91755 0.03131 0.92375

0.03149 0.92048 0.03149 0.92637

0.03168 0.92333 0.03168 0.92893

0.03186 0.92611 0.03186 0.93143

0.03204 0.9288 0.03204 0.93386

0.03222 0.93142 0.03222 0.93623

0.0324 0.93396 0.0324 0.93854

0.03258 0.93643 0.03258 0.94078

0.03276 0.93883 0.03276 0.94296

0.03294 0.94116 0.03294 0.94508

0.03312 0.94341 0.03312 0.94715

0.0333 0.9456 0.0333 0.94915

0.03349 0.94772 0.03349 0.95109

0.03367 0.94977 0.03367 0.95298

0.03385 0.95175 0.03385 0.95481

0.03403 0.95368 0.03403 0.95658

0.03421 0.95554 0.03421 0.9583

0.03439 0.95734 0.03439 0.95996

0.03457 0.95908 0.03457 0.96158

0.03475 0.96076 0.03475 0.96314

0.03493 0.96239 0.03493 0.96465

0.03511 0.96396 0.03511 0.96611

0.0353 0.96548 0.0353 0.96752

0.03548 0.96694 0.03548 0.96888

0.03566 0.96835 0.03566 0.9702

0.03584 0.96972 0.03584 0.97147

0.03602 0.97103 0.03602

**Fig. 14 Comparison between experimental results and theoretical calculation results of different cracked rock masses**

**(a) *β*=0°**

6.77753E-8 0.37507 -7.01919E-8 -0.03795 -1.32913E-9 0.15171 -3.93366E-8 0.05119 1.49647E-7 0.30505 8.2E-5 2.49947 9.2E-5 2.32422 1.1E-4 2.24126 1.3E-4 2.17126 1.7E-4 1.98257

1.95878E-7 1.22849 1.94979E-8 0.24666 1.18457E-7 0.66382 7.58751E-8 0.40972 4.32573E-7 1.00898 1.64E-4 4.9914 1.84E-4 4.6446 2.2E-4 4.48022 2.6E-4 4.34059 3.4E-4 3.96338

3.73137E-7 1.70684 3.05465E-7 0.42691 3.73231E-7 0.96018 3.73283E-7 0.6242 8.1604E-7 1.31405 2.46E-4 7.47253 2.76E-4 6.95885 3.3E-4 6.71529 3.9E-4 6.50705 5.1E-4 5.94188

6.20405E-7 1.67423 9.00797E-7 0.88228 8.36661E-7 1.15043 9.55602E-7 0.91472 1.26263E-6 1.3845 3.28E-4 9.94047 3.68E-4 9.26501 4.4E-4 8.94496 5.2E-4 8.66947 6.8E-4 7.91734

9.74922E-7 2.4135 1.7353E-6 0.33205 1.49062E-6 1.2355 1.77426E-6 0.70539 1.70806E-6 1.50182 4.1E-4 12.39323 4.6E-4 11.56136 5.5E-4 11.16777 6.5E-4 10.82667 8.5E-4 9.88896

1.41286E-6 2.84836 2.601E-6 1.29972 2.20762E-6 1.86663 2.64474E-6 1.42485 2.12894E-6 1.65424 4.92E-4 14.82909 5.52E-4 13.84631 6.6E-4 13.38231 7.8E-4 12.97745 0.00102 11.85586

1.87909E-6 3.41369 3.5161E-6 2.24841 2.96735E-6 2.54795 3.5659E-6 2.15836 2.58839E-6 1.65439 5.74E-4 17.2465 6.44E-4 16.11837 7.7E-4 15.5872 9.1E-4 15.12058 0.00119 13.81707

2.42725E-6 4.09859 4.57288E-6 3.10224 3.85007E-6 3.24037 4.63262E-6 2.85417 3.11333E-6 2.11182 6.56E-4 19.64409 7.36E-4 18.37615 8.8E-4 17.78109 0.00104 17.25483 0.00136 15.77157

3.1348E-6 4.14208 5.70895E-6 4.13631 4.86406E-6 3.72528 5.81516E-6 3.53771 3.69554E-6 2.75718 7.38E-4 22.02058 8.28E-4 20.61832 9.9E-4 19.96268 0.00117 19.37896 0.00153 17.71827

3.97044E-6 5.06616 6.89571E-6 5.08501 5.97638E-6 4.56802 7.07965E-6 4.34387 4.35258E-6 3.25001 8.2E-4 24.37481 9.2E-4 22.84362 0.0011 22.13067 0.0013 21.4917 0.0017 19.65606

4.84184E-6 5.81631 8.18776E-6 5.8914 7.16628E-6 5.26847 8.44472E-6 5.02195 5.03535E-6 3.41416 9.02E-4 26.7057 0.00101 25.05087 0.00121 24.2838 0.00143 23.59181 0.00187 21.58374

5.75495E-6 6.30553 9.56431E-6 7.16265 8.42559E-6 6.06068 9.89445E-6 5.9505 5.73566E-6 3.57839 9.84E-4 29.01226 0.0011 27.23891 0.00132 26.42085 0.00156 25.67801 0.00204 23.50012

6.76487E-6 7.33833 1.09577E-5 8.69005 9.74741E-6 7.21277 1.13878E-5 7.15626 6.46518E-6 3.82482 0.00107 31.29357 0.0012 29.40665 0.00143 28.54062 0.00169 27.74906 0.00221 25.40393

7.94163E-6 8.31678 1.23746E-5 9.9613 1.11739E-5 8.22514 1.29517E-5 8.18389 7.24965E-6 3.91872 0.00115 33.54875 0.00129 31.55305 0.00154 30.64191 0.00182 29.80371 0.00238 27.2939

9.36864E-6 8.36026 1.37784E-5 12.0674 1.27309E-5 9.19245 1.45801E-5 9.56693 8.15216E-6 4.79872 0.00123 35.77702 0.00138 33.67711 0.00165 32.72359 0.00195 31.84069 0.00255 29.16871

1.09625E-5 8.66467 1.51953E-5 13.73711 1.43868E-5 10.08081 1.62701E-5 10.71806 9.11784E-6 5.54957 0.00131 37.97763 0.00147 35.77788 0.00176 34.78454 0.00208 33.85878 0.00272 31.02704

1.2595E-5 9.96925 1.67811E-5 16.10885 1.61569E-5 11.73515 1.81159E-5 12.52979 1.00754E-5 6.3943 0.00139 40.14988 0.00156 37.85445 0.00187 36.82366 0.00221 35.85675 0.00289 32.86754

1.42276E-5 10.59981 1.85333E-5 18.04419 1.80185E-5 12.8898 2.01035E-5 13.92029 1.11066E-5 7.2742 0.00148 42.29315 0.00166 39.90595 0.00198 38.8399 0.00234 37.83339 0.00306 34.68883

1.59093E-5 12.03486 2.04051E-5 20.26414 1.99729E-5 14.53455 2.22079E-5 15.65941 1.2216E-5 8.14247 0.00156 44.40683 0.00175 41.93155 0.00209 40.83222 0.00247 39.7875 0.00323 36.48953

1.7603E-5 13.1329 2.23847E-5 22.46511 2.19932E-5 16.0191 2.44079E-5 17.3179 1.34553E-5 8.90514 0.00164 46.4904 0.00184 43.93048 0.0022 42.79964 0.0026 41.71792 0.0034 38.26827

1.93115E-5 13.88303 2.44736E-5 24.90326 2.40818E-5 17.45383 2.67055E-5 19.06069 1.49143E-5 8.97555 0.00172 48.54334 0.00193 45.90199 0.00231 44.7412 0.00273 43.62348 0.00357 40.02366

2.11407E-5 15.32896 2.66898E-5 27.79678 2.63068E-5 19.40658 2.91481E-5 21.24152 1.6481E-5 10.75879 0.0018 50.5652 0.00202 47.84538 0.00242 46.65596 0.00286 45.50306 0.00374 41.75431

2.31263E-5 16.32915 2.9092E-5 30.40569 2.87201E-5 21.03068 3.17966E-5 23.14637 1.81703E-5 11.83825 0.00189 52.55558 0.00212 49.76 0.00253 48.54305 0.00299 47.35557 0.00391 43.45884

2.52698E-5 17.29672 3.17567E-5 33.00511 3.13646E-5 22.63583 3.47167E-5 25.03842 2.00666E-5 13.25782 0.00197 54.51411 0.00221 51.64522 0.00264 50.40161 0.00312 49.17993 0.00408 45.13589

2.74431E-5 18.63393 3.47502E-5 35.69941 3.42063E-5 24.45 3.79261E-5 27.06724 2.21815E-5 14.73614 0.00205 56.44046 0.0023 53.50048 0.00275 52.23084 0.00325 50.97511 0.00425 46.7841

2.96789E-5 19.99287 3.80116E-5 37.92884 3.72298E-5 26.06477 4.13828E-5 28.79713 2.4494E-5 15.82745 0.00213 58.33433 0.00239 55.32523 0.00286 54.02996 0.00338 52.74013 0.00442 48.40217

3.19877E-5 21.35183 4.14783E-5 40.86031 4.04063E-5 27.99546 4.50365E-5 30.98509 2.69994E-5 17.46976 0.00221 60.19548 0.00248 57.11899 0.00297 55.79826 0.00351 54.47402 0.00459 49.98877

3.42981E-5 23.00431 4.50983E-5 43.96254 4.3668E-5 30.13509 4.88215E-5 33.34393 2.97598E-5 19.52317 0.0023 62.02371 0.00258 58.8813 0.00308 57.53505 0.00364 56.17587 0.00476 51.54266

3.67261E-5 24.31977 4.8877E-5 46.02122 4.70817E-5 31.65345 5.27773E-5 34.9536 3.28486E-5 20.63761 0.00238 63.81884 0.00267 60.61177 0.00319 59.2397 0.00377 57.84482 0.00493 53.0626

3.93388E-5 25.64611 5.28741E-5 49.12345 5.07171E-5 33.64631 5.69752E-5 37.24639 3.6218E-5 22.11584 0.00246 65.58073 0.00276 62.31002 0.0033 60.91163 0.0039 59.48006 0.0051 54.54742

4.21242E-5 27.38557 5.71194E-5 51.29596 5.4584E-5 35.40669 6.14369E-5 39.01619 3.98375E-5 23.89935 0.00254 67.30928 0.00285 63.97573 0.00341 62.55029 0.00403 61.08082 0.00527 55.996

4.50438E-5 29.19025 6.16207E-5 53.97128 5.86655E-5 37.42269 6.61574E-5 41.12729 4.3788E-5 26.05821 0.00262 69.00445 0.00294 65.60864 0.00352 64.1552 0.00416 62.64641 0.00544 57.40726

4.80929E-5 30.66879 6.63782E-5 56.67507 6.29591E-5 39.30474 7.11355E-5 43.19092 4.80729E-5 28.08778 0.00271 70.66619 0.00304 67.20849 0.00363 65.72594 0.00429 64.17617 0.00561 58.78021

5.13089E-5 32.61481 7.14034E-5 59.10373 6.74918E-5 41.27334 7.63923E-5 45.16968 5.26172E-5 30.7043 0.00279 72.29452 0.00313 68.77511 0.00374 67.2621 0.00442 65.66953 0.00578 60.11393

5.46873E-5 34.06073 7.67094E-5 61.36163 7.22682E-5 42.94005 8.19377E-5 46.93576 5.74422E-5 32.38209 0.00287 73.88949 0.00322 70.30835 0.00385 68.76338 0.00455 67.12597 0.00595 61.40755

5.81654E-5 36.03936 8.23989E-5 63.41081 7.73104E-5 44.75258 8.78401E-5 48.67353 6.25828E-5 35.42078 0.00295 75.45117 0.00331 71.8081 0.00396 70.2295 0.00468 68.54505 0.00612 62.66032

6.18089E-5 38.3985 8.84367E-5 65.56435 8.26351E-5 46.78329 9.40895E-5 50.55643 6.80799E-5 37.99026 0.00303 76.97967 0.0034 73.2743 0.00407 71.66025 0.00481 69.92638 0.00629 63.87157

6.56445E-5 40.11621 9.49281E-5 67.88865 8.83149E-5 48.60219 1.00784E-4 52.42088 7.39278E-5 39.82053 0.00312 78.47514 0.0035 74.70695 0.00418 73.05548 0.00494 71.26967 0.00646 65.04074

6.95919E-5 41.88829 1.01953E-4 68.66658 9.43497E-5 49.74969 1.07966E-4 53.28732 8.0096E-5 41.95587 0.0032 79.93775 0.00359 76.10606 0.00429 74.41508 0.00507 72.5747 0.00663 66.16735

7.36718E-5 44.08435 1.09459E-4 70.41218 1.00722E-4 51.52344 1.156E-4 54.87103 8.66456E-5 43.95038 0.00328 81.3677 0.00368 77.47172 0.0044 75.73903 0.0052 73.84132 0.0068 67.25105

7.79409E-5 46.20431 1.17473E-4 72.46856 1.07478E-4 53.40279 1.23723E-4 56.64211 9.35256E-5 45.40513 0.00336 82.76525 0.00377 78.80403 0.00451 77.02734 0.00533 75.06947 0.00697 68.29162

8.24081E-5 48.53084 1.25664E-4 73.91286 1.1444E-4 55.09967 1.32057E-4 58.05564 1.00723E-4 47.55227 0.00344 84.13065 0.00386 80.10315 0.00462 78.28011 0.00546 76.25917 0.00714 69.28895

8.70526E-5 50.97695 1.33872E-4 74.03619 1.21509E-4 56.25591 1.40459E-4 58.63145 1.08278E-4 49.2184 0.00353 85.4642 0.00396 81.36929 0.00473 79.49748 0.00559 77.41052 0.00731 70.24304

9.18803E-5 52.97732 1.4234E-4 74.42515 1.28821E-4 57.33111 1.49139E-4 59.29032 1.16185E-4 50.83757 0.00361 86.76622 0.00405 82.60269 0.00484 80.67965 0.00572 78.5237 0.00748 71.15407

9.69225E-5 55.29298 1.51266E-4 75.94306 1.36504E-4 59.05621 1.58273E-4 60.74968 1.24451E-4 52.3745 0.00369 88.03708 0.00414 83.80363 0.00495 81.82689 0.00585 79.59901 0.00765 72.0223

1.02335E-4 57.21724 1.60771E-4 77.812 1.44708E-4 60.76317 1.68014E-4 62.35882 1.33026E-4 53.67679 0.00377 89.27714 0.00423 84.97244 0.00506 82.93953 0.00598 80.63679 0.00782 72.84819

1.08179E-4 60.14556 1.70919E-4 80.00348 1.53504E-4 63.06706 1.78433E-4 64.38175 1.41939E-4 55.67115 0.00385 90.4868 0.00432 86.10948 0.00517 84.01797 0.00611 81.6375 0.00799 73.63228

1.14313E-4 63.10966 1.81696E-4 81.8155 1.62805E-4 65.21632 1.89476E-4 66.16432 1.5123E-4 56.98536 0.00394 91.66651 0.00442 87.21516 0.00528 85.06264 0.00624 82.60167 0.00816 74.3753

1.20675E-4 65.32746 1.93101E-4 83.49468 1.72577E-4 66.96997 2.01123E-4 67.70909 1.6102E-4 58.61615 0.00402 92.81671 0.00451 88.28992 0.00539 86.07405 0.00637 83.52992 0.00833 75.0781

1.27269E-4 66.94733 2.0507E-4 85.67668 1.82786E-4 68.68081 2.13321E-4 69.46087 1.71434E-4 60.42356 0.0041 93.93788 0.0046 89.33424 0.0055 87.05277 0.0065 84.42297 0.0085 75.74167

1.3409E-4 68.89335 2.17481E-4 87.34638 1.93364E-4 70.30788 2.25965E-4 70.94442 1.82359E-4 62.66395 0.00418 95.03052 0.00469 90.34862 0.00561 87.99941 0.00663 85.28158 0.00867 76.36715

1.41211E-4 70.90459 2.30355E-4 89.90786 2.04361E-4 72.36561 2.39094E-4 73.02306 1.93688E-4 63.66128 0.00426 96.09513 0.00478 91.33363 0.00572 88.91464 0.00676 86.10663 0.00884 76.95581

1.48664E-4 72.72015 2.43738E-4 92.14679 2.15821E-4 74.19012 2.52758E-4 74.85161 2.05394E-4 65.32728 0.00435 97.13227 0.00488 92.28985 0.00583 89.7992 0.00689 86.89907 0.00901 77.50905

1.56377E-4 74.57919 2.57487E-4 93.79751 2.27625E-4 75.76952 2.66812E-4 76.30516 2.17494E-4 66.41846 0.00443 98.14248 0.00497 93.21788 0.00594 90.65385 0.00702 87.65991 0.00918 78.02839

1.64306E-4 76.40563 2.7149E-4 96.61514 2.39688E-4 77.85934 2.81148E-4 78.51352 2.30016E-4 67.84978 0.00451 99.12635 0.00506 94.11837 0.00605 91.47942 0.00715 88.39024 0.00935 78.51547

1.72476E-4 77.78632 2.85639E-4 99.28097 2.51963E-4 79.68028 2.95681E-4 80.53255 2.4302E-4 69.18741 0.00459 100.08446 0.00515 94.992 0.00616 92.27678 0.00728 89.09121 0.00952 78.97205

1.80922E-4 79.73234 2.99871E-4 101.18785 2.64436E-4 81.41408 3.10369E-4 82.17087 2.56502E-4 70.32542 0.00467 101.01741 0.00524 95.83947 0.00627 93.04683 0.00741 89.76405 0.00969 79.39997

1.89612E-4 81.45005 3.14229E-4 103.72087 2.77113E-4 83.32691 3.25238E-4 84.1715 2.70446E-4 71.85059 0.00476 101.92583 0.00534 96.66149 0.00638 93.79053 0.00754 90.41002 0.00986 79.80117

1.98514E-4 82.90684 3.28684E-4 105.97876 2.89959E-4 84.99852 3.40254E-4 85.93978 2.84824E-4 73.01203 0.00484 102.81035 0.00543 97.45883 0.00649 94.50886 0.00767 91.03045 0.01003 80.17768

2.07624E-4 84.86372 3.43274E-4 108.02794 3.02994E-4 86.80125 3.55447E-4 87.67314 2.99575E-4 74.70159 0.00492 103.67161 0.00552 98.23225 0.0066 95.20285 0.0078 91.62671 0.0102 80.53156

2.16892E-4 86.62493 3.57954E-4 109.63125 3.16165E-4 88.31528 3.70766E-4 89.07594 3.14694E-4 76.33238 0.005 104.51027 0.00561 98.98254 0.00671 95.87354 0.00793 92.20021 0.01037 80.86495

2.26248E-4 88.14695 3.7265E-4 111.31044 3.29394E-4 89.75583 3.86124E-4 90.47982 3.30181E-4 77.92812 0.00508 105.32699 0.0057 99.71051 0.00682 96.52202 0.00806 92.75239 0.01054 81.18002

2.35732E-4 89.83205 3.87411E-4 112.56272 3.42729E-4 91.07764 4.01577E-4 91.63816 3.45992E-4 79.54713 0.00517 106.12244 0.0058 100.41699 0.00693 97.14939 0.00819 93.28474 0.01071 81.47896

2.4536E-4 91.79982 4.02273E-4 114.0996 3.56198E-4 92.65473 4.17159E-4 93.03945 3.62151E-4 81.37738 0.00525 106.89731 0.00589 101.10281 0.00704 97.75676 0.00832 93.79874 0.01088 81.76398

2.55084E-4 92.97394 4.17118E-4 115.37086 3.69711E-4 93.75516 4.32756E-4 94.10671 3.78722E-4 82.43334 0.00533 107.65228 0.00598 101.76883 0.00715 98.34528 0.00845 94.29589 0.01105 82.03725

2.64963E-4 94.90909 4.31915E-4 116.39544 3.83283E-4 95.08704 4.48359E-4 95.16712 3.95654E-4 83.72398 0.00541 108.38804 0.00607 102.4159 0.00726 98.9161 0.00858 94.77773 0.01122 82.30097

2.75144E-4 96.19194 4.46778E-4 118.16001 3.97057E-4 96.45837 4.64109E-4 96.57828 4.12945E-4 84.92077 0.00549 109.10528 0.00616 103.04489 0.00737 99.47038 0.00871 95.24575 0.01139 82.55725

2.8555E-4 97.09428 4.61597E-4 118.63436 4.10931E-4 97.07788 4.7989E-4 97.07051 4.30613E-4 86.00002 0.00558 109.8047 0.00626 103.65668 0.00748 100.00927 0.00884 95.70147 0.01156 82.80821

2.96074E-4 99.24686 4.76243E-4 120.08586 4.24774E-4 98.69972 4.9556E-4 98.45351 4.48562E-4 86.93887 0.00566 110.487 0.00635 104.25215 0.00759 100.53396 0.00897 96.14638 0.01173 83.05585

3.06723E-4 100.6058 4.90733E-4 121.61327 4.38601E-4 99.99858 5.11134E-4 99.72533 4.66746E-4 88.89802 0.00574 111.15288 0.00644 104.83217 0.0077 101.04559 0.0091 96.58198 0.0119 83.30212

3.17432E-4 102.4866 5.05027E-4 122.97938 4.52352E-4 101.45969 5.26559E-4 100.99758 4.85164E-4 89.66082 0.00582 111.80303 0.00653 105.39763 0.00781 101.54532 0.00923 97.0097 0.01207 83.54891

3.28227E-4 104.56307 5.19107E-4 124.1368 4.66034E-4 102.91494 5.41827E-4 102.17328 5.03877E-4 90.97474 0.0059 112.43815 0.00662 105.9494 0.00792 102.03431 0.00936 97.43096 0.01224 83.79796

3.39108E-4 106.40037 5.32985E-4 125.61675 4.79651E-4 104.4077 5.5695E-4 103.51101 5.22845E-4 91.4675 0.00599 113.05893 0.00672 106.48837 0.00803 102.51369 0.00949 97.84717 0.01241 84.05095

3.49936E-4 108.60732 5.4663E-4 127.31493 4.93111E-4 106.165 5.71858E-4 105.06597 5.42034E-4 92.61731 0.00607 113.66606 0.00681 107.01539 0.00814 102.98457 0.00962 98.25964 0.01258 84.30941

3.60739E-4 110.59682 5.5996E-4 128.48183 5.06384E-4 107.58539 5.86489E-4 106.23025 5.61466E-4 93.70848 0.00615 114.26023 0.0069 107.53133 0.00825 103.44805 0.00975 98.66969 0.01275 84.57478

3.71467E-4 112.60806 5.72855E-4 129.78154 5.19377E-4 109.07531 6.00728E-4 107.48559 5.81111E-4 94.4125 0.00623 114.84212 0.00699 108.03704 0.00836 103.90521 0.00988 99.07855 0.01292 84.84834

3.81973E-4 114.82587 5.85292E-4 130.92946 5.31996E-4 110.58989 6.14508E-4 108.68372 6.00987E-4 95.5738 0.00631 115.41239 0.00708 108.53336 0.00847 104.3571 0.01001 99.48741 0.01309 85.13127

3.92297E-4 117.31546 5.97239E-4 132.39046 5.44245E-4 112.36767 6.27816E-4 110.14115 6.21143E-4 95.56216 0.0064 115.97171 0.00718 109.02112 0.00858 104.80473 0.01014 99.8974 0.01326 85.42459

4.02534E-4 119.06579 6.08713E-4 134.14555 5.56186E-4 113.9451 6.40694E-4 111.64079 6.41494E-4 96.10193 0.00648 116.52074 0.00727 109.50114 0.00869 105.24911 0.01027 100.30957 0.01343 85.7292

4.12706E-4 121.15316 6.19771E-4 135.35037 5.67862E-4 115.42659 6.53198E-4 112.84963 6.62005E-4 96.4188 0.00656 117.06012 0.00736 109.97421 0.0088 105.69118 0.0104 100.72493 0.0136 86.04587

4.22811E-4 122.44686 6.30538E-4 137.29522 5.79342E-4 116.88393 6.65434E-4 114.38062 6.82758E-4 96.06686 0.00664 117.59049 0.00745 110.44112 0.00891 106.13187 0.01053 101.14441 0.01377 86.37522

4.3283E-4 124.42549 6.41111E-4 138.57594 5.90668E-4 118.35064 6.77478E-4 115.61697 7.03692E-4 96.87622 0.00672 118.11248 0.00754 110.90262 0.00902 106.57207 0.01066 101.56887 0.01394 86.71773

4.42849E-4 125.70834 6.51425E-4 140.37848 6.01851E-4 119.73907 6.89302E-4 117.05289 7.24758E-4 97.13417 0.00681 118.6267 0.00764 111.35947 0.00913 107.01261 0.01079 101.99909 0.01411 87.07378

4.52912E-4 126.55632 6.61356E-4 142.26638 6.12847E-4 120.97022 7.00812E-4 118.45647 7.45966E-4 97.66229 0.00689 119.13376 0.00773 111.81239 0.00924 107.45431 0.01092 102.43578 0.01428 87.44361

4.62907E-4 127.8881 6.71001E-4 143.51865 6.23649E-4 122.13304 7.12058E-4 119.54326 7.67278E-4 98.7651 0.00697 119.63425 0.00782 112.26208 0.00935 107.89793 0.01105 102.8796 0.01445 87.82733

4.72858E-4 128.93721 6.80526E-4 145.24528 6.34361E-4 123.38213 7.23188E-4 120.88233 7.88604E-4 99.27571 0.00705 120.12874 0.00791 112.70921 0.00946 108.34418 0.01118 103.33111 0.01462 88.22495

4.82772E-4 129.5895 6.89873E-4 147.1237 6.44955E-4 124.52094 7.34155E-4 122.24009 8.08E-4 99.83286 0.00713 120.6178 0.008 113.15445 0.00957 108.79375 0.01131 103.7908 0.01479 88.63636

4.92617E-4 130.37224 6.99136E-4 148.74596 6.55464E-4 125.60319 7.4503E-4 123.45712 8.32E-4 100.65414 0.00722 121.10198 0.0081 113.59843 0.00968 109.24727 0.01144 104.25911 0.01496 89.06137

5.0239E-4 131.86167 7.08362E-4 149.97928 6.65914E-4 126.82843 7.55852E-4 124.56346 8.56E-4 101.26432 0.0073 121.58182 0.00819 114.04175 0.00979 109.70532 0.01157 104.73638 0.01513 89.49968

5.12126E-4 133.63375 7.17566E-4 151.70589 6.76331E-4 128.40284 7.66643E-4 126.04893 8.72E-4 101.60449 0.00738 122.05783 0.00828 114.48499 0.0099 110.16845 0.0117 105.22291 0.0153 89.9509

5.21875E-4 135.46018 7.2674E-4 152.60715 6.86738E-4 129.63029 7.77413E-4 127.00686 8.96E-4 102.44919 0.00746 122.53052 0.00837 114.92872 0.01001 110.63714 0.01183 105.7189 0.01547 90.41457

5.31685E-4 137.51491 7.35906E-4 153.56534 6.97175E-4 130.98612 7.88195E-4 128.04816 9.12E-4 102.8599 0.00754 123.00039 0.00846 115.37345 0.01012 111.11187 0.01196 106.22452 0.01564 90.89017

5.41512E-4 139.42831 7.45109E-4 154.51403 7.07642E-4 132.27406 7.99013E-4 129.05464 9.36E-4 103.77518 0.00763 123.4679 0.00856 115.8197 0.01023 111.59302 0.01209 106.73985 0.01581 91.3771

5.51403E-4 141.43954 7.54312E-4 155.46273 7.18143E-4 133.60602 8.0985E-4 130.08093 9.52E-4 103.66956 0.00771 123.93352 0.00865 116.26794 0.01034 112.08097 0.01222 107.26492 0.01598 91.8747

5.61383E-4 143.20075 7.63603E-4 156.33554 7.28742E-4 134.79133 8.2079E-4 131.00709 9.76E-4 103.99807 0.00779 124.39768 0.00874 116.71861 0.01045 112.57603 0.01235 107.79971 0.01615 92.3823

5.71337E-4 145.41857 7.72949E-4 156.8668 7.39357E-4 136.02842 8.31768E-4 131.80284 9.92E-4 104.59632 0.00787 124.86081 0.00883 117.17214 0.01056 113.07847 0.01248 108.34412 0.01632 92.89913

5.81261E-4 147.1254 7.82208E-4 157.22731 7.49908E-4 136.95872 8.42664E-4 132.38371 0.00102 104.37328 0.00795 125.32332 0.00892 117.62892 0.01067 113.58853 0.01261 108.89802 0.01649 93.42444

5.91164E-4 148.88661 7.9138E-4 157.61627 7.60399E-4 137.92629 8.53479E-4 132.99415 0.00103 105.01882 0.00804 125.7856 0.00902 118.08932 0.01078 114.1064 0.01274 109.46122 0.01666 93.95743

6.01008E-4 150.64781 8.00457E-4 157.69216 7.70806E-4 138.75299 8.64195E-4 133.40031 0.00106 105.26514 0.00812 126.24803 0.00911 118.55367 0.01089 114.63223 0.01287 110.0335 0.01683 94.49728

6.10707E-4 151.59362 8.09436E-4 157.07551 7.81079E-4 138.90111 8.74783E-4 133.18948 0.00107 104.9132 0.0082 126.71095 0.0092 119.02229 0.011 115.16613 0.013 110.61457 0.017 95.04314

6.20308E-4 153.82232 8.18319E-4 157.45499 7.91245E-4 140.07479 8.8526E-4 133.8884 0.00109 105.37062 0.00828 127.17472 0.00929 119.49546 0.01111 115.70817 0.01313 111.20413 0.01717 95.59418

6.29817E-4 155.10516 8.26994E-4 157.17038 8.01246E-4 140.52399 8.95532E-4 133.96247 0.0011 104.92484 0.00836 127.63966 0.00938 119.97345 0.01122 116.25839 0.01326 111.8018 0.01734 96.14954

6.39157E-4 156.23036 8.35476E-4 157.13243 8.11048E-4 141.01326 9.05588E-4 134.16556 0.00113 105.26514 0.00845 128.10607 0.00948 120.45648 0.01133 116.81679 0.01339 112.40722 0.01751 96.70837

6.48391E-4 157.61649 8.43686E-4 156.38296 8.20642E-4 141.29975 9.15381E-4 133.95722 0.00114 105.49952 0.00853 128.57425 0.00957 120.94476 0.01144 117.38333 0.01352 113.01995 0.01768 97.26981

6.57549E-4 159.17115 8.51552E-4 156.09835 8.30006E-4 141.87127 9.24857E-4 134.08633 0.00116 105.73434 0.00861 129.04446 0.00966 121.43847 0.01155 117.95795 0.01365 113.63955 0.01785 97.83301

6.66563E-4 159.73647 8.59066E-4 155.7758 8.39096E-4 141.98053 9.33989E-4 133.99034 0.00118 105.75777 0.00869 129.51697 0.00975 121.93777 0.01166 118.54055 0.01378 114.26555 0.01802 98.39715

6.75445E-4 161.13891 8.66214E-4 155.09274 8.47912E-4 142.30425 9.4277E-4 133.82864 0.00119 106.33259 0.00877 129.99201 0.00984 122.44279 0.01177 119.131 0.01391 114.89744 0.01819 98.96138

6.84221E-4 161.30197 8.73091E-4 154.49506 8.56522E-4 142.10867 9.51287E-4 133.47167 0.00121 106.03957 0.00886 130.4698 0.00994 122.95364 0.01188 119.72915 0.01404 115.5347 0.01836 99.52489

6.92788E-4 162.04125 8.79612E-4 153.65073 8.6482E-4 142.06139 9.59438E-4 133.07045 0.00122 106.55577 0.00894 130.95056 0.01003 123.47041 0.01199 120.33481 0.01417 116.1768 0.01853 100.08689

7.01173E-4 162.22605 8.85828E-4 153.4515 8.72851E-4 142.05489 9.67273E-4 132.97788 0.00124 106.39137 0.00902 131.43447 0.01012 123.99314 0.0121 120.94777 0.0143 116.82319 0.0187 100.64657

7.09388E-4 162.84575 8.9177E-4 152.12332 8.80637E-4 141.73608 9.74824E-4 132.23673 0.00126 106.00438 0.0091 131.92171 0.01021 124.52189 0.01221 121.5678 0.01443 117.47329 0.01887 101.20318

7.17257E-4 163.11754 8.97288E-4 151.58256 8.88E-4 141.61504 9.81908E-4 131.93892 0.00127 105.78147 0.00918 132.41244 0.0103 125.05667 0.01232 122.19464 0.01456 118.12652 0.01904 101.75596

7.24791E-4 163.25887 9.02407E-4 151.1936 9.76319E-4 141.50361 0.00113 131.71374 0.00129 104.831 0.00927 132.90679 0.0104 125.59747 0.01243 122.82801 0.01469 118.78229 0.01921 102.30416

7.32068E-4 163.6285 9.072E-4 150.58644 9.83561E-4 141.39672 0.00113 131.39242 0.0013 104.5729 0.00935 133.40491 0.01049 126.14427 0.01254 123.46762 0.01482 119.43999 0.01938 102.84706

7.39021E-4 163.44368 9.11677E-4 150.26389 9.90419E-4 141.16841 0.00114 131.14453 0.00131 103.97435 0.00943 133.90689 0.01058 126.69702 0.01265 124.11314 0.01495 120.09901 0.01955 103.38397

7.45735E-4 163.42193 9.15863E-4 149.78953 9.96959E-4 140.94515 0.00115 130.83061 0.00133 103.54065 0.00951 134.41284 0.01067 127.25566 0.01276 124.76424 0.01508 120.75874 0.01972 103.91419

7.52219E-4 163.15015 9.19779E-4 149.35314 1E-3 140.62648 0.00115 130.49083 0.00134 103.45816 0.00959 134.92284 0.01076 127.82011 0.01287 125.42056 0.01521 121.41855 0.01989 104.43704

7.58451E-4 163.40019 9.23456E-4 148.7934 0.00101 140.48711 0.00116 130.17623 0.00135 103.51679 0.00968 135.43695 0.01086 128.39026 0.01298 126.08174 0.01534 122.07781 0.02006 104.95187

7.64457E-4 163.09579 9.26904E-4 147.89213 0.00101 139.94456 0.00117 129.52652 0.00137 103.68105 0.00976 135.95523 0.01095 128.96598 0.01309 126.74739 0.01547 122.73589 0.02023 105.45803

7.70202E-4 162.37826 9.30087E-4 147.20908 0.00102 139.3143 0.00117 128.93552 0.00138 103.51694 0.00984 136.47772 0.01104 129.54715 0.0132 127.41711 0.0156 123.39214 0.0204 105.95488

7.75715E-4 162.30217 9.32937E-4 146.38372 0.00103 138.90865 0.00117 128.38157 0.00139 103.81025 0.00992 137.00445 0.01113 130.13361 0.01331 128.09048 0.01573 124.04594 0.02057 106.4418

7.8104E-4 161.57376 9.35467E-4 145.805 0.00103 138.32044 0.00118 127.85645 0.0014 103.73983 0.01 137.53541 0.01122 130.72518 0.01342 128.76709 0.01586 124.69663 0.02074 106.91818

7.86214E-4 160.75838 9.3774E-4 144.91324 0.00103 137.55223 0.00118 127.10947 0.00142 103.38803 0.01009 138.07061 0.01132 131.32168 0.01353 129.44649 0.01599 125.34357 0.02091 107.38339

7.91208E-4 160.63881 9.39707E-4 143.5566 0.00104 136.88794 0.00119 126.20004 0.00142 103.45831 0.01017 138.61004 0.01141 131.92291 0.01364 130.12823 0.01612 125.98611 0.02108 107.83685

7.96052E-4 159.58426 9.41383E-4 142.59842 0.00104 135.98221 0.00119 125.36128 0.00143 103.18828 0.01025 139.15366 0.0115 132.52864 0.01375 130.81186 0.01625 126.62359 0.02125 108.27796

8.00749E-4 158.58407 9.42707E-4 141.22281 0.00105 134.91309 0.00119 124.26116 0.00145 103.15322 0.01033 139.70143 0.01159 133.13863 0.01386 131.49689 0.01638 127.25536 0.02142 108.70612

8.05284E-4 157.89916 9.43669E-4 139.56259 0.00105 133.85779 0.0012 123.03917 0.00146 102.78949 0.01041 140.25329 0.01168 133.75265 0.01397 132.18286 0.01651 127.88076 0.02159 109.12073

8.09744E-4 156.6924 9.44303E-4 138.34826 0.00105 132.7683 0.0012 122.00245 0.00146 102.66044 0.0105 140.80917 0.01178 134.37043 0.01408 132.86926 0.01664 128.49912 0.02176 109.5212

8.14003E-4 155.90966 9.44626E-4 136.90624 0.00106 131.76716 0.0012 120.90303 0.00148 102.33193 0.01058 141.36899 0.01187 134.99168 0.01419 133.5556 0.01677 129.10977 0.02193 109.90695

8.17935E-4 154.96382 9.44702E-4 136.03345 0.00106 130.94877 0.0012 120.142 0.00149 101.61627 0.01066 141.93265 0.01196 135.61612 0.0143 134.24135 0.0169 129.71204 0.0221 110.27736

8.21613E-4 154.22456 9.44479E-4 134.37322 0.00106 129.86901 0.0012 118.909 0.0015 101.34652 0.01074 142.50004 0.01205 136.24344 0.01441 134.926 0.01703 130.30524 0.02227 110.63182

8.25099E-4 152.95258 9.43913E-4 133.17788 0.00106 128.7587 0.0012 117.87146 0.0015 100.68936 0.01082 143.07104 0.01214 136.87332 0.01452 135.60901 0.01716 130.88868 0.02244 110.96974

8.28365E-4 151.80019 9.43024E-4 131.57457 0.00106 127.51865 0.0012 116.59195 0.00151 100.44302 0.01091 143.64552 0.01224 137.50542 0.01463 136.28983 0.01729 131.46167 0.02261 111.29048

8.31374E-4 150.94133 9.41765E-4 130.51204 0.00106 126.65402 0.0012 115.72472 0.00152 99.87976 0.01099 144.22332 0.01233 138.1394 0.01474 136.96791 0.01742 132.02349 0.02278 111.5934

8.34118E-4 149.89766 9.40191E-4 128.83284 0.00106 125.42873 0.0012 114.41771 0.00153 98.85907 0.01107 144.80429 0.01242 138.77488 0.01485 137.64267 0.01755 132.57341 0.02295 111.87787

8.3657E-4 148.85399 9.38402E-4 126.88801 0.00106 124.0839 0.0012 112.93736 0.00154 98.22538 0.01115 145.38825 0.01251 139.4115 0.01496 138.31354 0.01768 133.1107 0.02312 112.14321

8.38791E-4 147.98426 9.36333E-4 125.69267 0.00107 123.15461 0.0012 111.98128 0.00154 98.0612 0.01123 145.975 0.0126 140.04885 0.01507 138.97992 0.01781 133.63462 0.02329 112.38875

8.40709E-4 147.23412 9.33971E-4 124.55423 0.00106 122.30476 0.0012 111.08654 0.00155 97.32204 0.01132 146.56436 0.0127 140.68653 0.01518 139.6412 0.01794 134.14439 0.02346 112.61379

8.42311E-4 146.12521 9.31316E-4 123.25451 0.00106 121.22087 0.0012 110.01393 0.00156 97.00529 0.0114 147.15609 0.01279 141.32412 0.01529 140.29674 0.01807 134.63922 0.02363 112.81761

8.43629E-4 145.35333 9.28294E-4 122.24889 0.00106 120.421 0.00119 109.20146 0.00157 96.5126 0.01148 147.74997 0.01288 141.96116 0.0154 140.94592 0.0182 135.11832 0.0238 112.99947

8.44605E-4 144.08135 9.24847E-4 121.59429 0.00106 119.55405 0.00119 108.51675 0.00158 96.40692 0.01156 148.34576 0.01297 142.5972 0.01551 141.58805 0.01833 135.58084 0.02397 113.15861

8.45272E-4 143.30947 9.2102E-4 119.67793 0.00106 118.34433 0.00119 107.11002 0.00158 95.7382 0.01164 148.94318 0.01306 143.23176 0.01562 142.22248 0.01846 136.02593 0.02414 113.29424

8.45644E-4 142.9181 9.16962E-4 118.7482 0.00106 117.74983 0.00118 106.42412 0.00158 95.44487 0.01173 149.54196 0.01316 143.86435 0.01573 142.84849 0.01859 136.4527 0.02431 113.40553

8.45765E-4 141.59175 9.12684E-4 117.53388 0.00106 116.60653 0.00118 105.36319 0.00159 95.15168 0.01181 150.14181 0.01325 144.49444 0.01584 143.46535 0.01872 136.86025 0.02448 113.49163

8.45647E-4 140.68941 9.0813E-4 115.98751 0.00105 115.50461 0.00118 104.17145 0.00159 94.44758 0.01189 150.74241 0.01334 145.12149 0.01595 144.07231 0.01885 137.24763 0.02465 113.55167

8.45288E-4 139.96101 9.03292E-4 114.27987 0.00105 114.4084 0.00117 102.90972 0.0016 94.09569 0.01197 151.34343 0.01343 145.74495 0.01606 144.6686 0.01898 137.61384 0.02482 113.58472

8.44731E-4 138.94996 8.98143E-4 112.66708 0.00105 113.22766 0.00117 101.65263 0.0016 93.4152 0.01205 151.94452 0.01352 146.36423 0.01617 145.25341 0.01911 137.95786 0.02499 113.58984

8.43998E-4 138.21068 8.92659E-4 110.77917 0.00104 112.04543 0.00116 100.27108 0.00161 92.55871 0.01214 152.54531 0.01362 146.97871 0.01628 145.82589 0.01924 138.27863 0.02516 113.56602

8.43052E-4 137.64535 8.86771E-4 109.02409 0.00104 111.00124 0.00115 99.0114 0.00161 91.43245 0.01222 153.1454 0.01371 147.58775 0.01639 146.38517 0.01937 138.57503 0.02533 113.51225

8.41925E-4 137.19963 8.80417E-4 107.10773 0.00103 109.93831 0.00115 97.67072 0.00162 90.54067 0.0123 153.74438 0.0138 148.19067 0.0165 146.93032 0.0195 138.84589 0.0255 113.42743

8.4068E-4 136.03636 8.7363E-4 105.19136 0.00103 108.55247 0.00114 96.18472 0.00162 89.1445 0.01238 154.3418 0.01389 148.78677 0.01661 147.46039 0.01963 139.08999 0.02567 113.31044

8.39266E-4 135.55801 8.66522E-4 103.64499 0.00102 107.64135 0.00113 95.07885 0.00162 88.10027 0.01246 154.9372 0.01398 149.37532 0.01672 147.97437 0.01976 139.30605 0.02584 113.16011

8.37647E-4 134.63392 8.59066E-4 100.8653 0.00102 105.97465 0.00113 93.07799 0.00162 86.83297 0.01255 155.53008 0.01408 149.95551 0.01683 148.4712 0.01989 139.49271 0.02601 112.97521

8.35907E-4 133.77506 8.51159E-4 99.1956 0.00101 104.8368 0.00112 91.81458 0.00162 85.76536 0.01263 156.1199 0.01417 150.52654 0.01694 148.94976 0.02002 139.64856 0.02618 112.75446

8.3403E-4 132.66616 8.42806E-4 97.34565 0.00101 103.50532 0.00111 90.38293 0.00162 84.47489 0.01271 156.70612 0.01426 151.08754 0.01705 149.40888 0.02015 139.7721 0.02635 112.49653

8.31995E-4 131.64422 8.33981E-4 95.60004 9.99586E-4 102.25992 0.0011 89.03698 0.00162 83.325 0.01279 157.28812 0.01435 151.63757 0.01716 149.84731 0.02028 139.86176 0.02652 112.20001

8.29794E-4 131.10066 8.24718E-4 93.40856 9.92707E-4 101.02915 0.00109 87.49697 0.00162 81.97583 0.01287 157.86527 0.01444 152.17566 0.01727 150.26374 0.02041 139.91586 0.02669 111.86344

8.27425E-4 129.75256 8.15057E-4 92.00449 9.85489E-4 99.79067 0.00108 86.30782 0.00162 80.36855 0.01296 158.43688 0.01454 152.70078 0.01738 150.65676 0.02054 139.93264 0.02686 111.48529

8.24956E-4 129.27423 8.05029E-4 90.72375 9.77991E-4 98.99909 0.00107 85.37527 0.00162 79.12484 0.01304 159.00223 0.01463 153.21183 0.01749 151.02488 0.02067 139.91023 0.02703 111.06395

8.22365E-4 128.36101 7.94674E-4 89.24379 9.70223E-4 97.92216 0.00106 84.22467 0.00162 77.85759 0.01312 159.56053 0.01472 153.70761 0.0176 151.36653 0.0208 139.84664 0.0272 110.59776

8.19644E-4 127.176 7.83989E-4 87.96305 9.6218E-4 96.81257 0.00105 83.14903 0.00162 76.15642 0.0132 160.11094 0.01481 154.18687 0.01771 151.67999 0.02093 139.73977 0.02737 110.08495

8.16767E-4 126.18669 7.73039E-4 86.65384 9.53884E-4 95.77824 0.00104 82.09444 0.00162 74.98322 0.01328 160.65257 0.0149 154.64826 0.01782 151.96347 0.02106 139.58737 0.02754 109.52369

8.13843E-4 124.71902 7.618E-4 85.40157 9.45386E-4 94.55427 0.00102 80.98013 0.00162 73.72799 0.01337 161.18443 0.015 155.09034 0.01793 152.215 0.02119 139.38706 0.02771 108.91206

8.1087E-4 123.27311 7.50275E-4 83.86468 9.36687E-4 93.212 0.00101 79.68451 0.00162 72.95341 0.01345 161.70547 0.01509 155.51153 0.01804 152.43252 0.02132 139.13629 0.02788 108.24805

8.07705E-4 121.49016 7.38534E-4 82.26139 9.27743E-4 91.6882 9.99766E-4 78.27731 0.00162 71.53386 0.01353 162.21455 0.01518 155.91016 0.01815 152.61375 0.02145 138.83234 0.02805 107.52956

8.04316E-4 120.39214 7.26643E-4 80.8763 9.18575E-4 90.5708 9.87131E-4 77.15119 0.00161 71.07615 0.01361 162.71044 0.01527 156.2844 0.01826 152.75629 0.02158 138.47232 0.02822 106.75437

8.00643E-4 119.38107 7.1469E-4 79.64299 9.092E-4 89.56083 9.74334E-4 76.14172 0.00161 69.59795 0.01369 163.1918 0.01536 156.63229 0.01837 152.8575 0.02171 138.05312 0.02839 105.92021

7.96877E-4 117.33722 7.02489E-4 78.50455 8.9962E-4 88.1288 9.61265E-4 74.985 0.0016 68.45993 0.01378 163.65716 0.01546 156.95168 0.01848 152.91455 0.02184 137.57143 0.02856 105.02465

7.9304E-4 116.01088 6.89932E-4 76.96767 8.89783E-4 86.84035 9.47829E-4 73.71361 0.0016 67.50943 0.01386 164.10492 0.01555 157.24024 0.01859 152.92435 0.02197 137.02368 0.02873 104.0652

7.88991E-4 114.48886 6.76916E-4 75.76282 8.79544E-4 85.61325 9.33876E-4 72.61923 0.00159 65.70271 0.01394 164.53333 0.01564 157.49542 0.0187 152.88355 0.0221 136.40606 0.0289 103.03923

7.84837E-4 112.70591 6.6337E-4 72.9452 8.68924E-4 83.543 9.19377E-4 70.41968 0.00159 64.7054 0.01402 164.94045 0.01573 157.71442 0.01881 152.78851 0.02223 135.71447 0.02907 101.94401

7.80558E-4 111.32521 6.49552E-4 70.75372 8.58066E-4 81.93552 9.04571E-4 68.71015 0.00158 64.0248 0.0141 165.32417 0.01582 157.89419 0.01892 152.63523 0.02236 134.9445 0.02924 100.77672

7.76141E-4 109.46618 6.35713E-4 69.62476 8.47112E-4 80.59092 8.89695E-4 67.59706 0.00158 62.87512 0.01419 165.68214 0.01592 158.03135 0.01903 152.41934 0.02249 134.09141 0.02941 99.53439

7.71555E-4 107.82456 6.21908E-4 68.96068 8.36078E-4 79.55336 8.74791E-4 66.83132 0.00158 62.39419 0.01427 166.01174 0.01601 158.12218 0.01914 152.13606 0.02262 133.1501 0.02958 98.21397

7.66778E-4 106.48735 6.08112E-4 68.70452 8.24934E-4 78.83635 8.59828E-4 66.39339 0.00157 61.64329 0.01435 166.31009 0.0161 158.16255 0.01925 151.78011 0.02275 132.11506 0.02975 96.81229

7.6179E-4 104.78051 5.94289E-4 67.93608 8.13647E-4 77.72247 8.44762E-4 65.54634 0.00156 60.69279 0.01443 166.57395 0.01619 158.14788 0.01936 151.34569 0.02288 130.98037 0.02992 95.32608

7.56581E-4 103.80206 5.80407E-4 67.24353 8.02193E-4 76.97052 8.2956E-4 64.89633 0.00155 60.04762 0.01451 166.79968 0.01628 158.07307 0.01947 150.82639 0.02301 129.73962 0.03009 93.752

7.51213E-4 101.92128 5.66503E-4 66.74073 7.9063E-4 75.89791 8.1428E-4 64.18738 0.00154 59.2849 0.0146 166.98321 0.01638 157.93241 0.01958 150.21511 0.02314 128.38588 0.03026 92.0866

7.45712E-4 100.35577 5.52574E-4 65.80152 7.78972E-4 74.77078 7.98927E-4 63.25753 0.00154 58.55754 0.01468 167.11993 0.01647 157.7195 0.01969 149.50393 0.02327 126.91171 0.03043 90.32638

7.40105E-4 98.90985 5.38741E-4 65.45525 7.67308E-4 73.9643 7.83629E-4 62.73879 0.00154 58.36984 0.01476 167.20462 0.01656 157.42713 0.0198 148.68409 0.0234 125.30901 0.0306 88.46781

7.34348E-4 97.60525 5.24956E-4 63.55311 7.55582E-4 72.52127 7.68323E-4 61.23346 0.00153 57.37247 0.01484 167.23132 0.01665 157.04714 0.01991 147.74577 0.02353 123.56908 0.03077 86.50734

7.28351E-4 96.48548 5.11108E-4 62.78467 7.43675E-4 71.67156 7.5287E-4 60.5053 0.00152 56.89153 0.01492 167.19322 0.01674 156.57028 0.02002 146.67799 0.02366 121.68248 0.03094 84.44144

7.22184E-4 94.96345 4.97106E-4 62.31981 7.31574E-4 70.77747 7.37208E-4 59.89378 0.00151 56.35181 0.01501 167.08247 0.01684 155.98601 0.02013 145.46839 0.02379 119.63904 0.03111 82.26667

7.15846E-4 94.1807 4.82873E-4 60.87779 7.19231E-4 69.77632 7.21263E-4 58.79435 0.0015 55.89432 0.01509 166.89 0.01693 155.28225 0.02024 144.1031 0.02392 117.4278 0.03128 79.97971

7.09329E-4 92.60432 4.68542E-4 59.45475 7.06723E-4 68.42658 7.05159E-4 57.5466 0.0015 55.53042 0.01517 166.60528 0.01702 154.44513 0.02035 142.56641 0.02405 115.03695 0.03145 77.57744

7.0265E-4 91.83243 4.54218E-4 58.21196 6.94121E-4 67.51998 6.89003E-4 56.57936 0.0015 54.22818 0.01525 166.21595 0.01711 153.45859 0.02046 140.84054 0.02418 112.45382 0.03162 75.05706

6.95829E-4 90.31041 4.39892E-4 57.28224 6.81433E-4 66.4167 6.72795E-4 55.66452 0.00149 53.55939 0.01533 165.70747 0.0172 152.30399 0.02057 138.90528 0.02431 109.66483 0.03179 72.41614

6.88885E-4 89.38632 4.25582E-4 55.67894 6.6868E-4 65.27937 6.56557E-4 54.43123 0.00148 52.83202 0.01542 165.06259 0.0173 150.95954 0.02068 136.73756 0.02444 106.65558 0.03196 69.65279

6.81801E-4 88.53834 4.11249E-4 54.66383 6.5583E-4 64.44098 6.40247E-4 53.59716 0.00147 52.31574 0.0155 164.26068 0.01739 149.39964 0.02079 134.31106 0.02457 103.41081 0.03213 66.76581

6.74527E-4 87.23374 3.96837E-4 53.11746 6.42818E-4 63.15803 6.23793E-4 52.32397 0.00147 50.96657 0.01558 163.27687 0.01748 147.59399 0.0209 131.59555 0.0247 99.91459 0.0323 63.75482

6.67067E-4 86.39663 3.82339E-4 51.60903 6.29644E-4 62.10255 6.0719E-4 51.17021 0.00146 50.0749 0.01566 162.0809 0.01757 145.50654 0.02101 128.55634 0.02483 96.15049 0.03247 60.62052

6.59496E-4 85.30947 3.677E-4 50.16702 6.16318E-4 60.96442 5.90411E-4 50.00914 0.00146 49.20674 0.01574 160.6356 0.01766 143.09408 0.02112 125.15348 0.02496 92.10193 0.03264 57.36481

6.51775E-4 84.25493 3.52923E-4 48.79615 6.02819E-4 59.87299 5.73445E-4 48.90111 0.00145 48.29161 0.01583 158.8948 0.01776 140.3044 0.02123 121.34095 0.02509 87.7527 0.03281 53.99104

6.43931E-4 83.02644 3.38094E-4 47.88067 5.89215E-4 58.9082 5.56385E-4 48.05499 0.00145 46.6137 0.01591 156.80046 0.01785 137.07401 0.02134 117.0658 0.02522 83.08771 0.03298 50.50415

6.35943E-4 81.41743 -- -- 5.75539E-4 57.58646 5.39296E-4 46.86253 0.00144 45.17058 0.01599 154.27864 0.01794 133.32507 0.02145 112.26724 0.02535 78.09409 0.03315 46.91077

6.27827E-4 80.31941 -- -- 5.61772E-4 56.62702 5.22138E-4 45.96545 0.00143 44.48415 0.01607 151.2338 0.01803 128.96144 0.02156 106.87603 0.02548 72.76273 0.03332 43.21923

6.19543E-4 78.38969 -- -- 5.47906E-4 55.57081 5.04923E-4 45.30231 0.00142 43.34035 0.01615 147.54042 0.01812 123.8636 0.02167 100.81432 0.02561 67.09034 0.03349 39.43929

6.11028E-4 77.30797 -- -- 5.33884E-4 54.86203 4.87597E-4 44.76137 0.00142 41.82683 0.01624 143.0307 0.01822 117.88219 0.02178 93.99675 0.02574 61.08196 0.03366 35.58164

6.02289E-4 75.67723 -- -- 5.19716E-4 53.5476 4.70173E-4 43.58926 0.00142 40.11378 0.01632 137.47587 0.01831 110.83041 0.02189 86.33399 0.02587 54.75389 0.03383 31.65713

5.93305E-4 74.2748 -- -- 5.17399E-4 52.97628 4.71856E-4 43.39194 0.00141 39.2221

5.84124E-4 72.8941 -- -- -- -- -- -- 0.00141 38.22479

5.7479E-4 70.9807 -- -- -- -- -- -- 0.0014 37.11023

5.65272E-4 69.19775 -- -- -- -- -- -- 0.00139 36.28905

5.55551E-4 67.39307 -- -- -- -- -- -- 0.00139 36.03097

5.45598E-4 66.06673 -- -- -- -- -- -- 0.00138 34.62303

5.35404E-4 64.91434 -- -- -- -- -- -- 0.00138 34.1772

5.24994E-4 63.18576 -- -- -- -- -- -- 0.00138 33.75485

5.14321E-4 61.43543 -- -- -- -- -- -- 0.00137 33.43791

5.03376E-4 59.6851 -- -- -- -- -- -- 0.00136 32.53455

4.92165E-4 57.61949 -- -- -- -- -- -- -- --

4.80651E-4 55.85829 -- -- -- -- -- -- -- --

4.68818E-4 54.44499 -- -- -- -- -- -- -- --

4.56664E-4 53.16214 -- -- -- -- -- -- -- --

**(b) *β*=45°**

1.00688E-8 0.20225 2.10672E-7 0.50368 3.81911E-7 0.29267 2.95011E-7 0.41295 4.13015E-7 0.38238 1E-4 1.77275 1.2E-4 1.68766 1.35E-4 1.65774 1.55E-4 2.56706 2.1E-4 2.48349

2.87605E-8 0.30816 4.93001E-7 1.30762 1.07303E-6 0.80496 8.04144E-7 0.80276 1.1258E-6 0.70203 2E-4 3.53934 2.4E-4 3.34864 2.7E-4 3.28657 3.1E-4 5.00689 4.2E-4 4.82589

8.60369E-8 0.54891 9.67392E-7 1.56912 2.03347E-6 1.39961 1.49174E-6 1.17198 2.08844E-6 0.98565 3E-4 5.29879 3.6E-4 4.98712 4.05E-4 4.89103 4.65E-4 7.35009 6.3E-4 7.06155

2.4607E-7 0.52946 1.66251E-6 2.43108 3.22903E-6 1.96678 2.31474E-6 1.67483 3.24064E-6 1.41439 4E-4 7.05053 4.8E-4 6.60505 5.4E-4 6.47327 6.2E-4 9.61019 8.4E-4 9.20573

4.93142E-7 0.22085 2.58264E-6 2.53765 4.6084E-6 2.48823 3.22569E-6 1.95624 4.51597E-6 1.61861 5E-4 8.79412 6E-4 8.20373 6.75E-4 8.03472 7.75E-4 11.79591 0.00105 11.26833

7.44501E-7 -0.07805 3.80946E-6 3.37061 6.11886E-6 3.47622 4.19373E-6 2.62115 5.87122E-6 2.14387 6E-4 10.5292 7.2E-4 9.78413 8.1E-4 9.57645 9.3E-4 13.91366 0.00126 13.25665

1.0187E-6 -0.30945 5.35734E-6 3.62251 7.79461E-6 3.93366 5.25458E-6 2.8494 7.35641E-6 2.30348 7E-4 12.2555 8.4E-4 11.34703 9.45E-4 11.09932 0.00109 15.9685 0.00147 15.17644

1.36003E-6 -0.34812 7.13312E-6 4.6491 9.65702E-6 4.39107 6.45297E-6 3.31892 9.03415E-6 2.71642 8E-4 13.97276 9.6E-4 12.89308 0.00108 12.60403 0.00124 17.96458 0.00168 17.03247

1.77707E-6 -0.10745 9.16975E-6 5.25933 1.16705E-5 4.56493 7.7642E-6 3.62862 1.08699E-5 3.01119 9E-4 15.68077 0.00108 14.42283 0.00121 14.09121 0.0014 19.90544 0.00189 18.82878

2.28407E-6 0.04645 1.14643E-5 6.60568 1.39361E-5 5.04974 9.24922E-6 4.12742 1.29489E-5 3.45009 1E-3 17.37933 0.0012 15.93677 0.00135 15.5614 0.00155 21.79413 0.0021 20.56889

2.80828E-6 -0.31056 1.39524E-5 7.77759 1.65068E-5 5.3791 1.09312E-5 4.59922 1.53037E-5 3.88784 0.0011 19.06828 0.00132 17.43532 0.00149 17.01507 0.0017 23.63336 0.00231 22.25592

3.27981E-6 -0.28186 1.66741E-5 9.13356 1.93367E-5 5.72669 1.27959E-5 4.7015 1.79142E-5 3.93441 0.0012 20.74745 0.00144 18.91888 0.00162 18.45266 0.00186 25.42556 0.00252 23.89264

3.71719E-6 -0.18594 1.96681E-5 10.82855 2.2483E-5 6.07429 1.4907E-5 5.26763 2.08699E-5 4.46801 0.0013 22.41672 0.00156 20.38781 0.00176 19.87456 0.00201 27.17293 0.00273 25.48159

4.19174E-6 -0.22463 2.2996E-5 12.63969 2.59157E-5 6.31221 1.72285E-5 5.6546 2.41199E-5 4.83269 0.0014 24.07595 0.00168 21.84242 0.00189 21.28115 0.00217 28.87745 0.00294 27.02507

4.71774E-6 0.2763 2.66221E-5 14.30569 2.95408E-5 7.10806 1.96795E-5 6.29945 2.75513E-5 5.37051 0.0015 25.72503 0.0018 23.28303 0.00202 22.67277 0.00232 30.54097 0.00315 28.52521

5.33084E-6 0.37249 3.05606E-5 16.3397 3.34124E-5 7.56544 2.22975E-5 6.99319 3.12165E-5 6.0189 0.0016 27.36385 0.00192 24.70991 0.00216 24.04972 0.00248 32.1652 0.00336 29.98395

6.03109E-6 0.34322 3.48603E-5 18.17992 3.75804E-5 8.08688 2.50954E-5 7.60585 3.51335E-5 6.57093 0.0017 28.99232 0.00204 26.12333 0.0023 25.41231 0.00264 33.75171 0.00357 31.40312

6.76147E-6 0.20772 3.95799E-5 20.25264 4.19863E-5 8.9743 2.80504E-5 8.61691 3.92706E-5 7.47726 0.0018 30.61034 0.00216 27.52355 0.00243 26.76082 0.00279 35.30198 0.00378 32.78441

7.51626E-6 0.506 4.47309E-5 21.83128 4.66588E-5 9.65123 3.12225E-5 9.54033 4.37115E-5 8.32838 0.0019 32.21783 0.00228 28.91079 0.00257 28.09553 0.00295 36.8174 0.00399 34.12943

8.38676E-6 0.2163 5.0256E-5 23.94282 5.1572E-5 10.75818 3.45925E-5 10.58163 4.84295E-5 9.22802 0.002 33.81472 0.0024 30.28529 0.0027 29.41667 0.0031 38.29926 0.0042 35.43965

9.3858E-6 0.28319 5.61552E-5 26.02517 5.6679E-5 11.70957 3.81395E-5 11.58003 5.33952E-5 10.10986 0.0021 35.40094 0.00252 31.64725 0.00283 30.7245 0.00326 39.7488 0.00441 36.7165

1.0529E-5 0.48528 6.23955E-5 28.00111 6.19656E-5 13.04514 4.18578E-5 12.8118 5.86009E-5 11.16949 0.0022 36.97642 0.00264 32.99689 0.00297 32.01924 0.00341 41.16717 0.00462 37.96129

1.18493E-5 0.42702 6.89625E-5 30.17072 6.75072E-5 13.81364 4.57796E-5 13.79055 6.40914E-5 12.06269 0.0023 38.54111 0.00276 34.33439 0.00311 33.30113 0.00357 42.55547 0.00483 39.17529

1.32169E-5 0.56135 7.58463E-5 32.24344 7.33467E-5 15.19501 4.99474E-5 15.23863 6.99264E-5 13.34144 0.0024 40.09496 0.00288 35.65994 0.00324 34.57036 0.00372 43.91475 0.00504 40.35969

1.46417E-5 0.82104 8.31718E-5 34.65518 7.94041E-5 16.71362 5.43716E-5 16.82915 7.61202E-5 14.74572 0.0025 41.63791 0.003 36.97372 0.00338 35.82715 0.00388 45.24601 0.00525 41.51561

1.61423E-5 0.86864 9.10606E-5 37.37853 8.57451E-5 18.16809 5.90901E-5 18.06849 8.27261E-5 15.7925 0.0026 43.16991 0.00312 38.27592 0.00351 37.0717 0.00403 46.55019 0.00546 42.64414

1.76689E-5 1.34978 9.93489E-5 40.16623 9.24168E-5 19.86963 6.41377E-5 19.5911 8.97928E-5 17.09348 0.0027 44.69094 0.00324 39.56668 0.00364 38.30419 0.00419 47.82819 0.00567 43.7463

1.92028E-5 1.71555 1.07884E-4 42.4229 9.94047E-5 21.38823 6.95451E-5 21.09519 9.73631E-5 18.40701 0.0028 46.20095 0.00336 40.84619 0.00378 39.52481 0.00434 49.08087 0.00588 44.82307

2.06613E-5 2.49568 1.16708E-4 45.00907 1.06726E-4 23.27281 7.53052E-5 22.64926 1.05427E-4 19.70898 0.0029 47.6999 0.00348 42.11459 0.00392 40.73375 0.00449 50.30907 0.00609 45.87538

2.20345E-5 3.47781 1.25825E-4 46.99452 1.14325E-4 24.75475 8.1381E-5 24.28241 1.13933E-4 21.16445 0.003 49.18778 0.0036 43.37203 0.00405 41.93117 0.00465 51.51358 0.0063 46.90412

2.33567E-5 4.28697 1.35194E-4 49.21259 1.22282E-4 26.55699 8.78116E-5 25.7851 1.22936E-4 22.42688 0.0031 50.66455 0.00372 44.61867 0.00419 43.11724 0.00481 52.69514 0.00651 47.91014

2.46634E-5 5.25011 1.44835E-4 51.46926 1.30604E-4 28.09379 9.4572E-5 27.75124 1.32401E-4 24.22239 0.0032 52.1302 0.00384 45.85465 0.00432 44.29213 0.00496 53.85449 0.00672 48.89426

2.59889E-5 5.97218 1.54857E-4 53.37728 1.39141E-4 29.99673 1.01596E-4 29.56889 1.42234E-4 25.79791 0.0033 53.58469 0.00396 47.0801 0.00445 45.45601 0.00511 54.99233 0.00693 49.85726

2.71806E-5 7.05116 1.65312E-4 54.75272 1.47883E-4 32.14647 1.08876E-4 31.56612 1.52426E-4 27.51879 0.0034 55.02802 0.00408 48.29516 0.00459 46.60901 0.00527 56.10933 0.00714 50.79989

2.81859E-5 7.85969 1.76131E-4 56.64139 1.56812E-4 34.1865 1.16402E-4 33.61405 1.62963E-4 29.31211 0.0035 56.46017 0.0042 49.49997 0.00473 47.75131 0.00543 57.20612 0.00735 51.72286

2.91344E-5 8.54295 1.87336E-4 57.98773 1.6592E-4 36.16248 1.242E-4 35.48739 1.73879E-4 30.93332 0.0036 57.88113 0.00432 50.69464 0.00486 48.88303 0.00558 58.28335 0.00756 52.62686

2.98166E-5 9.72737 1.99006E-4 59.67288 1.75225E-4 37.73604 1.32282E-4 37.08778 1.85195E-4 32.33836 0.0037 59.29088 0.00444 51.87932 0.005 50.00434 0.00573 59.34159 0.00777 53.51255

3.02395E-5 9.74527 2.11085E-4 60.87399 1.84773E-4 39.60223 1.4064E-4 38.73554 1.96896E-4 33.74193 0.0038 60.68944 0.00456 53.05411 0.00513 51.11536 0.00589 60.38143 0.00798 54.38057

3.0747E-5 10.16823 2.23548E-4 62.54 1.9461E-4 41.80691 1.49251E-4 40.67393 2.08952E-4 35.39142 0.0039 62.07678 0.00468 54.21914 0.00526 52.21625 0.00605 61.40341 0.00819 55.23153

3.13975E-5 11.79626 2.36416E-4 64.08954 2.04794E-4 43.97503 1.5818E-4 42.62211 2.21451E-4 37.05759 0.004 63.45291 0.0048 55.37452 0.0054 53.30713 0.0062 62.40809 0.0084 56.066

3.22196E-5 13.05794 2.49625E-4 65.56187 2.15297E-4 45.77717 1.67374E-4 44.25702 2.34324E-4 38.45887 0.0041 64.81783 0.00492 56.52037 0.00554 54.38813 0.00636 63.39596 0.00861 56.88456

3.32218E-5 14.58954 2.63176E-4 67.20841 2.26181E-4 47.87205 1.76865E-4 46.26124 2.47611E-4 40.19669 0.0042 66.17155 0.00504 57.65681 0.00567 55.45939 0.00651 64.36753 0.00882 57.68775

3.44356E-5 16.21736 2.77105E-4 68.6709 2.37464E-4 49.20782 1.8668E-4 47.64367 2.61352E-4 41.41449 0.0043 67.51406 0.00516 58.78393 0.00581 56.52103 0.00666 65.32327 0.00903 58.47608

3.58866E-5 17.78734 2.91388E-4 69.99789 2.49063E-4 51.22038 1.96717E-4 49.41137 2.75404E-4 42.91837 0.0044 68.84537 0.00528 59.90184 0.00594 57.57317 0.00682 66.26365 0.00924 59.25005

3.75235E-5 19.45401 3.05964E-4 72.02206 2.61041E-4 52.99505 2.07026E-4 51.26927 2.89836E-4 44.5586 0.0045 70.1655 0.0054 61.01066 0.00607 58.61594 0.00698 67.18912 0.00945 60.01015

3.93292E-5 21.17801 3.20745E-4 73.88174 2.7337E-4 54.45881 2.17578E-4 52.78241 3.04609E-4 45.89124 0.0046 71.47446 0.00552 62.11048 0.00621 59.64945 0.00713 68.10011 0.00966 60.75684

4.12424E-5 23.24919 3.35735E-4 76.09981 2.85985E-4 56.63601 2.28322E-4 54.78606 3.19652E-4 47.61406 0.0047 72.77225 0.00564 63.20139 0.00634 60.67383 0.00728 68.99704 0.00987 61.49057

4.33244E-5 24.80953 3.51014E-4 77.85287 2.99009E-4 58.76746 2.3934E-4 56.84479 3.35076E-4 49.40272 0.0048 74.05889 0.00576 64.28351 0.00648 61.68918 0.00744 69.8803 0.01008 62.21177

4.5635E-5 26.96768 3.66523E-4 79.58647 3.12531E-4 61.6415 2.50643E-4 59.5248 3.509E-4 51.71377 0.0049 75.3344 0.00588 65.35692 0.00662 62.69562 0.0076 70.75029 0.01029 62.92084

4.82441E-5 28.48955 3.82191E-4 81.68838 3.26428E-4 64.13753 2.62191E-4 61.78838 3.67067E-4 53.65373 0.005 76.5988 0.006 66.42173 0.00675 63.69326 0.00775 71.60738 0.0105 63.6182

5.12403E-5 29.84763 3.98021E-4 83.95479 3.40585E-4 65.95796 2.73961E-4 63.60384 3.83545E-4 55.24139 0.0051 77.8521 0.00612 67.47801 0.00688 64.6822 0.00791 72.45194 0.01071 64.30421

5.45649E-5 31.87091 4.14009E-4 85.90163 3.5504E-4 68.29072 2.8598E-4 65.69549 4.00372E-4 57.02939 0.0052 79.09433 0.00624 68.52587 0.00702 65.66256 0.00806 73.28432 0.01092 64.97926

5.82279E-5 33.85583 4.30216E-4 87.84837 3.69808E-4 70.32166 2.98303E-4 67.67905 4.17624E-4 58.75671 0.0053 80.3255 0.00636 69.56538 0.00715 66.63443 0.00822 74.10485 0.01113 65.64369

6.22266E-5 35.21379 4.46624E-4 89.78549 3.84833E-4 72.62684 3.10929E-4 69.4254 4.35301E-4 60.18697 0.0054 81.54565 0.00648 70.59665 0.00729 67.59792 0.00837 74.91387 0.01134 66.29786

6.65352E-5 37.14102 4.63209E-4 91.47075 4.00072E-4 74.62122 3.23826E-4 71.11257 4.53357E-4 61.60949 0.0055 82.7548 0.0066 71.61975 0.00743 68.55313 0.00852 75.7117 0.01155 66.94208

7.10938E-5 38.94269 4.79936E-4 93.42732 4.15483E-4 76.36851 3.36988E-4 72.86958 4.71784E-4 63.14857 0.0056 83.95297 0.00672 72.63476 0.00756 69.50015 0.00868 76.49864 0.01176 67.57668

7.59507E-5 40.94697 4.96687E-4 95.18039 4.31056E-4 78.00611 3.50466E-4 74.4651 4.90652E-4 64.53728 0.0057 85.1402 0.00684 73.64177 0.00769 70.43909 0.00884 77.27498 0.01197 68.20196

8.11903E-5 42.36336 5.13438E-4 97.53395 4.46818E-4 79.58866 3.64287E-4 75.75137 5.10003E-4 65.61092 0.0058 86.31651 0.00696 74.64086 0.00783 71.37004 0.00899 78.04102 0.01218 68.81822

8.67668E-5 44.0495 5.30075E-4 99.70357 4.62763E-4 81.31767 3.78441E-4 77.28239 5.29817E-4 66.91592 0.0059 87.48193 0.00708 75.63211 0.00796 72.29309 0.00914 78.79703 0.01239 69.42574

9.26702E-5 45.81306 5.4662E-4 101.69875 4.78943E-4 82.98265 3.92939E-4 78.78781 5.50115E-4 68.20524 0.006 88.6365 0.0072 76.61559 0.0081 73.20833 0.0093 79.54328 0.0126 70.02479

9.89547E-5 47.27771 5.6308E-4 103.63587 4.95409E-4 83.83347 4.07847E-4 79.88882 5.70986E-4 69.2124 0.0061 89.78025 0.00732 77.59138 0.00823 74.11586 0.00945 80.28002 0.01281 70.61564

1.05519E-4 49.27256 5.79358E-4 105.51491 5.12078E-4 85.05016 4.23173E-4 81.28084 5.92442E-4 70.4611 0.0062 90.91321 0.00744 78.55955 0.00837 75.01576 0.00961 81.00752 0.01302 71.19854

1.12353E-4 50.09126 5.95452E-4 107.31642 5.29009E-4 86.18451 4.38898E-4 82.78617 6.14457E-4 71.84319 0.0063 92.03542 0.00756 79.52019 0.0085 75.90811 0.00976 81.726 0.01323 71.77372

1.19572E-4 51.92219 6.11271E-4 109.14702 5.46118E-4 88.12394 4.54919E-4 84.10867 6.36887E-4 72.89242 0.0064 93.14691 0.00768 80.47335 0.00864 76.79301 0.00992 82.43571 0.01344 72.34143

1.2717E-4 53.3 6.26782E-4 110.7064 5.63269E-4 89.15759 4.71212E-4 85.09902 6.59697E-4 73.75139 0.0065 94.24772 0.0078 81.41911 0.00877 77.67054 0.01008 83.13687 0.01365 72.90189

1.35164E-4 55.08279 6.4196E-4 112.14953 5.80546E-4 91.26166 4.87862E-4 86.62808 6.83007E-4 74.9887 0.0066 95.33789 0.00792 82.35753 0.00891 78.54078 0.01023 83.8297 0.01386 73.45532

1.43576E-4 56.38366 6.56715E-4 113.60239 5.97874E-4 92.89916 5.04763E-4 87.73322 7.06667E-4 75.86253 0.0067 96.41746 0.00804 83.28869 0.00904 79.4038 0.01039 84.5144 0.01407 74.00192

1.52424E-4 57.66505 6.71005E-4 115.02617 6.15158E-4 94.69221 5.21851E-4 89.07816 7.30591E-4 76.96094 0.0068 97.48647 0.00816 84.21265 0.00918 80.2597 0.01054 85.19119 0.01428 74.54188

1.61624E-4 59.29361 6.84851E-4 116.20771 6.32417E-4 96.83295 5.39175E-4 90.50714 7.54846E-4 78.08673 0.0069 98.54495 0.00828 85.12947 0.00931 81.10854 0.0107 85.86025 0.01449 75.07542

1.71099E-4 61.28833 6.98283E-4 117.08916 6.49621E-4 98.2966 5.56712E-4 91.49045 7.79397E-4 78.86307 0.007 99.59295 0.0084 86.03922 0.00945 81.9504 0.01085 86.52177 0.0147 75.60269

1.8088E-4 62.84928 7.1135E-4 118.38705 6.66692E-4 100.31825 5.74391E-4 92.72767 8.04147E-4 79.80836 0.0071 100.63052 0.00852 86.94196 0.00958 82.78537 0.011 87.17594 0.01491 76.12389

1.90933E-4 64.91174 7.24089E-4 119.67522 6.8364E-4 101.76372 5.92216E-4 93.81415 8.29102E-4 80.6962 0.0072 101.65768 0.00864 87.83774 0.00972 83.6135 0.01116 87.82294 0.01512 76.63918

2.01309E-4 67.15687 7.36525E-4 120.83751 7.00516E-4 103.68486 6.10183E-4 95.18013 8.54256E-4 81.79429 0.0073 102.6745 0.00876 88.72664 0.00985 84.43488 0.01132 88.46292 0.01533 77.14872

2.1219E-4 70.15355 7.48739E-4 122.30011 7.17276E-4 104.82842 6.28241E-4 96.04229 8.79537E-4 82.49943 0.0074 103.681 0.00888 89.60869 0.00999 85.24957 0.01147 89.09606 0.01554 77.65267

2.23634E-4 72.554 7.60731E-4 123.65608 7.33878E-4 106.34683 6.46339E-4 97.20927 9.04875E-4 83.45904 0.0075 104.67724 0.009 90.48397 0.01013 86.05764 0.01163 89.72251 0.01575 78.15117

2.35517E-4 73.58475 7.72502E-4 125.25407 7.50288E-4 107.91129 6.64444E-4 98.34556 9.30222E-4 84.37837 0.0076 105.66326 0.00912 91.35253 0.01026 86.85916 0.01178 90.34242 0.01596 78.64437

2.47581E-4 75.17448 7.84031E-4 126.5617 7.66522E-4 109.35667 6.82571E-4 99.52454 9.55599E-4 85.36335 0.0077 106.63911 0.00924 92.21441 0.0104 87.6542 0.01193 90.95593 0.01617 79.13239

2.59728E-4 78.13355 7.95302E-4 127.80152 7.82665E-4 110.8478 7.00698E-4 100.50925 9.80977E-4 86.13635 0.0078 107.60482 0.00936 93.06968 0.01053 88.44283 0.01209 91.56318 0.01638 79.61537

2.72014E-4 80.77411 8.06352E-4 128.57624 7.98632E-4 112.55843 7.18729E-4 101.78535 0.00101 87.17689 0.0079 108.56046 0.00948 93.91837 0.01066 89.22509 0.01225 92.16432 0.01659 80.09344

2.8436E-4 83.53056 8.1724E-4 129.95167 8.14342E-4 113.84845 7.36583E-4 102.81491 0.00103 88.03218 0.008 109.50605 0.0096 94.76055 0.0108 90.00107 0.0124 92.75945 0.0168 80.5667

2.96757E-4 86.50861 8.27985E-4 130.85238 8.29798E-4 115.46766 7.54239E-4 103.76617 0.00106 88.74763 0.0081 110.44166 0.00972 95.59626 0.01094 90.77081 0.01256 93.34871 0.01701 81.03526

3.09235E-4 88.6866 8.38569E-4 132.08247 8.44972E-4 116.94051 7.71668E-4 104.55454 0.00108 89.31768 0.0082 111.36732 0.00984 96.42554 0.01107 91.53438 0.01271 93.93221 0.01722 81.49924

3.21737E-4 91.10568 8.49058E-4 133.04135 8.59927E-4 118.58908 7.88925E-4 105.28 0.0011 89.79091 0.0083 112.28308 0.00996 97.24844 0.01121 92.29184 0.01287 94.51007 0.01743 81.95873

3.34277E-4 93.38999 8.59358E-4 134.16492 8.74655E-4 119.91754 8.06016E-4 106.05933 0.00113 90.37673 0.0084 113.189 0.01008 98.06501 0.01134 93.04324 0.01302 95.08238 0.01764 82.41382

3.46802E-4 95.70298 8.69493E-4 135.1238 8.89139E-4 121.4399 8.2293E-4 106.8929 0.00115 90.98557 0.0085 114.08511 0.0102 98.87529 0.01148 93.78864 0.01317 95.64925 0.01785 82.8646

3.59299E-4 98.33447 8.79475E-4 135.69522 9.03405E-4 122.85978 8.39722E-4 107.71105 0.00118 91.59614 0.0086 114.97146 0.01032 99.67931 0.01161 94.52809 0.01333 96.21078 0.01806 83.31116

3.71813E-4 100.58964 8.8932E-4 136.7897 9.17433E-4 124.22658 8.56404E-4 108.32151 0.0012 91.99793 0.0087 115.84811 0.01044 100.47712 0.01175 95.26165 0.01349 96.76706 0.01827 83.75357

3.84301E-4 103.06653 8.99172E-4 137.74858 9.31299E-4 125.5751 8.73027E-4 109.08498 0.00122 92.56359 0.0088 116.71509 0.01056 101.26876 0.01188 95.98936 0.01364 97.31816 0.01848 84.1919

3.96784E-4 105.71701 9.08944E-4 138.52341 9.44982E-4 126.6474 8.89629E-4 109.59845 0.00125 92.91508 0.0089 117.57246 0.01068 102.05426 0.01202 96.71128 0.0138 97.86418 0.01869 84.62622

4.09221E-4 108.24725 9.18582E-4 139.86002 9.58468E-4 128.1332 9.06254E-4 110.53786 0.00127 93.64144 0.009 118.42026 0.0108 102.83366 0.01215 97.42745 0.01395 98.40519 0.0189 85.0566

4.21594E-4 109.91005 9.28153E-4 139.92772 9.71849E-4 129.49634 9.22955E-4 111.534 0.00129 94.44884 0.0091 119.25854 0.01092 103.60699 0.01229 98.13792 0.0141 98.94126 0.01911 85.48309

4.3397E-4 112.26223 9.37656E-4 140.83826 9.84975E-4 130.77717 9.39657E-4 112.3999 0.00132 95.13389 0.0092 120.08734 0.01104 104.37428 0.01242 98.84274 0.01426 99.47246 0.01932 85.90574

4.46344E-4 114.20954 9.47104E-4 141.34197 9.97708E-4 131.3486 9.56314E-4 112.83821 0.00134 95.49411 0.0093 120.9067 0.01116 105.13556 0.01256 99.54194 0.01442 99.99884 0.01953 86.3246

4.58684E-4 116.22422 9.56435E-4 141.90376 0.00101 131.93209 9.729E-4 113.53658 0.00136 96.12529 0.0094 121.71668 0.01128 105.89087 0.01269 100.23558 0.01457 100.52048 0.01974 86.73972

4.71013E-4 118.25802 9.65622E-4 142.47507 0.00102 132.46596 9.88469E-4 114.36752 0.00138 96.90435 0.0095 122.51732 0.0114 106.64022 0.01283 100.9237 0.01473 101.03741 0.01995 87.15112

4.83321E-4 119.81001 9.74634E-4 142.39764 0.00103 132.48395 0.001 114.75635 0.00141 97.30947 0.0096 123.30866 0.01152 107.38366 0.01296 101.60632 0.01488 101.54969 0.02016 87.55885

4.95552E-4 121.60333 9.83443E-4 142.80436 0.00105 133.40236 0.00102 115.71744 0.00143 98.1579 0.0097 124.09075 0.01164 108.12118 0.0131 102.2835 0.01504 102.05736 0.02037 87.96293

5.07661E-4 123.58898 9.92024E-4 143.15321 0.00106 134.06442 0.00104 116.58743 0.00146 98.95553 0.0098 124.86362 0.01176 108.85283 0.01323 102.95526 0.01519 102.56046 0.02058 88.36339

5.19609E-4 125.33391 0.001 143.45341 0.00107 134.67172 0.00105 117.36841 0.00148 99.66928 0.0099 125.62732 0.01188 109.57862 0.01337 103.62164 0.01534 103.05902 0.02079 88.76024

5.31411E-4 127.06893 0.00101 143.25 0.00108 135.22764 0.00108 118.0262 0.00151 100.26268 0.01 126.38189 0.012 110.29857 0.0135 104.28267 0.0155 103.55307 0.021 89.1535

5.4305E-4 129.08361 0.00102 143.18219 0.00109 135.80381 0.00109 118.70798 0.00153 100.87771 0.0101 127.12737 0.01212 111.0127 0.01364 104.9384 0.01565 104.04265 0.02121 89.54318

5.54522E-4 130.13456 0.00102 143.55993 0.0011 135.99762 0.00111 119.39029 0.00155 101.56022 0.0102 127.86379 0.01224 111.72101 0.01377 105.58883 0.01581 104.52775 0.02142 89.92928

5.65798E-4 131.54164 0.00103 143.45341 0.00111 136.31328 0.00112 119.92154 0.00157 102.0628 0.0103 128.59121 0.01236 112.42353 0.01391 106.23401 0.01597 105.00841 0.02163 90.3118

5.76862E-4 132.99746 0.00104 143.09503 0.00112 136.8172 0.00113 120.40491 0.00159 102.48215 0.0104 129.30964 0.01248 113.12026 0.01404 106.87395 0.01612 105.48464 0.02184 90.69074

5.87805E-4 134.34901 0.00104 143.04659 0.00113 136.96545 0.00115 120.60732 0.00161 102.66873 0.0105 130.01913 0.0126 113.81121 0.01418 107.50869 0.01628 105.95643 0.02205 91.06608

5.98621E-4 135.48863 0.00105 142.59142 0.00114 137.62603 0.00117 121.23319 0.00163 103.21029 0.0106 130.71971 0.01272 114.49639 0.01431 108.13825 0.01643 106.42379 0.02226 91.43782

6.09323E-4 136.65909 0.00106 142.46544 0.00115 137.91618 0.00119 121.30297 0.00166 103.23278 0.0107 131.41142 0.01284 115.1758 0.01445 108.76263 0.01658 106.88672 0.02247 91.80594

6.19931E-4 137.7219 0.00106 142.28149 0.00115 138.219 0.0012 121.39111 0.00167 103.27234 0.0108 132.09428 0.01296 115.84944 0.01458 109.38187 0.01674 107.3452 0.02268 92.1704

6.30536E-4 138.74817 0.00107 141.94247 0.00116 138.34707 0.00121 121.50619 0.00169 103.37076 0.0109 132.76832 0.01308 116.51732 0.01472 109.99598 0.0169 107.79922 0.02289 92.53118

6.41147E-4 138.99361 0.00107 142.1845 0.00117 138.38365 0.00123 121.39729 0.00171 103.25001 0.011 133.43357 0.0132 117.17943 0.01485 110.60498 0.01705 108.24876 0.0231 92.88824

6.51684E-4 140.16251 0.00108 141.46785 0.00118 138.50264 0.00124 121.5002 0.00174 103.33725 0.0111 134.09005 0.01332 117.83575 0.01499 111.20887 0.01721 108.6938 0.02331 93.24155

6.62171E-4 140.95737 0.00108 141.21599 0.00119 138.36536 0.00126 121.19448 0.00176 103.04026 0.0112 134.73779 0.01344 118.4863 0.01512 111.80766 0.01736 109.13431 0.02352 93.59106

6.72607E-4 142.16304 0.00109 140.5865 0.00119 137.70667 0.00127 120.55334 0.00177 102.48234 0.0113 135.37681 0.01356 119.13104 0.01525 112.40137 0.01751 109.57025 0.02373 93.93671

6.83004E-4 143.38794 0.00109 140.16995 0.0012 137.54202 0.00128 120.31926 0.00178 102.26537 0.0114 136.00712 0.01368 119.76998 0.01539 112.98999 0.01767 110.00158 0.02394 94.27845

6.93323E-4 144.08627 0.00109 139.66631 0.00121 137.55121 0.00129 120.14036 0.00181 102.07591 0.0115 136.62875 0.0138 120.40308 0.01553 113.57354 0.01783 110.42824 0.02415 94.61623

7.0352E-4 144.96025 0.0011 138.87219 0.00121 136.56321 0.0013 119.5468 0.00182 101.62558 0.0116 137.2417 0.01392 121.03034 0.01566 114.152 0.01798 110.8502 0.02436 94.94996

7.13646E-4 145.74738 0.0011 137.96166 0.00122 136.43513 0.00132 119.56877 0.00184 101.67106 0.0117 137.84599 0.01404 121.65173 0.0158 114.72538 0.01813 111.26739 0.02457 95.27958

7.23641E-4 146.06406 0.0011 137.02209 0.00123 133.65408 0.00133 117.94345 0.00186 100.45115 0.0118 138.44162 0.01416 122.26721 0.01593 115.29367 0.01829 111.67975 0.02478 95.605

7.33467E-4 147.04594 0.00111 136.36353 0.00123 132.19043 0.00134 117.12791 0.00188 99.85098 0.0119 139.0286 0.01428 122.87677 0.01606 115.85687 0.01845 112.08719 0.02499 95.92614

7.43157E-4 147.62295 0.00111 135.37558 0.00124 131.37627 0.00135 116.48392 0.00189 99.31727 0.012 139.60692 0.0144 123.48036 0.0162 116.41496 0.0186 112.48965 0.0252 96.24291

7.52668E-4 148.38322 0.00111 134.38764 0.00124 131.25728 0.00136 116.36878 0.0019 99.2172 0.0121 140.1766 0.01452 124.07795 0.01633 116.96792 0.01876 112.88703 0.02541 96.5552

7.62021E-4 149.24933 0.00111 133.44818 0.00125 130.78162 0.00137 115.93547 0.00193 98.84546 0.0122 140.73761 0.01464 124.66949 0.01647 117.51574 0.01891 113.27925 0.02562 96.86291

7.712E-4 149.45401 0.00111 132.09215 0.00125 130.61696 0.00138 115.77724 0.00193 98.70813 0.0123 141.28995 0.01476 125.25494 0.01661 118.0584 0.01906 113.6662 0.02583 97.16592

7.80294E-4 150.00598 0.00111 130.98796 0.00125 130.35171 0.00139 115.40189 0.00195 98.36044 0.0124 141.83361 0.01488 125.83425 0.01674 118.59587 0.01922 114.04778 0.02604 97.46411

7.89298E-4 149.61332 0.00111 129.81601 0.00126 129.85767 0.0014 115.14078 0.00196 98.17273 0.0125 142.36857 0.015 126.40736 0.01688 119.12812 0.01938 114.42387 0.02625 97.75734

7.98076E-4 150.1942 0.00111 128.90562 0.00126 129.51917 0.00141 114.594 0.00197 97.65784 0.0126 142.89479 0.01512 126.9742 0.01701 119.65512 0.01953 114.79434 0.02646 98.04549

8.06636E-4 150.63282 0.00111 127.5109 0.00126 128.95199 0.00142 114.28543 0.00198 97.4331 0.0127 143.41226 0.01524 127.53473 0.01715 120.17683 0.01969 115.15905 0.02667 98.32839

8.14968E-4 151.38136 0.00111 126.43579 0.00126 128.62265 0.00143 113.91481 0.00199 97.10159 0.0128 143.92095 0.01536 128.08885 0.01728 120.69322 0.01984 115.51788 0.02688 98.60591

8.23145E-4 152.02756 0.00111 125.59312 0.00127 127.76274 0.00144 113.50711 0.00201 96.82399 0.0129 144.4208 0.01548 128.6365 0.01742 121.20423 0.01999 115.87065 0.02709 98.87785

8.31205E-4 152.83778 0.00111 124.24678 0.00127 127.43339 0.00145 113.12198 0.00202 96.47724 0.013 144.91177 0.0156 129.1776 0.01755 121.70981 0.02015 116.21722 0.0273 99.14406

8.39037E-4 153.18536 0.00111 122.97801 0.00127 126.18917 0.00145 112.50495 0.00203 96.04709 0.0131 145.39382 0.01572 129.71204 0.01768 122.20991 0.02031 116.5574 0.02751 99.40435

8.46692E-4 153.23395 0.00111 121.91264 0.00127 126.05199 0.00145 112.17277 0.00204 95.72231 0.0132 145.86688 0.01584 130.23974 0.01782 122.70447 0.02046 116.89101 0.02772 99.65851

8.54149E-4 153.92852 0.0011 120.01417 0.00127 125.66784 0.00146 112.02425 0.00204 95.63359 0.0133 146.33089 0.01596 130.76059 0.01795 123.19341 0.02062 117.21786 0.02793 99.90634

8.61379E-4 153.71695 0.0011 118.24175 0.00127 124.47854 0.00147 111.04154 0.00206 94.80987 0.0134 146.78578 0.01608 131.27447 0.01809 123.67667 0.02077 117.53773 0.02814 100.14762

8.68436E-4 153.88149 0.0011 116.87604 0.00126 124.08524 0.00147 110.63338 0.00206 94.45013 0.0135 147.23145 0.0162 131.78128 0.01823 124.15417 0.02092 117.8504 0.02835 100.38211

8.75277E-4 153.97856 0.0011 115.07465 0.00126 121.34992 0.00148 109.13871 0.00207 93.3594 0.0136 147.66783 0.01632 132.28087 0.01836 124.62581 0.02108 118.15564 0.02856 100.60957

8.81888E-4 153.3623 0.00109 113.45713 0.00126 120.8284 0.00148 108.57668 0.00207 92.86054 0.0137 148.0948 0.01644 132.77312 0.0185 125.09151 0.02124 118.45319 0.02877 100.82973

8.88266E-4 153.13157 0.00109 111.95587 0.00126 120.3253 0.00149 108.30136 0.00208 92.6595 0.0138 148.51227 0.01656 133.25787 0.01863 125.55116 0.02139 118.74279 0.02898 101.04232

8.9441E-4 152.61189 0.00109 110.55142 0.00125 119.91367 0.00149 107.64326 0.00208 92.04053 0.0139 148.9201 0.01668 133.73497 0.01877 126.00465 0.02155 119.02416 0.02919 101.24704

9.00263E-4 151.62928 0.00108 108.43026 0.00125 119.1543 0.00149 107.18549 0.00209 91.69276 0.014 149.31818 0.0168 134.20424 0.0189 126.45186 0.0217 119.29699 0.0294 101.44359

9.05777E-4 150.65629 0.00108 107.13239 0.00125 118.46813 0.0015 106.4916 0.00209 91.08426 0.0141 149.70634 0.01692 134.66551 0.01904 126.89267 0.02185 119.56097 0.02961 101.63163

9.1096E-4 150.18474 0.00107 105.53432 0.00124 117.6174 0.0015 105.72403 0.00209 90.42718 0.0142 150.08445 0.01704 135.11859 0.01917 127.32694 0.02201 119.81576 0.02982 101.81082

9.15838E-4 148.90325 0.00107 103.7231 0.00124 116.74845 0.0015 104.79414 0.0021 89.60286 0.0143 150.45232 0.01716 135.56325 0.0193 127.75451 0.02217 120.061 0.03003 101.9808

9.20417E-4 148.09412 0.00106 102.72554 0.00124 115.75121 0.00151 103.96673 0.00211 88.90861 0.0144 150.80977 0.01728 135.99929 0.01944 128.17523 0.02232 120.2963 0.03024 102.14117

9.24647E-4 146.95749 0.00105 101.22413 0.00123 115.16575 0.0015 103.28636 0.0021 88.29668 0.0145 151.15659 0.0174 136.42646 0.01957 128.58892 0.02247 120.52126 0.03045 102.29152

9.28548E-4 145.93627 0.00105 99.74228 0.00123 113.92163 0.00151 102.28024 0.00211 87.45796 0.0146 151.49257 0.01752 136.84451 0.01971 128.99539 0.02263 120.73546 0.03066 102.43142

9.32092E-4 144.28873 0.00104 98.26033 0.00122 113.00677 0.00151 101.36743 0.00211 86.65962 0.0147 151.81748 0.01764 137.25317 0.01985 129.39446 0.02279 120.93842 0.03087 102.56041

9.35273E-4 143.08475 0.00103 96.85602 0.00122 112.17438 0.00151 100.55531 0.00211 85.95256 0.0148 152.13104 0.01776 137.65215 0.01998 129.78589 0.02294 121.12967 0.03108 102.678

9.38135E-4 141.84179 0.00103 95.19974 0.00121 110.82039 0.00151 99.33935 0.00211 84.91275 0.0149 152.43298 0.01788 138.04113 0.02012 130.16947 0.0231 121.30869 0.03129 102.78366

9.40678E-4 140.91743 0.00102 93.97938 0.00121 109.71349 0.00151 98.28819 0.00211 84.00274 0.015 152.72301 0.018 138.41978 0.02025 130.54493 0.02325 121.47491 0.0315 102.87685

9.42948E-4 139.68428 0.00101 92.09061 0.0012 108.50591 0.00151 97.15954 0.00211 83.02898 0.0151 153.00078 0.01812 138.78773 0.02039 130.91201 0.0234 121.62776 0.03171 102.95697

9.44981E-4 138.59565 0.001 90.5894 0.00119 107.20684 0.00151 95.97763 0.00211 82.01532 0.0152 153.26595 0.01824 139.14461 0.02052 131.27041 0.02356 121.76661 0.03192 103.02341

9.46735E-4 137.21779 9.96227E-4 88.73945 0.00119 106.12737 0.00151 95.02443 0.00211 81.20336 0.0153 153.51812 0.01836 139.49 0.02066 131.61983 0.02372 121.89078 0.03213 103.07551

9.48157E-4 136.29318 9.87743E-4 87.28669 0.00118 105.18519 0.0015 93.95456 0.0021 80.24488 0.0154 153.75689 0.01848 139.82345 0.02079 131.95992 0.02387 121.99956 0.03234 103.11255

9.49261E-4 135.32028 9.79066E-4 85.8241 0.00117 104.16056 0.0015 92.90268 0.0021 79.31971 0.0155 153.9818 0.0186 140.14449 0.02092 132.29033 0.02403 122.09219 0.03255 103.13379

9.50074E-4 134.12551 9.70198E-4 84.25509 0.00116 103.06279 0.0015 92.00378 0.00209 78.56798 0.0156 154.19235 0.01872 140.4526 0.02106 132.61064 0.02418 122.16786 0.03276 103.13842

9.50628E-4 133.19122 9.61171E-4 82.8603 0.00116 101.79118 0.0015 90.62742 0.00209 77.34533 0.0157 154.38802 0.01884 140.74722 0.02119 132.92045 0.02433 122.22568 0.03297 103.1256

9.50968E-4 132.29528 9.52008E-4 81.50433 0.00115 100.77573 0.00149 89.87698 0.00209 76.73508 0.0158 154.56823 0.01896 141.02776 0.02133 133.21929 0.02449 122.26472 0.03318 103.0944

9.51018E-4 131.18752 9.42685E-4 80.09991 0.00114 99.65966 0.00149 88.7622 0.00208 75.75987 0.0159 154.73236 0.01908 141.29357 0.02147 133.50665 0.02465 122.28398 0.03339 103.04385

9.50723E-4 130.08916 9.33239E-4 78.73421 0.00113 97.96731 0.00148 87.28664 0.00207 74.50669 0.016 154.87971 0.0192 141.54395 0.0216 133.782 0.0248 122.28238 0.0336 102.97292

9.50165E-4 128.55689 9.23716E-4 77.4654 0.00112 97.02497 0.00148 86.318 0.00207 73.65453 0.0161 155.00955 0.01932 141.77816 0.02174 134.04475 0.02496 122.25877 0.03381 102.88048

9.49324E-4 127.47781 9.14092E-4 76.2644 0.00111 95.58879 0.00147 85.08665 0.00206 72.61294 0.0162 155.12108 0.01944 141.99536 0.02187 134.29427 0.02511 122.2119 0.03402 102.76533

9.48191E-4 126.26401 9.04402E-4 74.95687 0.0011 93.96948 0.00146 83.50075 0.00205 71.23113 0.0163 155.2134 0.01956 142.19469 0.02201 134.52984 0.02526 122.14043 0.03423 102.6262

9.46799E-4 124.93438 8.94657E-4 73.86239 0.00109 92.22228 0.00146 82.20952 0.00204 70.1811 0.0164 155.28558 0.01968 142.37517 0.02214 134.75073 0.02542 122.04293 0.03444 102.46169

9.45109E-4 123.60466 8.84781E-4 72.72918 0.00108 91.02378 0.00145 81.00242 0.00204 69.12338 0.0165 155.33655 0.0198 142.53577 0.02228 134.95609 0.02558 121.91782 0.03465 102.27033

9.43058E-4 122.16884 8.74783E-4 71.88656 0.00107 89.85285 0.00145 79.88471 0.00203 68.15469 0.0166 155.36518 0.01992 142.67534 0.02241 135.14504 0.02573 121.76344 0.03486 102.05052

9.40694E-4 121.11862 8.64767E-4 70.76298 0.00106 88.52637 0.00145 78.57212 0.00202 67.00861 0.0167 155.3702 0.02004 142.79265 0.02254 135.31659 0.02588 121.57796 0.03507 101.80054

9.38014E-4 119.18159 8.54852E-4 69.65876 0.00105 87.44696 0.00144 77.2116 0.00201 65.76896 0.0168 155.35025 0.02016 142.88635 0.02268 135.46964 0.02604 121.3594 0.03528 101.51853

9.34966E-4 117.85194 8.45058E-4 68.36097 0.00103 86.22108 0.00143 75.93984 0.00199 64.64814 0.0169 155.30382 0.02028 142.95494 0.02281 135.60303 0.0262 121.10564 0.03549 101.20249

9.31632E-4 116.36793 8.35326E-4 67.2374 0.00102 84.58358 0.00142 74.49368 0.00199 63.41624 0.017 155.22925 0.0204 142.99682 0.02295 135.71543 0.02635 120.81433 0.0357 100.85023

9.28075E-4 115.05751 8.25623E-4 65.804 0.00101 83.11064 0.00142 73.06993 0.00198 62.17907 0.0171 155.1247 0.02052 143.01019 0.02309 135.80541 0.02651 120.48297 0.03591 100.45942

9.24161E-4 113.37088 8.15973E-4 64.8742 9.95983E-4 81.48233 0.0014 71.38314 0.00196 60.69289 0.0172 154.98816 0.02064 142.99311 0.02322 135.87138 0.02666 120.10877 0.03612 100.0275

9.199E-4 111.44317 8.06363E-4 63.46979 9.8302E-4 80.15595 0.0014 70.12104 0.00196 59.5998 0.0173 154.81739 0.02076 142.94341 0.02336 135.91157 0.02681 119.68875 0.03633 99.5517

9.15314E-4 109.86312 7.96849E-4 62.33659 9.69882E-4 78.51844 0.00138 68.77361 0.00194 58.47156 0.0174 154.60992 0.02088 142.85871 0.02349 135.92404 0.02697 119.2196 0.03654 99.029

9.10325E-4 107.80996 7.87322E-4 60.4769 9.56505E-4 77.23762 0.00138 67.56861 0.00193 57.43046 0.0175 154.363 0.021 142.73637 0.02363 135.9066 0.02713 118.69772 0.03675 98.45614

9.05054E-4 106.15225 7.77743E-4 59.40177 9.42878E-4 76.10326 0.00137 66.39529 0.00192 56.39699 0.0176 154.07356 0.02112 142.57346 0.02376 135.85684 0.02728 118.11916 0.03696 97.82952

8.99588E-4 104.96715 7.68205E-4 58.02645 9.29066E-4 75.05123 0.00136 65.48949 0.0019 55.63 0.0177 153.73818 0.02124 142.36672 0.0239 135.77204 0.02744 117.47957 0.03717 97.14524

8.9398E-4 103.75301 7.58674E-4 56.67518 9.14992E-4 73.79787 0.00135 64.54606 0.00189 54.85873 0.0178 153.35303 0.02136 142.11251 0.02403 135.64915 0.02759 116.77415 0.03738 96.39902

8.88256E-4 102.68331 7.49078E-4 55.68251 9.00734E-4 72.39826 0.00134 63.58045 0.00188 54.08978 0.0179 152.9138 0.02148 141.80678 0.02416 135.48475 0.02774 115.99762 0.03759 95.58619

8.82352E-4 101.40192 7.39408E-4 53.83255 8.8639E-4 71.50168 0.00133 62.74687 0.00187 53.37142 0.018 152.41567 0.0216 141.44495 0.0243 135.27498 0.0279 115.14413 0.0378 94.7016

8.76238E-4 99.4551 7.29686E-4 52.79624 8.71987E-4 70.56863 0.00132 62.03234 0.00185 52.78444 0.0181 151.85314 0.02172 141.02191 0.02443 135.01544 0.02806 114.20722 0.03801 93.73962

8.69853E-4 97.97085 7.19899E-4 50.8495 8.57578E-4 70.2759 0.00131 61.71815 0.00184 52.50577 0.0182 151.22002 0.02184 140.53185 0.02457 134.70115 0.02821 113.17969 0.03822 92.69403

8.6324E-4 96.57357 7.1004E-4 49.69673 8.43093E-4 69.48913 0.0013 60.86146 0.00182 51.74393 0.0183 150.50926 0.02196 139.96821 0.02471 134.32641 0.02837 112.05354 0.03843 91.55802

8.56428E-4 95.23424 7.00115E-4 47.90506 8.28483E-4 68.23587 0.00129 59.84892 0.00181 50.90009 0.0184 149.7128 0.02208 139.32354 0.02484 133.8847 0.02852 110.81987 0.03864 90.32407

8.49371E-4 93.72096 6.90113E-4 46.65561 8.13732E-4 67.07404 0.00128 58.85909 0.00179 50.06409 0.0185 148.82139 0.0222 138.58933 0.02498 133.36848 0.02867 109.46873 0.03885 88.98386

8.42154E-4 92.00566 6.79973E-4 45.4545 7.98816E-4 65.95798 0.00127 58.03363 0.00178 49.39267 0.0186 147.82439 0.02232 137.75583 0.02511 132.76904 0.02883 107.98899 0.03906 87.52827

8.34761E-4 89.8659 6.69723E-4 44.10826 7.83765E-4 64.73215 0.00126 57.11962 0.00177 48.64747 0.0187 146.70951 0.02244 136.81184 0.02525 132.07625 0.02899 106.3682 0.03927 85.94721

8.27125E-4 88.12157 6.59363E-4 42.73294 7.6857E-4 62.79265 0.00125 55.59512 0.00175 47.38616 0.0188 145.46243 0.02256 135.74441 0.02538 131.27824 0.02914 104.59238 0.03948 84.22955

8.19247E-4 86.42527 6.48978E-4 41.22193 7.53163E-4 61.69496 0.00124 54.75816 0.00173 46.69945 0.0189 144.06646 0.02268 134.53855 0.02551 130.36105 0.0293 102.64585 0.03969 82.36308

8.11142E-4 84.353 6.38683E-4 39.94342 7.37628E-4 60.21297 0.00123 53.59093 0.00172 45.73321 0.019 142.50198 0.0228 133.17676 0.02565 129.30815 0.02945 100.51101 0.0399 80.33438

8.02899E-4 82.86863 6.28407E-4 38.34538 7.21993E-4 59.3348 0.00122 53.10208 0.0017 45.37359 0.0191 140.74584 0.02292 131.63858 0.02578 128.09983 0.0296 98.16815 0.04011 78.12882

7.94538E-4 81.33624 6.18141E-4 37.06685 7.06223E-4 57.66062 0.0012 52.03174 0.00168 44.54272 0.0192 138.77055 0.02304 129.89999 0.02592 126.71244 0.02976 95.59518 0.04032 75.73053

7.86019E-4 79.91973 6.07922E-4 36.03047 6.90411E-4 56.70001 0.0012 51.16988 0.00167 43.80588 0.0193 136.54333 0.02316 127.9326 0.02605 125.11737 0.02992 92.76753 0.04053 73.12252

7.77383E-4 78.10766 5.97684E-4 34.5002 6.74563E-4 55.60233 0.00118 50.07524 0.00166 42.84859 0.0194 134.0248 0.02328 125.70282 0.02619 123.27979 0.03007 89.65803 0.04074 70.28686

7.68704E-4 76.45007 5.87401E-4 33.69618 6.58624E-4 54.59602 0.00117 48.93304 0.00164 41.82539 0.0195 131.16749 0.0234 123.17077 0.02633 121.15692 0.03022 86.23704 0.04095 67.20516

7.59891E-4 74.29119 5.77101E-4 32.81484 6.42575E-4 53.77265 0.00116 48.06787 0.00163 41.06105 0.0196 127.91389 0.02352 120.28906 0.02646 118.69581 0.03038 82.47288 0.04116 63.85937

7.50865E-4 73.01935 5.66818E-4 31.51695 6.26438E-4 52.4645 0.00115 46.95585 0.00161 40.12235 0.0197 124.19424 0.02364 117.00153 0.0266 115.83053 0.03054 78.33284 0.04137 60.23304

7.41661E-4 71.52555 5.56566E-4 30.40318 6.10275E-4 51.01905 0.00114 45.64378 0.00159 38.99763 0.0198 119.92396 0.02376 113.24211 0.02673 112.47847 0.03069 73.78523 0.04158 56.31344

7.32279E-4 69.93533 5.46337E-4 29.67669 5.94061E-4 49.98529 0.00112 44.5098 0.00157 37.98786 0.0199 115.00142 0.02388 108.93454 0.02687 108.536 0.03085 68.80304 0.04179 52.09464

7.22785E-4 68.7985 5.36086E-4 29.10527 5.77762E-4 48.7503 0.00111 43.64691 0.00155 37.29795

7.13232E-4 67.67109 5.25806E-4 28.69847 5.6142E-4 47.83554 0.0011 42.85373 0.00153 36.6252

7.03628E-4 66.09043 -- -- 5.44994E-4 47.11276 0.00108 42.23491 0.00152 36.10192

6.93821E-4 65.05932 -- -- 5.28517E-4 45.84128 0.00107 41.11258 0.0015 35.14598

6.83742E-4 62.95827 -- -- 5.12146E-4 44.97219 0.00106 40.39704 0.00148 34.54676

6.73479E-4 61.84018 -- -- 4.95896E-4 44.8898 0.00104 40.13446 0.00146 34.28547

6.63053E-4 60.38496 -- -- 4.79623E-4 44.21286 0.00103 39.68198 0.00144 33.92883

6.52491E-4 58.8718 -- -- 4.63289E-4 43.09218 0.00102 38.98542 0.00143 33.39355

6.41867E-4 57.32003 -- -- 4.46942E-4 42.09046 0.00101 38.21156 0.00141 32.75631

6.31189E-4 55.8553 -- -- 4.30552E-4 41.01103 9.95276E-4 37.48726 0.00139 32.18469

6.20331E-4 54.01435 -- -- 4.1413E-4 39.58396 9.82065E-4 36.55671 0.00137 31.45735

6.09288E-4 52.3757 -- -- 3.97732E-4 38.69656 9.68866E-4 35.7361 0.00136 30.75101

5.98035E-4 50.90586 -- -- 3.81399E-4 37.70857 9.557E-4 34.75625 0.00134 29.89507

5.86584E-4 49.20449 -- -- 3.65127E-4 36.42774 9.42563E-4 33.73341 0.00132 29.04522

5.74989E-4 47.57566 -- -- 3.48927E-4 35.2294 9.29464E-4 32.6145

5.63284E-4 45.97575 -- -- 3.32852E-4 34.2872 9.16426E-4 31.76234

5.51515E-4 44.32764 -- -- 3.16903E-4 33.58271 9.03451E-4 31.06856

5.39644E-4 42.51531 -- -- 3.01053E-4 32.60392 8.90527E-4 30.34381

5.27628E-4 41.27208 -- -- 2.85286E-4 32.06419 8.82643E-4 29.70919

5.15539E-4 39.66228

5.03514E-4 38.58266

4.91616E-4 37.80205

4.79827E-4 37.28159

4.68076E-4 36.38521

4.56342E-4 35.56592

4.44576E-4 34.45754

**(c) *β*=60°**

5.75146E-8 0.19668 2.42273E-7 0.52886 1.46552E-7 0.11791 1.51348E-7 0.03754 3.94569E-8 0.10863 1.15E-4 2.08612 1.32E-4 2.019 1.38E-4 2.0095 1.55E-4 1.96136 1.9E-4 1.62127

1.44608E-7 0.54463 5.66951E-7 1.373 3.83833E-7 0.30133 4.56818E-7 -0.10321 8.91442E-8 0.29628 2.3E-4 4.14353 2.64E-4 4.00931 2.76E-4 3.97164 3.1E-4 3.88193 3.8E-4 3.22457

2.82638E-7 0.52951 1.1125E-6 1.64758 7.50226E-7 0.32753 8.44208E-7 -0.08444 2.48437E-7 0.71106 3.45E-4 6.1725 3.96E-4 5.97121 4.14E-4 5.88718 4.65E-4 5.76225 5.7E-4 4.81002

4.86398E-7 1.25569 1.91189E-6 2.55263 1.23526E-6 0.69435 1.33018E-6 -0.07506 4.57417E-7 1.13573 4.6E-4 8.1733 5.28E-4 7.90495 5.52E-4 7.75693 6.2E-4 7.60291 7.6E-4 6.37771

7.19738E-7 1.98189 2.97004E-6 2.66453 1.83021E-6 0.98256 1.94668E-6 -0.4504 7.07316E-7 1.12585 5.75E-4 10.14621 6.6E-4 9.81083 6.9E-4 9.58173 7.75E-4 9.4045 9.5E-4 7.92776

9.39928E-7 2.28461 4.38088E-6 3.53914 2.5473E-6 1.75551 2.68675E-6 -0.58176 1.12528E-6 1.82705 6.9E-4 12.09153 7.92E-4 11.68914 8.28E-4 11.36244 9.3E-4 11.16766 0.00114 9.46027

1.23078E-6 2.07257 6.16094E-6 3.80364 3.40221E-6 1.40177 3.5976E-6 -0.08445 1.66599E-6 2.3801 8.05E-4 14.00955 9.24E-4 13.54017 9.66E-4 13.09995 0.00109 12.89301 0.00133 10.97534

1.63994E-6 2.63234 8.20309E-6 4.88155 4.39845E-6 1.55898 4.61537E-6 -0.06569 2.20963E-6 2.93315 9.2E-4 15.90058 0.00106 15.36422 0.0011 14.79517 0.00124 14.58121 0.00152 12.4731

2.12795E-6 3.34337 1.05452E-5 5.5223 5.55173E-6 2.3581 5.71088E-6 0.33784 2.81319E-6 3.22944 0.00103 17.76492 0.00119 17.16161 0.00124 16.44905 0.0014 16.23293 0.00171 13.95366

2.72605E-6 3.84271 1.31839E-5 6.93596 6.87598E-6 3.10481 6.87443E-6 0.55366 3.52343E-6 3.83186 0.00115 19.60288 0.00132 18.93265 0.00138 18.06255 0.00155 17.84885 0.0019 15.41712

3.36196E-6 4.78083 1.60453E-5 8.16647 8.34154E-6 3.22285 8.04492E-6 0.60995 4.3155E-6 4.48367 0.00127 21.41479 0.00145 20.67766 0.00152 19.63663 0.0017 19.42968 0.00209 16.86361

3.97815E-6 5.21956 1.91752E-5 9.59024 9.92925E-6 3.27522 9.24181E-6 0.54424 5.16602E-6 4.83926 0.00138 23.20098 0.00158 22.39696 0.00166 21.17228 0.00186 20.97611 0.00228 18.29325

4.59105E-6 5.38595 2.26183E-5 11.36998 1.16007E-5 4.71625 1.04914E-5 1.0697 6.05017E-6 5.74784 0.0015 24.96176 0.00172 24.09089 0.00179 22.67051 0.00201 22.48887 0.00247 19.70615

5.32387E-6 6.36931 2.64454E-5 13.27167 1.33664E-5 6.00008 1.17466E-5 1.5389 7.05708E-6 6.765 0.00161 26.69749 0.00185 25.75978 0.00193 24.13233 0.00217 23.96867 0.00266 21.10243

6.29991E-6 6.76268 3.06154E-5 15.02097 1.53607E-5 6.28832 1.29713E-5 2.34581 8.1356E-6 7.6637 0.00173 28.40849 0.00198 27.40397 0.00207 25.55874 0.00232 25.41626 0.00285 22.48223

7.40742E-6 6.97452 3.51447E-5 17.15669 1.75328E-5 7.7163 1.41765E-5 3.30288 9.32081E-6 8.41427 0.00184 30.09511 0.00211 29.02381 0.00221 26.95078 0.00248 26.83236 0.00304 23.84565

8.62504E-6 7.4586 4.00893E-5 19.08892 1.98795E-5 8.98708 1.54137E-5 4.09106 1.06828E-5 9.29322 0.00196 31.75769 0.00224 30.61963 0.00235 28.30946 0.00264 28.21773 0.00323 25.19284

1.01006E-5 8.26063 4.55169E-5 21.26527 2.24809E-5 10.38887 1.67258E-5 5.18893 1.21486E-5 9.84634 0.00207 33.39659 0.00238 32.19179 0.00248 29.63582 0.00279 29.57311 0.00342 26.5239

1.17535E-5 8.9564 5.14405E-5 22.92284 2.5337E-5 11.55492 1.81476E-5 6.11793 1.36962E-5 11.00184 0.00218 35.01215 0.00251 33.74065 0.00262 30.93088 0.00295 30.89925 0.00361 27.83897

1.35544E-5 10.01557 5.77944E-5 25.13996 2.85124E-5 13.2448 1.96514E-5 6.92486 1.54177E-5 11.78195 0.0023 36.60474 0.00264 35.26655 0.00276 32.19566 0.0031 32.19691 0.0038 29.13819

1.56379E-5 11.07448 6.45785E-5 27.32643 3.20141E-5 14.68596 2.12912E-5 8.5857 1.73322E-5 12.88813 0.00242 38.1747 0.00277 36.76986 0.0029 33.43117 0.00326 33.46682 0.00399 30.42167

1.79202E-5 12.07311 7.17548E-5 29.40117 3.59135E-5 16.0353 2.31809E-5 9.21436 1.94132E-5 13.87577 0.00253 39.7224 0.0029 38.25093 0.00304 34.63844 0.00341 34.70975 0.00418 31.68955

2.05066E-5 13.08679 7.93069E-5 31.67926 4.02736E-5 17.3061 2.53817E-5 10.16214 2.16653E-5 14.94227 0.00265 41.2482 0.00304 39.71013 0.00317 35.81846 0.00357 35.92643 0.00437 32.94196

2.33837E-5 13.69186 8.72232E-5 33.85561 4.51118E-5 18.94374 2.79143E-5 11.09099 2.40181E-5 16.03852 0.00276 42.75246 0.00317 41.14781 0.00331 36.97222 0.00372 37.11763 0.00456 34.17903

2.65418E-5 14.4938 9.56476E-5 36.38794 5.04839E-5 20.42401 3.07552E-5 11.89799 2.64777E-5 17.65821 0.00288 44.23554 0.0033 42.56435 0.00345 38.10072 0.00388 38.28406 0.00475 35.4009

3.0063E-5 15.88568 1.0472E-4 39.24746 5.63444E-5 21.53756 3.39724E-5 12.89263 2.92251E-5 18.71489 0.00299 45.69781 0.00343 43.96011 0.00359 39.20491 0.00403 39.42648 0.00494 36.60771

3.39358E-5 16.47572 1.14251E-4 42.17454 6.26987E-5 23.64679 3.76769E-5 13.9717 3.23306E-5 19.86054 0.00311 47.13964 0.00356 45.33545 0.00373 40.28576 0.00419 40.54562 0.00513 37.79957

3.82426E-5 17.82218 1.24067E-4 44.54404 6.95345E-5 25.40218 4.19215E-5 14.81611 3.57693E-5 20.99617 0.00322 48.56138 0.0037 46.69073 0.00386 41.3442 0.00434 41.6422 0.00532 38.97664

4.28975E-5 18.8965 1.34214E-4 47.25952 7.67996E-5 27.55089 4.66869E-5 15.76391 3.96288E-5 21.85547 0.00333 49.96341 0.00383 48.02633 0.004 42.38117 0.00449 42.71693 0.00551 40.13905

4.79354E-5 20.06136 1.44699E-4 49.34425 8.45026E-5 29.83023 5.19909E-5 16.72094 4.39385E-5 22.61586 0.00345 51.34608 0.00396 49.34261 0.00414 43.39756 0.00465 43.77052 0.0057 41.28694

5.34102E-5 21.60461 1.55473E-4 51.67322 9.26397E-5 32.09669 5.7842E-5 17.80955 4.87216E-5 23.3961 0.00357 52.70977 0.00409 50.63993 0.00428 44.39429 0.00481 44.80369 0.00589 42.42043

5.92418E-5 22.78468 1.6656E-4 54.04272 1.01238E-4 33.77368 6.41888E-5 18.97306 5.40309E-5 24.33445 0.00368 54.05483 0.00422 51.91865 0.00442 45.37221 0.00496 45.81712 0.00608 43.53968

6.54709E-5 24.22185 1.78086E-4 56.04614 1.10295E-4 36.2104 7.09967E-5 20.40856 5.98122E-5 25.09485 0.0038 55.38163 0.00436 53.17915 0.00455 46.33219 0.00511 46.81148 0.00627 44.64482

7.20943E-5 25.78014 1.90109E-4 57.49036 1.19835E-4 37.69081 7.8271E-5 21.54399 6.61035E-5 25.82558 0.00391 56.69053 0.00449 54.42178 0.00469 47.27505 0.00527 47.78746 0.00646 45.73599

7.90743E-5 27.76206 2.02551E-4 59.47346 1.29876E-4 39.42002 8.60132E-5 22.58546 7.29823E-5 26.76383 0.00402 57.98189 0.00462 55.6469 0.00483 48.20161 0.00543 48.74572 0.00665 46.81333

8.64704E-5 29.27507 2.15436E-4 60.88712 1.40355E-4 41.39847 9.42748E-5 23.8242 8.03814E-5 27.36621 0.00414 59.25606 0.00475 56.85488 0.00497 49.11268 0.00558 49.6869 0.00684 47.87698

9.42336E-5 31.43859 2.28857E-4 62.65652 1.51253E-4 43.28492 1.03054E-4 25.11899 8.82569E-5 28.30446 0.00426 60.51341 0.00488 58.04606 0.00511 50.00901 0.00573 50.61165 0.00703 48.92707

1.0229E-4 33.11798 2.42748E-4 63.91769 1.62561E-4 44.35899 1.12297E-4 26.3857 9.66073E-5 29.04522 0.00437 61.75429 0.00502 59.2208 0.00524 50.89137 0.00589 51.52059 0.00722 49.96376

1.10688E-4 35.76561 2.5708E-4 65.667 1.74343E-4 45.65611 1.21968E-4 27.69002 1.05446E-4 29.99317 0.00449 62.97904 0.00515 60.37947 0.00538 51.76048 0.00605 52.41433 0.00741 50.98718

1.19502E-4 38.05008 2.71878E-4 67.29402 1.86566E-4 46.71716 1.32149E-4 29.27574 1.14794E-4 30.84255 0.0046 64.18803 0.00528 61.52239 0.00552 52.61706 0.0062 53.29347 0.0076 51.99747

1.28783E-4 41.59487 2.87069E-4 68.83996 1.99112E-4 48.57135 1.42895E-4 31.09724 1.24609E-4 31.8399 0.00471 65.38159 0.00541 62.64993 0.00566 53.46178 0.00636 54.15862 0.00779 52.99477

1.38463E-4 44.94908 3.02652E-4 70.56883 2.11998E-4 49.88772 1.54086E-4 33.35758 1.34919E-4 32.68938 0.00483 66.56008 0.00554 63.76243 0.0058 54.29533 0.00651 55.01033 0.00798 53.97923

1.4841E-4 46.87037 3.18671E-4 72.10444 2.25029E-4 51.02746 1.65667E-4 34.61486 1.45855E-4 34.29446 0.00494 67.72382 0.00568 64.86023 0.00593 55.11833 0.00666 55.84917 0.00817 54.95098

1.58593E-4 49.07932 3.35096E-4 73.49778 2.38064E-4 52.27193 1.77617E-4 35.95669 1.57301E-4 36.07674 0.00506 68.87316 0.00581 65.94366 0.00607 55.93142 0.00682 56.67569 0.00836 55.91016

1.68934E-4 51.48491 3.51859E-4 75.62316 2.51212E-4 53.77866 1.89881E-4 37.28919 1.69088E-4 37.47921 0.00517 70.00844 0.00594 67.01307 0.00621 56.7352 0.00698 57.49043 0.00855 56.85693

1.79405E-4 53.61802 3.68857E-4 77.57583 2.64571E-4 54.9576 2.02421E-4 38.66842 1.81247E-4 38.88156 0.00529 71.12998 0.00607 68.06878 0.00635 57.53023 0.00713 58.2939 0.00874 57.79141

1.90039E-4 56.11448 3.86095E-4 79.9048 2.78212E-4 55.96643 2.15264E-4 40.029 1.93872E-4 40.0667 0.00541 72.23812 0.0062 69.11113 0.00649 58.3171 0.00728 59.08662 0.00893 58.71375

2.00846E-4 57.86954 4.03666E-4 81.74551 2.92023E-4 57.1717 2.28404E-4 41.81188 2.06987E-4 41.26165 0.00552 73.33317 0.00634 70.14044 0.00662 59.09632 0.00744 59.86907 0.00912 59.6241

2.11922E-4 59.91178 4.21501E-4 83.56579 3.05989E-4 57.64328 2.41775E-4 43.34124 2.20579E-4 42.80238 0.00564 74.41545 0.00647 71.15702 0.00676 59.86842 0.0076 60.64175 0.00931 60.52258

2.23274E-4 62.13594 4.3952E-4 85.7728 3.20181E-4 58.62592 2.55414E-4 44.89888 2.3471E-4 43.70105 0.00575 75.48529 0.0066 72.1612 0.0069 60.63389 0.00775 61.4051 0.0095 61.40934

2.34828E-4 64.22362 4.57724E-4 88.15253 3.34476E-4 59.69999 2.69381E-4 45.97806 2.49385E-4 44.75783 0.00587 76.54299 0.00673 73.15329 0.00704 61.39321 0.00791 62.15959 0.00969 62.28453

2.46624E-4 66.00892 4.7611E-4 90.19671 3.48983E-4 60.42066 2.83619E-4 47.69505 2.64587E-4 46.14045 0.00598 77.58887 0.00686 74.13359 0.00718 62.14684 0.00806 62.90565 0.00988 63.14827

2.58606E-4 68.02109 4.94748E-4 92.24079 3.63698E-4 61.24606 2.98162E-4 49.40292 2.80316E-4 47.16746 0.00609 78.62321 0.007 75.10242 0.00731 62.89521 0.00822 63.64371 0.01007 64.00071

2.70765E-4 69.68544 5.13618E-4 94.27476 3.78513E-4 62.34646 3.13076E-4 50.96056 2.96583E-4 48.59948 0.00621 79.64634 0.00713 76.06006 0.00745 63.63874 0.00837 64.37418 0.01026 64.84199

2.83069E-4 71.62177 5.3269E-4 96.04429 3.93483E-4 63.32896 3.28257E-4 52.39616 3.13354E-4 50.18951 0.00632 80.65853 0.00726 77.00683 0.00759 64.37783 0.00852 65.09745 0.01045 65.67225

2.95487E-4 72.52955 5.51926E-4 98.09869 4.08501E-4 65.21555 3.43714E-4 54.21644 3.30622E-4 51.65119 0.00644 81.66008 0.00739 77.943 0.00773 65.11286 0.00868 65.81392 0.01064 66.49162

3.08044E-4 74.52667 5.7119E-4 99.93941 4.23556E-4 66.21122 3.59569E-4 55.69907 3.48379E-4 53.21156 0.00656 82.65127 0.00752 78.86886 0.00787 65.84419 0.00884 66.52394 0.01083 67.30025

3.207E-4 75.85826 5.90454E-4 102.41065 4.38746E-4 68.13702 3.75775E-4 57.41627 3.666E-4 54.93986 0.00667 83.63239 0.00766 79.7847 0.008 66.57217 0.00899 67.22788 0.01102 68.09826

3.33546E-4 76.97769 6.09586E-4 104.68875 4.54042E-4 69.3161 3.92225E-4 59.30225 3.85253E-4 56.42138 0.00678 84.60371 0.00779 80.6908 0.00814 67.29713 0.00914 67.92609 0.01121 68.8858

3.46498E-4 78.52093 6.28613E-4 106.78369 4.69415E-4 71.1107 4.0903E-4 61.59177 4.04257E-4 58.33725 0.0069 85.56551 0.00792 81.58741 0.00828 68.01938 0.0093 68.61889 0.0114 69.663

3.59534E-4 79.08073 6.47542E-4 108.81766 4.84901E-4 72.2244 4.26202E-4 63.27136 4.23634E-4 60.21363 0.00701 86.51804 0.00805 82.47483 0.00842 68.73922 0.00945 69.30661 0.01159 70.43

3.72733E-4 80.36671 6.66262E-4 110.79066 5.00484E-4 73.97994 4.43673E-4 64.89469 4.43409E-4 61.44806 0.00713 87.46157 0.00818 83.35329 0.00856 69.45693 0.00961 69.98954 0.01178 71.18692

3.85955E-4 82.34848 6.8477E-4 112.68224 5.16139E-4 75.56508 4.61518E-4 66.87453 4.63492E-4 63.3444 0.00724 88.39636 0.00832 84.22307 0.00869 70.17276 0.00976 70.668 0.01197 71.93391

3.9922E-4 83.33209 7.02962E-4 114.60437 5.31923E-4 76.83587 4.79695E-4 68.71368 4.83877E-4 65.29974 0.00736 89.32266 0.00845 85.08442 0.00883 70.88698 0.00992 71.34225 0.01216 72.67109

4.12518E-4 85.0718 7.20799E-4 116.24172 5.47726E-4 78.68312 4.98153E-4 70.48703 5.04603E-4 66.51453 0.00747 90.24072 0.00858 85.93758 0.00897 71.59982 0.01008 72.01257 0.01235 73.3986

4.25807E-4 86.29731 7.38254E-4 117.75701 5.63519E-4 79.9276 5.16896E-4 72.56085 5.256E-4 67.83773 0.00759 91.15077 0.00871 86.78279 0.00911 72.3115 0.01023 72.67922 0.01254 74.11658

4.39191E-4 88.17332 7.55222E-4 119.28251 5.793E-4 81.97129 5.35828E-4 74.48443 5.46884E-4 69.57606 0.0077 92.05305 0.00884 87.6203 0.00925 73.02223 0.01039 73.34245 0.01273 74.82514

4.52618E-4 90.27636 7.71656E-4 120.77748 5.94878E-4 83.53024 5.54904E-4 76.59565 5.68501E-4 71.018 0.00782 92.9478 0.00898 88.45033 0.00938 73.73222 0.01054 74.00249 0.01292 75.52442

4.66066E-4 91.72873 7.87579E-4 122.0181 6.10299E-4 84.78802 5.7417E-4 78.68812 5.90444E-4 72.63758 0.00793 93.83524 0.00911 89.27312 0.00952 74.44163 0.0107 74.65958 0.01311 76.21455

4.79543E-4 94.05847 8.03025E-4 122.94362 6.25639E-4 86.21591 5.93606E-4 80.76173 6.12685E-4 74.30666 0.00805 94.71559 0.00924 90.08889 0.00966 75.15064 0.01085 75.31391 0.0133 76.89565

4.92987E-4 95.78347 8.18052E-4 124.3064 6.4083E-4 87.8929 6.132E-4 82.86372 6.35182E-4 76.15339 0.00816 95.58907 0.00937 90.89785 0.0098 75.85942 0.011 75.96571 0.01349 77.56785

5.06414E-4 97.26607 8.32702E-4 125.65898 6.55817E-4 89.6091 6.32869E-4 84.75902 6.57871E-4 77.85214 0.00828 96.45589 0.0095 91.70022 0.00994 76.56812 0.01116 76.61517 0.01368 78.23128

5.19864E-4 98.93043 8.47004E-4 126.87939 6.70546E-4 91.15503 6.52541E-4 86.92661 6.80704E-4 79.66921 0.00839 97.31625 0.00964 92.4962 0.01007 77.27686 0.01132 77.26246 0.01387 78.88607

5.33171E-4 100.97283 8.6105E-4 128.41512 6.85012E-4 91.96727 6.72244E-4 88.7845 7.03604E-4 81.48638 0.00851 98.17036 0.00977 93.286 0.01021 77.98578 0.01147 77.90777 0.01406 79.53233

5.46223E-4 102.37977 8.74841E-4 129.83888 6.99179E-4 93.13319 6.91916E-4 90.73627 7.26544E-4 83.3234 0.00862 99.01841 0.0099 94.06981 0.01035 78.695 0.01163 78.55126 0.01425 80.17018

5.59089E-4 104.60359 8.88377E-4 131.51677 7.13043E-4 94.23359 7.11514E-4 92.29391 7.49484E-4 84.97265 0.00874 99.86059 0.01003 94.84782 0.01049 79.40462 0.01178 79.19308 0.01444 80.79976

5.71789E-4 106.99413 9.01636E-4 132.88978 7.26613E-4 95.15056 7.31E-4 94.23615 7.72353E-4 86.10838 0.00885 100.69709 0.01016 95.62023 0.01063 80.11474 0.01193 79.83339 0.01463 81.42118

5.84302E-4 108.91558 9.14597E-4 134.1916 7.39826E-4 96.35597 7.50373E-4 95.47468 7.95167E-4 87.97485 0.00897 101.52809 0.0103 96.38721 0.01076 80.82545 0.01209 80.47232 0.01482 82.03455

5.96603E-4 110.42842 9.27305E-4 135.00505 7.52608E-4 98.2294 7.69633E-4 97.12629 8.17859E-4 88.99215 0.00908 102.35376 0.01043 97.14894 0.0109 81.53684 0.01225 81.11 0.01501 82.64

6.08601E-4 111.72961 9.39826E-4 136.44925 7.65102E-4 99.48704 7.88694E-4 98.74951 8.4039E-4 89.82168 0.0092 103.17427 0.01056 97.9056 0.01104 82.24897 0.0124 81.74655 0.0152 83.23764

6.20245E-4 113.30309 9.52183E-4 137.395 7.77399E-4 100.49586 8.07597E-4 99.87552 8.62803E-4 91.15501 0.00931 103.98978 0.01069 98.65734 0.01118 82.96191 0.01256 82.38209 0.01539 83.82759

6.3161E-4 114.84633 9.64354E-4 138.68659 7.89443E-4 103.0635 8.26312E-4 100.94527 8.85129E-4 92.21169 0.00943 104.80047 0.01082 99.40434 0.01132 83.67572 0.01271 83.01671 0.01558 84.40996

6.42721E-4 115.9205 9.76417E-4 139.69342 8.01335E-4 105.09417 8.44825E-4 102.47473 9.0735E-4 93.30805 0.00954 105.60647 0.01096 100.14674 0.01145 84.39045 0.01287 83.65053 0.01577 84.98487

6.53508E-4 116.67691 9.88262E-4 140.87317 8.13037E-4 106.82353 8.63221E-4 103.92918 9.29472E-4 94.32515 0.00966 106.40795 0.01109 100.8847 0.01159 85.10613 0.01302 84.28361 0.01596 85.55242

6.63954E-4 118.74956 9.99917E-4 141.87999 8.24556E-4 108.90642 8.81436E-4 104.72665 9.5158E-4 94.83865 0.00977 107.20504 0.01122 101.61837 0.01173 85.82281 0.01317 84.91606 0.01615 86.11273

6.74083E-4 120.73165 0.00101 142.47998 8.35975E-4 111.50039 8.99514E-4 106.02155 9.73741E-4 95.68803 0.00989 107.99788 0.01135 102.34788 0.01187 86.54052 0.01333 85.54794 0.01634 86.66591

6.8395E-4 121.80581 0.00102 143.62919 8.47283E-4 112.745 9.17535E-4 107.6542 9.95848E-4 97.20892 0.01001 108.78662 0.01148 103.07338 0.01201 87.25927 0.01349 86.17931 0.01653 87.21205

6.93639E-4 122.97068 0.00103 144.63601 8.5846E-4 114.95895 9.35386E-4 108.78964 0.00102 97.89036 0.01012 109.57137 0.01162 103.79499 0.01214 87.97909 0.01364 86.81025 0.01672 87.75128

7.03221E-4 125.53513 0.00105 145.44958 8.69514E-4 117.18621 9.53079E-4 109.97191 0.00104 99.01617 0.01023 110.35227 0.01175 104.51285 0.01228 88.69998 0.0138 87.4408 0.01691 88.28368

7.12651E-4 127.94839 0.00106 146.85302 8.80457E-4 118.41766 9.70606E-4 111.31384 0.00106 100.15201 0.01035 111.12943 0.01188 105.22707 0.01242 89.42195 0.01395 88.07101 0.0171 88.80938

7.22048E-4 129.83944 0.00107 146.92411 8.91206E-4 120.35648 9.87983E-4 113.01218 0.00108 100.4483 0.01047 111.90296 0.01201 105.93777 0.01256 90.14501 0.0141 88.70092 0.01729 89.32845

7.3146E-4 131.15566 0.00108 147.88017 9E-4 121.99413 0.00101 114.10991 0.00111 101.48523 0.01058 112.67299 0.01214 106.64507 0.0127 90.86914 0.01426 89.33058 0.01748 89.84102

7.40755E-4 133.57644 0.00109 148.40907 9.09E-4 123.95927 0.00102 114.89805 0.00113 102.84812 0.0107 113.4396 0.01228 107.34907 0.01283 91.59433 0.01442 89.95999 0.01767 90.34717

7.49983E-4 135.07408 0.0011 148.99895 9.27E-4 124.43085 0.00104 116.09908 0.00115 103.83566 0.01081 114.20291 0.01241 108.04988 0.01297 92.32058 0.01457 90.58919 0.01786 90.847

7.59199E-4 136.85922 0.00111 149.59882 9.36E-4 124.98112 0.00106 117.57238 0.00117 105.00116 0.01093 114.96301 0.01254 108.74758 0.01311 93.04786 0.01473 91.2182 0.01805 91.3406

7.68446E-4 138.50855 0.00112 149.51752 9.45E-4 126.25191 0.00107 118.61385 0.00119 106.02817 0.01104 115.71998 0.01267 109.44229 0.01325 93.77614 0.01488 91.84702 0.01824 91.82808

7.77657E-4 139.62814 0.00113 149.94458 9.54E-4 127.45718 0.00109 119.99328 0.00121 106.65029 0.01116 116.47391 0.0128 110.13407 0.01339 94.50541 0.01504 92.47566 0.01843 92.30952

7.86709E-4 140.12729 0.00114 150.31087 9.63E-4 128.15153 0.0011 120.55619 0.00123 107.25267 0.01127 117.22489 0.01294 110.82303 0.01352 95.23562 0.01519 93.10412 0.01862 92.785

7.95641E-4 140.86852 0.00115 150.62608 9.72E-4 129.27811 0.00112 121.26932 0.00126 108.15145 0.01138 117.97298 0.01307 111.50923 0.01366 95.96674 0.01534 93.73239 0.01881 93.25463

8.08E-4 141.98811 0.00116 150.4125 9.9E-4 129.95943 0.00114 122.29214 0.00128 108.77357 0.0115 118.71826 0.0132 112.19274 0.0138 96.69873 0.0155 94.36046 0.019 93.71848

8.16E-4 143.27425 0.00117 150.3413 9.99E-4 130.69298 0.00115 123.18358 0.0013 109.20812 0.01162 119.4608 0.01333 112.87365 0.01394 97.43155 0.01565 94.98832 0.01919 94.17665

8.24E-4 144.28778 0.00117 150.73793 0.00101 131.67563 0.00117 123.69955 0.00132 110.11671 0.01173 120.20064 0.01346 113.55201 0.01408 98.16514 0.01581 95.61593 0.01938 94.6292

8.32E-4 145.72511 0.00118 150.62608 0.00103 132.57958 0.00118 124.80691 0.00134 110.44276 0.01185 120.93786 0.0136 114.22788 0.01421 98.89945 0.01597 96.24329 0.01957 95.07623

8.4E-4 146.49673 0.0012 150.24978 0.00103 133.05116 0.0012 125.49186 0.00136 111.06488 0.01196 121.67249 0.01373 114.90131 0.01435 99.63444 0.01612 96.87034 0.01976 95.51781

8.48E-4 147.34403 0.0012 150.19892 0.00104 133.39168 0.00121 126.39273 0.00138 111.32169 0.01208 122.40459 0.01386 115.57237 0.01449 100.37003 0.01628 97.49706 0.01995 95.95402

8.56E-4 147.61649 0.00121 149.72099 0.00105 133.98129 0.00123 126.89937 0.0014 111.5291 0.01219 123.1342 0.01399 116.24108 0.01463 101.10617 0.01643 98.12341 0.02014 96.38494

8.64E-4 148.53914 0.00122 149.58871 0.00107 134.25642 0.00124 127.20895 0.00141 111.84512 0.01231 123.86134 0.01412 116.90749 0.01477 101.84279 0.01658 98.74932 0.02033 96.81063

8.72E-4 148.91735 0.00122 149.39556 0.00108 134.74116 0.00126 127.52806 0.00143 112.49679 0.01242 124.58606 0.01426 117.57164 0.0149 102.57982 0.01674 99.37476 0.02052 97.23117

8.8E-4 149.96127 0.00123 149.03959 0.00109 135.68446 0.00127 127.8564 0.00145 112.67465 0.01253 125.30837 0.01439 118.23356 0.01504 103.31718 0.0169 99.99966 0.02071 97.64663

8.88E-4 151.45924 0.00123 149.29372 0.0011 136.11669 0.00128 128.1004 0.00147 113.09928 0.01265 126.02829 0.01452 118.89326 0.01518 104.05481 0.01705 100.62396 0.0209 98.05707

8.96E-4 152.20062 0.00124 148.54124 0.00112 136.6538 0.0013 127.79071 0.00149 112.75371 0.01277 126.74585 0.01465 119.55078 0.01532 104.79261 0.01721 101.2476 0.02109 98.46255

9.04E-4 152.69978 0.00124 148.27679 0.00112 137.43986 0.00131 128.34439 0.0015 113.35619 0.01288 127.46105 0.01478 120.20612 0.01546 105.53051 0.01736 101.87049 0.02128 98.86314

9.12E-4 153.98577 0.00125 147.61583 0.00113 137.74118 0.00132 128.31611 0.00152 113.50418 0.013 128.17389 0.01492 120.85929 0.01559 106.26842 0.01751 102.49256 0.02147 99.2589

9.2E-4 154.5454 0.00125 147.17845 0.00114 138.5404 0.00134 128.12847 0.00154 113.34627 0.01311 128.88437 0.01505 121.51031 0.01573 107.00625 0.01767 103.11373 0.02166 99.64989

9.28E-4 154.3187 0.00125 146.64963 0.00115 139.15619 0.00135 128.65397 0.00155 113.52402 0.01323 129.59249 0.01518 122.15916 0.01587 107.74389 0.01783 103.73391 0.02185 100.03615

9.36E-4 155.1356 0.00127 145.8158 0.00116 139.65381 0.00136 128.20359 0.00157 113.73143 0.01334 130.29823 0.01531 122.80584 0.01601 108.48126 0.01798 104.353 0.02204 100.41775

9.44E-4 156.3459 0.00127 144.85974 0.00117 140.70198 0.00137 128.37257 0.00159 113.57342 0.01346 131.00157 0.01544 123.45034 0.01615 109.21826 0.01813 104.9709 0.02223 100.79472

9.52E-4 156.8905 0.00127 143.87319 0.00118 140.81988 0.00138 128.25985 0.0016 113.47452 0.01357 131.70249 0.01558 124.09264 0.01628 109.95477 0.01829 105.58751 0.02242 101.16712

9.6E-4 156.82998 0.00128 143.18171 0.00119 141.40949 0.00139 127.98777 0.00162 113.60297 0.01368 132.40095 0.01571 124.73272 0.01642 110.69068 0.01845 106.20271 0.02261 101.53499

9.68E-4 157.79829 0.00128 142.14436 0.0012 142.22173 0.0014 128.3819 0.00163 113.52402 0.0138 133.09693 0.01584 125.37055 0.01656 111.42589 0.0186 106.81639 0.0228 101.89837

9.76E-4 158.28244 0.00128 141.10702 0.00121 142.37897 0.00141 128.05346 0.00165 113.3068 0.01392 133.79037 0.01597 126.00609 0.0167 112.16027 0.01876 107.42842 0.02299 102.2573

9.84E-4 158.41866 0.00128 140.12059 0.00121 141.97278 0.00142 127.95959 0.00166 113.72151 0.01403 134.48123 0.0161 126.6393 0.01684 112.8937 0.01891 108.03868 0.02318 102.61181

9.92E-4 159.23564 0.00128 138.69676 0.00122 142.07765 0.00143 127.93151 0.00167 113.37583 0.01415 135.16945 0.01624 127.27013 0.01697 113.62606 0.01906 108.64702 0.02337 102.96193

1E-3 160.03739 0.00128 137.53736 0.00123 141.92027 0.00144 127.34976 0.00169 113.54386 0.01426 135.85496 0.01637 127.89853 0.01711 114.35721 0.01922 109.25331 0.02356 103.3077

0.00101 159.73491 0.00128 136.30681 0.00124 141.38331 0.00145 126.88052 0.0017 113.21781 0.01438 136.5377 0.0165 128.52443 0.01725 115.08701 0.01938 109.85738 0.02375 103.64913

0.00101 159.41718 0.00128 135.3509 0.00125 140.54474 0.00146 126.45821 0.00171 112.98085 0.01449 137.21758 0.01663 129.14777 0.01739 115.81532 0.01953 110.45909 0.02394 103.98625

0.00102 159.31115 0.00128 133.88645 0.00126 139.95513 0.00146 126.20499 0.00172 112.45742 0.01461 137.89452 0.01676 129.76848 0.01753 116.54199 0.01969 111.05827 0.02413 104.31907

0.00102 160.00706 0.00128 132.75758 0.00127 139.69316 0.00147 125.77336 0.00174 112.48698 0.01472 138.56843 0.0169 130.38645 0.01766 117.26687 0.01984 111.65474 0.02432 104.64761

0.00103 159.6441 0.00128 131.87278 0.00128 138.98579 0.00148 125.54815 0.00175 111.81546 0.01483 139.23919 0.01703 131.00162 0.0178 117.98981 0.01999 112.24832 0.02451 104.97187

0.00103 159.70454 0.00128 130.45912 0.00128 137.57091 0.00149 124.68487 0.00176 111.37098 0.01495 139.9067 0.01716 131.61386 0.01794 118.71062 0.02015 112.83882 0.0247 105.29187

0.00104 159.82556 0.00128 129.12691 0.00129 137.78066 0.00149 124.21577 0.00177 111.06488 0.01507 140.57084 0.01729 132.22308 0.01808 119.42914 0.02031 113.42604 0.02489 105.6076

0.00105 159.79531 0.00128 128.00827 0.0013 137.00763 0.0015 123.4369 0.00178 109.98836 0.01518 141.23148 0.01742 132.82916 0.01822 120.14519 0.02046 114.00977 0.02508 105.91905

0.00105 160.06767 0.00127 126.01488 0.0013 136.4575 0.00151 122.66742 0.00179 109.67245 0.0153 141.88846 0.01756 133.43197 0.01835 120.85857 0.02062 114.58979 0.02527 106.22622

0.00106 160.11301 0.00127 124.15384 0.00131 135.90709 0.00151 122.28273 0.0018 109.18849 0.01541 142.54166 0.01769 134.03137 0.01849 121.5691 0.02077 115.16586 0.02546 106.5291

0.00106 159.52299 0.00127 122.71984 0.00131 135.76315 0.00152 121.92616 0.0018 108.70454 0.01553 143.19089 0.01782 134.62722 0.01863 122.27657 0.02092 115.73774 0.02565 106.82766

0.00106 158.96318 0.00127 120.82838 0.00131 135.31762 0.00152 121.24123 0.00181 108.47739 0.01564 143.83598 0.01795 135.21936 0.01877 122.98075 0.02108 116.30519 0.02584 107.12187

0.00106 159.14486 0.00125 119.12999 0.00131 135.25216 0.00153 120.95035 0.00182 107.97366 0.01576 144.47675 0.01808 135.80761 0.01891 123.68143 0.02124 116.86792 0.02603 107.41171

0.00106 158.60009 0.00125 117.55366 0.00132 135.04248 0.00153 120.1996 0.00183 107.26258 0.01587 145.113 0.01822 136.39179 0.01904 124.37836 0.02139 117.42566 0.02622 107.69714

0.00107 158.14631 0.00125 116.07899 0.00132 134.92456 0.00153 119.33644 0.00183 107.12436 0.01598 145.74451 0.01835 136.97172 0.01918 125.07131 0.02155 117.97811 0.02641 107.97811

0.00107 157.61665 0.00124 113.85177 0.00133 134.92462 0.00154 118.54817 0.00184 106.10724 0.0161 146.37105 0.01848 137.54717 0.01932 125.76002 0.0217 118.52495 0.0266 108.25458

0.00108 157.19313 0.00124 112.48901 0.00133 133.86331 0.00154 117.59109 0.00185 105.2776 0.01622 146.99237 0.01861 138.11792 0.01946 126.4442 0.02185 119.06586 0.02679 108.52647

0.00108 156.51244 0.00123 110.81104 0.00133 134.08616 0.00154 117.23453 0.00185 104.47761 0.01633 147.60823 0.01874 138.68374 0.0196 127.12357 0.02201 119.60049 0.02698 108.79373

0.00108 156.42154 0.00123 108.90926 0.00134 133.811 0.00154 115.83637 0.00186 103.94437 0.01645 148.21832 0.01888 139.24437 0.01973 127.79784 0.02217 120.12847 0.02717 109.05627

0.00109 156.4215 0.00122 107.86182 0.00134 132.61894 0.00155 115.36731 0.00186 103.17408 0.01656 148.82236 0.01901 139.79953 0.01987 128.46668 0.02232 120.64942 0.02736 109.31402

0.00109 155.31712 0.00121 106.28534 0.00134 132.35677 0.00155 114.06296 0.00187 102.37407 0.01667 149.42002 0.01914 140.34894 0.02001 129.12975 0.02247 121.16293 0.02755 109.56687

0.00109 154.3791 0.00121 104.72939 0.00135 131.93761 0.00155 112.94644 0.00187 101.7321 0.01679 150.01097 0.01927 140.89227 0.02015 129.78671 0.02263 121.66856 0.02774 109.81472

0.00109 153.75869 0.0012 103.17335 0.00135 130.6013 0.00155 111.9048 0.00187 100.56675 0.01691 150.59483 0.0194 141.42919 0.02029 130.43718 0.02279 122.16586 0.02793 110.05746

0.0011 153.72851 0.00118 101.69882 0.00135 129.94633 0.00155 110.48798 0.00188 100.07293 0.01702 151.17122 0.01954 141.95935 0.02042 131.08075 0.02294 122.65434 0.02812 110.29495

0.0011 152.85098 0.00118 99.95973 0.00135 128.83266 0.00155 109.4089 0.00188 99.1051 0.01714 151.73971 0.01967 142.48235 0.02056 131.71702 0.0231 123.13349 0.02831 110.52705

0.0011 151.8828 0.00117 98.67835 0.00136 127.99433 0.00155 108.11406 0.00188 98.4236 0.01725 152.29987 0.0198 142.9978 0.0207 132.34552 0.02325 123.60275 0.0285 110.75361

0.0011 151.42884 0.00116 96.69514 0.00136 126.56634 0.00155 106.86608 0.00188 97.21877 0.01736 152.8512 0.01993 143.50524 0.02084 132.9658 0.0234 124.06155 0.02869 110.97446

0.0011 151.30779 0.00115 95.11887 0.00136 125.38721 0.00155 105.60866 0.00188 96.92247 0.01748 153.3932 0.02006 144.0042 0.02098 133.57733 0.02356 124.50928 0.02888 111.1894

0.0011 150.82378 0.00115 93.17642 0.00136 124.20814 0.00155 104.52017 0.00188 95.74723 0.0176 153.92532 0.0202 144.49418 0.02111 134.17959 0.02372 124.94527 0.02907 111.39823

0.0011 149.88576 0.00114 91.65102 0.00136 123.36968 0.00155 103.23471 0.00189 95.34244 0.01771 154.44696 0.02033 144.97464 0.02125 134.77199 0.02387 125.36881 0.02926 111.60073

0.0011 149.15948 0.00113 90.11531 0.00136 122.57058 0.00155 102.19316 0.00189 94.16718 0.01783 154.95749 0.02046 145.44498 0.02139 135.35392 0.02403 125.77917 0.02945 111.79666

0.0011 148.88708 0.00112 88.46784 0.00136 121.22119 0.00155 101.00153 0.00189 93.26848 0.01794 155.45621 0.02059 145.90458 0.02153 135.92473 0.02418 126.17553 0.02964 111.98574

0.0011 147.58612 0.00111 87.00332 0.00136 120.67105 0.00155 99.81916 0.00188 92.29075 0.01806 155.94241 0.02072 146.35276 0.02167 136.48371 0.02433 126.55705 0.02983 112.16768

0.0011 145.96718 0.00109 85.57955 0.00136 119.78004 0.00154 98.77769 0.00188 90.85871 0.01817 156.41527 0.02086 146.78879 0.0218 137.0301 0.02449 126.92279 0.03002 112.34217

0.0011 145.57377 0.00108 84.10491 0.00136 119.22988 0.00154 97.37018 0.00188 89.95999 0.01828 156.87396 0.02099 147.21188 0.02194 137.56308 0.02465 127.27177 0.03021 112.50885

0.0011 144.81731 0.00107 82.67092 0.00136 117.86754 0.00154 96.06586 0.00188 88.81441 0.0184 157.31753 0.02112 147.62118 0.02208 138.08179 0.0248 127.60294 0.0304 112.66734

0.0011 143.53137 0.00106 81.33867 0.00135 117.02898 0.00153 94.64902 0.00188 87.62924 0.01852 157.74499 0.02125 148.01577 0.02222 138.58527 0.02496 127.91514 0.03059 112.81723

0.0011 142.32115 0.00105 80.07762 0.00135 116.13811 0.00153 92.75362 0.00188 86.57251 0.01863 158.15525 0.02138 148.39464 0.02236 139.0725 0.02511 128.20714 0.03078 112.95806

0.0011 141.47371 0.00104 78.70471 0.00135 115.91547 0.00153 91.25234 0.00187 85.46648 0.01875 158.54714 0.02152 148.75671 0.02249 139.54238 0.02526 128.47762 0.03097 113.08933

0.0011 139.55238 0.00103 77.55551 0.00135 115.57482 0.00152 89.8354 0.00187 84.68625 0.01886 158.91938 0.02165 149.1008 0.02263 139.99372 0.02542 128.72514 0.03116 113.21049

0.0011 138.81101 0.00102 76.36564 0.00134 114.63155 0.00152 88.39038 0.00187 83.34304 0.01897 159.27057 0.02178 149.42563 0.02277 140.42519 0.02558 128.94813 0.03135 113.32094

0.0011 137.82773 0.00101 75.48089 0.00134 113.80615 0.00151 86.75772 0.00186 82.27654 0.01909 159.5992 0.02191 149.7298 0.02291 140.8354 0.02573 129.14491 0.03154 113.42001

0.0011 136.32991 9.94482E-4 74.30113 0.00134 113.37396 0.00151 84.95619 0.00186 81.08149 0.01921 159.90361 0.02204 150.01179 0.02305 141.22279 0.02588 129.31364 0.03173 113.50699

0.0011 135.36151 9.8308E-4 73.1417 0.00134 112.32577 0.0015 83.29535 0.00185 79.61 0.01932 160.18199 0.02218 150.26991 0.02318 141.58567 0.02604 129.45231 0.03192 113.58108

0.0011 134.8472 9.71817E-4 71.77902 0.00133 111.35629 0.00149 81.50304 0.00185 78.10895 0.01944 160.43234 0.02231 150.50233 0.02332 141.92219 0.0262 129.55875 0.03211 113.64138

0.0011 133.99241 9.60625E-4 70.59927 0.00133 110.66199 0.00149 80.1894 0.00184 76.8151 0.01955 160.65248 0.02244 150.70704 0.02346 142.23032 0.02635 129.63055 0.0323 113.68692

0.0011 132.89548 9.49466E-4 69.0942 0.00133 110.11174 0.00148 78.7162 0.00184 75.63978 0.01966 160.84 0.02257 150.88181 0.0236 142.50783 0.02651 129.66512 0.03249 113.71663

0.0011 131.82126 9.38369E-4 68.11791 0.00133 109.46983 0.00147 77.35562 0.00183 74.59313 0.01978 160.99225 0.0227 151.02419 0.02374 142.75225 0.02666 129.65956 0.03268 113.72929

0.0011 131.38267 9.27317E-4 66.64328 0.00132 109.16848 0.00147 76.14527 0.00183 72.97334 0.01989 161.10628 0.02284 151.13148 0.02387 142.96085 0.02681 129.61071 0.03287 113.72358

0.0011 130.70177 9.16376E-4 65.45342 0.00132 108.55285 0.00146 74.77526 0.00182 71.78831 0.02001 161.17884 0.02297 151.20065 0.02401 143.13061 0.02697 129.5151 0.03306 113.69801

0.0011 130.29311 9.0542E-4 63.50075 0.00132 108.15971 0.00145 73.7713 0.00181 70.49446 0.02013 161.2063 0.0231 151.22836 0.02415 143.25818 0.02713 129.36885 0.03325 113.65091

0.0011 129.5217 8.94404E-4 62.37186 0.00131 107.63578 0.00144 72.54209 0.00181 69.25011 0.02024 161.18461 0.02323 151.21088 0.02429 143.3398 0.02728 129.1677 0.03344 113.58042

0.0011 129.56705 8.83436E-4 60.92777 0.00131 106.91511 0.00144 71.82896 0.0018 67.82791 0.02036 161.10924 0.02336 151.14401 0.02443 143.37127 0.02744 128.90688 0.03363 113.48445

0.0011 128.59884 8.72475E-4 59.50894 0.00131 106.31259 0.00143 70.44953 0.00179 66.69228 0.02047 160.97512 0.0235 151.02307 0.02456 143.34791 0.02759 128.5811 0.03382 113.36062

0.0011 128.14492 8.6144E-4 58.46664 0.00131 105.76228 0.00142 69.90538 0.00178 65.29974 0.02058 160.77651 0.02363 150.84277 0.0247 143.26441 0.02774 128.18443 0.03401 113.20627

0.0011 128.34151 8.50319E-4 56.52418 0.00131 104.97631 0.00141 68.48844 0.00178 63.83315 0.0207 160.50697 0.02376 150.59712 0.02484 143.11478 0.0279 127.71022 0.0342 113.01837

0.0011 127.87261 8.39139E-4 55.43605 0.00131 104.38688 0.00141 67.30617 0.00177 62.9888 0.02082 160.15914 0.02389 150.27936 0.02498 142.89224 0.02806 127.151 0.03439 112.79345

0.0011 127.85755 8.27884E-4 53.39198 0.00131 103.45661 0.0014 66.04888 0.00176 61.76419 0.02093 159.72469 0.02402 149.88174 0.02512 142.58905 0.02821 126.49832 0.03458 112.52756

0.00109 127.58514 8.16546E-4 52.18157 0.00131 102.40857 0.00139 64.50057 0.00175 60.81612 0.02105 159.19404 0.02416 149.39542 0.02525 142.19636 0.02837 125.74262 0.03477 112.21614

0.00109 127.05559 8.05132E-4 50.30031 0.00131 100.98055 0.00138 63.28079 0.00174 59.75934 0.02116 158.55624 0.02429 148.81025 0.02539 141.704 0.02852 124.87304 0.03496 111.85395

0.00109 126.55632 7.9363E-4 48.98839 0.0013 99.343 0.00137 61.96714 0.00173 59.11748 0.02127 157.79859 0.02442 148.11449 0.02553 141.10024 0.02867 123.87715 0.03515 111.43489

0.00109 125.98157 7.81969E-4 47.72722 0.0013 97.27306 0.00137 60.68168 0.00172 57.89277 0.02139 156.90641 0.02455 147.2945 0.02567 140.37143 0.02883 122.74073 0.03534 110.95185

0.00109 125.43677 7.70181E-4 46.31367 0.0013 95.43891 0.00136 59.48988 0.00171 56.71756 0.02151 155.86252 0.02468 146.33439 0.02581 139.50168 0.02899 121.44741 0.03553 110.39647

0.00109 124.63488 7.58267E-4 44.86959 0.0013 93.05452 0.00135 57.93234 0.00171 55.40418 0.02162 154.64681 0.02482 145.21548 0.02594 138.47229 0.02914 119.97826 0.03572 109.75889

0.00109 124.49884 7.46325E-4 43.28303 0.00129 90.81438 0.00134 56.34652 0.0017 53.65593 0.02174 153.23548 0.02495 143.91569 0.02608 137.2612 0.0293 118.31131 0.03591 109.02738

0.00108 123.69692 7.34485E-4 41.94059 0.00129 88.62652 0.00133 54.95776 0.00169 52.27342 0.02185 151.60026 0.02508 142.40877 0.02622 135.84215 0.02945 116.42089 0.0361 108.18789

0.00108 122.19914 7.22668E-4 40.26265 0.00129 85.70508 0.00133 53.19373 0.00168 50.85132 0.02196 149.70732 0.02521 140.66325 0.02636 134.18366 0.0296 114.27696 0.03629 107.22343

0.00108 120.82241 7.10862E-4 38.92019 0.00128 82.56082 0.00132 51.69245 0.00167 49.34024 0.02208 147.51581 0.02534 138.64113 0.0265 132.24773 0.02976 111.84408 0.03648 106.11332

0.00108 119.73309 6.9911E-4 37.83199 0.00128 80.2683 0.00131 50.43506 0.00166 48.47115 0.02219 144.97604 0.02548 136.29614 0.02663 129.98809 0.02992 109.08037 0.03667 104.83211

0.00108 119.15816 -- -- 0.00128 77.18964 0.0013 49.15893 0.00165 47.30573 0.02231 142.02705 0.02561 133.57142 0.02677 127.34789 0.03007 105.93604 0.03686 103.34818

0.00108 117.40298 -- -- 0.00127 74.77913 0.00129 47.74209 0.00164 46.69343 0.02243 138.59336 0.02574 130.39649 0.02691 124.25663 0.03022 102.35179 0.03705 101.6218

0.00108 115.20936 -- -- 0.00127 72.30308 0.00128 46.82252 0.00163 45.29109 0.02254 134.58065 0.02587 126.68312 0.02705 120.62609 0.03038 98.25678 0.03724 99.60244

0.00107 113.99902 -- -- 0.00126 70.06284 0.00127 45.67772 0.00162 44.2739 0.02266 129.86996 0.026 122.3198 0.02719 116.34477 0.03054 93.56659 0.03743 97.225

0.00107 112.6979 -- -- 0.00126 68.41201 0.00126 43.9418 0.00161 43.69125 0.02277 124.31 0.02614 117.16437 0.02732 111.27049 0.03069 88.18115 0.03762 94.40454

0.00107 111.53299 -- -- 0.00125 66.48621 0.00125 42.79712 0.0016 42.26901 0.02288 117.70718 0.02627 111.03431 0.02746 105.22074 0.03085 81.98372 0.03781 91.02868

0.00107 109.08197 -- -- 0.00125 64.79627 0.00124 41.07054 0.00159 41.48886

0.00107 107.78079 -- -- 0.00124 63.19805 0.00123 39.785 0.00158 40.47162

0.00106 105.8747 -- -- 0.00123 61.49496 0.00122 38.31193 0.00157 39.57298

0.00106 103.96844 -- -- 0.00123 59.75251 0.00121 37.25157 0.00156 38.79281

0.00106 101.09378 -- -- 0.00122 58.80933 0.0012 35.7221 0.00155 38.32852

0.00106 99.09665 -- -- 0.00121 58.11493 0.00119 34.84003 0.00154 37.58785

0.00106 97.25087 -- -- 0.00121 57.13234 0.00118 33.48888 0.00153 36.70888

0.00105 95.04224 -- -- 0.00121 55.84844 0.00117 32.28777 0.00152 36.21518

0.00105 92.3642 -- -- 0.0012 55.63875 0.00116 31.13367 0.00151 34.95092

0.00104 90.47282 -- -- 0.00119 54.60388 0.00115 30.58016 0.0015 33.835

0.00104 88.06741 -- -- 0.00118 54.093 0.00114 29.32274 0.00149 32.77827

0.00103 86.16115 -- -- 0.00117 52.53404 0.00114 28.28126 0.00148 31.44509

0.00103 84.07331 -- -- 0.00116 51.38116 0.00113 27.49304 0.00147 30.77342

0.00102 82.3184 -- -- 0.00115 49.58636 0.00112 27.39917 0.00146 29.6575

0.00102 80.29103 -- -- 0.00114 48.55139 -- -- 0.00144 28.77845

0.00102 78.43022 -- -- 0.00113 46.92687 -- -- 0.00143 28.37359

0.00101 76.67516 -- -- 0.00112 45.77399 -- -- 0.00142 27.54406

1E-3 75.17734 -- -- 0.00112 44.58176 -- -- 0.00141 26.45765

9.92E-4 73.95183 -- -- -- -- -- -- -- --

9.92E-4 72.95335 -- -- -- -- -- -- -- --

9.84E-4 71.63697 -- -- -- -- -- -- -- --

9.76E-4 71.09237 -- -- -- -- -- -- -- --

9.68E-4 68.94406 -- -- -- -- -- -- -- --

9.6E-4 68.12699 -- -- -- -- -- -- -- --

9.52E-4 66.96213 -- -- -- -- -- -- -- --

4.44576E-4 34.45754

**(d) *β*=90°**

8.11888E-9 -0.28575 1.49122E-7 0.60398 6.16373E-8 0.25194 1.33548E-7 -0.12541 3.08683E-7 0.03919 8.9E-5 2.28138 1.2E-4 2.23545 1.8E-4 3.08195 1.95E-4 3.00083 2E-4 3.29558

8.23487E-8 -0.29561 4.44054E-7 1.42227 2.1076E-7 0.46356 4.40491E-7 -0.21498 6.59866E-7 0.11757 1.78E-4 4.52278 2.4E-4 4.4358 3.6E-4 6.10503 3.9E-4 5.94283 4E-4 6.47914

2.54006E-7 -0.0197 9.09647E-7 2.45488 4.47367E-7 1.01783 8.05593E-7 -0.18363 1.06026E-6 0.10973 2.67E-4 6.72464 3.6E-4 6.60139 5.4E-4 9.06977 5.85E-4 8.82655 6E-4 9.5609

5.8688E-7 -0.23648 1.5923E-6 3.32188 7.4959E-7 1.35039 1.22777E-6 0.45685 1.48974E-6 0.35271 3.56E-4 8.88744 4.8E-4 8.73256 7.2E-4 11.97676 7.8E-4 11.65257 8E-4 12.5466

1.02646E-6 -0.18722 2.55828E-6 4.39346 1.1731E-6 1.35039 1.77489E-6 0.83307 1.97736E-6 0.52515 4.45E-4 11.01166 6E-4 10.82969 9E-4 14.82658 9.75E-4 14.42147 1E-3 15.44051

1.49852E-6 0.17736 3.76783E-6 5.10459 1.67216E-6 2.14651 2.33923E-6 1.15555 2.49631E-6 0.80732 5.34E-4 13.0978 7.2E-4 12.89314 0.00108 17.61982 0.00117 17.13385 0.0012 18.24615

2.08307E-6 -0.04927 5.2226E-6 6.76066 2.17718E-6 1.8341 2.9402E-6 1.63927 2.95263E-6 1.16787 6.23E-4 15.14636 8.4E-4 14.92329 0.00126 20.3571 0.00136 19.79032 0.0014 20.96658

2.79406E-6 -0.38429 6.93254E-6 7.89068 2.89297E-6 2.13643 3.62301E-6 1.73781 3.38433E-6 1.46572 7.12E-4 17.15786 9.6E-4 16.92054 0.00144 23.03905 0.00156 22.39152 0.0016 23.60458

3.57463E-6 -0.29561 8.9838E-6 9.0207 3.64057E-6 2.62015 4.3726E-6 2.4813 3.75342E-6 1.64599 8.01E-4 19.13284 0.00108 18.88529 0.00162 25.66631 0.00176 24.93808 0.0018 26.16267

4.65445E-6 -0.04927 1.14012E-5 10.38452 4.26491E-6 2.86201 5.24497E-6 3.03669 3.98157E-6 1.9987 8.9E-4 21.07184 0.0012 20.81793 0.0018 28.23952 0.00195 27.43065 0.002 28.64322

6.03466E-6 -1.16272 1.41683E-5 11.77756 4.97274E-6 3.76898 6.18411E-6 3.60102 4.11354E-6 2.37493 9.79E-4 22.97541 0.00132 22.71889 0.00198 30.75936 0.00215 29.86991 0.0022 31.04848

7.47285E-6 -0.47297 1.73661E-5 13.43364 5.80584E-6 4.67596 7.14479E-6 4.44306 4.23657E-6 2.97846 0.00107 24.84411 0.00144 24.58857 0.00216 33.22649 0.00234 32.25651 0.0024 33.38058

8.925E-6 -0.15766 2.09733E-5 14.93384 6.79004E-6 5.43177 8.26487E-6 5.33883 4.45801E-6 3.55064 0.00116 26.67852 0.00156 26.42742 0.00234 35.6416 0.00253 34.59116 0.0026 35.64156

1.03701E-5 0.94594 2.50078E-5 16.73603 7.94723E-6 6.24804 9.53572E-6 5.96588 4.68171E-6 4.27958 0.00125 28.47921 0.00168 28.23585 0.00252 38.00538 0.00273 36.87453 0.0028 37.8334

1.18339E-5 1.87217 2.95511E-5 18.71357 9.33307E-6 7.31626 1.08841E-5 6.52126 4.83605E-6 4.74986 0.00134 30.24677 0.0018 30.0143 0.0027 40.31853 0.00293 39.10734 0.003 39.95798

1.34635E-5 2.46338 3.45616E-5 20.50602 1.09635E-5 8.03176 1.2351E-5 7.56036 5.00604E-6 5.51015 0.00142 31.98179 0.00192 31.76322 0.00288 42.58176 0.00312 41.2903 0.0032 42.01717

1.52496E-5 3.67537 3.99831E-5 22.47382 1.27628E-5 9.16044 1.39083E-5 8.17845 5.18052E-6 6.5918 0.00151 33.68486 0.00204 33.48306 0.00306 44.7958 0.00331 43.42412 0.0034 44.01274

1.7139E-5 4.70014 4.58436E-5 24.87026 1.46279E-5 10.48059 1.5511E-5 9.74606 5.41539E-6 7.32858 0.0016 35.35658 0.00216 35.17425 0.00324 46.96136 0.00351 45.50952 0.0036 45.94646

1.91548E-5 5.67565 5.21117E-5 26.75038 1.64492E-5 11.7302 1.712E-5 10.8389 5.74421E-6 8.62186 0.00169 36.99756 0.00228 36.83726 0.00342 49.07917 0.00371 47.54725 0.0038 47.82001

2.12425E-5 6.6807 5.8625E-5 28.92275 1.83282E-5 13.28214 1.87569E-5 12.17362 6.14012E-6 9.8054 0.00178 38.60841 0.0024 38.47255 0.0036 51.14997 0.0039 49.53802 0.004 49.63507

2.34207E-5 7.96167 6.53852E-5 31.71858 2.02608E-5 14.76353 2.04307E-5 13.51728 6.68592E-6 11.33383 0.00187 40.18974 0.00252 40.08056 0.00378 53.17449 0.00409 51.4826 0.0042 51.39325

2.5745E-5 9.45941 7.24255E-5 33.74483 2.23644E-5 15.56973 2.22637E-5 14.99531 7.36144E-6 12.55656 0.00196 41.74216 0.00264 41.66177 0.00396 55.15349 0.00429 53.38172 0.0044 53.09615

2.81969E-5 10.83891 7.96679E-5 36.32635 2.47961E-5 16.81933 2.42108E-5 16.36586 8.21367E-6 14.01443 0.00205 43.26628 0.00276 43.21664 0.00414 57.08772 0.00449 55.23612 0.0046 54.74532

3.07694E-5 12.514 8.71008E-5 39.43392 2.74604E-5 18.3612 2.63348E-5 17.74535 9.21578E-6 15.73097 0.00214 44.76272 0.00288 44.74564 0.00432 58.97791 0.00468 57.04657 0.0048 56.34227

3.34974E-5 14.1497 9.47657E-5 42.27846 3.03594E-5 19.99375 2.87557E-5 19.08902 1.03409E-5 17.56507 0.00223 46.23209 0.003 46.24922 0.0045 60.82483 0.00488 58.81382 0.005 57.88851

3.64155E-5 15.88392 1.02661E-4 45.12299 3.34949E-5 21.52553 3.14569E-5 20.97015 1.18172E-5 19.09349 0.00231 47.67501 0.00312 47.72787 0.00468 62.62923 0.00507 60.53861 0.0052 59.38547

3.95309E-5 17.49991 1.10757E-4 47.93831 3.69148E-5 22.94645 3.45823E-5 22.60046 1.37499E-5 20.5984 0.0024 49.09208 0.00324 49.18204 0.00486 64.39186 0.00526 62.22172 0.0054 60.8346

4.27634E-5 19.11589 1.19102E-4 51.0751 4.06249E-5 24.29684 3.81299E-5 24.36514 1.59755E-5 22.38548 0.00249 50.48393 0.00336 50.6122 0.00504 66.11349 0.00546 63.8639 0.0056 62.23729

4.61014E-5 20.66289 1.27738E-4 53.83197 4.46592E-5 25.72785 4.20265E-5 26.23732 1.853E-5 24.3058 0.00258 51.85115 0.00348 52.01883 0.00522 67.79487 0.00566 65.46591 0.0058 63.59491

4.97015E-5 22.49566 1.36623E-4 56.8129 4.90355E-5 27.12862 4.63022E-5 28.28865 2.15788E-5 26.23396 0.00267 53.19436 0.0036 53.40238 0.0054 69.43675 0.00585 67.0285 0.006 64.90879

5.35685E-5 23.63867 1.4579E-4 59.89124 5.37914E-5 28.95265 5.0985E-5 30.30415 2.4925E-5 27.79373 0.00276 54.51416 0.00372 54.76333 0.00558 71.0399 0.00605 68.55243 0.0062 66.18026

5.76499E-5 25.51085 1.5521E-4 62.51172 5.89153E-5 30.88752 5.59736E-5 32.22111 2.85354E-5 29.62784 0.00285 55.81113 0.00384 56.10214 0.00576 72.60507 0.00624 70.03846 0.0064 67.41059

6.19448E-5 27.35346 1.64843E-4 65.58031 6.42778E-5 32.79218 6.11949E-5 34.58597 3.249E-5 31.47762 0.00294 57.08589 0.00396 57.41928 0.00594 74.13301 0.00643 71.48734 0.0066 68.60104

6.63801E-5 29.47197 1.74722E-4 68.44434 6.98012E-5 35.34178 6.66574E-5 37.34496 3.67378E-5 33.78201 0.00303 58.33901 0.00408 58.71519 0.00612 75.62447 0.00663 72.89983 0.0068 69.75284

7.1001E-5 31.46239 1.84816E-4 71.3668 7.53983E-5 38.00224 7.23892E-5 39.62023 4.12294E-5 35.718 0.00312 59.57109 0.0042 59.99035 0.0063 77.08019 0.00683 74.27666 0.007 70.8672

7.5887E-5 33.27544 1.95318E-4 74.26979 8.10988E-5 39.72549 7.83234E-5 42.2807 4.5882E-5 37.95969 0.0032 60.7827 0.00432 61.24521 0.00648 78.50093 0.00702 75.6186 0.0072 71.9453

8.10299E-5 35.07865 2.06334E-4 77.33445 8.70974E-5 42.69835 8.44753E-5 45.01282 5.06465E-5 40.23272 0.00329 61.97441 0.00444 62.48022 0.00666 79.88742 0.00722 76.92637 0.0074 72.98829

8.64129E-5 37.10847 2.17719E-4 80.89407 9.33845E-5 44.88517 9.0892E-5 47.12685 5.56502E-5 42.42738 0.00338 63.1468 0.00456 63.69583 0.00684 81.2404 0.00741 78.20073 0.0076 73.99728

9.20192E-5 38.85255 2.294E-4 83.53404 9.98683E-5 47.8278 9.76232E-5 49.62607 6.09293E-5 44.25364 0.00347 64.30041 0.00468 64.89248 0.00702 82.5606 0.0076 79.44239 0.0078 74.97339

9.78096E-5 40.66561 2.41406E-4 85.6577 1.06581E-4 50.43787 1.04721E-4 52.35819 6.63513E-5 46.32289 0.00356 65.43581 0.0048 66.07064 0.0072 83.84874 0.0078 80.65209 0.008 75.91769

1.03835E-4 42.79398 2.53736E-4 87.76189 1.13607E-4 52.91693 1.12128E-4 55.10823 7.19322E-5 48.56457 0.00365 66.55354 0.00492 67.23072 0.00738 85.10556 0.008 81.83055 0.0082 76.83124

1.10111E-4 44.6563 2.66335E-4 89.75891 1.21E-4 55.2146 1.19859E-4 57.39246 7.7748E-5 50.57896 0.00374 67.65415 0.00504 68.37317 0.00756 86.33176 0.00819 82.9785 0.0084 77.71505

1.16584E-4 46.45949 2.79288E-4 91.78516 1.28702E-4 57.65335 1.27943E-4 59.87377 8.40671E-5 52.41306 0.00383 68.73815 0.00516 69.49842 0.00774 87.52805 0.00839 84.09663 0.0086 78.57012

1.23254E-4 48.44006 2.92663E-4 93.61658 1.36801E-4 60.0518 1.36361E-4 61.90718 9.08738E-5 54.01986 0.00392 69.80607 0.00528 70.60689 0.00792 88.69514 0.00858 85.18566 0.0088 79.39745

1.30224E-4 50.47975 3.0645E-4 94.92195 1.45279E-4 62.39985 1.45028E-4 64.21829 9.81077E-5 55.87748 0.004 70.85844 0.0054 71.69901 0.0081 89.83373 0.00877 86.24628 0.009 80.19798

1.37474E-4 52.18441 3.20643E-4 96.4319 1.5402E-4 64.91923 1.53972E-4 66.20691 1.05928E-4 57.20994 0.00409 71.89575 0.00552 72.7752 0.00828 90.9445 0.00897 87.27919 0.0092 80.97264

1.44914E-4 54.89414 3.35201E-4 97.99055 1.63067E-4 67.57969 1.63236E-4 68.08804 1.14271E-4 58.69918 0.00418 72.9185 0.00564 73.83586 0.00846 92.02813 0.00916 88.28508 0.0094 81.72235

1.52563E-4 56.77617 3.50201E-4 99.17902 1.72543E-4 70.01844 1.72783E-4 70.06771 1.23082E-4 60.31381 0.00427 73.92718 0.00576 74.88141 0.00864 93.08531 0.00936 89.26461 0.0096 82.44798

1.60426E-4 58.9538 3.65673E-4 100.45517 1.82525E-4 73.10407 1.82579E-4 72.16383 1.32387E-4 61.74818 0.00436 74.92228 0.00588 75.91224 0.00882 94.11671 0.00955 90.21846 0.0098 83.1504

1.68525E-4 61.09202 3.81482E-4 101.70209 1.93073E-4 76.40758 1.92782E-4 74.33162 1.42254E-4 63.37849 0.00445 75.90426 0.006 76.92875 0.009 95.12297 0.00975 91.14729 0.01 83.83044

1.77002E-4 63.1317 3.97688E-4 103.2705 2.04132E-4 78.39284 2.03611E-4 76.83196 1.52966E-4 64.79718 0.00454 76.87359 0.00612 77.93134 0.00918 96.10475 0.00995 92.05176 0.0102 84.48894

1.85906E-4 65.89893 4.14287E-4 104.60508 2.15564E-4 81.03315 2.14982E-4 78.55969 1.64657E-4 66.76235 0.00463 77.83072 0.00624 78.92039 0.00936 97.06271 0.01014 92.9325 0.0104 85.12668

1.94953E-4 68.82705 4.31219E-4 106.16373 2.27401E-4 83.35097 2.26803E-4 80.32436 1.7715E-4 68.85729 0.00472 78.77609 0.00636 79.89627 0.00954 97.99746 0.01034 93.79016 0.0106 85.74444

2.04072E-4 69.00442 4.48484E-4 107.4204 2.39678E-4 85.29592 2.39145E-4 81.84718 1.90368E-4 69.93111 0.00481 79.71014 0.00648 80.85936 0.00972 98.90963 0.01053 94.62536 0.0108 86.34297

2.13399E-4 70.43318 4.65965E-4 108.82318 2.52451E-4 87.74475 2.52164E-4 83.10127 2.04332E-4 71.31844 0.0049 80.63329 0.0066 81.81003 0.0099 99.79986 0.01073 95.43873 0.011 86.92301

2.23054E-4 72.53199 4.83648E-4 110.51822 2.65669E-4 89.49824 2.65945E-4 84.30162 2.19029E-4 72.70577 0.00498 81.54596 0.00672 82.74865 0.01008 100.66873 0.01092 96.23088 0.0112 87.48527

2.33104E-4 74.73918 5.016E-4 111.92101 2.79347E-4 91.45328 2.80519E-4 85.19739 2.34488E-4 74.04609 0.00507 82.44856 0.00684 83.67555 0.01026 101.51685 0.01112 97.00239 0.0114 88.03044

2.4349E-4 77.03507 5.19816E-4 112.98284 2.93587E-4 93.41839 2.95867E-4 86.21858 2.50753E-4 75.35504 0.00516 83.34148 0.00696 84.5911 0.01044 102.34481 0.01131 97.75388 0.0116 88.5592

2.5425E-4 79.54772 5.38231E-4 114.15183 3.08418E-4 95.05094 3.11981E-4 87.55329 2.67802E-4 76.49156 0.00525 84.2251 0.00708 85.49563 0.01062 103.1532 0.01151 98.48591 0.0118 89.0722

2.65383E-4 81.10459 5.56759E-4 114.89219 3.23773E-4 96.77419 3.28861E-4 88.67302 2.85583E-4 77.57322 0.00534 85.09982 0.0072 86.38949 0.0108 103.94257 0.0117 99.19907 0.012 89.57007

2.76821E-4 83.56799 5.75307E-4 116.17808 3.39599E-4 98.51761 3.46354E-4 89.90023 3.04059E-4 79.11731 0.00543 85.96599 0.00732 87.273 0.01098 104.71349 0.01189 99.89391 0.0122 90.05344

2.8852E-4 85.79488 5.93791E-4 117.50294 3.55919E-4 100.26101 3.64529E-4 91.37826 3.23083E-4 80.3714 0.00552 86.82397 0.00744 88.14648 0.01116 105.46652 0.01209 100.57099 0.0124 90.52289

3.00439E-4 87.95281 6.12128E-4 118.55502 3.72732E-4 101.36954 3.83429E-4 92.6861 3.42595E-4 81.93117 0.00561 87.67412 0.00756 89.01025 0.01134 106.20219 0.01229 101.23086 0.0126 90.97901

3.12557E-4 90.61327 6.30308E-4 119.94807 3.90246E-4 103.38504 4.02971E-4 94.62098 3.62626E-4 83.2558 0.0057 88.51677 0.00768 89.86462 0.01152 106.92103 0.01248 101.87404 0.0128 91.42236

3.24838E-4 92.80075 6.48385E-4 120.96119 4.08443E-4 104.58427 4.23185E-4 96.47523 3.83091E-4 84.81557 0.00578 89.35225 0.0078 90.7099 0.0117 107.62357 0.01268 102.50107 0.013 91.85348

3.37234E-4 95.36269 6.66309E-4 122.40295 4.27157E-4 106.09589 4.44083E-4 97.51434 4.03981E-4 86.21858 0.00587 90.18089 0.00792 91.54638 0.01188 108.31032 0.01287 103.11245 0.0132 92.27291

3.49689E-4 98.22021 6.83927E-4 123.24073 4.46378E-4 107.5672 4.65573E-4 99.09091 4.25269E-4 87.40213 0.00596 91.003 0.00804 92.37435 0.01206 108.98178 0.01307 103.70869 0.0134 92.68114

3.62137E-4 100.6836 7.01214E-4 124.49739 4.66098E-4 109.29046 4.87499E-4 100.981 4.46912E-4 88.69541 0.00605 91.81888 0.00816 93.1941 0.01224 109.63843 0.01326 104.29029 0.0136 93.07868

3.74539E-4 103.45245 7.18194E-4 125.28645 4.8628E-4 110.43929 5.09894E-4 102.42319 4.68952E-4 89.91814 0.00614 92.62883 0.00828 94.0059 0.01242 110.28077 0.01346 104.85773 0.0138 93.46599

3.86985E-4 105.70892 7.34877E-4 126.14371 5.06938E-4 111.97106 5.32706E-4 103.84749 4.91465E-4 91.13304 0.00623 93.43313 0.0084 94.81002 0.0126 110.90926 0.01365 105.41149 0.014 93.84355

3.99466E-4 107.98508 7.51259E-4 126.97175 5.28082E-4 112.97882 5.55836E-4 105.27178 5.14366E-4 92.34793 0.00632 94.23205 0.00852 95.60674 0.01278 111.52437 0.01385 105.95202 0.0142 94.2118

4.11906E-4 110.42876 7.6724E-4 127.0984 5.496E-4 114.26873 5.79286E-4 106.25713 5.37625E-4 93.60203 0.00641 95.02587 0.00864 96.39629 0.01296 112.12654 0.01404 106.47978 0.0144 94.57116

4.24341E-4 113.01039 7.82776E-4 127.12761 5.71534E-4 115.22611 6.02935E-4 107.48434 5.61335E-4 94.49557 0.0065 95.81485 0.00876 97.17895 0.01314 112.71621 0.01423 106.99523 0.0146 94.92206

4.36729E-4 115.07964 7.97961E-4 127.51727 5.93807E-4 116.44547 6.26743E-4 108.56823 5.85276E-4 95.3264 0.00659 96.59922 0.00888 97.95494 0.01332 113.29383 0.01443 107.49878 0.0148 95.2649

4.49006E-4 117.26713 8.12898E-4 128.11152 6.16357E-4 117.1509 6.5066E-4 109.38339 6.09487E-4 96.36887 0.00667 97.37925 0.009 98.72451 0.0135 113.8598 0.01463 107.99087 0.015 95.60005

4.61166E-4 119.563 8.27532E-4 128.74472 6.39132E-4 118.5819 6.74631E-4 110.65538 6.3406E-4 97.13698 0.00676 98.15515 0.00912 99.48789 0.01368 114.41453 0.01482 108.47192 0.0152 95.9279

4.73253E-4 121.44503 8.41889E-4 129.25129 6.61981E-4 118.80361 6.98703E-4 111.51534 6.58812E-4 97.59944 0.00685 98.92716 0.00924 100.2453 0.01386 114.95844 0.01502 108.94232 0.0154 96.24881

4.8518E-4 123.26794 8.56036E-4 130.11829 6.84978E-4 119.81137 7.22868E-4 112.26779 6.83903E-4 98.33621 0.00694 99.69549 0.00936 100.99697 0.01404 115.49191 0.01521 109.40247 0.0156 96.5631

4.96899E-4 125.32733 8.69847E-4 131.22883 7.08208E-4 120.65788 7.46838E-4 113.23523 7.09237E-4 98.53216 0.00703 100.46036 0.00948 101.7431 0.01422 116.01531 0.01541 109.85276 0.0158 96.87113

5.08424E-4 126.96303 8.83374E-4 132.62185 7.31587E-4 121.0509 7.70666E-4 113.63833 7.34666E-4 98.83002 0.00712 101.22195 0.0096 102.4839 0.0144 116.52903 0.0156 110.29356 0.016 97.17321

5.19727E-4 128.86476 8.96725E-4 134.29741 7.54944E-4 121.84701 7.94498E-4 113.87123 7.60335E-4 99.08083 0.00721 101.98047 0.00972 103.21957 0.01458 117.03342 0.0158 110.72524 0.0162 97.46964

5.30817E-4 130.88473 9.0974E-4 136.30419 7.78144E-4 122.66329 8.18145E-4 114.53411 7.86083E-4 99.0338 0.0073 102.73611 0.00984 103.9503 0.01476 117.52884 0.01599 111.14815 0.0164 97.76072

5.41645E-4 132.30364 9.22444E-4 138.35964 8.01164E-4 122.98577 8.41623E-4 114.94618 8.11651E-4 99.16705 0.00739 103.48903 0.00996 104.67628 0.01494 118.01562 0.01619 111.56265 0.0166 98.04673

5.52095E-4 133.53534 9.34864E-4 140.51255 8.23944E-4 122.96561 8.64998E-4 114.80286 8.36969E-4 99.44922 0.00748 104.23941 0.01008 105.39768 0.01512 118.4941 0.01638 111.96906 0.0168 98.32794

5.62185E-4 133.89006 9.46911E-4 142.94794 8.46432E-4 122.47182 8.88278E-4 114.78494 8.62064E-4 99.28462 0.00756 104.98742 0.0102 106.1147 0.0153 118.9646 0.01657 112.36771 0.017 98.6046

5.71972E-4 134.87542 9.587E-4 145.10084 8.68664E-4 122.33074 9.11499E-4 114.71326 8.86896E-4 99.30813 0.00765 105.73321 0.01032 106.82748 0.01548 119.42743 0.01677 112.75893 0.0172 98.87697

5.81455E-4 135.17103 9.70259E-4 147.42907 8.906E-4 122.31058 9.34731E-4 114.65951 9.11385E-4 99.28461 0.00774 106.47693 0.01044 107.53619 0.01566 119.8829 0.01697 113.14302 0.0174 99.14528

5.90651E-4 135.84107 9.81559E-4 149.24099 9.12294E-4 122.53228 9.57975E-4 114.62368 9.35549E-4 99.20624 0.00783 107.21872 0.01056 108.241 0.01584 120.33131 0.01716 113.52028 0.0176 99.40975

5.99549E-4 136.16624 9.92702E-4 151.52053 9.33816E-4 122.42144 9.81206E-4 114.75805 9.59445E-4 99.4649 0.00792 107.95871 0.01068 108.94204 0.01602 120.77292 0.01735 113.891 0.0178 99.67061

6.08174E-4 137.11218 1E-3 152.81615 9.55324E-4 122.52221 0.001 114.96409 9.83147E-4 99.81761 0.00801 108.69704 0.0108 109.63947 0.0162 121.20803 0.01755 114.25547 0.018 99.92804

6.16591E-4 138.60992 0.00101 154.37481 9.76922E-4 123.72143 0.00103 115.34927 0.00101 100.33492 0.0081 109.43383 0.01092 110.33341 0.01638 121.63689 0.01775 114.61396 0.0182 100.18224

6.24822E-4 139.72336 0.00103 155.18337 9.98552E-4 123.99352 0.00105 115.85987 0.00103 100.95412 0.00819 110.1692 0.01104 111.02401 0.01656 122.05977 0.01794 114.96672 0.0184 100.4334

6.32881E-4 141.27038 0.00104 156.14778 0.00102 125.45476 0.00107 116.58545 0.00105 101.71441 0.00828 110.90324 0.01116 111.71139 0.01674 122.47691 0.01813 115.31401 0.0186 100.68169

6.40821E-4 142.63017 0.00105 156.42055 0.00104 126.38188 0.0011 117.51705 0.00108 102.79608 0.00837 111.63607 0.01128 112.39567 0.01692 122.88855 0.01833 115.65609 0.0188 100.92727

6.48694E-4 144.0885 0.00106 156.43029 0.00106 127.47026 0.00112 118.2695 0.0011 103.97961 0.00845 112.36778 0.0114 113.07696 0.0171 123.29492 0.01853 115.99318 0.019 101.1703

6.56515E-4 145.70449 0.00107 156.67384 0.00109 129.20359 0.00114 120.04314 0.00112 105.24937 0.00854 113.09846 0.01152 113.75537 0.01728 123.69625 0.01872 116.32552 0.0192 101.41091

6.64336E-4 147.48797 0.00108 155.61199 0.00111 130.91676 0.00117 121.17182 0.00115 106.51131 0.00863 113.82819 0.01164 114.43101 0.01746 124.09273 0.01892 116.65332 0.0194 101.64925

6.72099E-4 149.56706 0.00109 154.71576 0.00113 132.09583 0.00119 122.34529 0.00117 107.65565 0.00872 114.55706 0.01176 115.10397 0.01764 124.48459 0.01911 116.97679 0.0196 101.88544

6.79728E-4 150.3652 0.0011 154.16049 0.00115 133.2749 0.00121 123.88602 0.00119 108.93327 0.00881 115.28514 0.01188 115.77434 0.01782 124.87201 0.0193 117.29615 0.0198 102.11959

6.87392E-4 151.94179 0.00111 153.16687 0.00118 134.53457 0.00123 125.33719 0.00121 109.74058 0.0089 116.01248 0.012 116.44222 0.018 125.25517 0.0195 117.61157 0.02 102.35182

6.95046E-4 153.18332 0.00112 151.74458 0.0012 135.5121 0.00126 126.32254 0.00124 110.59493 0.00899 116.73916 0.01212 117.10767 0.01818 125.63426 0.0197 117.92326 0.0202 102.58222

7E-4 155.20331 0.00113 150.90681 0.00122 136.41907 0.00128 127.39747 0.00126 111.67658 0.00908 117.46522 0.01224 117.77078 0.01836 126.00945 0.01989 118.23138 0.0204 102.81088

7.07E-4 155.91769 0.00113 149.62092 0.00125 137.16482 0.0013 128.9203 0.00128 112.06848 0.00917 118.19073 0.01236 118.43161 0.01854 126.38089 0.02008 118.53611 0.0206 103.03788

7.21E-4 157.28242 0.00114 148.57857 0.00126 137.56792 0.00133 130.16542 0.00131 112.89149 0.00926 118.91571 0.01248 119.09023 0.01872 126.74873 0.02028 118.8376 0.0208 103.2633

7.28E-4 158.4944 0.00115 147.31217 0.00128 138.15744 0.00135 131.0657 0.00133 113.27555 0.00934 119.64022 0.0126 119.7467 0.0189 127.11313 0.02048 119.13602 0.021 103.48721

7.35E-4 159.30238 0.00116 146.02628 0.00131 138.48496 0.00136 132.04657 0.00135 113.60475 0.00943 120.36427 0.01272 120.40106 0.01908 127.47421 0.02067 119.4315 0.0212 103.70966

7.42E-4 160.04139 0.00116 145.03265 0.00133 138.7772 0.00139 133.06774 0.00138 114.07503 0.00952 121.08791 0.01284 121.05336 0.01926 127.8321 0.02087 119.72419 0.0214 103.93069

7.49E-4 160.84939 0.00117 143.56166 0.00134 138.8276 0.00142 132.80798 0.00139 114.34152 0.00961 121.81115 0.01296 121.70365 0.01944 128.18692 0.02106 120.01421 0.0216 104.15036

7.56E-4 161.30266 0.00118 142.2368 0.00137 139.3214 0.00143 133.17523 0.00142 114.3572 0.0097 122.53401 0.01308 122.35196 0.01962 128.53878 0.02126 120.30168 0.0218 104.36868

7.63E-4 161.78548 0.00118 141.41852 0.00139 140.01674 0.00146 133.47084 0.00144 114.41206 0.00979 123.2565 0.0132 122.99832 0.0198 128.88779 0.02145 120.58672 0.022 104.5857

7.7E-4 162.29787 0.00119 140.30797 0.00142 140.66169 0.00148 133.77541 0.00146 114.47476 0.00988 123.97863 0.01332 123.64274 0.01998 129.23402 0.02165 120.86943 0.0222 104.80142

7.77E-4 162.85951 0.00119 139.09027 0.00143 140.98418 0.0015 133.72166 0.00149 114.14558 0.00997 124.7004 0.01344 124.28526 0.02016 129.57757 0.02184 121.14991 0.0224 105.01585

7.84E-4 162.9679 0.0012 138.43759 0.00145 141.16557 0.00152 133.51563 0.0015 114.12206 0.01006 125.4218 0.01356 124.92588 0.02034 129.91852 0.02203 121.42826 0.0226 105.229

7.91E-4 162.64274 0.0012 137.23937 0.00146 141.53845 0.00153 133.16628 0.00153 114.12988 0.01015 126.14283 0.01368 125.56461 0.02052 130.25693 0.02223 121.70454 0.0228 105.44085

7.91E-4 163.0763 0.00121 136.71333 0.00149 141.29658 0.00156 132.70047 0.00154 113.39313 0.01023 126.86347 0.0138 126.20144 0.0207 130.59286 0.02243 121.97884 0.023 105.65139

7.98E-4 162.73141 0.00121 135.89503 0.0015 141.28651 0.00157 131.75096 0.00157 113.40095 0.01032 127.58369 0.01392 126.83637 0.02088 130.92636 0.02262 122.25121 0.0232 105.8606

8.05E-4 163.06645 0.00121 135.19365 0.00152 141.11519 0.00159 130.89997 0.00158 112.66418 0.01041 128.30348 0.01404 127.4694 0.02106 131.25747 0.02281 122.52173 0.0234 106.06845

8.12E-4 162.86936 0.00122 134.20973 0.00154 140.88341 0.00161 130.0221 0.00159 112.47606 0.0105 129.02281 0.01416 128.10049 0.02124 131.58623 0.02301 122.79043 0.0236 106.27489

8.12E-4 162.8201 0.00122 133.74214 0.00155 140.22836 0.00162 129.19798 0.00162 111.97443 0.01059 129.74163 0.01428 128.72963 0.02142 131.91266 0.02321 123.05736 0.0238 106.47988

8.19E-4 162.63288 0.00122 132.76799 0.00157 139.08961 0.00164 128.65156 0.00163 111.18279 0.01068 130.4599 0.0144 129.35679 0.0216 132.23678 0.0234 123.32256 0.024 106.68337

8.19E-4 162.42596 0.00122 132.09581 0.00158 139.05938 0.00165 127.66621 0.00165 110.96331 0.01077 131.17758 0.01452 129.98192 0.02178 132.55859 0.0236 123.58604 0.0242 106.88528

8.26E-4 161.5687 0.00123 131.44314 0.0016 137.08418 0.00166 126.89584 0.00166 110.2187 0.01086 131.89461 0.01464 130.60498 0.02196 132.87809 0.02379 123.84783 0.0244 107.08555

8.33E-4 161.12529 0.00123 130.57613 0.00161 136.86249 0.00169 126.27776 0.00169 110.02275 0.01095 132.61094 0.01476 131.22593 0.02214 133.19527 0.02398 124.10793 0.0246 107.28408

8.33E-4 160.27788 0.00123 129.65068 0.00162 135.49194 0.0017 126.00902 0.0017 109.48977 0.01104 133.32649 0.01488 131.84469 0.02232 133.5101 0.02418 124.36634 0.0248 107.4808

8.33E-4 159.31224 0.00123 128.52066 0.00164 134.43381 0.00172 125.8657 0.00171 109.16056 0.01112 134.04119 0.015 132.4612 0.0225 133.82256 0.02438 124.62305 0.025 107.6756

8.4E-4 159.15457 0.00123 127.83876 0.00166 133.59737 0.00173 125.68654 0.00173 108.51785 0.01121 134.75497 0.01512 133.0754 0.02268 134.1326 0.02457 124.87804 0.0252 107.86836

8.4E-4 157.95244 0.00123 126.17294 0.00167 133.35552 0.00174 125.7582 0.00174 108.39243 0.0113 135.46774 0.01524 133.68719 0.02286 134.44017 0.02476 125.1313 0.0254 108.05898

8.47E-4 157.34153 0.00123 125.38387 0.00167 133.15396 0.00176 125.28344 0.00176 107.71053 0.01139 136.17941 0.01536 134.29649 0.02304 134.74522 0.02496 125.38277 0.0256 108.24731

8.47E-4 156.68134 0.00123 123.77651 0.00168 132.54932 0.00177 124.81764 0.00177 107.3343 0.01148 136.88989 0.01548 134.9032 0.02322 135.04765 0.02516 125.63242 0.0258 108.43323

8.47E-4 155.96203 0.00122 122.59778 0.00169 131.73305 0.00178 124.52203 0.00178 106.8797 0.01157 137.59905 0.0156 135.50721 0.0234 135.34741 0.02535 125.88018 0.026 108.61657

8.47E-4 155.39052 0.00122 120.86378 0.0017 131.84388 0.00179 124.18163 0.0018 106.46427 0.01166 138.30679 0.01572 136.1084 0.02358 135.64438 0.02555 126.126 0.0262 108.79717

8.54E-4 154.43473 0.00122 119.16875 0.00172 130.91677 0.00181 123.07984 0.0018 105.71966 0.01175 139.013 0.01584 136.70665 0.02376 135.93846 0.02574 126.36979 0.0264 108.97487

8.54E-4 153.62674 0.00122 117.69777 0.00173 130.41289 0.00181 122.51549 0.00181 105.12396 0.01184 139.71753 0.01596 137.30181 0.02394 136.22952 0.02594 126.61146 0.0266 109.14947

8.54E-4 152.99611 0.00121 116.00272 0.00174 128.7904 0.00182 121.45846 0.00182 104.41854 0.01193 140.42025 0.01608 137.89375 0.02412 136.51745 0.02613 126.85092 0.0268 109.32078

8.54E-4 151.8728 0.00121 114.57071 0.00174 127.99428 0.00183 120.41041 0.00184 103.68176 0.01202 141.12101 0.0162 138.48231 0.0243 136.80209 0.02633 127.08806 0.027 109.48859

8.54E-4 150.72979 0.00121 112.95361 0.00175 126.40204 0.00185 119.21007 0.00185 102.89796 0.0121 141.81965 0.01632 139.06731 0.02448 137.08328 0.02652 127.32276 0.0272 109.65266

8.54E-4 149.30103 0.0012 111.56057 0.00176 125.54546 0.00185 117.51705 0.00185 102.16119 0.01219 142.51601 0.01644 139.64857 0.02466 137.36085 0.02671 127.55487 0.0274 109.81277

8.54E-4 148.03978 0.0012 110.00192 0.00178 124.8098 0.00186 116.41525 0.00186 101.28332 0.01228 143.20991 0.01656 140.22589 0.02484 137.63461 0.02691 127.78425 0.0276 109.96864

8.54E-4 146.98543 0.00119 108.43353 0.00178 123.88267 0.00186 115.23282 0.00188 100.38979 0.01237 143.90115 0.01668 140.79907 0.02502 137.90434 0.02711 128.01074 0.0278 110.12003

8.54E-4 145.50742 0.00119 107.04048 0.00179 122.91523 0.00187 113.89811 0.00188 99.44138 0.01246 144.58954 0.0168 141.36789 0.0252 138.16984 0.0273 128.23415 0.028 110.26662

8.54E-4 144.44323 0.00118 105.3357 0.00179 121.83693 0.00187 113.30689 0.00189 98.48514 0.01255 145.27486 0.01692 141.9321 0.02538 138.43085 0.02749 128.45431 0.0282 110.40813

8.54E-4 142.37398 0.00117 103.73809 0.0018 120.70826 0.00189 112.06176 0.00189 97.48187 0.01264 145.95689 0.01704 142.49145 0.02556 138.68713 0.02769 128.67099 0.0284 110.54422

8.54E-4 141.49702 0.00117 101.78977 0.0018 119.48888 0.00189 111.21078 0.0019 96.43941 0.01273 146.63538 0.01716 143.04567 0.02574 138.93838 0.02789 128.88397 0.0286 110.67455

8.54E-4 140.08796 0.00116 100.15318 0.00181 118.39044 0.00189 110.36874 0.0019 95.56154 0.01282 147.31007 0.01728 143.59445 0.02592 139.18431 0.02808 129.09301 0.0288 110.79876

8.47E-4 138.36359 0.00115 98.11719 0.00181 116.68734 0.00189 109.21319 0.00192 94.80125 0.01291 147.98068 0.0174 144.1375 0.0261 139.42459 0.02828 129.29785 0.029 110.91645

8.47E-4 136.92496 0.00114 96.64621 0.00181 115.80052 0.0019 108.05764 0.00192 93.74311 0.01299 148.64694 0.01752 144.67448 0.02628 139.65889 0.02847 129.49819 0.0292 111.02721

8.47E-4 135.93961 0.00113 94.73687 0.00182 114.44006 0.0019 107.22457 0.00192 92.86525 0.01308 149.30852 0.01764 145.20504 0.02646 139.88682 0.02867 129.69375 0.0294 111.13061

8.47E-4 134.28422 0.00112 92.96389 0.00182 113.2106 0.0019 105.80027 0.00193 91.77576 0.01317 149.9651 0.01776 145.72878 0.02664 140.10798 0.02886 129.88417 0.0296 111.22617

8.4E-4 132.73721 0.00111 91.66826 0.00182 112.17262 0.0019 104.88658 0.00193 90.65491 0.01326 150.61631 0.01788 146.24531 0.02682 140.32196 0.02906 130.06912 0.0298 111.31341

8.4E-4 130.08659 0.0011 90.3629 0.00182 111.27572 0.0019 103.29211 0.00193 89.39299 0.01335 151.26179 0.018 146.75418 0.027 140.52827 0.02925 130.24821 0.03 111.39179

8.33E-4 128.49032 0.00109 89.40822 0.00182 109.82456 0.0019 101.87677 0.00193 88.47594 0.01344 151.90112 0.01812 147.25494 0.02718 140.72644 0.02944 130.42103 0.0302 111.46076

8.33E-4 127.31774 0.00108 88.33665 0.00184 109.3207 0.0019 100.39874 0.00193 86.924 0.01353 152.53389 0.01824 147.74709 0.02736 140.91593 0.02964 130.58714 0.0304 111.51971

8.33E-4 126.01707 0.00107 87.08972 0.00184 108.24239 0.0019 99.3238 0.00193 85.83452 0.01362 153.15962 0.01836 148.23008 0.02754 141.09617 0.02984 130.74607 0.0306 111.56801

8.26E-4 124.75582 0.00106 85.52133 0.00184 107.11371 0.0019 98.00701 0.00193 84.42367 0.01371 153.77782 0.01848 148.70333 0.02772 141.26655 0.03003 130.89731 0.0308 111.60499

8.19E-4 123.25808 0.00105 83.81656 0.00184 105.74317 0.0019 96.92312 0.00193 83.13038 0.0138 154.38796 0.0186 149.16625 0.0279 141.4264 0.03022 131.04031 0.031 111.6299

8.19E-4 121.72092 0.00104 82.47222 0.00184 104.58426 0.0019 95.35551 0.00193 82.05658 0.01388 154.98947 0.01872 149.61815 0.02808 141.57503 0.03042 131.17447 0.0312 111.64199

8.12E-4 120.21333 0.00103 81.45909 0.00182 103.16333 0.0019 94.28058 0.00192 80.81816 0.01397 155.58175 0.01884 150.05832 0.02826 141.71167 0.03062 131.29917 0.0314 111.64043

8.12E-4 119.07032 0.00102 80.61157 0.00182 101.76255 0.00189 92.60547 0.00192 79.50137 0.01406 156.16411 0.01896 150.486 0.02844 141.8355 0.03081 131.4137 0.0316 111.62432

8.05E-4 116.77444 1E-3 79.61793 0.00182 99.96876 0.00189 91.20806 0.00192 78.23944 0.01415 156.73587 0.01908 150.90036 0.02862 141.94563 0.03101 131.51734 0.0318 111.59271

7.98E-4 115.43436 9.90105E-4 78.5561 0.00182 98.53776 0.00189 90.11522 0.0019 76.91481 0.01424 157.29625 0.0192 151.30048 0.0288 142.04111 0.0312 131.60928 0.032 111.54459

7.91E-4 114.25194 9.77119E-4 78.04954 0.00182 97.06645 0.00187 88.60135 0.0019 75.76262 0.01433 157.84441 0.01932 151.68541 0.02898 142.1209 0.03139 131.68865 0.0322 111.47887

7.91E-4 112.41917 9.63837E-4 77.09486 0.00181 95.73621 0.00187 87.5085 0.00189 74.79854 0.01442 158.37946 0.01944 152.0541 0.02916 142.1839 0.03159 131.75451 0.0324 111.39437

7.84E-4 110.94114 9.50346E-4 76.97797 0.00181 93.93234 0.00186 86.17379 0.00189 73.5131 0.01451 158.90043 0.01956 152.4054 0.02934 142.22888 0.03179 131.80584 0.0326 111.28981

7.77E-4 109.27589 9.36696E-4 76.00381 0.00181 92.36026 0.00185 85.17052 0.00188 72.49415 0.0146 159.40624 0.01968 152.73807 0.02952 142.25454 0.03198 131.84154 0.0328 111.16384

7.7E-4 107.3643 9.22951E-4 75.57518 0.0018 90.89902 0.00185 83.7731 0.00188 71.29493 0.01469 159.89576 0.0198 153.05077 0.0297 142.25945 0.03218 131.8604 0.033 111.01499

7.63E-4 105.84686 9.09021E-4 75.10758 0.0018 89.80057 0.00183 82.12488 0.00186 70.50329 0.01477 160.36771 0.01992 153.34203 0.02988 142.24207 0.03237 131.86112 0.0332 110.84166

7.56E-4 104.48706 8.94906E-4 74.06523 0.00179 88.29903 0.00182 80.85287 0.00185 69.43731 0.01486 160.82072 0.02004 153.61025 0.03006 142.2007 0.03256 131.84226 0.0334 110.64212

7.49E-4 102.95976 8.80606E-4 73.76325 0.00179 87.07964 0.00182 79.59878 0.00184 68.27728 0.01495 161.25329 0.02016 153.85366 0.03024 142.1335 0.03276 131.80227 0.0336 110.41452

7.42E-4 101.26495 8.66052E-4 73.13978 0.00178 85.55794 0.00181 78.81946 0.00184 67.66591 0.01504 161.66375 0.02028 154.07036 0.03042 142.03845 0.03295 131.73942 0.0338 110.15682

7.35E-4 99.66868 8.51345E-4 72.10717 0.00178 83.70368 0.00179 77.63703 0.00182 66.78805 0.01513 162.05029 0.0204 154.25822 0.0306 141.91336 0.03315 131.65186 0.034 109.86681

7.28E-4 98.15123 8.36537E-4 71.17199 0.00176 82.07113 0.00178 76.64272 0.00181 65.82397 0.01522 162.41089 0.02052 154.41492 0.03078 141.7558 0.03335 131.53752 0.0342 109.5421

7.14E-4 96.87027 8.21682E-4 70.42188 0.00175 80.45873 0.00177 75.63049 0.0018 65.18125 0.01531 162.74334 0.02064 154.5379 0.03096 141.5631 0.03354 131.39412 0.0344 109.18006

7.07E-4 95.59916 8.06804E-4 69.58411 0.00175 79.35021 0.00176 74.77054 0.00178 64.38177 0.0154 163.04516 0.02076 154.62431 0.03114 141.33233 0.03374 131.21917 0.0346 108.77783

7E-4 94.20981 7.91836E-4 68.26899 0.00174 78.20137 0.00174 73.53437 0.00177 63.96635 0.01549 163.31363 0.02088 154.671 0.03132 141.06025 0.03393 131.00989 0.0348 108.33226

6.91895E-4 92.7909 7.76719E-4 67.2169 0.00173 77.35487 0.00173 72.38778 0.00176 63.20606 0.01558 163.54569 0.021 154.67449 0.0315 140.74326 0.03413 130.76321 0.035 107.83991

6.83082E-4 91.80554 7.61469E-4 65.45368 0.00172 75.95409 0.00172 71.37554 0.00174 62.5555 0.01566 163.73793 0.02112 154.63086 0.03168 140.37739 0.03432 130.4757 0.0352 107.29698

6.74094E-4 90.38663 7.46106E-4 64.20675 0.0017 75.13781 0.0017 70.856 0.00173 62.30468 0.01575 163.8865 0.02124 154.53575 0.03186 139.95819 0.03451 130.14356 0.0354 106.69931

6.64838E-4 88.14003 7.30675E-4 62.57017 0.0017 73.81766 0.00169 70.09458 0.0017 61.56007 0.01584 163.98711 0.02136 154.38427 0.03204 139.48071 0.03471 129.76252 0.0356 106.04227

6.55271E-4 86.32697 7.15137E-4 61.06022 0.00169 72.46728 0.00166 69.55712 0.00169 60.98788 0.01593 164.03486 0.02148 154.17088 0.03222 138.9394 0.0349 129.32779 0.0358 105.32076

6.45484E-4 84.10007 6.99529E-4 59.73537 0.00168 70.73394 0.00165 67.89993 0.00167 60.49409 0.01602 164.02424 0.0216 153.88938 0.0324 138.32805 0.0351 128.83398 0.036 104.52911

6.35596E-4 82.61218 6.83843E-4 58.78069 0.00167 69.61534 0.00164 67.10269 0.00166 60.03164 0.01611 163.94893 0.02172 153.53268 0.03258 137.63962 0.0353 128.275 0.0362 103.66103

6.25627E-4 81.22283 6.68069E-4 57.13436 0.00166 68.39597 0.00162 66.11734 0.00163 59.34974 0.0162 163.80172 0.02184 153.09275 0.03276 136.86619 0.03549 127.64395 0.0364 102.70948

6.15572E-4 79.51816 6.52262E-4 55.84848 0.00163 67.44868 0.00161 65.20364 0.00162 58.88729 0.01629 163.57432 0.02196 152.56038 0.03294 135.99875 0.03569 126.93297 0.0366 101.66663

6.05382E-4 78.34559 6.36281E-4 54.15345 0.00162 66.62233 0.00159 64.58555 0.00161 58.11133 0.01638 163.25716 0.02208 151.92502 0.03312 135.02705 0.03588 126.13308 0.0368 100.52367

5.95099E-4 76.76903 6.20134E-4 52.23435 0.00161 65.48357 0.00157 64.11079 0.00158 56.86507 0.01647 162.8391 0.0222 151.17447 0.0333 133.93935 0.03608 125.23397 0.037 99.27074

5.84807E-4 75.54718 6.03918E-4 50.54905 0.0016 64.82853 0.00156 63.27772 0.00157 55.61882 0.01655 162.30714 0.02232 150.29459 0.03348 132.7222 0.03627 124.22375 0.0372 97.89674

5.74486E-4 74.16768 5.87631E-4 48.68842 0.00158 63.87117 0.00153 62.50735 0.00154 54.4196 0.01664 161.64601 0.02244 149.26888 0.03366 131.36004 0.03646 123.08866 0.0374 96.38911

5.64101E-4 72.29551 5.7137E-4 47.05183 0.00157 63.32699 0.00152 62.10425 0.00153 53.65931 0.01673 160.8376 0.02256 148.078 0.03384 129.83488 0.03666 121.81265 0.0376 94.7337

5.53624E-4 71.07367 5.55258E-4 45.33731 0.00155 62.32931 0.0015 61.13681 0.00151 52.76577 0.01682 159.86038 0.02268 146.69911 0.03402 128.12572 0.03685 120.37692 0.0378 92.91445

5.43115E-4 69.32959 5.39232E-4 43.56435 0.00154 61.75489 0.00148 60.91286 0.00149 51.87223 0.01691 158.68843 0.0228 145.10507 0.0342 126.20804 0.03705 118.75936 0.038 90.91315

5.32582E-4 67.86141 5.2322E-4 41.81086 0.00152 61.04948 0.00147 59.57816 0.00147 50.64166 0.017 157.29039 0.02292 143.26341 0.03438 124.05293 0.03725 116.93376 0.0382 88.70916

5.21982E-4 65.90055 5.07235E-4 40.23272 0.00151 60.22311 0.00144 58.89737 0.00144 49.62271 0.01709 155.62788 0.02304 141.13496 0.03456 121.62625 0.03744 114.86886 0.0384 86.27901

5.11391E-4 64.85608 4.91359E-4 38.62536 0.00149 60.0518 0.00143 58.32407 0.00142 48.47835 0.01718 153.65352 0.02316 138.67213 0.03474 118.8874 0.03764 112.52718 0.0386 83.59614

5.00767E-4 63.28936 -- 36.81343 0.00148 58.94328 0.0014 56.65792 0.0014 47.45157 0.01727 151.3081 0.02328 135.81654 0.03492 115.78787 0.03783 109.86345 0.0388 80.63057

4.90078E-4 61.92958 -- 42.44563 0.00146 58.68126 0.00139 55.76214 0.00138 46.31505 0.01735 148.51681 0.0234 132.49602 0.0351 112.26947 0.03803 106.82268 0.039 77.34873

4.79375E-4 60.53037 -- 42.37712 0.00144 58.38901 0.00136 54.91116 0.00136 45.2334 0.01744 145.18383 0.02352 128.62046 0.03528 108.26221 0.03822 103.33767 0.0392 73.71352

4.68663E-4 58.79614 -- 42.36417 0.00143 57.35103 0.00134 53.77352 0.00134 44.47311 0.01753 141.18467 0.02364 124.07637 0.03546 103.68179 0.03841 99.32598 0.0394 69.68483

4.57889E-4 57.21958 -- 42.39417 0.0014 56.66576 0.00133 52.46569 0.00132 43.67363 0.01762 136.3551 0.02376 118.71969 0.03564 98.42706 0.03861 94.6863 0.0396 65.22111

4.47058E-4 55.84993 -- 42.45236 0.00139 55.75879 0.0013 51.39971 0.00129 43.0701 0.01771 130.47503 0.02388 112.36645 0.03582 92.37802 0.0388 89.29458 0.0398 60.28243

4.36216E-4 54.23395 -- 42.52088 0.00137 54.76111 0.00128 50.07396 0.00127 42.25494

4.25418E-4 52.83474 -- 42.5795 0.00136 53.89445 0.00126 48.63176 0.00125 41.55736

4.14685E-4 51.49466 -- 42.6056 0.00133 53.22934 0.00124 47.73598 0.00123 40.72653

4.03953E-4 50.07575 -- 42.57634 0.00132 51.86887 0.00121 46.11462 0.00121 39.69974

3.93228E-4 48.74552 -- 42.47036 0.0013 51.35492 0.00119 44.90532 0.00118 39.21378

3.82535E-4 47.68134 -- 42.27093 0.00128 49.74252 0.00117 43.51687 0.00116 38.37511

3.71905E-4 46.43979 -- 41.96689 0.00126 49.01694 0.00115 42.53151 0.00114 37.69319

3.61376E-4 45.16868 -- 41.55435 0.00125 48.25105

3.50946E-4 44.06508 -- 41.03626 0.00122 47.44989

3.40566E-4 42.44911 -- 40.42081 0.00121 46.47741

3.30229E-4 41.40462 -- 39.72033 0.00119 45.87277

3.19979E-4 39.96601 -- 38.94902 0.00117 45.17742

3.0972E-4 38.68505 -- 38.12194 0.00115 44.23013

2.99416E-4 37.5913 -- 37.25382 0.00113 43.46424

2.89156E-4 36.05415 -- 36.36001 0.00112 42.35572

2.78935E-4 34.98996 -- 35.45526 0.0011 40.8844

2.68703E-4 33.69915 -- 34.55609 0.00108 39.99758

2.58484E-4 33.1572 -- 33.67943 -- --

2.48252E-4 32.20141 -- 32.84214 -- --

2.37993E-4 31.37371 -- 32.0601 -- --

2.27753E-4 30.39821 -- 31.34563 -- --

2.17515E-4 29.403 -- 30.70555 -- --

2.07236E-4 28.68369 -- 30.1401 -- --

1.97001E-4 27.79687 -- 29.64284 -- --

1.96359E-4 27.03814 -- 29.20094 -- --

**Fig. 15 Variation of fitting parameters of different cracked rock masses with impact times**

**(a) *k***

1 0.0061 1 0.012 1 0.015 1 0.009 1 0.0042

3 0.006 3 0.01 2 0.011 3 0.008 3 0.0048

5 0.0058 5 0.008 3 0.009 5 0.007 5 0.005

7 0.0056 7 0.0075 4 0.0085 7 0.0065 7 0.005

8 0.0054 5 0.0073 8 0.0061 9 0.0038

**(b) *m***

1 1.5 1 1.2 1 1.2 1 1.4 1 1.38

3 1.7 3 1.4 2 0.9 3 1.3 3 1.42

5 1.9 5 2 3 0.8 5 1.45 5 1.48

7 2.2 7 2.8 4 0.7 7 1.49 7 1.72

8 2.4 5 0.72 8 1.42 9 2.1

**(c) *η***

1 0.029 1 0.018 1 0.01 1 0.019 1 0.025

3 0.018 3 0.015 2 0.008 3 0.018 3 0.018

5 0.012 5 0.014 3 0.01 5 0.018 5 0.012

7 0.007 7 0.008 4 0.0065 7 0.012 7 0.012

8 0.004 5 0.005 8 0.012 9 0.007

**(d) *E*_M_**

1 12 1 10.5 1 9 1 10.9 1 13.2

3 11.3 3 10.1 2 8 3 10.5 3 13

5 10.6 5 9.2 3 8 5 10.5 5 12.4

7 9 7 8.8 4 7.2 7 10 7 12.2

8 8.6 5 6.8 8 9.6 9 11

**Fig. 16 Effect of strain rate on stress-strain relationship of rock masses**

8.755E-5 1.41388 8.755E-5 1.42319 8.755E-5 1.44829 8.755E-5 1.46774

1.751E-4 2.72322 1.751E-4 2.84305 1.751E-4 2.89334 1.751E-4 2.93226

2.6265E-4 4.07899 2.6265E-4 4.25909 2.6265E-4 4.33464 2.6265E-4 4.39307

3.502E-4 5.43039 3.502E-4 5.67101 3.502E-4 5.7719 3.502E-4 5.84984

4.3775E-4 6.77721 4.3775E-4 7.07857 4.3775E-4 7.20487 4.3775E-4 7.30237

5.253E-4 8.11925 5.253E-4 8.48159 5.253E-4 8.63339 5.253E-4 8.75044

6.1285E-4 9.45636 6.1285E-4 9.87993 6.1285E-4 10.05728 6.1285E-4 10.19393

7.004E-4 10.78842 7.004E-4 11.27343 7.004E-4 11.47645 7.004E-4 11.63269

7.8795E-4 12.1153 7.8795E-4 12.66202 7.8795E-4 12.89074 7.8795E-4 13.06661

8.755E-4 13.43689 8.755E-4 14.04556 8.755E-4 14.30009 8.755E-4 14.49559

9.6305E-4 14.75311 9.6305E-4 15.42396 9.6305E-4 15.70439 9.6305E-4 15.91956

0.00105 16.06387 0.00105 16.79717 0.00105 17.10355 0.00105 17.3384

0.00114 17.36909 0.00114 18.16509 0.00114 18.49752 0.00114 18.75208

0.00123 18.6687 0.00123 19.52766 0.00123 19.88621 0.00123 20.16049

0.00132 19.96262 0.00132 20.88482 0.00132 21.26958 0.00132 21.5636

0.0014 21.25081 0.0014 22.23649 0.0014 22.64757 0.0014 22.96135

0.00149 22.53321 0.00149 23.58266 0.00149 24.02011 0.00149 24.3537

0.00158 23.80976 0.00158 24.92324 0.00158 25.38718 0.00158 25.74057

0.00167 25.0804 0.00167 26.25822 0.00167 26.74872 0.00167 27.12195

0.00175 26.34511 0.00175 27.58752 0.00175 28.10469 0.00175 28.49778

0.00184 27.60382 0.00184 28.91113 0.00184 29.45506 0.00184 29.86804

0.00193 28.8565 0.00193 30.22901 0.00193 30.7998 0.00193 31.23269

0.00202 30.10311 0.00202 31.54113 0.00202 32.13887 0.00202 32.5917

0.0021 31.34361 0.0021 32.84744 0.0021 33.47223 0.0021 33.94504

0.00219 32.57798 0.00219 34.14793 0.00219 34.79989 0.00219 35.29268

0.00228 33.80619 0.00228 35.44257 0.00228 36.1218 0.00228 36.63463

0.00237 35.02818 0.00237 36.73133 0.00237 37.43795 0.00237 37.97084

0.00245 36.24395 0.00245 38.01421 0.00245 38.7483 0.00245 39.30129

0.00254 37.45347 0.00254 39.29116 0.00254 40.05286 0.00254 40.62598

0.00263 38.65669 0.00263 40.56218 0.00263 41.3516 0.00263 41.94488

0.00272 39.85363 0.00272 41.82725 0.00272 42.6445 0.00272 43.25799

0.0028 41.04424 0.0028 43.08635 0.0028 43.93157 0.0028 44.56529

0.00289 42.22849 0.00289 44.33948 0.00289 45.21277 0.00289 45.86677

0.00298 43.40638 0.00298 45.58661 0.00298 46.48812 0.00298 47.16245

0.00307 44.5779 0.00307 46.82775 0.00307 47.75759 0.00307 48.45229

0.00315 45.743 0.00315 48.06287 0.00315 49.02119 0.00315 49.73629

0.00324 46.90169 0.00324 49.29197 0.00324 50.2789 0.00324 51.01446

0.00333 48.05394 0.00333 50.51504 0.00333 51.53073 0.00333 52.28679

0.00342 49.19976 0.00342 51.73209 0.00342 52.77667 0.00342 53.55328

0.0035 50.33911 0.0035 52.9431 0.0035 54.01672 0.0035 54.81394

0.00359 51.47199 0.00359 54.14807 0.00359 55.25088 0.00359 56.06876

0.00368 52.59839 0.00368 55.34699 0.00368 56.47915 0.00368 57.31775

0.00377 53.71829 0.00377 56.53987 0.00377 57.70154 0.00377 58.56091

0.00385 54.83169 0.00385 57.72671 0.00385 58.91804 0.00385 59.79825

0.00394 55.93858 0.00394 58.90751 0.00394 60.12867 0.00394 61.02978

0.00403 57.03894 0.00403 60.08227 0.00403 61.33342 0.00403 62.25548

0.00412 58.13278 0.00412 61.25098 0.00412 62.5323 0.00412 63.4754

0.0042 59.22007 0.0042 62.41366 0.0042 63.72534 0.0042 64.68952

0.0043 60.30083 0.0043 63.5703 0.0043 64.91251 0.0043 65.89787

0.00438 61.37503 0.00438 64.72092 0.00438 66.09385 0.00438 67.10044

0.00447 62.44268 0.00447 65.86551 0.00447 67.26936 0.00447 68.29726

0.00455 63.50376 0.00455 67.00408 0.00455 68.43903 0.00455 69.48834

0.00465 64.55827 0.00465 68.13666 0.00465 69.6029 0.00465 70.67368

0.00473 65.60623 0.00473 69.26322 0.00473 70.76098 0.00473 71.85331

0.00482 66.64759 0.00482 70.38379 0.00482 71.91327 0.00482 73.02725

0.0049 67.68238 0.0049 71.49837 0.0049 73.05979 0.0049 74.1955

0.005 68.71058 0.005 72.60698 0.005 74.20055 0.005 75.35809

0.00508 69.73219 0.00508 73.70961 0.00508 75.33556 0.00508 76.51504

0.00516 70.74721 0.00516 74.80629 0.00516 76.46486 0.00516 77.66635

0.00525 71.75563 0.00525 75.89702 0.00525 77.58844 0.00525 78.81206

0.00534 72.75746 0.00534 76.9818 0.00534 78.70633 0.00534 79.95218

0.00543 73.75268 0.00543 78.06067 0.00543 79.81854 0.00543 81.08672

0.00551 74.7413 0.00551 79.13363 0.00551 80.9251 0.00551 82.21572

0.0056 75.7233 0.0056 80.20067 0.0056 82.02601 0.0056 83.3392

0.00569 76.69869 0.00569 81.26183 0.00569 83.1213 0.00569 84.45718

0.00578 77.66746 0.00578 82.31711 0.00578 84.21099 0.00578 85.56968

0.00586 78.62961 0.00586 83.36654 0.00586 85.29511 0.00586 86.67672

0.00595 79.58514 0.00595 84.4101 0.00595 86.37365 0.00595 87.77833

0.00604 80.53402 0.00604 85.44782 0.00604 87.44666 0.00604 88.87453

0.00613 81.47628 0.00613 86.47973 0.00613 88.51414 0.00613 89.96535

0.00621 82.41191 0.00621 87.50582 0.00621 89.57613 0.00621 91.05082

0.0063 83.34088 0.0063 88.52611 0.0063 90.63263 0.0063 92.13095

0.00639 84.2632 0.00639 89.54062 0.00639 91.68368 0.00639 93.20579

0.00648 85.17887 0.00648 90.54936 0.00648 92.7293 0.00648 94.27534

0.00656 86.08787 0.00656 91.55234 0.00656 93.76951 0.00656 95.33965

0.00665 86.9902 0.00665 92.54958 0.00665 94.80432 0.00665 96.39873

0.00674 87.88585 0.00674 93.54108 0.00674 95.83377 0.00674 97.45262

0.00683 88.77481 0.00683 94.52687 0.00683 96.85788 0.00683 98.50134

0.00691 89.65707 0.00691 95.50696 0.00691 97.87666 0.00691 99.54493

0.007 90.53263 0.007 96.48136 0.007 98.89016 0.007 100.5834

0.00709 91.40146 0.00709 97.45009 0.00709 99.89838 0.00709 101.6168

0.00718 92.26356 0.00718 98.41315 0.00718 100.90134 0.00718 102.64515

0.00726 93.11892 0.00726 99.37056 0.00726 101.89908 0.00726 103.66847

0.00735 93.96752 0.00735 100.32233 0.00735 102.89162 0.00735 104.6868

0.00744 94.80935 0.00744 101.26847 0.00744 103.87898 0.00744 105.70018

0.00753 95.6444 0.00753 102.209 0.00753 104.86117 0.00753 106.70862

0.00761 96.47262 0.00761 103.14392 0.00761 105.83824 0.00761 107.71217

0.0077 97.29403 0.0077 104.07326 0.0077 106.8102 0.0077 108.71083

0.00779 98.10859 0.00779 104.997 0.00779 107.77707 0.00779 109.70466

0.00788 98.91627 0.00788 105.91517 0.00788 108.73887 0.00788 110.69369

0.00796 99.71707 0.00796 106.82778 0.00796 109.69563 0.00796 111.67792

0.00805 100.51095 0.00805 107.73482 0.00805 110.64738 0.00805 112.65742

0.00814 101.29788 0.00814 108.63632 0.00814 111.59413 0.00814 113.63219

0.00823 102.07784 0.00823 109.53227 0.00823 112.5359 0.00823 114.60227

0.00831 102.8508 0.00831 110.42268 0.00831 113.47272 0.00831 115.5677

0.0084 103.61672 0.0084 111.30756 0.0084 114.40461 0.0084 116.5285

0.00849 104.37558 0.00849 112.18691 0.00849 115.33158 0.00849 117.4847

0.00858 105.12732 0.00858 113.06074 0.00858 116.25365 0.00858 118.43634

0.00866 105.87192 0.00866 113.92903 0.00866 117.17086 0.00866 119.38344

0.00876 106.60932 0.00876 114.79179 0.00876 118.08321 0.00876 120.32603

0.00885 107.33951 0.00885 115.64903 0.00885 118.99074 0.00885 121.26414

0.00893 108.0624 0.00893 116.50074 0.00893 119.89344 0.00893 122.1978

0.00902 108.77796 0.00902 117.34692 0.00902 120.79134 0.00902 123.12704

0.00911 109.48614 0.00911 118.18756 0.00911 121.68446 0.00911 124.05189

0.0092 110.18687 0.0092 119.02265 0.0092 122.57281 0.0092 124.97237

0.00928 110.88012 0.00928 119.85219 0.00928 123.45641 0.00928 125.88853

0.00937 111.5658 0.00937 120.67616 0.00937 124.33526 0.00937 126.80036

0.00946 112.24383 0.00946 121.49456 0.00946 125.20939 0.00946 127.70792

0.00955 112.91419 0.00955 122.30738 0.00955 126.0788 0.00955 128.61123

0.00963 113.57676 0.00963 123.11457 0.00963 126.94351 0.00963 129.5103

0.00972 114.23146 0.00972 123.91614 0.00972 127.80353 0.00972 130.40517

0.00981 114.87824 0.00981 124.71207 0.00981 128.65884 0.00981 131.29586

0.0099 115.517 0.0099 125.50233 0.0099 129.5095 0.0099 132.18239

0.00998 116.14763 0.00998 126.2869 0.00998 130.35546 0.00998 133.06479

0.01007 116.77004 0.01007 127.06573 0.01007 131.19676 0.01007 133.94308

0.01016 117.38413 0.01016 127.83881 0.01016 132.03338 0.01016 134.81728

0.01025 117.98979 0.01025 128.60609 0.01025 132.86534 0.01025 135.68742

0.01033 118.58691 0.01033 129.36755 0.01033 133.69263 0.01033 136.5535

0.01042 119.17535 0.01042 130.12314 0.01042 134.51523 0.01042 137.41555

0.01051 119.75499 0.01051 130.8728 0.01051 135.33317 0.01051 138.27359

0.0106 120.3257 0.0106 131.61651 0.0106 136.14641 0.0106 139.12763

0.01068 120.88733 0.01068 132.35417 0.01068 136.95495 0.01068 139.97769

0.01077 121.43973 0.01077 133.08578 0.01077 137.7588 0.01077 140.82379

0.01086 121.98275 0.01086 133.81124 0.01086 138.5579 0.01086 141.66593

0.01095 122.51622 0.01095 134.53049 0.01095 139.35227 0.01095 142.50413

0.01103 123.03995 0.01103 135.24345 0.01103 140.14187 0.01103 143.33838

0.01112 123.55375 0.01112 135.95006 0.01112 140.92669 0.01112 144.16871

0.01121 124.05744 0.01121 136.65022 0.01121 141.70669 0.01121 144.99512

0.0113 124.55081 0.0113 137.34386 0.0113 142.48185 0.0113 145.81762

0.01138 125.03363 0.01138 138.03086 0.01138 143.25212 0.01138 146.63618

0.01147 125.50568 0.01147 138.71113 0.01147 144.01748 0.01147 147.45084

0.01156 125.9667 0.01156 139.38455 0.01156 144.77788 0.01156 148.26158

0.01165 126.41645 0.01165 140.05101 0.01165 145.53326 0.01165 149.06839

0.01173 126.85466 0.01173 140.71036 0.01173 146.28358 0.01173 149.87127

0.01182 127.28101 0.01182 141.36249 0.01182 147.02879 0.01182 150.67021

0.01191 127.69524 0.01191 142.00725 0.01191 147.76882 0.01191 151.46518

0.012 128.097 0.012 142.64448 0.012 148.50359 0.012 152.25618

0.01208 128.48596 0.01208 143.27401 0.01208 149.23303 0.01208 153.04319

0.01217 128.86176 0.01217 143.89564 0.01217 149.95707 0.01217 153.82617

0.01226 129.22403 0.01226 144.50921 0.01226 150.67562 0.01226 154.60512

0.01235 129.57236 0.01235 145.11451 0.01235 151.38856 0.01235 155.37998

0.01243 129.90633 0.01243 145.71131 0.01243 152.09581 0.01243 156.15072

0.01252 130.22548 0.01252 146.29937 0.01252 152.79723 0.01252 156.91729

0.01261 130.52936 0.01261 146.87845 0.01261 153.49272 0.01261 157.67966

0.0127 130.81745 0.0127 147.44827 0.0127 154.18213 0.0127 158.43778

0.01278 131.08921 0.01278 148.00855 0.01278 154.86533 0.01278 159.19157

0.01287 131.34409 0.01287 148.55897 0.01287 155.54214 0.01287 159.94096

0.01296 131.58148 0.01296 149.09921 0.01296 156.2124 0.01296 160.68591

0.01305 131.80075 0.01305 149.62891 0.01305 156.87592 0.01305 161.42631

0.01313 132.00121 0.01313 150.14768 0.01313 157.5325 0.01313 162.16208

0.01323 132.18215 0.01323 150.65511 0.01323 158.18193 0.01323 162.89311

0.01331 132.34281 0.01331 151.15076 0.01331 158.82397 0.01331 163.61931

0.0134 132.48235 0.0134 151.63416 0.0134 159.45835 0.0134 164.34054

0.01348 132.59993 0.01348 152.10479 0.01348 160.0848 0.01348 165.05668

0.01358 132.69461 0.01358 152.56211 0.01358 160.70302 0.01358 165.76758

0.01366 132.76541 0.01366 153.00551 0.01366 161.31268 0.01366 166.47307

0.01375 132.81129 0.01375 153.43435 0.01375 161.91343 0.01375 167.17301

0.01383 132.83109 0.01383 153.84795 0.01383 162.50487 0.01383 167.86716

0.01393 132.82364 0.01393 154.24555 0.01393 163.08658 0.01393 168.55533

0.01401 132.78763 0.01401 154.62634 0.01401 163.65809 0.01401 169.23729

0.0141 132.72171 0.0141 154.98945 0.0141 164.21892 0.0141 169.91278

0.01418 132.62438 0.01418 155.33391 0.01418 164.76852 0.01418 170.5815

0.01428 132.49406 0.01428 155.65869 0.01428 165.30627 0.01428 171.24316

0.01436 132.32905 0.01436 155.96264 0.01436 165.83153 0.01436 171.89741

0.01445 132.12753 0.01445 156.24457 0.01445 166.34357 0.01445 172.54387

0.01453 131.88752 0.01453 156.50309 0.01453 166.84159 0.01453 173.18212

0.01463 131.60691 0.01463 156.73676 0.01463 167.32473 0.01463 173.81171

0.01471 131.28339 0.01471 156.94395 0.01471 167.79203 0.01471 174.43208

0.0148 130.91451 0.0148 157.1229 0.0148 168.24242 0.0148 175.04271

0.01488 130.49761 0.01488 157.27166 0.01488 168.67473 0.01488 175.64293

0.01498 130.02978 0.01498 157.3881 0.01498 169.08766 0.01498 176.23203

0.01506 129.50791 0.01506 157.46983 0.01506 169.47975 0.01506 176.80923

0.01515 128.9286 0.01515 157.51425 0.01515 169.84939 0.01515 177.37363

0.01523 128.28815 0.01523 157.51845 0.01523 170.19477 0.01523 177.92424

0.01533 127.58256 0.01533 157.47919 0.01533 170.51386 0.01533 178.45992

0.01541 126.80748 0.01541 157.39286 0.01541 170.80439 0.01541 178.97941

0.0155 125.95814 0.0155 157.25544 0.0155 171.06379 0.0155 179.48125

0.01558 125.02935 0.01558 157.06241 0.01558 171.28914 0.01558 179.96381

0.01568 124.01544 0.01568 156.80866 0.01568 171.47716 0.01568 180.42519

0.01576 122.91021 0.01576 156.48845 0.01576 171.62406 0.01576 180.86326

0.01585 121.70686 0.01585 156.09528 0.01585 171.72554 0.01585 181.27553

0.01593 120.39796 0.01593 155.62172 0.01593 171.77661 0.01593 181.65913

0.01603 118.97533 0.01603 155.05932 0.01603 171.77154 0.01603 182.01069

0.01611 117.43004 0.01611 154.39837 0.01611 171.70365 0.01611 182.32632

0.0162 115.75224 0.0162 153.62769 0.0162 171.56512 0.0162 182.60138

0.01628 113.93117 0.01628 152.73435 0.01628 171.34678 0.01628 182.83041

0.01638 111.95503 0.01638 151.70325 0.01638 171.03774 0.01638 183.00688

0.01646 109.81088 0.01646 150.5168 0.01646 170.62504 0.01646 183.1229

0.01655 107.48463 0.01655 149.15424 0.01655 170.09307 0.01655 183.16891

0.01663 104.96096 0.01663 147.59101 0.01663 169.4229 0.01663 183.13318

0.01673 102.22336 0.01673 145.79781 0.01673 168.59136 0.01673 183.00114

0.01681 99.25414 0.01681 143.7395 0.01681 167.56979 0.01681 182.75449

0.0169 96.03468 0.0169 141.37357 0.0169 166.32227 0.0169 182.36998

0.01698 92.5458 0.01698 138.64819 0.01698 164.80327 0.01698 181.81754

0.01708 88.76838 0.01708 135.49976 0.01708 162.95421 0.01708 181.05759

0.01716 84.68443 0.01716 131.84956 0.01716 160.69844 0.01716 180.03704

0.01725 80.27879 0.01725 127.59946 0.01725 157.93384 0.01725 178.68301

0.01733 75.54156 0.01733 122.62653 0.01733 154.52157 0.01733 176.89258

0.01743 70.47165 0.01743 116.77643 0.01743 150.26824 0.01743 174.51577
