# Supplementary material for: Damage evolution and constitutive model of the rock masses with non-penetrating cracks under repeated impact loading
Source: PLoS One. 2023 Sep 15;18(9):e0289022. doi: 10.1371/journal.pone.0289022 (PMC10503746; doi:10.1371/journal.pone.0289022)
Supplement: Attachment — Submitted filename: Response to Reviewers.docx [file pone.0289022.s002.docx]

Thank the editor for reviewing the manuscript and putting forward valuable suggestions. The following is our revision of the manuscript according to the expert review opinions, as well as some descriptions of the revision.

**Comments of reviewers**

**Question 1:** I recommend that the title of the manuscript be slightly changed. Instead of the term "cyclic-impact disturbances", which may be associated with fatigue tests, I suggest replacing it with the phrase "repeated impact loading". Generally speaking, the term "disturbances" does not fit the issue of the manuscript. I recommend replacing it with "loading" throughout the paper.

**Response 1:** Modifications have been made based on the suggestions made by the reviewers.

**Question 2:** There is no information in the manuscript about the number of material specimens made. In other words, how many specimens were used for quasi-static and high strain rate tests and how many test repetitions were made under given experimental conditions (section 2.1).

**Response 2:** Repeated impact tests by Split Hopkinson Pressure Bar are performed on non-penetrating cracked granite specimens with different prefabricated-crack inclination angles (0, 30, 45, 60, and 90°). Three specimens were selected for each working condition for testing. And it has been modified in the text.

**Question 3:** Fig. 1a - no axis of symmetry and no symbol indicating the diameter of a cylindrical specimen. In addition, prefabricated crack surface S2 was marked incorrectly in Fig. 1b.

**Response 3:** Based on the suggestions of the reviewers, Figure 1 in the text has been modified.

**Question 4:** Incorrect terminology: line 108 and 110, Fig. 2; "warhead", "spindle punch"; the commonly used term is "striker bar" or more accurately "cone-shaped striker". I also propose to replace the term "impact times" with "impact number".

**Response 4:** The terminology in the main text and Figure 2 have been modified.

**Question 5:** Line 103; "....SHPB device with a diameter of 50 mm...." diameter of what?

**Response 5:** The experiment used a separate SHPB device with a bar diameter of 50 mm. And it has been modified in the text.

**Question 6:** Line 117; "...with certain strength." Are you sure it's about "strength" and not "force"?

**Response 6:** In the test of repeated impact loading, rock samples were simultaneously applied with axial static stress *P*_0_ and dynamic impact loading *P*_d_ with certain force. It has been modified in the text.

**Question 7:** Line 118; force in MPa?

**Response 7:** axial static stress *P*_0_ = 8 MPa. It has been modified in the text.

**Question 8:** Please explain how the material specimen was loaded with an axial static force on the SHPB stand.

**Response 8:** Firstly, apply axial pressure to the rock sample at a rate of 0.5 MPa/s until the predetermined axial pressure value is reached and remains stable. Then repeatedly impact the rock specimen with the set impact pressure, adjusting the axial pressure value before each impact to ensure consistency with the set value. When the rock is damaged and the test is completed, record and save the data collected after each impact. Supplementary explanations have been provided in the text.

**Question 9:** Fig. 3 has already been presented in [27]. I believe that a literature reference should be added in the caption of the Figure.

**Response 9:** A reference has been added to the title of Figure 3.

**Question 10:** Fig. 4; Incorrectly marked wave profiles. The descriptions of the reflected and transmitted waves should be interchanged.

**Response 10:** According to the issues pointed out by the reviewers, the author has made modifications in Figure 4.

**Question 11:** line 162; I recommend adding a description of determining the dynamic deformation modulus, especially since in work [28] I could not find a description of determining the above-mentioned parameter.

**Response 11:** The weighted average of the secant modulus, first type secant modulus, and deformation modulus of the loading section is defined as the dynamic deformation modulus, which is used to reflect the compressive deformation characteristics of rocks during the dynamic loading stage. It has been modified in the text.

**Question 12:** Both quasi-static and dynamic strength tests were carried out using specimens with a length to diameter ratio of L/d = 2. According to the recommendations presented in the literature, dynamic tests of rocks, concrete and similar materials under the SHPB test conditions should be performed on a specimens with an L/d ratio of 1. This ratio is important because of the need to achieve an equilibrium stress state in the specimen during dynamic loading. This is the basic methodological requirement of the SHPB technique. Therefore, in order to recognize that the results presented in the manuscript are credible, the manuscript should be supplemented with an analysis of the state of equilibrium stress in the applied loading conditions. This is my most important remark, which should be taken into account in order to recognize that the developed constitutive model and the determined material parameters are correct.

**Response 12:** Based on the suggestions of the reviewers, an analysis of the equilibrium stress state under the applied loading conditions has been added to the manuscript. The transmitted wave and superimposed wave basically coincide and remain in the post-peak area for a certain period of time, indicating that the sample can achieve stress equilibrium conditions well during the loading process.

**Question 13:** The sentence (lines 166 and 167) is incomprehensible.

**Response 13:** The author has made revisions to the problematic areas in the article.

**Question 14:** The sentence (lines 184 and 185) is rather redundant as it does not refer to the content of the paragraph.

**Response 14:** The author has removed the redundant lines in this paragraph of the article.

**Question 15:** Line 430; statement "...their peak strength decreases with increased strain." is not true.

**Response 15:** "... their peak strength decreases as the number of repeated impacts increases." It has been modified in the text.

**Question 16:** Line 455; How was the strain rate value determined? From Fig. 4 it can be seen that the strain rate during the SHPB experiments was not constant. So what was the strain rate estimation procedure?

**Response 16:** During the experimental testing, the selected impact pressure is 0.18MPa, and the strain rate of the rock specimen at the first impact is calculated according to the following equation. In the test, the strain rate of the test piece is indirectly calculated by measuring the Strain gauge voltage on the incident rod and the transmission rod. The three-wave method calculation formula is as follows:

**Question 17:** Line 465 and 466; I believe that giving the peak strength value with an accuracy of two decimal places is a mistake. The SHPB technique does not allow for such exact determination of the stress in the specimen material.

**Response 17:** Modifications have been made based on the suggestions made by the reviewers.

**Question 18:** Line 490 and 491; statement "Higher coefficient of determination R2 indicates a better fitting effect" is obvious and redundant in the chapter where the conclusions are presented.

**Response 18:** The author has removed the redundant lines in this paragraph of the article.

**Question 19:** I am not an expert in English, but grammatical and editorial errors and incorrectly used phrases. etc., are abundant in the manuscript.

**Response 19:** The author has corrected some grammatical errors and spelling errors in the text.

Thank you again for the valuable revision suggestions provided by the expert reviewers.
